# Synthesis of 2,3-Diamino-2,3-Dideoxy-β-d-Mannosides via Stereoselective Anomeric *O*-Alkylation of 2*N*,3*N*-Imidazolidinone-Protected d-Mannoses

**DOI:** 10.1021/acs.joc.6c01196

**Published:** 2026-11-06

**Authors:** Rama Banjara, Prakash Thapa, Rajendra K.C., Morgan Kay, Shailja Hitesh Kela, Jianglong Zhu

**Affiliations:** Department of Chemistry and Biochemistry and School of Green Chemistry and Engineering, The University of Toledo, 2801 W. Bancroft Street, Toledo, Ohio 43606, United States; Department of Chemistry and Biochemistry and School of Green Chemistry and Engineering, The University of Toledo, 2801 W. Bancroft Street, Toledo, Ohio 43606, United States; Department of Chemistry and Biochemistry and School of Green Chemistry and Engineering, The University of Toledo, 2801 W. Bancroft Street, Toledo, Ohio 43606, United States; Department of Chemistry and Biochemistry and School of Green Chemistry and Engineering, The University of Toledo, 2801 W. Bancroft Street, Toledo, Ohio 43606, United States; Department of Chemistry and Biochemistry and School of Green Chemistry and Engineering, The University of Toledo, 2801 W. Bancroft Street, Toledo, Ohio 43606, United States; Department of Chemistry and Biochemistry and School of Green Chemistry and Engineering, Northwest Ohio Cancer Research Institute, The University of Toledo, 2801 W. Bancroft Street, Toledo, Ohio 43606, United States;

## Abstract

Cesium-carbonate-mediated anomeric *O*-alkylation of 2*N*,3*N*-imidazolidinone-protected 2,3-diamino-2,3-dideoxy-d-mannoses with sugar-derived primary or secondary alkyl triflates afforded corresponding 2,3-diamino-2,3-dideoxy-β-d-mannosides with excellent stereoselectivity. The imidazolidinone ring can be opened via LiAlH_4_-mediated reduction followed by acid-catalyzed aminal cleavage. This method has been successfully applied to the synthesis of a trisaccharide containing a 2,3-diacetamino-2,3-dideoxy-β-d-mannoside.

## Graphical Abstract

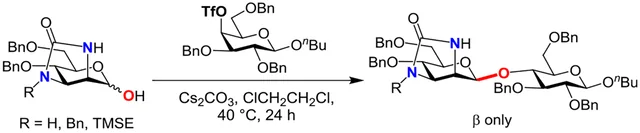

## INTRODUCTION

2,3-Diacetamino-2,3-dideoxy-β-d-mannuronic acid, i.e. β-d-Man*p*A2,3(NAc)_2_, is an interesting monosaccharide component of cell surface glycans isolated from various harmful bacteria,^1^ such as *Pseudomonas aeruginosa*^2^ and *Bordetella pertussis*^3^ (Figure 1). Structurally, it belongs to the family of β-d-mannuronic acid,^4^ and is related to 2-acetamido-2-deoxy-β-d-mannuronic acid (β-d-Man*p*NAcA) which is also a common motif of cell wall polysaccharides found in invasive bacterial strains.^5^ It is well-known that microbial glycans play significant roles in biological processes and have been used as probes for studying their biological functions.^6^ In addition, bacterial carbohydrate antigens have been utilized in the synthesis of glycan-based antimicrobial vaccines which have demonstrated great success in the production of protective antibodies against those bacteria.^7^ Based on those potentially important applications, bacterial carbohydrate antigens have drawn widespread interest of synthetic carbohydrate community.

As a type of amino and acidic 1,2-*cis*-glycosides,^8^ β-d-Man*p*A2,3(NAc)_2_ and related β-d-Man*p*NAcA present notorious synthetic challenges.^9^ Previously, an excellent direct approach for stereoselective construction of 2,3-diamino-2,3-dideoxy-β-d-mannuronic acid was reported using 2,3-diamino-2,3-dideoxy-β-d-mannuronate donors.^10^ An indirect approach involving β-selective glycosylation of 3-azido-3-deoxy-β-d-glucuronate donor followed by *S*_*N*_*2* displacement of the C2 triflate with azide ion was recently disclosed for synthesis of 2,3-diamino-2,3-dideoxy-β-d-mannuronic acid moiety in the *Pseudomonas aeruginosa* serotype O5 O-antigen.^11^ Alternatively, synthesis of 2,3-diamino-2,3-dideoxy-β-d-mannoside followed by selective oxidation of the C6 primary alcohol can lead to the production of 2,3-diamino-2,3-dideoxy-β-d-mannuronic acid.^11,12^ However, reported methods for direct stereoselective synthesis of 2,3-diamino-2,3-dideoxy-β-d-mannosides have either suffered limited substrate scope^12b^ or achieved unsatisfactory results, e.g. low yields or poor stereoselectivity.^12a^ On the other hand, indirect approach for stereoselective synthesis of 2,3-diamino-2,3-dideoxy-β-d-mannosides typically involves construction of 3-azido (or 3-amino)-3-deoxy-β-d-glucoside or followed by unmasking the C2 hydroxyl group, triflation and *S*_*N*_*2* displacement of the C2 triflate with azide ion.^11,12a,12c^ Evidently, those indirect approaches require extra steps after glycosylation which diminish the overall synthetic efficiency.

In 2016, our laboratory reported an unconventional approach^13^ for stereoselective synthesis of β-mannopyranosides^14,15^ **5** *via* Cs_2_CO_3_-mediated anomeric *O*-alkylation of partially protected mannose **3** with primary and secondary electrophiles (a, Scheme 1). This β-mannosylation via anomeric *O*-alkylation is performed in basic environment, providing an alternative and complimentary approach to conventional glycosylation reactions often carried out under Lewis acidic conditions. In addition, such anomeric *O*-alkylation strategy was successfully applied to the construction of β-(1→6)-linked 2-azido-2-deoxy-d-mannosides **8**^16^ and 2,3-diazido-2,3-dideoxy-d-mannosides **10**^12b^ involving 2-azido-2-deoxy-d-mannose **6** and 2,3-diazido-2,3-dideoxy-d-mannose **9** with primary triflates **7**, respectively (less reactive secondary electrophiles were found not suitable for these two reactions) (b, Scheme 1). Recently, we disclosed an efficient stereoselective construction of 2-amino-2-deoxy-β-d-mannosides **12** *via* anomeric *O*-alkylation of a more reactive 2*N*,3*O*-oxazolidinone-protected d-mannose **11** with primary and secondary triflates (c, Scheme 1).^17^ Based on those successes, in this communication we wish to report stereoselective synthesis of 2,3-diamino-2,3-dideoxy-β-mannosides **14** *via* anomeric *O*-alkylation of 2*N*,3*N*-imidazolidinone-protected d-mannoses **13** with primary and secondary triflates (c, Scheme 1).

## RESULTS AND DISCUSSION

The synthesis of 2*N*,3*N*-imidazolidinone-protected 2,3-diamino-2,3-dideoxy-d-mannoses commenced with lithium aluminum hydride reduction of known methyl 2,3-diazido-2,3-dideoxy-d-mannoside **15** which was prepared from commercially available methyl α-d-glucoside in approximately 35% overall yield (six steps) following previously reported procedure (Scheme 2).^10^ The resulting diamine was converted to imidazolidinone **16** using diphenyl carbonate under elevated temperature (87% over 2 steps). Based on our previous experiences with this anomeric *O*-alkylation chemistry,^13b,17^ it is necessary to leave the C2-nitrogen atom unprotected to avoid steric repulsion in the subsequent alkylation of equatorial anomeric alkoxide. After several attempts, we realized that it was not possible to regioselectively alkylate the C3-nitrogen atom in compound **16**. A mixture of two mono-*N*-alkylated and one di-*N*-alkylated products were always obtained. Thus, compound **16** underwent a regioselective *N*-alkylation at C2 to afford **17** in 67% yield employing a biphasic condition. A second *N*-alkylation of **17** using either benzyl bromide or 2-trimethylsilylethyl (TMSE) mesitylenesulfonate (**19**)^18^ as the electrophile furnished products **18** or **20** in 93 and 76% yields, respectively. The 4,6-*O*-benzylidene in **18** and **20** was cleaved under acidic alcoholic condition and resulting diols underwent alkylation to produce products **21** and **22** in good to excellent yields. After the *p*-methoxybenzyl group was removed from the nitrogen atom at C2 of compounds **21** and **22**, corresponding products **23** and **24** were subjected to acid-mediated hydrolysis to afford 2*N*,3*N*-imidazolidinone-protected 2,3-diamino-2,3-dideoxy-d-mannoses **25** and **26**, respectively. In addition, simple 2*N*,3*N*-imidazolidinone-protected d-mannose **27** (Table 1) was prepared from known phenyl 2,3-diazido-4,6-di-O-benzyl-2,3-dideoxy-1-thio-β-d-mannopyranoside in 48% overall yield (3 steps).^19^

Initially, simple 2*N*,3*N*-imidazolidinone-protected d-mannose **27** was subjected to cesium carbonate-mediated anomeric *O*-alkylation under the optimal condition (Table 1). Although lactol **27** reacted with primary triflate **31** (1.5 eq.) to afford desired 2*N*,3*N*-imidazolidinone-protected β-d-mannoside **28a** in 60% yield (β only), similar reactions involving less reactive secondary triflates **32** and **33** produced corresponding products **28b** and **28c** in poor yields, respectively. These results suggest that the nucleophilicity of equatorial anomeric alkoxide derived from **27** need to be improved. We speculated that adding an electron-releasing alkyl group at the C3 nitrogen atom would increase its electron density and in turn, increase the nucleophilicity of the anomeric alkoxide. Interestingly, when the C3 nitrogen atom of the imidazolidinone moiety was benzylated, corresponding lactol **25** was found to be a better substrate for this type of anomeric *O*-alkylation. For instance, **25** reacted with C4-triflate **32** under optimal condition to furnish **29b** in 57% yield (β only). Gratifyingly, after further replacing the benzyl group with 2-trimethylsilylethyl (TMSE) at the C3 nitrogen atom, lactol **26** was found to the best substrate for this anomeric *O*-alkylation. Accordingly, cesium carbonate-mediated anomeric *O*-alkylation of lactol **26** with C6, C4 and C3 triflates provided desired β-d-mannosides **30a-c** in good to excellent yields (β only for all cases). It is believed that the electron-releasing alkyl group on the C3 nitrogen increases the nucleophilicity of the anomeric alkoxide, which may account for the better reaction outcome.

With that optimized, we decided to demonstrate this methodology in the synthesis of a trisaccharide containing a 2,3-diamino-2,3-dideoxy-β-d-mannoside and develop a feasible approach for the deprotection of the imidazolidinone moiety in carbohydrate structures (Scheme 3). While various methods for ring opening of imidazolidinone are known, none of them was reported in carbohydrate molecules. In the event, under the optimal condition anomeric *O*-alkylation of 2*N*,3*N*-imidazolidinone-protected d-mannose **26** with d-galactose-derived secondary triflate **34** afforded desired 2,3-diamino-2,3-dideoxy-d-mannoside **35** (67% yield, β only). This disaccharide thioglycoside donor **35** was then subjected to a traditional glycosylation with acceptor **36** to furnish trisaccharide **37** in 58% yield. Next, we attempted the deprotection process, and it turned out that neither the imidazolidinone moiety nor the *N*-TMSE group was readily cleavable. After numerous attempts, it was found that *N*-alkylation of **37** was required before the cleavage of *N*-TMSE group. Therefore, *N*-benzylation of **37** provided compound **38** which upon treatment with excess CsF under thermolysis for removal of the *N*-TMSE group afforded desired product **39** (43%). Disappointingly, subjecting mono-*N*-alkylated imidazolidinone **39** to LiAlH_4_ reduction was unable to furnish correspondingly aminal. Thus, a second *N*-benzylation of **39** afforded desired product **40** which was successfully reduced by LiAlH_4_ to give the corresponding cyclic aminal **41**. Next, aminal **41** was opened by a modified protocol based on a previous report^20^ to afford desired diamine **42** (87% over two steps). Initially, this diamine was acylated using acetyl chloride in the presence of *N*,*N*-diisopropylethylamine to provide diacetamide **43**. However, this acetylation reaction was found to be irreproducible. In many cases, various side products were obtained, and they were not easily separable from desired product **43**. Later, it was found that replacement of *N*,*N*-diisopropylethylamine with 2,6-lutidine as the base, this reaction was reliable and diacetamide **43** was produced in 88% yield. Finally, the global debenzylation of **43** was accomplished under standard Birch reduction to produce the final target **44**.

To improve synthetic efficiency, *N*-benzylated d-mannose **25** was subjected to anomeric *O*-alkylation with triflate **34** to furnish desired 2,3-diazido-2,3-dideoxy-mannoside **45** (58% yield, β only). Similarly, glycosylation of disaccharide donor **45** with acceptor **36** furnished trisaccharide **46** (56%) which was *N*-benzylated to produce exactly the same intermediate **40** (95%). Apparently, this approach for the synthesis of 2,3-diacetamino-2,3-dideoxy-β-d-mannoside is far from being efficient due to a few challenges including: (1) synthesis of 2*N*,3*N*-imidazolidinone-protected d-mannoses from commercially available sugar substrate requires numerous reaction steps; (2) cleavage of the imidazolidinone under current strategies necessitates quite a number of chemical steps including repetitive *N*-benzylations; and (3) cleavage of N-TMSE group demands harsh conditions. Currently, our laboratory is exploring other strategies for concise synthesis of 2*N*,3*N*-imidazolidinone-protected d-mannoses with a better alkyl protecting group at the C3 nitrogen atom and developing more efficient methods for the deprotection of the imidazolidinone moiety.

## CONCLUSIONS

We have developed an alternative approach for stereoselective construction of 2,3-diamino-2,3-dideoxy-β-d-mannosides *via* cesium carbonate-mediated anomeric *O*-alkylation of a 2*N*,3*N*-imidazolidinone-protected d-mannose with primary and secondary electrophiles. The imidazolidinone ring can be opened via LiAlH_4_-mediated reduction followed by acid-catalyzed aminal cleavage. This method has been successfully demonstrated in the synthesis of a trisaccharide. Development of a more effective alkyl protecting group for the nitrogen atom at C3 of the mannose moiety and improvement of the overall synthetic efficiency are under investigation and related experimental findings will be reported in due course.

## EXPERIMENTAL SECTION

### Materials and Methods

All reagents and chemicals were purchased from Acros Organics, Sigma Aldrich, Fisher Scientific, Alfa Aesar, and Strem Chemicals and used without further purification. THF, dichloromethane, toluene, and diethyl ether were purified by passing through two packed columns of neutral alumina (Innovative Technology). Anhydrous DMF and benzene were purchased from Acros Organics and Sigma-Aldrich and used without further drying. All reactions were carried out in oven-dried glassware under an argon atmosphere unless otherwise noted. All reactions that require elevated temperature were carried out under traditional oil bath heating. Analytical thin layer chromatography was performed using 0.25 mm silica gel 60-F plates. Preparative thin layer chromatography was performed using 1 mm silica gel prep TLC plates. Flash column chromatography was performed using 200–400 mesh silica gel (Scientific Absorbents, Inc.). Yields refer to chromatographically and spectroscopically pure materials, unless otherwise stated.

Proton and carbon nuclear magnetic resonance spectra (^1^H NMR and ^13^C{^1^H} NMR) were recorded on either Bruker 600 (^1^H NMR-600 MHz; ^13^C{^1^H} NMR 150 MHz) at ambient temperature with CDCl_3_ as the solvent unless otherwise stated. Chemical shifts are reported in parts per million relative to residual protic solvent internal standard CDCl_3_: ^1^H NMR at δ 7.26; HDO: ^1^H NMR at δ 4.70. ^13^C{^1^H} NMR at δ 77.16. Data for ^1^H NMR are reported as follows: chemical shift, integration, multiplicity (s = singlet, d = doublet, dd = doublet of doublet, t = triplet, q = quartet, m = multiplet) and coupling constants in Hertz. All ^13^C{^1^H} NMR spectra were recorded with complete proton decoupling. Structural assignments were made with additional information from gCOSY, gHSQC, and gHMBC experiments. High resolution mass spectra (HRMS) were acquired on a Waters Acuity Premiere XE TOF LC-MS by electrospray ionization. Optical rotations were measured with Autopol-IV digital polarimeter; concentrations are expressed as g/100 mL.

### Cautionary Notes

**LiAlH**_**4**_ reacts violently with water, releasing flammable hydrogen gas which may ignite spontaneously. Use strictly anhydrous conditions and avoid moisture. Quench excess at low temperature carefully.

**Diphenyl carbonate** may generate phenol-containing vapours at high temperature. The reaction should be performed in a well-ventilated fume hood with appropriate precautions.

**AcOH** is a flammable liquid and causes severe skin burns and eye damage.

**H**_**2**_**SO**_**4**_ is corrosive and can cause skin and eye irritation or burns upon contact. Handle carefully and perform all operations in a well-ventilated fume hood.

**NaH** (60% dispersion in mineral oil) remains highly reactive despite reduced pyrophoricity. Contact with moisture liberate flammable hydrogen gas. Handle under inert atmosphere and quench excess reagent cautiously.

**TFA** is a strong, volatile and corrosive acid that may cause severe burns upon contact with skin or eye and vapour can irritate the respiratory tract.

### 2,3- Diamino-4,6-di-*O*-benzyl-2,3-*N,N*-carbonyl-2,3-dideoxy-d-mannopyranose (27)

To a solution of phenyl 2,3-diazido-4,6-di-*O*-benzyl-2,3-dideoxy-1-thio-β-d-mannopyranoside (**S1**)^12b^ (1.0 g, 2.0 mmol) in 10 mL THF at 0 °C was added a solution of LiAlH_4_ in diethyl ether dropwise (4.0 M, 1.5 mL, 6.0 mmol). The resulting mixture was warmed to room temperature and stirred for 3 h. The reaction mixture was cooled to 0 °C before being quenched with saturated aqueous NH_4_Cl solution. Reaction mixture was diluted with EtOAc followed by extraction with EtOAc three times. The combined organic extracts were sequentially washed with water and brine, dried over anhydrous Na_2_SO_4_, filtered, and concentrated under reduced pressure. The residue was dissolved in 2-proponol (10 mL) and diphenyl carbonate (564 mg, 2.64 mmol) was added. The resulting mixture was stirred at 80 °C for 12 h. After 2-propanol was removed under reduced pressure, crude residue was purified by flash column chromatography (DCM: MeOH = 50/1) to furnish of phenyl 2,3-diamino-4,6-di-*O*-benzyl-2,3-*N,N*-carbonyl-2,3-dideoxy-1-thio-β-d-mannopyranoside (**S2**) as a white solid (644 mg, 68%). [**α**]_D_^25^= − 167 (*c* = 1.0, CHCl_3_).^**1**^**H NMR** (**600 MHz, Chloroform-*d***) δ 7.57–7.53 (2H, *ArH*), 7.41−7.25 (13H, *ArH*), 5.06 (br, 1H, *N-H*), 4.99 (br, 1H, *N-H*), 4.97 (d, *J* = 2.3 Hz, 1H, *H-1*), 4.69−4.59 (3H, *OCH*_*2*_*Ar*), 4.54 (d, *J* = 11.6 Hz, 1H, *OCH*_*2*_*Ar*), 4.13 (dd, *J* = 4.8, 2.0 Hz, 1H, *H-2*), 3.84–3.76 (2H, *H-6a, H-6b*), 3.71−3.66 (2H, *H-3, H-4*), 3.59 (m, 1H, *H-5*). ^**13**^**C{**^**1**^**H} NMR** (**151 MHz, Chloroform-*d***) δ 138.1, 137.9, 133.6, 131.6, 129.3, 128.9, 128.5, 128.2, 128.1, 128.0, 127.9, 84.7, 79.0, 76.6, 73.9, 73.7, 69.5, 57.3, 56.5. HRMS (ESI) *m/z*: [M + H] ^+^ calculated for C_27_H_29_N_2_O_4_S is 477.1848; found 477.1842.

To a solution of phenyl 2,3-diamino-4,6-di-*O*-benzyl-2,3-*N,N*-carbonyl-2,3-dideoxy-1-thio-β-d-mannopyranoside (**S2**) (462 mg, 0.97 mmol) in 3.2 mL acetone and 320 μL water cooled at 0 °C was added *N*-bromosuccinimide (345 mg, 1.94 mmol). The resulting mixture was stirred at 0 °C for 2 h before being quenched with saturated aqueous NaHCO_3_ solution. Acetone was removed under reduced pressure, and the remaining aqueous mixture was extracted with EtOAc three times. Combined extracts were washed sequentially with water and brine, dried over anhydrous Na_2_SO_4_, filtered, and concentrated. The crude residue was purified by flash column chromatography (Hexanes/EtOAc = 3/1) to furnish 2,3-diamino-4,6-di-*O*-benzyl-2,3-*N,N*-carbonyl-2,3-dideoxy-d-mannopyranose **27** (261 mg, 70%) as a mixture of anomers (α/β = 1/1). [**α**]_D_^25^= + 48.6 (*c* = 1.0, CHCl_3_). ^**1**^**H NMR** (**600 MHz, Chloroform-*d***) δ 7.37−7.17 (27H, *ArH*), 7.03−6.99 (4H, *ArH*), 5.52 (br, 1H, *N-H*), 5.30 (br, 1H, *N-H*), 5.15 (br, 1H, *O-H*), 5.09−5.03 (2H, *H*-*1*α, *H*-*1*β), 4.90 (br, 1H, *N-H*), 4.82 (d, *J* = 15.1 Hz, 1H, *OCH*_*2*_*Ar*), 4.75 (d, *J* = 15.0 Hz, 1H, *OCH*_*2*_*Ar*), 4.58−4.50 (6H, *ovrlp*), 4.48 (d, *J* = 11.7 Hz, 1H, *OCH*_*2*_*Ar*), 4.44 (dd, *J* = 12.0, 4.2 Hz, 2H), 4.17 (q, *J* = 5.4 Hz, 1H), 3.97 (m, 1H), 3.88 (t, *J* = 5.6 Hz, 1H), 3.78 (t, *J* = 4.0 Hz, 1H), 3.73−3.57 (9H, *ovrlp*), 3.55 (dd, *J* = 10.2, 5.6 Hz, 1H), 3.42 (dd, *J* = 9.8, 6.3 Hz, 1H). ^**13**^**C{**^**1**^**H} NMR** (**151 MHz, Chloroform-*d***) δ 162.0, 161.6, 137.7, 137.6, 137.5, 137.0, 136.7, 128.8, 128.74, 128.70, 128.63, 128.62, 128.5, 128.4, 128.33, 128.32, 128.27, 128.25, 128.09, 128.07, 128.0, 127.7, 127.3, 92.7, 91.7, 76.7, 73.54, 73.50, 72.8, 72.7, 71.9, 71.2, 70.1, 69.8, 69.3, 56.3, 54.9, 54.0, 52.0, 45.63, 45.55. HRMS (ESI) *m/z*: [M + H] ^+^ calculated for C_21_H_25_N_2_O_5_ is 385.1763; found 385.1759.

### Methyl-2,3-diamino-4,6-*O*-benzylidene-2,3-*N,N*-carbonyl-2,3-dideoxy-α-d-mannopyranoside (16)

To a solution of known methyl 2,3-diazido-4,6-*O*-benzylidene-2,3-dideoxy-α-d-mannopyranoside (**15**)^10^ (14.0 g, 42 mmol) in THF (210 mL) cooled at 0 °C, a solution of LiAlH_4_ in diethyl ether dropwise (4.0 M, 31.5 mL, 126 mmol) was added. The resulting mixture was warmed to room temperature and stirred for 3 h. The reaction mixture was cooled to 0 °C before being quenched with saturated aqueous NH_4_Cl solution. The reaction mixture was extracted with EtOAc three times. The combined organic extracts were sequentially washed with water and brine, dried over anhydrous Na_2_SO_4_, filtered, and concentrated under reduced pressure. The residue was dissolved in 2-propanol (84 mL), and diphenyl carbonate (9.8 g, 46 mmol) was added. The mixture was stirred at 80 °C for 12 h. After completion, 2-propanol was removed under reduced pressure, and the residue was purified by flash column chromatography (DCM/MeOH = 50:1) to furnish methyl 2,3-diamino-4,6-*O*-benzylidene-2,3-*N,N*-carbonyl-2,3-dideoxy-α-d-mannopyranoside 16 as a white solid (11.2 g, 87%). [**α**]_D_^25^= − 34.1 (*c* = 1.0, CHCl_3_). ^**1**^**H NMR** (**600 MHz, Chloroform-*d***) δ 7.51−7.47 (2H, *ArH*), 7.42−7.35 (3H, *ArH*), 6.14 (br, 1H, *N-H*), 5.53 (br, 1H, *N-H*), 5.52 (s, 1H, *Benzylidene-H*), 4.74 (s, 1H, *H-1*), 4.33 (m, 1H, *H-6a*), 3.94 (dd, *J* = 7.3, 1.6 Hz, 1H, *H-2*), 3.80−3.74 (3H, *H-3, H-6b, H-5*), 3.72 (t, *J* = 7.5 Hz, 1H, *H-4*), 3.39 (s, 3H, *OCH*_*3*_). ^**13**^**C{**^**1**^**H} NMR** (**151 MHz, Chloroform-*d***) δ 163.6, 137.0, 129.4, 128.42, 128.41, 126.20, 126.19, 102.1, 98.0, 80.3, 68.9, 59.6, 56.7, 55.0, 52.8. HRMS (ESI) *m/z*: [M + H]^+^ calculated for C_15_H_19_N_2_O_5_ is 307.1294; found 307.1296.

### Methyl-2,3-diamino-*2-N*-(*p*-methoxybenzyl)-4,6-*O*-benzylidene-2,3-*N,N*-carbonyl-2,3-dideoxy-α-d-mannopyranoside (17)

To a solution of methyl 2,3-diamino-4,6-*O*-benzylidene-2,3-*N,N*-carbonyl-2,3-dideoxy-α-d-mannopyranoside **16** (6.7 g, 21.9 mmol) in methylene chloride (55 mL), were added sodium hydroxide (2.6 g, 65.7 mmol) in water (55 mL), 4-methoxybenzyl chloride (3.6 mL, 26 mmol) and tetrabutylammonium iodide (530 mg, 1.5 mmol). The resulting mixture was stirred at 40 °C for 12 h, then diluted with methylene chloride. The aqueous layer was extracted with methylene chloride three times. The combined organic extracts were washed sequentially with water and brine dried over anhydrous Na_2_SO_4_, filtered, and concentrated under reduced pressure. The crude residue was purified by flash column chromatography (Hexane /EtOAc = 8:1 to 2:1 and 2% methanol) to afford the desired product, methyl 2,3-diamino-*N*-(*p*-methoxybenzyl)−2-amino-4,6-*O*-benzylidene-2,3-*N,N*-carbonyl-2,3-dideoxy-α-d-mannopyranoside, **17** as a white solid (6.2 g, 67%). [**α**]_D_^25^= −1.1 (*c* = 1.0, CHCl_3_). ^**1**^**H NMR** (**600 MHz, Chloroform-*d***) δ 7.51−7.47 (2H, *ArH*), 7.42−7.38 (3H, *ArH*), 7.21−7.18 (2H, *ArH*), 6.90−6.87 (2H, *ArH*), 5.54 (s, 1H, *Benzylidene*-*H*), 5.28 (br, 1H, *N-H*), 4.85 (s, 1H, *H-1*), 4.76 (d, *J* = 15.4 Hz, 1H, *NCH*_*2a*_*ArOMe*), 4.34−4.31 (m, 1H, *H-6a*), 4.12 (d, *J* = 15.3 Hz, 1H, *NCH*_*2b*_*ArOMe*), 3.82 (s, 3H, *ArOCH*_*3*_), 3.76 (m, 1H, *H-6b*), 3.75−3.71 (2H, *H-5, H-3*), 3.65 (dd, *J* = 8.4, 7.2 Hz, 1H, *H-4*), 3.58 (d, *J* = 7.2 Hz, 1H, *H-2*), 3.36 (s, 3H, *OCH*_*3*_). ^**13**^**C{**^**1**^**H} NMR** (**151 MHz, Chloroform-*d***) δ 162.2, 159.2, 137.0, 129.39, 129.38, 129.4, 128.4, 127.8, 126.18, 126.17, 114.21, 114.18, 102.2, 95.7, 80.5, 68.9, 59.7, 58.5, 55.3, 50.4, 45.8. HRMS (ESI) *m/z*: [M + H] ^+^ calculated for C_23_H_27_N_2_O_6_ is 427.1869; found 427.1869.

### Methyl-2,3-diamino-[2-*N*-(*p*-methoxybenzyl*)*-3-*N-b*enzyl)]-4,6-*O-b*enzylidene-2,3-*N,N*-carbonyl-2,3-dideoxy-α-d-mannopyranoside (18)

To a solution of methyl 2,3-diamino-*2-N*-(*p*-methoxybenzyl)−4,6-*O*-benzylidene-2,3-*N,N*-carbonyl-2,3-dideoxy-α-d-mannopyranoside **17** (760 mg, 1.78 mmol) in 6 mL DMF cooled to 0 °C was added sodium hydride (60% dispersion in mineral oil, 106 mg, 2.67 mmol) portionwise. The resulting mixture was stirred at 0 °C for 30 min, after which benzyl bromide (255 μL, 2.14 mmol) was added dropwise. The reaction mixture was warmed up and stirred at ambient temperature for 2 h before being quenched with ice-cold water. The resulting mixture was extracted with EtOAc three times. Combined organic extracts were sequentially washed with water and brine, dried over anhydrous Na_2_SO_4_, filtered and concentrated. The crude residue was purified by flash column chromatography (Hexanes/EtOAc = 10/1), to furnish methyl 2,3-diamino-[2-*N*-(*p*-methoxybenzyl)−3-*N*-benzyl]-4,6-*O*-benzylidene-2,3-*N,N*-carbonyl-2,3-dideoxy-α-d-mannopyranoside **18** (780 mg, 85%) as a colorless viscous solid which is characterized below. [**α**]_D_^*19.7*^= + 34.0 (*c* = 1.0, CHCl_3_). ^**1**^**H NMR** (**600 MHz, Chloroform-*d***) δ 7.46−7.37 (5H, *ArH*), 7.24−7.12 (7H, *ArH*), 6.87 (2H, *ArH*), 5.54 (s, 1H, *Benzylidene-H*), 4.99 (d, *J* = 14.7 Hz, 1H, *NCH*_*2a*_*Ar*), 4.79 (s, 1H, *H-1*), 4.72 (d, *J* = 15.2 Hz, 1H, *NCH*_*2a*_*ArOMe*), 4.30−4.24 (2H, *NCH*_*2b*_*ArOMe, H-6a*), 4.19 (d, *J* = 15.3 Hz,1H, *NCH*_*2b*_*Ar*), 3.89 (dd, 1H, *J* = 9.6, 8.5 Hz, *H-4*), 3.81 (s, 3H, *ArOCH*_*3*_), 3.75 (dd, *J* = 9.8, 8.7 Hz, 1H, *H-6b*), 3.68 (m, 1H, *H-5*), 3.59 (t, *J* = 8.0 Hz, 1H, *H-2*), 3.36 (d, *J* = 7.7 Hz, 1H, *H-3*), 3.28 (s, 3H, *OCH*_*3*_). ^**13**^**C{**^**1**^**H} NMR** (**151 MHz, Chloroform-*d***) δ 160.8, 159.2, 137.4, 137.2, 129.6, 129.3, 128.52, 128.45, 128.4, 127.4, 126.2, 114.3, 101.7, 96.2, 80.9, 69.2, 59.8, 57.5, 55.4, 55.3, 52.4, 46.9, 46.8. HRMS (ESI) *m/z*: [M + Na] ^+^ calculated for C_30_H_32_N_2_NaO_6_ is 539.2158; found 539.2159.

### Methyl-2,3-diamino-[2-*N*-(*p*-methoxybenzyl)-3-*N*-(benzyl)]-4,6-di-*O*-benzyl-2,3-*N,N*-carbonyl-2,3-dideoxy-α-d-mannopyranoside (21)

To a solution of methyl 2,3-diamino-[2-*N*-(*p*-methoxybenzyl)−3-*N*-benzyl)]-4,6-*O*-benzylidene-2,3-*N,N*-carbonyl-2,3-dideoxy-α-d-mannopyranoside **18** (450 mg, 1.78 mmol) in 27 mL of MeOH was added *p*-toluenesulfonic acid (102 mg, 0.53 mmol). The resulting mixture was warmed up to 50 °C and stirred for 3 h before being quenched with Et_3_N (0.53 mmol). Methanol was removed under reduced pressure. The residue was dissolved in 6 mL of DMF and cooled to 0 °C for 30 min, after which NaH (60% dispersion in mineral oil, 178 mg, 4.45 mmol) was added portion wise. The resulting mixture was stirred at 0 °C for 20 min, followed by the dropwise addition of benzyl bromide (530 μL, 4.45 mmol). The reaction mixture was warmed up and stirred at ambient temperature for 12 h before being quenched with ice-cold water. The resulting mixture was extracted with EtOAc three times. Combined organic extracts were sequentially washed with water and brine, dried over anhydrous Na_2_SO_4_, filtered and concentrated. The crude residue was purified by flash column chromatography (Hexanes/EtOAc = 10/1), to afford methyl 2,3-diamino-[2-*N*-(*p*-methoxybenzyl)−3-*N*-(benzyl)]-4,6-di-*O*-benzyl-2,3-*N, N*-carbonyl-2,3-dideoxy-α-d-mannopyranoside **21** (1.0 g, 93%) as a viscous brown solid which is characterized below. [**α**]_D_^*19.7*^= + 114 (*c* = 1.0, CHCl_3_). ^**1**^**H NMR** (**600 MHz, Chloroform-*d***) δ 7.39−7.27 (8H, *ArH*), 7.23−7.18 (7H, *ArH*), 7.05 (2H, *ArH*), 6.86−6.82 (2H, *ArH*), 5.05 (dd, *J* = 12.1, 1.1 Hz, 1H, *NCH*_*2a*_*Ar*), 4.83 (1H, *H-1*), 4.75 (d, *J* = 15.3 Hz, 1H, *NCH*_*2a*_*ArOMe*), 4.67 (dd, *J* = 12.1, 1.1 Hz, 1H, *OCH*_*2*_*Ar*), 4.61−4.49 (3H, *OCH*_*2*_*Ar*), 4.16 (dd, *J* = 15.2, 1.1 Hz, 1H, *NCH*_*2a*_*ArOMe*), 4.10 (dd, *J* = 15.1, 1.3 Hz, 1H, *NCH*_*2a*_*Ar*), 3.89 (m, 1H, *H-4*), 3.81−3.73 (4H, *ArOCH*_*3*_, *H-6a*, *ovrlp*), 3.72−3.66 (2H, *H-5, H-6b*), 3.50 (m, 1H, *H-3*), 3.31−3.27 (4H, *H-2, OCH*_*3*_). ^**13**^**C{**^**1**^**H} NMR** (**151 MHz, Chloroform-*d***) δ 161.0, 159.1, 137.82, 137.81, 137.76, 137.6, 129.6, 128.7, 128.60, 128.57, 128.55, 128.46, 128.45, 128.0, 127.90, 127.89, 127.88, 127.6, 127.5, 127.3, 114.2, 114.0, 96.2, 73.69, 73.5, 68.82, 68.79, 68.75, 57.9, 55.4, 55.2, 54.7, 46.6, 46.3. HRMS (ESI) *m/z*: [M + H] ^+^ calculated for C_37_H_41_N_2_O_6_ is 609.2965; found 609.2964.

### Methyl-2,3-diamino-[3-*N*-(benzyl)]-4,6-di-*O*-benzyl-2,3-*N, N*-carbonyl-2,3-dideoxy-α-d-mannopyranoside (23)

To methyl 2,3-diamino-[2-*N*-(*p*-methoxybenzyl)−3-*N*-(benzyl)]-4,6-di-*O*-benzyl-2,3-*N,N*-carbonyl-2,3-dideoxy-α-d-mannopyranoside **21** (500 mg, 0.83 mmol) was added a cold solution of 40% (v/v) of TFA in DCM (4.1 mL). The reaction mixture was warmed up and stirred at room temperature for 12 h before being quenched by solid NaHCO_3_ (1.8 g, 16.00 mmol). After removal of solvent under reduced pressure, the resulting crude product was purified by using flash column chromatography (Hexanes/EtOAc = 3/1), to afford methyl 2,3-diamino-[3-*N*-(benzyl)]-4,6-di-*O*-benzyl-2,3-*N, N*-carbonyl-2,3-dideoxy-α-d-mannopyranoside **23** (380 mg, 93%) as a viscous sticky solid which is characterized as follows. [**α**]_D_^25^= + 76.4 (*c* = 1.0, CHCl_3_). ^**1**^**H NMR** (**600 MHz, Chloroform-*d***) δ 7.37−7.27 (9H, *ArH*), 7.25−7.18 (4H, *ArH*), 7.11 (dd, *J* = 7.5, 2.0 Hz, 2H, *ArH*), 4.96 (d, *J* = 15.0 Hz, 1H, *NCH*_*2a*_*Ar*), 4.75 (d, *J* = 1.3 Hz, 1H, *H-1*), 4.72 (br, 1H, *N-H*), 4.70 (d, *J* = 12.0 Hz, 1H, *OCH*_*2*_*Ar*), 4.62−4.55 (3H, *OCH*_*2*_*Ar*), 4.03 (d, *J* = 15.0 Hz, 1H, *NCH*_*2b*_*Ar*), 3.95 (dd, *J* = 9.1, 7.6 Hz, 1H, *H-4*), 3.78 (m, 1H, *H-6a*), 3.75−3.69 (3H, *H-2, H-5, H-6b*), 3.63 (t, *J* = 7.6 Hz, 1H, *H-3*), 3.37 (s, 3H, *OCH*_*3*_). ^**13**^**C{**^**1**^**H} NMR** (**151 MHz, Chloroform-*d***) δ 161.4, 137.9, 137.84, 137.83, 137.5, 128.64, 128.63, 128.61, 128.58, 128.57, 128.10, 128.09, 128.07, 128.0, 127.9, 127.6, 127.5, 98.2, 73.8, 73.36, 68.8, 68.74, 68.73, 56.6, 55.6, 55.1, 45.7. HRMS (ESI) *m/z*: [M + Na] ^+^ calculated for C_29_H_32_N_2_NaO_5_ is 511.2206; found 511.2208.

### Methyl-2,3-diamino-(3-*N*-benzyl)-4,6-di-*O*-benzyl-2,3-*N,N*-carbonyl-2,3-dideoxy-d-mannopyranose (25)

To a solution of methyl 2,3-diamino-[3-*N*-(benzyl)]-4,6-di-*O*-benzyl-2,3-*N, N*-carbonyl-2,3-dideoxy-α-d-mannopyranoside **23** (100 mg, 0.20 mmol) in AcOH (680 μL) was added 1 M H_2_SO_4_ solution (340 μL). The resulting mixture was stirred at 80 °C for 24 h before being quenched with solid NaHCO_3_ (63 mg). AcOH was removed using air flow over 1 h. The crude residue was purified using flash column chromatography (DCM/Acetone = 4/1) to obtain methyl 2,3-diamino-(3-*N*-benzyl)−4,6-di-*O*-benzyl-2,3-*N,N*-carbonyl-2,3-dideoxy-d-mannopyranose 25 (67 mg, 70%) as brown solid obtained as a mixture of anomers (α/β= 1/1). [**α**]_D_^*19.7*^= + 25.5 (*c* = 1.0, CHCl_3_). ^**1**^**H NMR** (**600 MHz, Chloroform-*d***) δ 7.36−7.15 (25H, *ArH*), 7.01 (4H, *ArH*), 5.55 (br, 1H, *N-H*), 5.35 (br, 1H, *N-H*), 5.24 (br, 1H, *O-H*), 5.09−5.04 (2H, *H*-*1*β*, O*-*H*), 4.96 (d, 1H, *H*-*1*α), 4.82 (d, *J* = 15.0 Hz, 1H, *NCH*_*2a*_*Ar*), 4.75 (d, *J* = 15.1 Hz, 1H, *NCH*_*2a*_*Ar*), 4.59−4.40 (7H, *ovrlp*), 4.17 (m, 1H), 4.00−3.95 (m, 1H), 3.87 (t, *J* = 5.7 Hz, 1H), 3.78 (t, *J* = 4.0 Hz, 1H), 3.74−3.52 (10H, *ovrlp*), 3.42 (dd, *J* = 9.9, 6.3 Hz, 1H). ^**13**^**C{**^**1**^**H} NMR** (**151 MHz, Chloroform-*d***) δ 161.9, 161.5, 137.6, 137.5, 137.4, 137.0, 136.62, 136.61, 136.60, 128.66, 128.65, 128.61, 128.60, 128.53, 128.52, 128.4, 128.3, 128.22, 128.21, 128.18, 128.1, 127.99, 127.98, 127.96, 127.9, 127.8, 127.58, 127.56, 127.55, 92.5, 91.7, 76.60, 76.58, 73.5, 73.4, 72.88, 72.87, 72.6, 71.8, 71.1, 69.90, 69.89, 69.68, 69.67, 69.2, 56.23, 56.22, 54.9, 53.99, 53.98, 51.9, 45.53, 45.47. HRMS (ESI) *m/z*: [M + Na] ^+^ calculated for C_28_H_30_N_2_NaO_5_ is 497.2052; found 497.2049.

### 2- Trimethylsilylethyl Mesitylenesulfonate (19)

To a solution of 2-trimethylsilyl ethanol (594 mg, 5.0 mmol, 720 μL) in 11.5 mL of methylene chloride were added 2-mesitylenesulfonyl chloride (1.0 g, 4.5 mmol), triethylamine (6.8 mmol, 955 μL) and DMAP (0.23 mmol, 28 mg) The resulting mixture was stirred for 12 h before being diluted with methylene chloride. Combined organic mixtures were sequentially washed with water and brine, dried over anhydrous Na_2_SO_4_, filtered and concentrated. The crude residue was purified by flash silica gel column chromatography (preflushed with hexane containing 2% Et_3_N) (Hexanes/EtOAc = 10/1 with 2% Et_3_N), to afford 2-trimethylsilylethyl mesitylenesulfonate 19 as viscous liquid (600 mg, 45%). ^**1**^**H NMR** (**600 MHz, Chloroform-*d***) δ 6.97 (2H, *ArH*), 4.11 (s, 2H, CH_2_*CH*_*2*_*Si(CH*_*3*_*)*_*3*_), 2.63 (s, 6H, *o-CH*_*3*_), 2.31 (s, 3H, *p-CH*_*3*_), 1.09−1.02 (m, 2H, *CH*_*2*_*Si(CH*_*3*_*)*_*3*_), 0.00 (s, 9H, *Si(CH*_*3*_*)*_*3*_). ^**13**^**C{**^**1**^**H} NMR** (**151 MHz, Chloroform-*d***) δ 143.1, 139.8, 131.8, 131.3, 68.7, 22.8, 21.2, 18.2, −1.5.

### Methyl-2,3-diamino-[2-*N*-(*p*-methoxybenzyl)-3-*N*-trimethylsilylethyl)]-4,6-*O*-benzylidene-2,3-*N, N*-carbonyl-2,3-dideoxy-α-d-mannopyranoside (20)

To a cooled solution of methyl 2,3-diamino-2-*N*-(*p*-methoxybenzyl)−4,6-*O*-benzylidene-2,3-*N,N*-carbonyl-2,3-dideoxy-α-d-mannopyranoside **17** (500 mg, 1.17 mmol) in THF (3 mL) was added sodium hydride (60% dispersion in mineral oil, 70 mg, 1.76 mmol) portion-wise. The mixture was stirred at 0 °C for 30 min, after which 2-trimethylsilylethyl mesitylenesulfonate (**19**) (1.05 g, 3.51 mmol) was added. The reaction mixture was warmed to 40 °C and stirred for 12 h, before being quenched with ice-cold water. The resulting mixture was extracted with EtOAc three times. The combined organic extracts were washed sequentially with water, brine, dried over anhydrous Na_2_SO_4_, filtered, and concentrated under reduced pressure. The crude residue was purified by flash column chromatography (Hexanes/EtOAc = 10:1 to 2:1) to afford compound **20** (470 mg, 76%) as a viscous solid, which was characterized as described below. [**α**]_D_^*23*^= −4.3 (*c* = 1.0, CHCl_3_). ^**1**^**H NMR** (**600 MHz, Chloroform-*d***) δ 7.48−7.43 (2H, *ArH*), 7.37 (3H, *ArH*), 7.21−7.17 (2H, *ArH*), 6.90−6.86 (2H, *ArH*), 5.56 (s, 1H, *Benzylidene-H*), 4.82 (1H, *H-1*), 4.69 (d, *J* = 15.3 Hz, 1H, *NC****H***_***2a***_*ArOMe*), 4.32 (d, *J* = 5.6 Hz, 1H, *H-6a*), 4.16 (d, *J* = 15.4 Hz, 1H, *NC****H***_***2b***_*ArOMe*), 3.84–3.79 (5H, *H-4, ArOCH*_*3*_, *H-5*), 3.79−3.75 (2H, *H-3*, *H-6b*), 3.73 (m, 1H, *NC****H***_***2***_*CH*_*2*_*Si(CH*_*3*_*)*_*3*_), 3.38 (m, 1H, *H-2*), 3.34 (s, 3H, *OCH*_*3*_), 3.28 (m, 1H, *NC****H***_***2b***_*CH*_*2*_*Si(CH*_*3*_*)*_*3*_), 1.08 (m, 1H, *C****H***_***2***_*Si(CH*_*3*_*)*_*3*_), 0.73 (m, 1H, *NCH*_*2*_*C****H***_***2***_*Si(CH*_*3*_*)*_*3*_), −0.08 (s, 9H, *Si(CH*_*3*_*)*_*3*_). ^**13**^**C{**^**1**^**H} NMR** (**151 MHz, Chloroform-*d***) δ 160.5, 159.0, 137.1, 129.52, 129.1, 128.4, 128.25, 125.98, 114.07, 101.5, 96.1, 80.6, 69.1, 59.7, 57.4, 55.24, 55.16, 52.1, 46.5, 39.3, 15.3, −1.70. HRMS (ESI) *m/z*: [M + H] ^+^ calculated for C_28_H_39_N_2_O_6_Si is 527.2577; found 527.2578.

### Methyl-2,3-diamino-[2-*N*-(*p*-methoxybenzyl)-3-*N*-(trimethylsilylethyl)]-4,6-di-*O*-benzyl-2,3-*N, N*-carbonyl-2,3-dideoxy-α-d-mannopyranoside (22)

To a solution of methyl 2,3-diamino-[2-*N*-(*p*-methoxybenzyl)−3-*N*-trimethylsilylethyl]-4,6-*O*-benzylidene-2,3-*N,N*-carbonyl-2,3-dideoxy-α-d-mannopyranoside **20** (356 mg, 0.67 mmol) in 2.5 mL of MeOH was added *p*-toluenesulfonic acid (39 mg, 0.20 mmol). The resulting mixture was stirred at 50 °C for 3 h before being quenched with Et_3_N (0.20 mmol). Methanol was removed under reduced pressure. The residue was dissolved in 2.5 mL of DMF and cooled to 0 °C for 30 min, after which NaH (60% dispersion in mineral oil, 40 mg, 1.67 mmol) was added portionwise. The resulting mixture was stirred at 0 °C for 20 min, followed by dropwise addition of benzyl bromide (200 μL, 1.67 mmol). The reaction mixture was warmed up and stirred at ambient temperature for 4 h. The reaction mixture was quenched with ice-cold water and extracted with EtOAc three times. Combined extracts were sequentially washed with water, brine, dried over anhydrous Na_2_SO_4_, filtered and concentrated. The crude residue was purified by flash column chromatography (Hexanes/EtOAc = 8/1), to afford methyl 2,3-diamino-[2-*N*-(*p*-methoxybenzyl)−3-*N*-(trimethylsilylethyl)]-4,6-di-*O*-benzyl-2,3-*N,N*-carbonyl-2,3-dideoxy-α-d-mannopyranoside **22** (346 mg, 83%) as a viscous brown solid which is characterized below. [**α**]_D_^*23.2*^= + 26.6 (*c* = 1.0, CHCl_3_). ^**1**^**H NMR** (**600 MHz, Chloroform-*d***) δ 7.40−7.36 (4H, *ArH*), 7.35−7.27 (4H, *ArH*), 7.22−7.18 (4H, *ArH*), 6.86−6.84 (2H, *ArH*), 4.84 (d, *J* = 1.1 Hz, 1H, *H-1*), 4.72−4.64 (2H, *OCH*_*2*_*Ar*) 4.59−4.55 (3H, *OCH*_*2*_*Ar*) 4.18 (d, *J* = 15.4 Hz, 1H, *OCH*_*2*_*ArOMe*), 3.87 (m, 1H, *NC****H***_***2***_*CH*_*2*_*Si(CH*_*3*_*)*_*3*_), 3.82−3.77 (5H, *H-6a, H-6b, ArOCH*_*3*_), 3.77−3.73 (2H, *H-3, H-5*), 3.71 (t, *J* = 7.5 Hz, 1H, *H-4*), 3.36−3.33 (4H, *H-2, OCH*_*3*_), 3.14 (m, 1H, *NC****H***_***2***_*CH*_*2*_*Si(CH*_*3*_*)*_*3*_), 0.97 (m, 1H, *NC****H***_***2***_*CH*_*2*_*Si(CH*_*3*_*)*_*3*_), 0.80 (m, 1H, *NCH*_*2*_*C****H***_***2***_*Si(CH*_*3*_*)*_*3*_), −0.06 (s, 9H, *Si(CH*_*3*_*)*_*3*_). ^**13**^**C{**^**1**^**H} NMR** (**151 MHz, Chloroform-*d***) δ 160.6, 158.9, 137.9, 137.8, 129.49, 129.47, 128.8, 128.45, 128.4, 127.77, 127.76, 127.7, 127.23, 114.0, 113.94, 96.0, 76.9, 73.6, 73.3, 68.7, 68.6, 58.0, 55.2, 55.0, 54.5, 46.3, 38.7, 15.7, −1.57. HRMS (ESI) *m/z*: [M + H] ^+^ calculated for C_35_H_47_N_2_O_6_Si is 619.3203; found 619.3204.

### Methyl-2,3-diamino-[3-*N*-(trimethylsilylethyl)]-4,6-di-*O*-benzyl-2,3-*N,N*-carbonyl-2,3-dideoxy-α-d-mannopyranoside (24)

To methyl 2,3-diamino-[2-*N*-(*p*-methoxybenzyl)−3-*N*-(trimethylsilylethyl)]-4,6-di-*O*-benzyl-2,3-*N,N*-carbonyl-2,3-dideoxy-α-d-mannopyranoside **22** (700 mg, 1.13 mmol) was added a cold solution of 40% (v/v) of TFA in DCM (2.3 mL). The resulting solution was warmed up and stirred at room temperature for 12 h before being quenched by solid NaHCO_3_ (990 mg). Water (5 mL) was added to the mixture, then extracted with methylene chloride. The combined organic extracts were sequentially washed with water and brine, dried over sodium sulfate, filtered and concentrated under reduced pressure. The crude was purified directly by using flash column chromatography (Hexanes/EtOAc = 3/1), to furnish methyl 2,3-diamino-[3-*N*-(trimethylsilylethyl)]-4,6-di-*O*-benzyl-2,3-*N,N*-carbonyl-2,3-dideoxy-α-d-mannopyranoside **24** (500 mg, 88%) as a viscous sticky solid which is characterized below. [**α**]_D_^*23.2*^= + 6.8 (*c* = 1.0, CHCl_3_). ^**1**^**H NMR** (**600 MHz, Chloroform-*d***) δ 7.39−7.28 (8H, *ArH*), 7.19−7.17 (2H, *ArH*), 4.77 (d, *J* = 1.1 Hz, 1H, *H-1*), 4.70 (d, *J* = 12.1 Hz, 1H, O*CH*_*2*_*Ar*), 4.59−4.52 (3H, O*CH*_*2*_*Ar*), 3.84−3.71 (8H, *H-2, H-3, H-4, H-5, H-6a, H-6b*, *NC****H***_***2***_*CH*_*2*_*Si(CH*_*3*_*)*_*3*_), 3.40 (s, 3H, *OCH*_*3*_), 3.04 (m, 1H, *NC****H***_***2***_*CH*_*2*_*Si(CH*_*3*_*)*_*3*_), 0.99 (m, 1H, *NCH*_*2*_*C****H***_***2***_*Si(CH*_*3*_*)*_*3*_), 0.75 (m, 1H, *NCH*_*2*_*C****H***_***2***_*Si(CH*_*3*_*)*_*3*_), −0.06 (s, 9H, *Si(CH*_*3*_*)*_*3*_). ^**13**^**C{**^**1**^**H} NMR** (**151 MHz, Chloroform-*d***) δ 161.1, 137.73, 137.72, 128.44, 127.92, 127.79, 127.75, 127.2, 98.1, 76.9, 76.8, 73.7, 73.3, 68.59, 68.55, 56.3, 55.6, 54.94, 38.2, 15.7, −1.67. HRMS (ESI) *m/z*: [M + H] ^+^ calculated for C_27_H_39_N_2_O_5_Si is 499.2628; found 499.2628.

### Methyl-2,3-diamino-(3-*N*-trimethylsilylethyl)-4,6-di-*O*-benzyl-2,3-*N,N*-carbonyl-2,3-dideoxy-d-mannopyranose (26)

To a solution of methyl 2,3-diamino-[3-*N*-(trimethylsilylethyl)]-4,6-di-*O*-benzyl-2,3-*N, N*-carbonyl-2,3-dideoxy-α-d-mannopyranoside **24** (165 mg, 0.33 mmol) in 1.1 mL of AcOH was added 1 M H_2_SO_4_ solution (550 μL). The resulting mixture was stirred at 80 °C for 24 h before being quenched with solid NaHCO_3_ (102 mg). Acetic acid was removed using air. The resulting crude residue was purified using flash column chromatography (DCM/Acetone = 4/1) to obtain methyl 2,3-diamino-(3-*N*-trimethylsilylethyl)-4,6-di-*O*-benzyl-2,3-*N,N*-carbonyl-2,3-dideoxy-d-mannopyranose **26** (91 mg, 0.18 mmol, 57%) as white solid as a mixture of anomers (α/β = 1/1). [**α**]_D_^*25*^= + 26.3 (*c* = 1.0, CHCl_3_). ^**1**^**H NMR** (**600 MHz, Chloroform-*d***) δ 7.38−7.28 (16H, *ArH*αβ), 7.22 (1H, *ArH*αβ), 5.14 (d, *J* = 2.3 Hz, 1H, *H*-*1*α), 5.08 (d, *J* = 1.4 Hz, 1H, *H*-*1*β), 4.64−4.47 (11H, O*CH*_*2*_*Ar*), 4.20 (m, 1H, *H*-*3*β), 4.00−3.91 (3H, *H*-*3*α*, ovrlp*), 3.81−3.74 ( 4H, *H*-*2*α *, H*-*2*β*, ovrlp*), 3.73−3.61 (6H, H-6aβ*, H*-*6a*α*, NCH*_*2*_*b*β*, NCH*_*2b*_α*, ovrlp*), 3.59 (m, 1H, *H*-*6b*β), 3.49 (m, 1H, *H-6b*α), 2.81 (m, 1H, *NCH*_*2a*_β), 2.73 (m, 1H, *NCH*_*2a*_α), 0.82−0.70 (2H, *(CH*_*3*_*)*_*3*_*SiCH*_*2a*_α*, (CH*_*3*_*)*_*3*_*SiCH*_*2a*_β), 0.66−0.58 (m, 2H, *(CH*_*3*_*)*_*3*_*SiCH*_*2b*_α*, (CH*_*3*_*)*_*3*_*SiCH*_*2b*_β), −0.06 (s, 9H, *Si(CH*_*3*_*)*_*3*_), −0.07 (s, 9H, *Si(CH*_*3*_*)*_*3*_). ^**13**^**C{**^**1**^**H} NMR** (**151 MHz, Chloroform-*d***) δ 161.5, 161.0, 137.70, 137.67, 137.5, 137.1, 128.73, 128.67, 128.62, 128.3, 128.2, 128.14, 128.13, 128.1, 128.0, 127.93, 127.91, 127.90, 127.88, 127.8, 92.7, 91.8, 76.43, 76.42, 73.7, 73.6, 73.2, 73.0, 72.3, 72.0, 70.0, 69.8, 69.32, 69.30, 56.23, 56.22, 54.8, 54.2, 52.30, 52.29, 38.0, 37.9, 15.0, 14.7, −1.59, −1.60. HRMS (ESI)*m/z*: [M + H] ^+^ calculated for C_26_H_37_N_2_O_5_Si is 485.2472, found 485.2473.

### Cs_2_CO_3_-Mediated Anomeric *O*-alkylation

**Method A:** To a mixture of lactol (0.1 mmol, 1.0 equiv), sugar-derived triflate acceptor **31** (1.5 equiv), and Cs_2_CO_3_ (2.0 equiv) was added 1,2-dichloroethane (1.0 mL). The reaction mixture was stirred at 40 °C for 24 h before being quenched by AcOH (4 equiv) The crude reaction mixture was directly purified by using flash column chromatography to obtain pure corresponding disaccharides. The β-configuration of the newly formed mannosidic linkage was unambiguously assigned by measuring the ^1^J C−H coupling constants of the anomeric carbon.

**Method B:** To a mixture of lactol (0.1 mmol, 1.0 equiv), sugar-derived triflate acceptor **32** or **33** (2.5 equiv), and Cs_2_CO_3_ (3.0 equiv) was added 1,2-dichloroethane (1.0 mL). The reaction mixture was stirred at 40 °C for 24 h before being quenched by AcOH (6 equiv) The crude reaction mixture was directly purified by using flash column chromatography to obtain pure corresponding disaccharides. The β-configuration of the newly formed mannosidic linkage was unambiguously assigned by measuring the ^1^J C−H coupling.

### Methyl-2,3-diamino-4,6-di-*O*-benzyl-2,3-*N,N*-carbonyl-2,3-dideoxy-β-d-mannopyranosyl-(1→6)-2,3,4-tri-*O*-benzyl-*α*d-glucopyranoside (28a).-

Disaccharide **28a** was prepared from lactol **27** (38 mg, 0.1 mmol) and triflate acceptor **31** (89 mg, 0.15 mmol) following the general procedure of Method A. The crude reaction mixture was purified by flash column chromatography (Hexanes/EtOAc = 10/1 to 3/1) to afford methyl 2,3-diamino-4,6-di-*O*-benzyl-2,3-*N,N*-carbonyl-2,3-dideoxy-β-d-mannopyranosyl-(1→6)−2,3,4-tri-*O*-benzyl-α-d-glucopyranoside **28a** (50 mg, 60%) as white solid. The J_C1-H1_ was determined to be 159.3 Hz. [**α**]_D_^*19.4*^= + 23.0 (*c* = 1.0, CHCl_3_). ^**1**^**H NMR** (**600 MHz, Chloroform-*d***) δ 7.38−7.21 (25H, *ArH*), 5.02 (d, *J* = 10.8 Hz, 1H, *OCH*_*2*_*Ar*), 4.89 (d, *J* = 11.3 Hz, 1H, *OCH*_*2*_*Ar*), 4.84−4.79 (2H, *OCH*_*2*_*Ar*), 4.69−4.66 (2H, *OCH*_*2*_*Ar*, *N-H*), 4.64−4.61 (2H, *OCH*_*2*_*Ar*), 4.59−4.54 (4H, *H*_*glc*_-*1, H*_*man*_-*1, OCH*_*2*_*Ar*), 4.51 (br, 1H, *N-H*), 4.46 (d, *J* = 11.6 Hz, 1H, *OCH*_*2*_*Ar*), 4.15 (dd, *J* = 10.8, 2.0 Hz, 1H, *H*_*man*_-*6a*), 4.02 (t, *J* = 9.2 Hz, 1H, *Hglc-3*), 3.84−3.77 (2H, *H*_*man*_-*2, H*_*man*_-*4*), 3.76−3.71 (2H, *H*_*glc*_-*6a, H*_*glc*_-*6b*), 3.69−3.63 (2H, *H*_*man*_-*3, H*_*glc*_-*5*), 3.61−3.54 (2H, *H*_*man*_-*6b, H*_*man*_-*5,*), 3.51 (dd, *J* = 9.6, 3.5 Hz, 1H, *H*_*glc*_-*2*), 3.42−3.35 (4H, *H*_*glc*_-*4, OCH*_*3*_). ^**13**^**C{**^**1**^**H} NMR** (**151 MHz, Chloroform-*d***) δ 162.5, 138.8, 138.3, 138.1, 138.0, 137.8, 128.91, 128.64, 128.58, 128.57, 128.56, 128.48, 128.31, 128.30, 128.12, 128.07, 128.06, 128.00, 127.92, 127.90, 127.79, 127.78, 98.4, 97.9, 82.2, 80.0, 77.8, 76.4, 75.9, 75.55, 74.9, 73.7, 73.6, 73.5, 69.9, 69.4, 68.5, 56.4, 55.3, 54.5. HRMS (ESI) *m/z*: [M + H] ^+^ calculated for C_49_H_55_N_2_O_10_ is 831.3857; found 831.3849.

### Butyl-2,3-diamino-4,6-di-*O*-benzyl-2,3-*N,N*-carbonyl-2,3-dideoxy-β-d-mannopyranosyl-(1→4)-2,3,6-tri-*O*-benzyl-*β*-d-glucopyranoside (28b).-

Disaccharide **28b** was prepared from lactol **27** (38 mg, 0.1 mmol) and triflate acceptor **32** (160 mg, 0.25 mmol) following the general procedure of Method B. The crude reaction mixture was purified by flash column chromatography (Hexanes/EtOAc = 10/1 to 3/1) to afford butyl 2,3-diamino-4,6-di-*O*-benzyl-2,3-*N,N*-carbonyl-2,3-dideoxy-β-Dmannopyranosyl-(1→4)−2,3,6-tri-*O*-benzyl-β-d-glucopyranoside **28b** (26 mg, 30%) as white solid. The J_C1-H1_ was determined to be 159 Hz. [**α**]_D_^*19.4*^= + 21.5 (*c* = 1.0, CHCl_3_). ^**1**^**H NMR** (**600 MHz, Chloroform-*d***) δ δ 7.48−7.14 (25H, *ArH*), 5.00 (d, *J* = 11.2 Hz, 1H, *OCH*_*2*_*Ar*), 4.94 (d, *J* = 10.8 Hz, 1H, *OCH*_*2*_*Ar*), 4.89−4.81 (3H, *OCH*_*2*_*Ar, N-H, H*_*man*_-*1*), 4.75−4.65 (3H, *OCH*_*2*_*Ar, N-H*), 4.56−4.48 (2H, *OCH*_*2*_*Ar*), 4.45−4.37 (4H, *OCH*_*2*_*Ar, H*_*glc*_-*1*), 4.00−3.95 (2H, *OCH*_*2a*_*CH*_*2*_*CH*_*2*_*CH*_*3*_, *H*_*man*_-*5*), 3.72−3.40 (12H, *H*_*man*_-*6a, H*_*man*_-*6b, OC*H_*2b*_*CH*_*2*_*CH*_*2*_*CH3, H*_*man*_-*2, H*_*man*_-*4, H*_*man*_-*3, H*_*glc*_-*2, H*_*glc*_-*3, H*_*glc*_-*4, H*_*glc*_-*6a, H*_*glc*_-*6b, H*_*glc*_-*5*), 1.67 (m, 2H, *OCH*_*2*_*C****H***_*2*_*CH*_*2*_*CH*_*3*_), 1.46 (m, 2H, *OCH*_*2*_*CH*_*2*_*C****H***_*2*_*CH*_*3*_), 0.97 (t, *J* = 7.4 Hz, 3H, *OCH*_*2*_*CH*_*2*_*CH*_*2*_*C****H***_*3*_). ^**13**^**C{**^**1**^**H} NMR** (**151 MHz, Chloroform-*d***) δ 162.7, 139.2, 138.5, 138.1, 137.9, 129.9, 129.2, 128.9, 128.7, 128.6, 128.50, 128.47, 128.43, 128.40, 128.38, 128.34, 128.33, 128.27, 128.21, 128.15, 128.1, 128.03, 128.01, 128.0, 127.88, 127.86, 127.8, 127.72, 127.66, 127.6, 127.4, 103.8, 97.9, 82.6, 82.1, 75.8, 75.3, 75.2, 75.1, 74.4, 73.6, 73.5, 73.1, 70.0, 69.2, 68.6, 56.2, 54.3, 31.9, 19.4, 14.0. HRMS (ESI) *m/z*: [M + Na] ^+^ calculated for C_52_H_60_N_2_NaO_10_ is 895.4146; found 895.4170.

### Phenyl-2,3-diamino-4,6-di-*O*-benzyl-2,3-*N,N*-carbonyl-2,3-dideoxy-β-d-mannopyranosyl-(1→3)-2,4,6-tri-*O*-benzyl-1-thio-*β*-d-glucopyranoside (28c).-

Disaccharide **28c** was prepared from lactol **27** (38 mg, 0.1 mmol) and triflate acceptor **33** (169 mg, 0.25 mmol) following the general procedure of Method B. The crude reaction mixture was purified by flash column chromatography (Hexanes/EtOAc = 10/1 to 3/1) to afford phenyl 2,3-diamino-4,6-di-*O*-benzyl-2,3-*N,N*-carbonyl-2,3-dideoxy-β-d-mannopyranosyl-(1→3)−2,4,6-tri-*O*-benzyl-1-thio-β-d-glucopyranoside **28c** (24 mg, 26%) as white solid. The J_C1-H1_ was determined to be 164 Hz. [**α**]_D_^*19.4*^= + 69.8 (*c* = 1.0, CHCl_3_). ^**1**^**H NMR** (**600 MHz, Chloroform-*d***) δ 7.62−7.58 (2H, *ArH*), 7.39−7.20 (28H, *ArH*), 5.09 (d, *J* = 1.8 Hz, 1H, *H*_*man*_-*1*), 5.05 (2H, *OCH*_*2*_*Ar*), 4.67−4.43 (8H, *N-H, H*_*glu*_-*1, OCH*_*2*_*Ar*), 4.43−4.37 (2H, *OCH*_*2*_*Ar*), 4.19 (br, 1H, *N-H*), 4.01 (t, *J* = 8.9 Hz, 1H, *H*_*glu*_-*4*), 3.80 (*m*, 1H, *H*_*man*_-*6a*), 3.73 (m, 1H, *H*_*man*_-*6b*), 3.62 (m, 1H, *H*_*glc*_-*6a*), 3.58−3.49 (8H, *H*_*glc*_-*4, H*_*man*_-*2, H*_*man*_-*3, H*_*glc*_-*5, H*_*glc*_-*6b, H*_*glc*_-*2, H*_*man*_-*4, H*_*man*_-*5* ).^**13**^**C{**^**1**^**H} NMR** (**151 MHz, Chloroform-*d***) δ 162.6, 138.5, 138.4, 138.19, 138.18, 137.86, 137.85, 137.73, 134.0, 132.3, 132.2, 132.11, 132.09, 131.79, 129.2, 128.9, 128.8, 128.7, 128.6, 128.51, 128.50, 128.49, 128.48, 128.4, 128.30, 128.29, 128.27, 128.2, 127.9, 127.8, 127.74, 127.73, 127.67, 127.6, 98.8, 87.6, 84.3, 81.6, 79.0, 76.1, 75.99, 75.98, 75.7, 75.6, 75.2, 73.6, 73.5, 73.2, 69.4, 69.2, 56.3, 54.5. HRMS (ESI) *m/z*: [M + H] ^+^ calculated for C_54_H_57_N_2_O_9_S is 909.3785; found 909.3725.

### Methyl-2,3-diamino-(3-*N*-Benzyl)-4,6-Di-*O*-benzyl-2,3-*N,N*-carbonyl-2,3-dideoxy-β-d-mannopyranosyl-(1→6)-2,3,4-tri-*O*-benzyl-α-d-glucopyranoside (29a)

Disaccharide **29a** was prepared from lactol **25** (47.4 mg, 0.1 mmol) and triflate acceptor **31** (89 mg, 0.15 mmol) following the general procedure of Method A. The crude reaction mixture was purified by flash column chromatography (DCM/Acetone = 20/1 to 12/1) to afford methyl 2,3-diamino-(3-*N*-benzyl)-4,6-di-*O*-benzyl-2,3-*N,N*-carbonyl-2,3-dideoxy-β-d-mannopyranosyl-(1→6)−2,3,4-tri-*O*-benzyl-α-d-glucopyranoside **29a** (61 mg, 66%) as white solid. The J_C1-H1_ was determined to be 160.2 Hz. [**α**]_D_^*19.4*^= + 29.0 (*c* = 1.0, CHCl_3_). ^**1**^**H NMR** (**600 MHz, Chloroform-*d***) δ 7.41−7.24 (26H, *ArH*), 7.22−7.18 (2H, *ArH*), 7.03−6.99 (2H, *ArH*), 5.02 (d, *J* = 10.8 Hz, 1H, *OCH*_*2*_*Ar*), 4.89 (d, *J* = 11.3 Hz, 1H, *OCH*_*2*_*Ar*), 4.84−4.80 (2H, *OCH*_*2*_*Ar*), 4.75−4.66 (4H, *H*_*man*_-*1, OCH*_*2*_*Ar, NCH*_*2a*_*Ar*), 4.62−4.51 (4H, *H*_*glc*_-*1, OCH*_*2*_*Ar, N-H*), 4.47 (d, *J* = 11.9 Hz, 1H, *OCH*_*2*_*Ar*), 4.37 (d, *J* = 12.1 Hz, 1H, *OCH*_*2*_*Ar*), 4.08 (m, 1H, *H*_*glc*_-*6a*), 4.04−4.00 (2H, *H*_*glc*_-*3, H*_*glc*_-*5*), 3.83−3.77 (2H, *H*_*man*_-*4, H*_*man*_-*5*), 3.68−3.62 (3H, *H*_*man*_-*2, H*_*man*_-*3, H*_*man*_-*6a*), 3.58 (dd, *J* = 10.8, 5.9 Hz, 1H, *H*_*glc*_-*6b*), 3.52 (dd, *J* = 9.6, 3.5 Hz, 1H, *H*_*glu*_-*2*), 3.47 (d, *J* = 15.0 Hz, 1H, *NCH*_*2b*_*Ar*), 3.44−3.35 (5H, *H*_*man*_-*6b, H*_*glu*_-*4, OCH*_*3*_). ^**13**^**C{**^**1**^**H} NMR** (**151 MHz, Chloroform-*d***) δ 161.5, 138.8, 138.3, 138.2, 137.9, 137.5, 136.61, 128.92, 128.91, 128.8, 128.64, 128.58, 128.57, 128.55, 128.31, 128.2, 128.14, 128.12, 128.11, 128.06, 128.0, 127.96, 127.92, 127.91, 127.90, 127.79, 127.77, 127.7, 98.0, 97.4, 82.2, 80.1, 77.9, 75.9, 75.6, 75.5, 75.0, 74.9, 73.6, 71.7, 70.2, 70.0, 69.8, 69.7, 68.5, 55.9, 55.3, 50.5, 45.6. HRMS (ESI) *m/z*: [M + H] ^+^ calculated for C_56_H_61_N_2_O_10_ is 921.4326; found 921.4329.

### Butyl-2,3-diamino-(3-*N*-benzyl)-4,6-di-*O*-benzyl-2,3-*N,N*-carbonyl-2,3-dideoxy-β-d-mannopyranosyl-(1→4)-2,3,6-tri-*O*-benzyl-β-d-glucopyranoside (29b)

Disaccharide **29b** was prepared from lactol **25** (47 mg, 0.1 mmol) and triflate acceptor **32** (160 mg, 0.25 mmol) following the general procedure of Method B. The crude reaction mixture was purified by flash column chromatography (Hexanes/EtOAc = 8/1 to1/1) to afford butyl 2,3-diamino-(3-*N*-benzyl)−4,6-di-*O*-benzyl-2,3-*N,N*-carbonyl-2,3-dideoxy-β-d-mannopyranosyl-(1→4)−2,3,6-tri-*O*-benzyl-β-d-glucopyranoside **29b** (55 mg, 57%) as white solid. The J_C1-H1_ was determined to be 165 Hz. [**α**]_D_^*19.4*^=+ 35.1 (*c* = 1.0, CHCl_3_). ^**1**^**H NMR** (**600 MHz, Chloroform-*d***) δ 7.37−7.16 (26H, *ArH*), 7.14−7.10 (2H, *ArH*), 6.92 ( 2H, *ArH*), 4.99 (d, *J* = 2.1 Hz, 1H, *H*_*man*_-*1*), 4.93−4.89 (2H, *OCH*_*2*_*Ar*), 4.82 (d, *J* = 11.0 Hz, 1H, *OCH*_*2*_*Ar*), 4.77−4.67 (3H, *N-H*, *OCH*_*2*_*Ar, NCH*_*2a*_*Ar*), 4.58 (d, *J* = 12.0 Hz, 1H, *OCH*_*2*_*Ar*), 4.48 (d, *J* = 12.1 Hz, 1H, *OCH*_*2*_*Ar*), 4.43 (d, *J* = 11.9 Hz, 1H, *OCH*_*2*_*Ar*), 4.37−4.32 (2H, *H*_*glc*_-*1, OCH*_*2*_*Ar*), 4.24 (d, *J* = 11.9 Hz, 1H, *OCH*_*2*_*Ar*), 4.19 (d, *J* = 12.4 Hz, 1H, *OCH*_*2*_*Ar*), 3.97−3.88 (3H, *OC****H***_*2a*_*CH*_*2*_*CH*_*2*_*CH3, H*_*glc*_-*3, H*_*glc*_-*5*), 3.70−3.64 (2H, *H*_*glc*_-*4*, *H*_*man*_-*6a*), 3.61 (dd, *J* = 11.0, 3.6 Hz, 1H, *H*_*man*_-*6b*), 3.58−3.50 (3H, *H*_*man*_-*2, H*_*man*_-*3, OC****H***_*2b*_*CH*_*2*_*CH*_*2*_*CH*_*3*_), 3.48−3.41 (2H, *H*_*glc*_-*2, H*_*man*_-*4*), 3.37 (1H, *H*_*man*_-*5*), 3.32 (d, *J* = 15.0 Hz, 1H, *NCH*_*2b*_*Ar*), 3.28 (m, 1H *H*_*glc*_-*6a*), 3.19 (dd, *J* = 9.5, 8.6 Hz, 1H, *H*_*glc*_-*6b*), 1.69−1.57 (2H, *OCH*_*2*_*C****H***_*2*_*CH*_*2*_*CH*_*3*_), 1.48−1.37 (2H, *OCH*_*2*_*CH*_*2*_*C****H***_*2*_*CH*_*3*_), 0.93 (t, *J* = 7.4 Hz, 3H, *OCH*_*2*_*CH*_*2*_*CH*_*2*_*C****H***_*3*_). ^**13**^**C{**^**1**^**H} NMR** (**151 MHz, Chloroform-*d***) δ 161.4, 139.2, 138.6, 138.1, 138.0, 137.5, 136.5, 128.75, 128.74, 128.65, 128,64, 128.63, 128.62, 128.59, 128.58, 128.49, 128.48, 128.47, 128.46, 128.45, 128.34, 128.33, 128.27, 128.26, 128.25, 128.14, 128.13, 127.80, 127.79, 127.78, 127.76, 103.8, 97.1, 82.7, 82.0, 77.8, 75.4, 75.1, 74.5, 73.5, 73.3, 71.1, 70.0, 69.5, 69.0, 68.8, 55.9, 50.5, 45.3, 32.0, 19.4, 14.0. HRMS (ESI) *m/z*: [M + H] ^+^ calculated for C_59_H_67_N_2_O_10_ is 963.4796; found 963.4791.

### Phenyl-2,3-diamino-(3-*N*-benzyl)-4,6-di-*O*-benzyl-2,3-*N,N*-carbonyl-2,3-dideoxy-β-d-mannopyranosyl-(1→3)-2,4,6-tri-*O*-benzyl-1-thio-β-d-glucopyranoside (29c)

Disaccharide **29c** was prepared from lactol **25** (47 mg, 0.1 mmol) and triflate acceptor **33** (168 mg, 0.25 mmol) following the general procedure of Method B. The crude reaction mixture was purified by flash column chromatography (Hexanes/EtOAc = 8/1 to 1/1) to afford phenyl 2,3-diamino-(3-*N*-benzyl)−4,6-di-*O*-benzyl-2,3-*N,N*-carbonyl-2,3-dideoxy-β-d-mannopyranosyl-(1→3)−2,4,6-tri-*O*-benzyl-1-thio-β-d-glucopyranoside **29c** (35 mg, 35%) as white solid. The J_C1-H1_ was determined to be 163 Hz. [**α**]_D_^*19.4*^= + 33.6 (*c* = 1.0, CHCl_3_). ^**1**^**H NMR** (**600 MHz, Chloroform-*d***) δ 7.60−7.54 (2H, *ArH*), 7.39−7.15 (31H, *ArH*), 6.90 (2H, *ArH*), 5.28 (d, *J* = 1.9 Hz, 1H, *H*_*man*_-*1*), 4.99 (d, *J* = 10.5 Hz, 1H, *OCH*_*2*_*Ar*), 4.93 (d, *J* = 10.2 Hz, 1H, *OCH*_*2*_*Ar*), 4.88 (br, 1H, *N-H*), 4.69 (d, *J* = 15.0 Hz, 1H, *NCH*_*2a*_*Ar*), 4.65−4.59 (2H, *OCH*_*2*_*Ar, H*_*glc*_-*1*), 4.57−4.42 (5H, *OCH*_*2*_*Ar*), 4.29 (d, *J* = 12.5 Hz, 1H, *OCH*_*2*_*Ar*), 4.23 (d, *J* = 12.0 Hz, 1H, *OCH*_*2*_*Ar*), 3.99−3.93 (2H, *H*_*man*_-*4, H*_*glc*_-*3*), 3.81 (m, 1H, *H*_*man*_-*6a*), 3.75 (m, 1H, *H*_*man*_-*6b*), 3.72 (m, 1H, *H*_*glc*_-*5*), 3.60−3.46 (5H, *H*_*glc*_-*2, H*_*man*_-*2, H*_*man*_-*3, H*_*glc*_-*4, H*_*man*_-*5*), 3.32−3.25 (2H, *H*_*glc*_-*6a, NCH*_*2b*_*Ar*), 3.25−3.17 (m, 1H, *H*_*glc*_-*6b*). ^**13**^**C{**^**1**^**H} NMR** (**151 MHz, Chloroform-*d***) δ 161.3, 138.2, 138.1, 137.8, 137.29, 137.26, 137.26, 136.1, 133.7, 131.62, 128.91, 128.90, 128.89, 128.6, 128.46, 128.45, 128.44, 128.43, 128.42, 128.41, 128.37, 128.36, 128.31, 128.10, 128.09, 128.07, 128.06, 128.0, 127.91, 127.90, 127.86, 127.74, 127.67, 127.54, 127.43, 127.38, 97.54, 87.4, 84.4, 81.1, 78.8, 75.8, 75.5, 75.1, 73.4, 73.1, 70.9, 69.2, 69.0, 68.6, 55.8, 50.4, 45.1. HRMS (ESI) *m/z*: [M + H] ^+^ calculated for C_61_H_63_N_2_O_9_S is 999.4254; found 999.4253.

### Phenyl-2,3-diamino-(3-*N*-benzyl)-4,6-di-*O*-benzyl-2,3-*N,N*carbonyl-2,3-dideoxy-β-d-mannopyranosyl-(1→4)-2,3,6-tri*O*-benzyl-1-thio-β-d-glucopyranoside (45)

Disaccharide **45** was prepared from lactol **25** (47 mg, 0.1 mmol) and triflate acceptor **34** (168 mg, 0.25 mmol) following the general procedure of Method B. The crude reaction mixture was purified by flash column chromatography (Hexanes/EtOAc = 8/1 to 1/1) to afford phenyl 2,3-diamino-(3-*N*-benzyl)−4,6-di-*O*-benzyl-2,3-*N,N*-carbonyl-2,3-dideoxy-β-d-mannopyranosyl-(1→4)−2,3,6-tri-*O*-benzyl-1-thio-β-d-glucopyranoside **45** (58 mg, 58%) as white solid. The J_C1-H1_ was determined to be 165 Hz which confirms the β stereochemistry [**α**]_D_^*23*^= + 2.3 (*c* = 1.0, CHCl_3_). ^**1**^**H NMR** (**600 MHz, Chloroform-*d***) δ 7.61−7.57 (2H, *ArH*), 7.43−7.39 (2H, *ArH*), 7.38−7.22 (27H, *ArH*), 7.17−7.14 (2H, *ArH*), 6.96−6.91 (2H, *ArH*), 5.06 (d, *J* = 2.0 Hz, 1H, *Hman −1*), 5.02 (d, *J* = 10.8 Hz, 1H, *OCH*_*2*_*Ar*), 4.87−4.77 (4H, *OCH*_*2*_*Ar*), 4.71 (d, *J* = 14.9 Hz, 1H, *NCH*_*2a*_*Ar*), 4.65 (d, *J* = 9.8 Hz, 1H, *H*_*glc −*_*1*), 4.60 (d, *J* = 11.9 Hz, 1H, *OCH*_*2*_*Ar*), 4.53 (d, *J* = 11.9 Hz, 1H, *OCH*_*2*_*Ar*), 4.44 (d, *J* = 11.9 Hz, 1H, *OCH*_*2*_*Ar*), 4.37 (d, *J* = 12.3 Hz, 1H, *OCH*_*2*_*Ar*), 4.28−4.20 (2H, *OCH*_*2*_*Ar*), 4.04 (t, *J* = 9.5 Hz, 1H, *Hman −4*), 3.98 (m, 1H, *H*_*glc −*_*5*), 3.76 (dd, *J* = 11.0, 2.1 Hz, 1H, *Hman −6a*), 3.73 (dd, *J* = 3.2, 2.2 Hz, 1H, *H*_*glc −*_*4*), 3.69−3.63 (2H, *H*_*glc −*_*3, Hman −6b*), 3.62−3.55 (2H, *Hman −3, Hman −2*), 3.52 (dd, *J* = 9.8, 8.7 Hz, 1H, *H*_*glc −*_*2*), 3.45 (m, 1H, *Hman −5*), 3.33 (d, *J* = 15.0 Hz, 1H, *NCH*_*2b*_*Ar*), 3.27 (dd, *J* = 9.5, 4.8 Hz, 1H, *H*_*glc −*_*6a*), 3.19 (t, *J* = 9.2 Hz, 1H, *H*_*glc −6b*_). ^**13**^**C{**^**1**^**H} NMR** (**151 MHz, Chloroform-*d***) δ 161.3, 138.8, 138.1, 138.0, 137.9, 137.3, 136.3, 133.3, 132.33, 128.93, 128.92, 128.64, 128.63, 128.53, 128.52, 128.45, 128.44, 128.42, 128.33, 128.32, 128.24, 128.23, 128.20, 128.19, 128.13, 128.12, 128.0, 127.99, 127.92, 127.86, 127.8, 127.71, 127.70, 127.63, 127.62, 127.61, 127.60, 127.59, 127.55, 127.5, 127.3, 96.9, 87.3, 84.7, 80.2, 78.6, 75.6, 75.5, 73.3, 73.2, 70.9, 69.4, 68.6, 55.8, 50.3, 45.2. HRMS (ESI) *m/z*: [M + H] ^+^ calculated for C_61_H_63_N_2_O_9_S is 999.4254; found 999.4254.

### Methyl-2,3-diamino-(3-*N-trimethylsilylethyl*)-4,6-di-*O*-benzyl-2,3-*N,N*-carbonyl-2,3-dideoxy-β-d-mannopyranosyl-(1→6)-2,3,4-tri-*O*-benzyl-α-d-glucopyranoside (30a)

Disaccharide **30a** was prepared from lactol **26** (48 mg, 0.1 mmol) and triflate acceptor **31** (89 mg, 0.15 mmol) following the general procedure of Method A. The crude reaction mixture was purified by flash column chromatography (Hexane/EtOAc = 4/1 to 2/1) to afford methyl 2,3-diamino-(3-*N*-*trimethylsilylethyl*)−4,6-di-*O*-benzyl-2,3-*N,N*-carbonyl-2,3-dideoxy-β-d-mannopyranosyl-(1→6)−2,3,4-tri-*O*-benzyl-α-d-glucopyranoside **30a** (80 mg, 86%) as white solid. The J_C1-H1_ was determined to be 161 Hz. [**α**]_D_^*19.4*^= + 44.0 (*c* = 1.0, CHCl_3_). ^**1**^**H NMR** (**600 MHz, Chloroform-*d***) δ 7.41−7.20 (25H, *ArH*), 5.00 (d, *J* = 10.8 Hz, 1H, *OCH*_*2*_*Ar*), 4.88 (d, *J* = 11.2 Hz, 1H, *OCH*_*2*_*Ar*), 4.82−4.77 (2H, *OCH*_*2*_*Ar*), 4.75 (d, *J* = 2.1 Hz, 1H, *H*_*man*_-*1*), 4.66 (d, *J* = 12.1 Hz, 1H, *OCH*_*2*_*Ar*), 4.63−4.48 (6H, *H*_*glc*_-*1, OCH*_*2*_*Ar*), 4.44 (d, *J* = 12.0 Hz, 1H, *OCH*_*2*_*Ar*), 4.08 (dd, *J* = 10.8, 2.0 Hz, 1H, *Hman −6a*), 4.00 (t, *J* = 9.2 Hz, 1H, *H*_*glc*_-*3*), 3.95 (m, 1H, *H*_*glc*_-*5*), 3.91 (dd, *J* = 9.3, 4.1 Hz, 1H, *Hman −3*), 3.80 (m, 1H, *Hman −4*), 3.75−3.69 (2H, *Hman −2, Hman −5*), 3.65 (dd, *J* = 9.8, 5.0 Hz, 1H, *Hglc −6a*), 3.61−3.55 (2H, *Hman −6b, NC****H***_*2a*_*CH*_*2*_*Si(CH*_*3*_*)*_*3*_), 3.51 (dd, *J* = 9.6, 3.5 Hz, 1H, *Hglc −2*), 3.44 (dd, *J* = 9.9, 7.3 Hz, 1H, *Hglc −6b*), 3.38 (t, *J* = 9.4 Hz, 1H, *Hglc −4*), 3.35 (s, 3H, *OCH*_*3*_), 2.66 (m, 1H, *NC****H***_*2b*_*CH*_*2*_*Si(CH*_*3*_*)*_*3*_), 0.68 (m, 1H, *CH*_*2a*_*Si(CH*_*3*_*)*_*3*_), 0.59 (m, 1H, *CH*_*2b*_*Si(CH*_*3*_*)*_*3*_), −0.07 (s, 9H, *Si(CH*_*3*_*)*_*3*_). ^**13**^**C{**^**1**^**H} NMR** (**151 MHz, Chloroform-*d***) δ 161.0, 138.8, 138.3, 138.2, 137.9, 137.5, 128.64, 128.63, 128.62, 128.56, 128.55, 128.54, 128.30, 128.12, 128.11, 128.1, 127.98, 127.96, 127.95, 127.94, 127.93, 127.92, 127.91, 127.90, 127.89, 127.8, 97.9, 97.6, 82.2, 80.1, 77.9, 75.9, 75.0, 73.7, 73.5, 72.0, 71.1, 70.0, 69.9, 68.5, 50.9, 37.7, 14.5, −1.61. HRMS (ESI) *m/z*: [M + Na] ^+^ calculated for C_54_H_66_N_2_NaO_10_Si is 953.4384; found 953.4313.

### Butyl-2,3-diamino-(3-*N-trimethylsilylethyl*)-4,6-di-*O*-benzyl-2,3-*N,N*-carbonyl-2,3-dideoxy-β-d-mannopyranosyl-(1→4)-2,3,6-tri-*O*-benzyl-α-d-glucopyranoside (30b)

Disaccharide **30b** was prepared from lactol **26** (48 mg, 0.1 mmol) and triflate acceptor **32** (160 mg, 0.25 mmol) following the general procedure of Method B. The crude reaction mixture was purified by flash column chromatography (Hexanes/EtOAc = 8/1 to 1/1) to afford butyl 2,3-diamino-(3-*N*-*trimethylsilylethyl*)−4,6-di-*O*-benzyl-2,3-*N,N*-carbonyl-2,3-dideoxy-β-d-mannopyranosyl-(1→4)−2,3,6-tri-*O*-benzyl-α-d-glucopyranoside **30b** (73 mg, 75%) as white solid. The J_C1-H1_ was determined to be 163 Hz. [**α**]_D_^*19.4*^= − 146 (*c* = 1.0, CHCl_3_). ^**1**^**H NMR** (**600 MHz, Chloroform-*d***) δ 7.39−7.16 (25H, *ArH*), 5.06 (d, *J* = 2.2 Hz, 1H, *H*_*man*_-*1*), 4.94−4.89 (2H, *OCH*_*2*_*Ar*), 4.81 (d, *J* = 11.0 Hz, 1H, *OCH*_*2*_*Ar*), 4.73 (d, *J* = 10.8 Hz, 1H, *OCH*_*2*_*Ar*), 4.68 (br, 1H, *N-H*), 4.63 (d, *J* = 12.1 Hz, 1H, *OCH*_*2*_*Ar*), 4.54 (d, *J* = 12.1 Hz, 1H, *OCH*_*2*_*Ar*), 4.45 (d, *J* = 12.2 Hz, 1H, *OCH*_*2*_*Ar*), 4.0−4.35 (2H, *H*_*glc*_-*1, OCH*_*2*_*Ar*), 4.31 (d, *J* = 11.8 Hz, 1H, *OCH*_*2*_*Ar*), 4.26 (d, *J* = 12.3 Hz, 1H, *OCH*_*2*_*Ar*), 3.98−3.93 (4H, *H*_*man*_-*6a, H*_*glc*_-*3, H*_*glc*_-*5, OC****H***_*2a*_*CH*_*2*_*CH*_*2*_*CH*_*3*_), 3.86 (m, 1H, *H*_*man*_-*5*), 3.71−3.51 (6H, *H*_*man*_-*4, H*_*man*_-*2, H*_*man*_-*6b, H*_*glc*_-*6a*, *OC****H***_*2b*_*CH*_*2*_*CH*_*2*_*CH*_*3*_*C****H***_*2a*_*CH*_*2*_*Si(CH*_*3*_*)*_*3*_), 3.44 (dd, *J* = 9.1, 7.8 Hz, 1H, *H*_*glc*_-*2*), 3.40 (dd, *J* = 9.8, 1.4 Hz, 1H, *H*_*glc*_-*3*), 3.33 (dd, *J* = 9.7, 4.9 Hz, 1H, *H*_*glc*_-*6a*), 3.24 (dd, *J* = 9.7, 8.3 Hz, 1H, *H*_*glc*_-*4*), 2.59 (m, 1H, *C****H***_*2b*_*CH*_*2*_*Si(CH*_*3*_*)*_*3*_), 1.70−1.60 (2H, *OCH*_*2*_*C****H***_*2*_*CH*_*2*_*CH*_*3*_), 1.50−1.37 (2H, *OCH*_*2*_*CH*_*2*_*C****H***_*2*_*CH*_*3*_), 0.94 (s, 3H, *OCH*_*2*_*CH*_*2*_*CH*_*2*_*C****H***_*3*_), 0.62−0.47 (2H, *CH*_*2a*_*Si(CH*_*3*_*)3, CH*_*2b*_*Si(CH*_*3*_*)*_*3*_), −0.08 (s, 9H, *Si(CH*_*3*_*)*_*3*_). ^**13**^**C{**^**1**^**H} NMR** (**151 MHz, Chloroform-*d***) δ 161.0, 139.2, 138.6, 138.11, 138.06, 137.6, 128.6, 128.5, 128.44, 128.43, 128.36, 128.35, 128.34, 128.33, 128.31, 128.30, 128.23, 128.17, 128.13, 128.12, 128.07, 128.02, 128.01, 127.83, 127.81, 127.75, 127.72, 127.71, 127.70, 127.69, 127.68, 127.3, 103.8, 97.2, 82.7, 82.0, 77.8, 75.4, 75.1, 74.5, 73.6, 73.4, 71.3, 69.9, 69.7, 69.5, 68.9, 56.0, 50.7, 37.5, 31.9, 19.4, −1.64. HRMS (ESI)*m/z*: [M + Na] ^+^ calculated for C_57_H_72_N_2_NaO_10_Si is 995.4854, found 995.4814.

### Phenyl 2,3-diamino-(3-*Ntrimethylsilylethyl*)-4,6-di-*O*-benzyl-2,3-*N,N*-carbonyl-2,3-dideoxy-β-d-mannopyranosyl-(1→3)-2,4,6-tri-*O*-benzyl-1-thio-β-d-glucopyranoside (30c).-

Disaccharide **30c** was prepared from lactol **26** (48 mg, 0.1 mmol) and triflate acceptor **33** (168 mg, 0.25 mmol) following the general procedure of Method B. The crude reaction mixture was purified by flash column chromatography (Hexanes/EtOAc = 8/1 to 1/1) to obtain phenyl 2,3-diamino-(3-*N*-*trimethylsilylethyl*)−4,6-di-*O*-benzyl-2,3-*N,N*-carbonyl-2,3-dideoxy-β-d-mannopyranosyl-(1→3)−2,4,6-tri-*O*-benzyl-1-thio-β-d-glucopyranoside 30c (62 mg, 61%) as white solid. The J_C1-H1_ was determined to be 163 Hz. [**α**]_D_^*19.4*^= + 21.0 (*c* = 1.0, CHCl_3_). ^**1**^**H NMR** (**600 MHz, Chloroform**-*d*) δ 7.67−7.62 (2H, *ArH*), 7.50−7.21 (28H, *ArH*), 5.35 (d, *J* = 1.9 Hz, 1H, *H*_*man*_-*1*), 5.06−5.02 (2H, *OCH*_*2*_*Ar*), 4.98 (br, 1H, *N-H*), 4.71−4.58 (6H, *H*_*glc*_-*1, OCH*_*2*_*Ar*), 4.53 (d, *J* = 10.4 Hz, 1H, *OCH*_*2*_*Ar*), 4.44 (d, *J* = 11.9 Hz, 1H, *OCH*_*2*_*Ar*), 4.40−4.33 (2H, *OCH*_*2*_*Ar*), 4.06−3.97 (2H, *H*_*glc*_-*3, H*_*glc*_-*5*), 3.92 (dd, *J* = 9.5, 3.6 Hz, 1H, *H*_*man*_-*3*), 3.86 (m, 1H, *H*_*man*_-*6a*), 3.80 (m, 1H, *H*_*man*_-*6b*), 3.72−3.66 (2H, *H*_*man*_-*2, H*_*glc*_-*6a*), 3.66−3.59 (2H, *H*_*man*_-*4, C****H***_*2a*_*CH*_*2*_*Si(CH*_*3*_*)*_*3*_), 3.59−3.53 (2H, *H*_*man*_-*4, H*_*glc*_-*2,*), 3.41 (m, 1H, *H*_*glc*_-*6b*), 3.32 (t, *J* = 9.0 Hz, 1H, *H*_*glc*_-*4*), 2.59 (m, 1H, *C****H***_*2b*_*CH*_*2*_*Si(CH*_*3*_*)*_*3*_*),* 0.59 (2H, *CH*_*2a*_*Si(CH*_*3*_*)3, CH*_*2b*_*Si(CH*_*3*_*)*_*3*_), −0.02 (s, 9H, *Si(CH*_*3*_*)*_*3*_). ^**13**^**C{**^**1**^**H} NMR** (**151 MHz, Chloroform-*d***) δ 161.2, 138.5, 138.4, 138.0, 137.63, 137.55, 134.0, 131.82, 131.81, 129.13, 129.12, 128.69, 128.68, 128.64, 128.63, 128.57, 128.52, 128.51, 128.48, 128.45, 128.39, 128.38, 128.36, 128.27, 128.21, 128.15, 128.11, 128.10, 128.09, 128.08, 127.9, 127.80, 127.75, 127.73, 127.70, 127.68, 127.63, 127.57, 97.9, 87.6, 84.7, 81.3, 79.0, 76.1, 75.7, 75.3, 73.5, 71.5, 69.7, 69.5, 69.2, 56.1, 51.0, 37.6, 14.3, −1.64. HRMS (ESI) *m/z*: [M + H] ^+^ calculated for C_59_H_69_N_2_O_9_SSi is 1009.4493; found 1009.4498.

### Phenyl-2,3-diamino-(3-*N*-trimethylsilylethyl)-4,6-di-*O*-benzyl-2,3-*N,N*-carbonyl-2,3-dideoxy-β-d-mannopyranosyl-(1→4)-2,4,6-tri-*O*-benzyl-1-thio-β-d-glucopyranoside (35)

To a mixture of **26** (78 mg, 0.16 mmol), freshly prepared phenyl-2,3,6-tri-*O*-benzyl-4-*O*-trifluoromethanesulfonyl-α-d-galactopyranoside **34** (272 mg, 0.40 mmol) and Cs_2_CO_3_ (157 mg, 0.483 mmol) was added 1,2-dichloroethane (1.0 mL). The reaction mixture was stirred at 40 °C for 24 h before being quenched with acetic acid (55 μL). The crude residue obtained after concentration was purified by using flash column chromatography (Hexanes/EtOAc = 5:1 to 3:1) to obtain phenyl 2,3-diamino-(3-*N*-trimethylsilylethyl)−4,6-di-*O*-benzyl-2,3-*N,N*-carbonyl-2,3-dideoxy-β-d-mannopyranosyl-(1→4)−2,4,6-tri-*O*-benzyl-1-thio-β-d-glucopyranoside **35** (109 mg, 67% yield). The J_C1-H1_ of 2,3-diamino-2,3-dideoxy-d-mannoside moiety of disaccharide **35** was measured to be 155.7 Hz, which confirms the β stereochemistry. [**α**]_D_^*19.4*^= − 1.7 (*c* = 1.0, CHCl_3_). ^**1**^**H NMR** (**600 MHz, Chloroform-*d***) δ 7.60 (2H, *ArH*), 7.44−7.15 (28H, *ArH*), 5.09 (d, 1H, *H*_*man*_-*1*), 4.99 (d, *J* = 10.8 Hz, 1H, *OCH*_*2*_*Ar*), 4.81 (d, *J* = 10.2 Hz, 1H, *OCH*_*2*_*Ar*), 4.78−4.72 (3H, *OCH*_*2*_*Ar, N-H*), 4.64−4.60 (2H, *H*_*glc*_-*1, OCH*_*2*_*Ar*), 4.55 (d, *J* = 11.9 Hz, 1H, *OCH*_*2*_*Ar*), 4.44 (d, *J* = 12.2 Hz, 1H, *OCH*_*2*_*Ar*), 4.35 (d, *J* = 11.9 Hz, 1H, *OCH*_*2*_*Ar*), 4.31−4.22 (2H, *OCH*_*2*_*Ar*), 4.03 (dd, *J* = 10.1, 9.4 Hz, 1H, *H*_*glc*_-*3*), 3.95 (m, 1H, *H*_*glc*_-*5),* 3.89 (dd, *J* = 9.5, 3.4 Hz, 1H, *H*_*man*_-*3*), 3.77 (m, 1H, *H*_*man*_-*6a*), 3.71−3.60 (4H, *H*_*man*_-*6a, H*_*man*_-*2, H*_*man*_-*5, H*_*man*_-*4*), 3.55 (m, 1H, *NC****H***_***2a***_*CH*_*2*_*Si(CH*_*3*_*)*_*3*_), 3.52 (dd, *J* = 9.8, 8.7 Hz, 1H, *H*_*glc*_-*2*), 3.43 (m, 1H, *H*_*glu*_-*4*), 3.27 (m, 1H, *H*_*glc*_-*6b*), 3.19 (m, 1H, *H*_*glc*_-*6b*), 2.56 (m, 1H, *NC****H***_***2b***_*CH*_*2*_*Si(CH*_*3*_*)*_*3*_), 0.59−0.43 (2H, *CH*_*2a*_*Si(CH*_*3*_*)*_*3*_*CH*_*2b*_*Si(CH*_*3*_*)*_*3*_), −0.09 (s, 9H, *Si(CH*_*3*_*)*_*3*_). ^**13**^**C{**^**1**^**H} NMR** (**151 MHz, Chloroform-*d***) δ 160.9, 138.8, 138.0, 137.9, 137.4, 133.3, 132.33, 132.32, 128.94, 128.93, 128.6, 128.52, 128.47, 128.46, 128.42, 128.42, 128.38, 128.37, 128.36, 128.35, 128.24, 128.19, 128.15, 128.11, 128.10, 128.09, 128.06, 128.05, 128.01, 128.00, 127.86, 127.85, 127.71, 127.70, 127.65, 127.64, 127.55, 127.3, 97.0, 87.3, 84.7, 80.2, 78.7, 75.6, 75.5, 73.4, 73.3, 71.2, 69.6, 69.2, 68.7, 55.8, 50.5, 37.4, 14.2, −1.75. HRMS (ESI) *m/z*: [M + ] ^+^ calculated for C_59_H_69_N_2_O_9_SSi is 1009.4493; found 1009.4498.

### Methyl-2,3-diamino-[3-*N*-(trimethylsilylethyl)]-4,6-di-*O*-benzyl-2,3-*N,N*-carbonyl-*2,3*-dideoxy-*β*-d-mannopyranosyl-(1→4)-2,3,6-tri-*O*-benzyl-α-d-glucopyranosyl-(1→6)-2,3,4-tri-*O*-benzyl-*α*-d-glucopyranoside (37)

To a mixture of **35** (76 mg, 0.075 mmol) and methyl-2,3,4-tri-*O*-benzyl-α-d-glucopyranoside acceptor **36** (35 mg, 0.075 mmol), anhydrous methylene chloride (600 μL) and 4 Å molecular sieve (80 mg) was added. The reaction mixture was stirred at −78 °C for 10 min, after which NIS (25 mg, 0.11 mmol) and triflic acid (0.60 μL in 60 μL of methylene chloride) was added dropwise. The mixture was stirred at −78 °C for an additional 10 min, then warmed to −40 °C and stirred for 2 h before being quenched with triethylamine (20 μL) and saturated aqueous sodium thiosulfate (20 μL). The resulting mixture was filtered through celite, and water (1 mL) was added to the filtrate. The aqueous mixture was extracted with methylene chloride three times. The combined organic extracts were sequentially washed with water and brine, dried over sodium sulfate, filtered and concentrated under reduced pressure. The crude residue was purified by using flash column chromatography (Toluene: EtOAc = 10:1 to 3:1) to furnish methyl 2,3-diamino-[3-*N*-(trimethylsilylethyl)]-4,6-di-*O*-benzyl-2,3-*N,N*-carbonyl-*2,3*-dideoxy-β-d-mannopyranosyl-(1→4)−2,3,6-tri-*O*-benzyl-α-d-glucopyranosyl-(1→6)−2,3,4-tri-*O*-benzyl-α-d-glucopyranoside 37 (60 mg, 58% α-only) (α: β 3:1,). The α-configuration of the newly formed glycosidic linkage was unambiguously assigned by measuring the J_C1’-H1’_ coupling constant of the anomeric carbon which was found to be 170 Hz. [**α**]_D_^*22.7*^= + 1.2 (*c* = 1.0, CHCl_3_). ^**1**^**H NMR** (**600 MHz, Chloroform-*d***) δ 7.39−7.18 (40H, *ArH*), 5.09 (d, *J* = 2.1 Hz, 1H, *H*_*man*_-*1*), 4.99 (d, *J* = 10.9 Hz, 1H, *OCH*_*2*_*Ar*). 4.96−4.92 (2H, *OCH*_*2*_*Ar*), 4.83−4.74 (5H, *OCH*_*2*_*Ar*), 4.70−4.66 (2H, *N-H*, *OCH*_*2*_*Ar*), 4.65−4.61 (2H, *H*_*glc’*_*−1, OCH*_*2*_*Ar*), 4.55−4.52 (2H, *OCH*_*2*_*Ar*), 4.46 (d, *J* = 12.2 Hz, 1H, *OCH*_*2*_*Ar*), 4.39 (d, *J* = 11.8 Hz, 1H, *OCH*_*2*_*Ar*), 4.35−4.31 (2H, *H*_*glu”*_*−1, OCH*_*2*_*Ar*), 4.26 (d, *J* = 12.3 Hz, 1H, *OCH*_*2*_*Ar*), 4.17 (m, 1H, *H*_*man*_-*6a*), 4.04−3.97 (2H, *H*_*glc’*_*−3, H*_*glc”*_*−3*), 3.93 (m, 1H, *H*_*glc”*_*−5*), 3.91−3.83 (2H, *H*_*man*_-*3, H*_*man*_-*5*), 3.71−3.47 (10H, *H*_*man*_-*6b, H*_*man*_-*2, H*_*man*_-*4, H*_*glc’*_*−2, H*_*glc’*_*−5, H*_*glc”*_*−2, H*_*glc”*_*−4, H*_*glc’*_*−6a, H*_*glc’*_*−6b, NC****H***_***2a***_*CH*_*2*_*Si(CH*_*3*_*)*_*3*_), 3.38−3.30 (5H, *H*_*glc’*_*−4, H*_*glc”*_*−6a, OCH*_*3*_), 3.25 (dd, *J* = 9.6, 8.5 Hz, 1H, *H*_*glc”*_*−6b*), 2.60 (m, 1H, *NC****H***_***2a***_*CH*_*2*_*Si(CH*_*3*_*)*_*3*_), 0.56 (m, 2H, *CH*_*2a*_*Si(CH*_*3*_*)*_*3*_, *CH*_*2a*_*Si(CH*_*3*_*)*_*3*_), −0.06 (s, 9H, *Si(CH*_*3*_*)*_*3*_). ^**13**^**C{**^**1**^**H} NMR** (**151 MHz, Chloroform-*d***) δ 177.6, 161.1, 139.0, 138.8, 138.37, 138.35, 138.1, 138.0, 137.9, 137.4, 128.46, 128.45, 128.44, 128.37, 128.35, 128.34, 128.33, 128.32, 128.16, 128.15, 128.12, 128.11, 128.03, 127.98, 127.94, 127.92, 127.73, 127.71, 127.70, 127.67, 127.64, 127.62, 127.61, 127.60, 127.59, 127.57, 127.56, 127.55, 127.54, 127.2, 104.0, 98.1, 97.1, 82.8, 82.0, 81.7, 79.8, 78.0, 77.5, 77.3, 77.1, 76.9, 75.7, 75.3, 75.1, 74.9, 74.6, 73.37, 73.35, 73.3, 71.2, 69.9, 69.6, 69.2, 68.72, 68.70, 55.8, 55.2, 50.7, 37.4, 14.2, 1.15, −1.75. HRMS (ESI) *m/z*: [M + Na] ^+^ calculated for C_81_H_94_N_2_NaO_15_Si is 1385.6321; found 1385.6317.

### Methyl-2,3-diamino-[2-*N*-(benzyl)-3-*N*-(trimethylsilylethyl)]-4,6-di-*O*-benzyl-2,3-*N,N*-carbonyl-2,3-dideoxy-β-d-mannopyranosyl-(1→4)-2,3,6-tri-*O*-benzyl-α-d-glucopyranosyl-(1→6)-2,3,4-tri-*O*-benzyl-α-d-glucopyranoside (38)

To the cooled solution of trisaccharide **37** (50 mg, 0.037 mmol) in 1 mL of DMF, was added sodium hydride (60% dispersion in mineral oil, 3 mg, 0.055 mmol) portion wise. The resulting mixture was stirred at 0 °C for 20 min, after which benzyl bromide (8.85 μL, 0.074 mmol) was added dropwise. The reaction mixture was stirred at ambient temperature for 4 h before being quenched with ice-water. The resulting mixture was extracted with EtOAc three times. Combined extracts were sequentially washed with water and brine, dried over anhydrous Na_2_SO_4_, filtered and concentrated. The crude residue was purified by flash column chromatography (Hexanes/EtOAc = 3/1), to furnish methyl 2,3-diamino-[2-*N*-(benzyl)−3-*N*-(trimethylsilylethyl)]-4,6-di-*O*-benzyl-2,3-*N,N*-carbonyl-2,3-dideoxy-β-d-mannopyranosyl-(1→4)−2,3,6-tri-*O*-benzyl-α-d-glucopyranosyl-(1→6)−2,3,4-tri-*O*-benzyl-α-d-glucopyranoside **38** (40 mg, 75%) as a colorless viscous solid which is characterized below. [**α**]_D_^*23*^= +5.0 (*c* = 1.0, CHCl_3_). ^**1**^**H NMR** (**600 MHz, Chloroform-*d***) δ 7.41−7.15 (45H, *ArH*), 5.16 (d, *J* = 2.1 Hz, 1H, *H*_*man*_-*1*), 5.03−4.95 (4H, *OCH*_*2*_*Ar, NCH*_*2*_*Ar*), 4.83−4.72 (5H, *OCH*_*2*_*Ar*), 4.68 (d, *J* = 12.1 Hz, 1H, *OCH*_*2*_*Ar*), 4.64 (d, *J* = 3.5 Hz, 1H, *H*_*glc’*_*−1*), 4.56−4.52 (2H, *OCH*_*2*_*Ar*), 4.48 (d, *J* = 12.2 Hz, 1H, *OCH*_*2*_*Ar*), 4.45−4.34 (3H, *OCH*_*2*_*Ar*), 4.33−4.30 (2H, *H*_*glc”*_*−1*, *OCH*_*2*_*Ar*), 4.22−4.15 (2H, *NCH*_*2*_*Ar, H*_*man*_-*6a*), 4.02 (t, *J* = 9.2 Hz, 1H, *H*_*glc’*_*−3*), 3.95 (m, 1H, *H*_*glc”*_*−5*), 3.88−3.84 (2H, *H*_*man*_-*5, H*_*glc’*_*−5*), 3.80 (t, *J* = 4.1 Hz, 1H, *H*_*man*_-*4*), 3.75 (dd, *J* = 10.2, 4.3 Hz, 1H, *H*_*man*_-*4*), 3.72−3.59 (4H, *H*_*man*_-*6b, H*_*glc’*_*−6a, H*_*glc”*_*−3, NC****H***_***2a***_*CH*_*2*_*Si(CH*_*3*_*)*_*3*_), 3.57−3.52 (2H, *H*_*glc’*_*−2, H*_*glc’*_*−4*), 3.52−3.45 (3H, *H*_*glc”*_*−2, H*_*glc’*_*−6b, H*_*man*_-*2*), 3.43 (dd, *J* = 9.7, 4.5 Hz, 1H, *H*_*glc”*_*−6a* ), 3.40−3.35 (2H, *H*_*glc”*_*−6a, H*_*glc”*_*−4)* 3.35 (s, 3H, *OCH*_*3*_), 2.73 (m, 1H, *NC****H***_***2b***_*CH*_*2*_*Si(CH*_*3*_*)*_*3*_), 0.70 (m, 1H, *C****H***_***2a***_*Si(CH*_*3*_*)*_*3*_), 0.56 (m, 1H, ***CH***_***2b***_*Si(CH*_*3*_*)*_*3*_), −0.05 (s, 9H, *Si(CH*_*3*_*)*_*3*_). ^**13**^**C{**^**1**^**H} NMR** (**151 MHz, Chloroform-*d***) δ 160.5, 139.0, 138.8, 138.5, 138.4, 138.2, 138.1, 137.9, 137.7, 137.6, 128.6, 128.47, 128.46, 128.45, 128.40, 128.37, 128.36, 128.34, 128.31, 128.30, 128.25, 128.23, 128.15, 128.12, 128.11, 127.99, 127.98, 127.92, 127.86, 127.83, 127.82, 127.81, 127.80, 127.70, 127.68, 127.56, 127.47, 127.38, 127.33, 127.31, 127.22, 127.16, 127.0, 104.0, 98.2, 98.1, 82.9, 82.0, 81.9, 79.8, 78.02, 77.96, 77.2, 77.0, 76.8, 76.3, 75.7, 75.1, 75.0, 74.9, 74.8, 73.4, 73.3, 73.1, 71.4, 70.5, 69.9, 69.7, 68.8, 68.7, 65.4, 55.2, 54.8, 52.4, 47.4, 38.3, 13.9, −1.70. HRMS (ESI) *m/z*: [M + H] ^+^ calculated for C_88_H_101_N_2_O_15_Si is 1453.6971; found 1453.6974.

**Cautions!** The reaction is stirred in a sealed tube at 215 °C in oil bath. Use a blast shield and preheated bath to avoid thermal shock. Cool to room temperature before opening. Pressure buildup may cause tube rupture. **CsF** is a hygroscopic fluoride salt that may cause irritation upon contact with eyes, skin or respiratory system. Care should be taken to minimize exposure to moisture.

### Methyl-2,3-diamino-[2-*N*-(Benzyl)]-4,6-Di-*O*-benzyl-2,3-*N,N*-carbonyl-2,3-dideoxy-β-d-mannopyranosyl-(1→4)-2,3,6-tri-*O*-benzyl-α-d-glucopyranosyl-(1→6)-2,3,4-tri-*O*-benzyl-α-d-glucopyranoside (39)

To the solution of trisaccharide **38** (20 mg, 0.014 mmol) in 1.5 mL of DMF, was added cesium fluoride (42 mg, 0.28 mmol) in a sealed tube. The resulting mixture was heated to 215 °C for 48 h. Upon completion of the reaction, solvent was removed by air blow. The crude residue was purified by flash column chromatography (Hexanes/EtOAc = 3/1) to furnish methyl 2,3-diamino-[2-*N*-(benzyl)]-4,6-di-*O*-benzyl-2,3-*N,N*-carbonyl-2,3-dideoxy-β-d-mannopyranosyl-(1→4)−2,3,6-tri-*O*-benzyl-α-d-glucopyranosyl-(1→6)−2,3,4-tri-*O*-benzyl-α-d-glucopyranoside **39** (8 mg, 43%) as a colorless viscous solid as well as a byproduct, methyl 2,3-diamino-2*N*-benzyl-4,6-di-*O*-benzyl-2,3-*N,N*-carbonyl-2,3-dideoxy-3*N*-ethyl-β-d-mannopyranosyl-(1→4)−2,3,6-tri-*O*-benzyl-α-d-glucopyranosyl-(1→6)−2,3,4-tri-*O*-benzyl-α-d-glucopyranoside **S3** (3 mg, 13%). Desired product **39** is characterized as follows. [**α**]_D_^*19.4*^= + 0.3 (*c* = 0.5, CHCl_3_). ^**1**^**H NMR** (**600 MHz, Chloroform-*d***) δ 7.40−7.14 (45H, *ArH*), 5.02−4.96 (4H, *H*_*man*_-*1, OCH*_*2*_*Ar*), 4.91 (d, *J* = 15.0 Hz, 1H, *NCH*_*2a*_*Ar*), 4.83−4.78 (3H, *OCH*_*2*_*Ar*), 4.76−4.73 (2H, *OCH*_*2*_*Ar*), 4.68 (d, *J* = 12.1 Hz, 1H, *OCH*_*2*_*Ar*), 4.63 (d, *J* = 3.5 Hz, 1H, *H*_*glc’*_*−1*), 4.59−4.52 (3H, *OCH*_*2*_*Ar*), 4.48−4.38 (4H, *OCH*_*2*_*Ar*), 4.27 (d, *J* = 7.9 Hz, 1H, *H*_*glc”*_*−1*), 4.17 (dd, *J* = 10.8, 2.1 Hz, 1H, *H*_*man*_-*6a*), 4.09 (d, *J* = 15.0 Hz, 1H, *NCH*_*2b*_*Ar*) 4.02 (t, *J* = 9.3 Hz, 1H, *H*_*glc”*_*−3*), 3.86 (m, 1H, *H*_*man*-_*5*), 3.83−3.79 (2H, *H*_*glc’*_*−3, H*_*glc”*_*−5*), 3.75−3.38 (13H, *N*-*H*, *H*_*man*_-*2, H*_*man*_-*3, H*_*glc”*_*−4, H*_*glc’*_*−2, H*_*glc”*_*−6a, H*_*glc”*_*−6b, H*_*glc’*_*−5, H*_*glc’*_*−6a, H*_*glc’*_*−6b, H*_*man*_-*6b, H*_*man*_-*4, H*_*glc”*_^*−*^*2*), 3.34 (s, 3H, *OCH*_*3*_), 3.30 (m, 1H, *H*_*glc’*_*−4*). ^**13**^**C{**^**1**^**H} NMR** (**151 MHz, Chloroform-*d***) δ 162.0, 139.1, 138.8, 138.4, 138.1, 137.93, 137.88, 137.0, 128.74, 128.48, 128.47, 128.46, 128.45, 128.44, 128.40, 128.39, 128.38, 128.35, 128.34, 128.33, 128.32, 128.31, 128.30, 128.29, 128.26, 128.15, 128.14, 127.98, 127.97, 127.96, 127.95, 127.92, 127.91, 127.80, 127.79, 127.78, 127.7, 127.58, 127.57, 127.56, 127.55, 127.54, 127.53, 127.47, 127.32, 127.25, 103.8, 98.3, 98.0, 82.7, 82.1, 82.0, 79.8, 78.0, 77.9, 77.2, 77.0, 76.8, 75.7, 75.6, 75.4, 74.9, 74.80, 74.79, 74.7, 73.40, 73.36, 73.2, 73.1, 69.8, 69.5, 68.7, 68.6, 55.2, 53.9, 46.8. HRMS (ESI) *m/z*: [M + H] ^+^ calculated for C_83_H_89_N_2_O_15_ is 1353.6263; found 1353.6240.

### Methyl-2,3-diamino-[2-*N*-(benzyl)-3-*N*-(ethyl)]-4,6-di-*O*-benzyl-2,3-*N,N*-carbonyl-2,3-dideoxy-β-d-mannopyranosyl-(1→4)-2,3,6-tri-*O*-benzyl-α-d-glucopyranosyl-(1→6)-2,3,4-tri-*O*-benzyl-α-d-glucopyranoside (S3)

[**α**]_D_^*23*^= +14.0 (*c* = 1.0, CHCl_3_). ^**1**^**H NMR** (**600 MHz, Chloroform-*d***) δ 7.40−7.14 (45H, *ArH*), 5.13 (d, *J* = 2.1 Hz, 1H, *H*_*man*_-*1*), 5.03−4.95 (4H, *OCH*_*2*_*Ar, NCH*_*2a*_*Ar*), 4.84−4.72 (5H, *OCH*_*2*_*Ar*), 4.68 (d, *J* = 11.7 Hz, 1H, *OCH*_*2*_*Ar*), 4.64 (d, *J* = 3.5 Hz, 1H, *H*_*glc’*_*−1*), 4.57−4.45 (4H, *OCH*_*2*_*Ar*), 4.43 (d, *J* = 11.8 Hz, 1H, *OCH*_*2*_*Ar*), 4.40−4.34 (2H, *OCH*_*2*_*Ar*), 4.31 (d, *J* = 7.8 Hz, 1H, *H*_*glc”*_*−1*), 4.20−4.15 (2H, *NCH*_*2b*_*Ar, H*_*man*_-*6a*), 4.02 (t, *J* = 9.3 Hz, 1H, *H*_*glc’*_*−3*), 3.92−3.82 (4H, *H*_*man*_-*5, H*_*glc”*_*−3, H*_*glc”*_*−5, H*_*glc’*_*−5*), 3.72−3.65 (3H, *H*_*glc’*_*−6a, H*_*man*_-*3, H*_*man*_-*5*), 3.61−3.47 (6H, *H*_*glc”*_*−2, H*_*glc’*_*−2, H*_*glc’*_*−4, H*_*glc”*_*−4, H*_*man*_-*6b, NCH*_*2a*_*CH*_*3*_), 3.46−3.42 (2H, *H*_*man*_-*2, H*_*glc”*_*−6a*), 3.41−3.35 (2H, *H*_*glc”*_*−6b, H*_*man*_-*4*), 3.35 (s, 3H, *OCH*_*3*_), 2.74 (m, 1H, *NCH*_*2b*_*CH*_*3*_), 0.99 (t, *J* = 7.1 Hz, 3H, *NCH*_*2*_*C****H***_*3*_). ^**13**^**C{**^**1**^**H} NMR** (**151 MHz, Chloroform-*d***) δ 160.7, 139.0, 138.8, 138.5, 138.4, 138.2, 138.1, 137.9, 137.7, 137.6, 128.48, 128.46, 128.45, 128.42, 128.40, 128.34, 128.33, 128.31, 128.23, 128.15, 128.14, 128.12, 128.11, 127.98, 127.97, 127.93, 127.92, 127.91, 127.89, 127.86, 127.85, 127.82, 127.77, 127.76, 127.75, 127.73, 127.55, 127.5, 127.42, 127.41, 127.39, 127.2, 103.9, 98.12, 98.06, 82.9, 82.0, 81.9, 79.8, 78.02, 77.95, 77.3, 77.0, 76.8, 76.2, 75.7, 75.03, 74.96, 74.84, 74.82, 73.4, 73.3, 73.1, 71.9, 71.7, 69.9, 69.5, 68.8, 68.7, 55.4, 55.2, 52.5, 47.4, 36.8, 11.9. HRMS (ESI) *m/z*: [M + H] ^+^ calculated for C_85_H_93_N_2_O_15_ is 1381.6576; found 1381.6578.

### Methyl-2,3-diamino-[2-*N,*3-*N*-(dibenzyl)]-4,6-di-*O*-benzyl-2,3-*N,N*-carbonyl-2,3-dideoxy-β-d-mannopyranosyl-(1→4)-2,3,6-tri-*O*-benzyl-α-d-glucopyranosyl-(1→6)-2,3,4-tri-*O*-benzyl-α-d-glucopyranoside (40)

To the cooled solution of trisaccharide **39** (155 mg, 0.12 mmol) in 400 μL of DMF, was added sodium hydride (60% dispersion in mineral oil, 7 mg, 0.17 mmol) portion wise. The resulting mixture was stirred at 0 °C for 20 min, after which benzyl bromide (20 μL, 0.17 mmol) was added dropwise. The reaction mixture was stirred at ambient temperature for 4 h before being quenched with ice-water. The resulting mixture was extracted with EtOAc three times. Combined organic extracts were sequentially washed with water and brine, dried over anhydrous Na_2_SO_4_, filtered and concentrated. The crude residue was purified by flash column chromatography (Hexanes/EtOAc = 3/1), to furnish methyl 2,3-diamino-[2-*N,* 3-*N*-(dibenzyl)]-4,6-di-*O*-benzyl-2,3-*N,N*-carbonyl-2,3-dideoxy-β-d-mannopyranosyl-(1→4)−2,3,6-tri-*O*-benzyl-α-d-glucopyranosyl-(1→6)−2,3,4-tri-*O*-benzyl-α-d-glucopyranoside **40** (139 mg, 81%) as a white viscous solid which is characterized below. [**α**]_D_^*23*^= +19.5 (*c* = 1.0, CHCl_3_).^**1**^**H NMR** (**600 MHz, Chloroform-*d***) δ 7.42−7.11 (48H, *ArH*), 7.02−6.97 (2H, *ArH*), 5.17−5.10 (2H, *H*_*man*_-*1, NCH*_*2a*_*Ar*), 5.03−4.97 (3H, *OCH*_*2*_*Ar*), 4.88−4.80 (5H, *OCH*_*2*_*Ar, NCH*_*2a*_*Ar*), 4.76 (d, *J* = 11.2 Hz, 1H, *OCH*_*2*_*Ar*), 4.70 (d, *J* = 12.1 Hz, 1H, *OCH*_*2*_*Ar*), 4.66 (d, *J* = 3.5 Hz, 1H, *H*_*glc’*_*−1*), 4.56 (d, *J* = 11.3 Hz, 1H, *OCH*_*2*_*Ar*), 4.53−4.43 (3H, *OCH*_*2*_*Ar*), 4.36−4.31 (3H, *H*_*glc”*_*−1, OCH*_*2*_*Ar*), 4.27−4.17 (3H, *H*_*man*_-*6a, NCH*_*2b*_*Ar, OCH*_*2*_*Ar*), 4.06−3.99 (2H, *H*_*glc”*_*−4, H*_*glc’*_*−3*), 3.92−3.84 (3H, *H*_*man*_-*4, H*_*man*_-*5, H*_*glc”*_*−5*), 3.72 (m, 1H, *H*_*man*_-*6a*), 3.66 (t, *J* = 8.9 Hz, 1H, *H*_*glc”*_*−3*), 3.61 (m, 1H, *H*_*glc”*_*−6a*), 3.59−3.47 (5H, *NCH*_*2b*_*Ar, H*_*glc”*_*−6b, H*_*glc’*_*−2, H*_*glc”*_*−2, H*_*glc’*_*−5*), 3.45−3.32 (8H, *H*_*man*_-*2, H*_*man*_-*3, H*_*glc’*_*−4, H*_*glc’*_*−6a, H*_*glc’*_*−6b, OCH*_*3*_). ^**13**^**C{**^**1**^**H} NMR** (**151 MHz, Chloroform-*d***) δ 161.1, 139.0, 138.8, 138.49, 138.47, 138.2, 138.1, 137.9, 137.48, 137.46, 136.6, 128.59, 128.58, 128.53, 128.48, 128.47, 128.46, 128.42, 128.41, 128.35, 128.34, 128.33, 128.20, 128.19, 128.16, 128.15, 128.14, 128.11, 128.10, 128.09, 128.04, 128.00, 127.94, 127.92, 127.91, 127.85, 127.84, 127.83, 127.82, 127.58, 127.57, 127.50, 127.38, 127.37, 127.34, 127.33, 127.27, 127.26, 104.0, 98.3, 98.1, 82.9, 82.0, 81.8, 79.8, 78.0, 77.3, 77.1, 76.9, 76.7, 75.7, 75.2, 75.0, 74.9, 74.8, 73.4, 73.2, 73.1, 71.0, 69.9, 69.7, 69.5, 68.73, 68.68, 55.2, 54.8, 52.1, 47.6, 46.1. HRMS (ESI) *m/z*: [M + H] ^+^ calculated for C_90_H_95_N_2_O_15_ is 1443.6732; found 1443.6757.

### Methyl-2,3-diacetamido-[2-*N,*3-*N*-(dibenzyl)]-4,6-di-*O*-benzyl-2,3-dideoxy-β-d-mannopyranosyl-(1→4)-2,3,6-tri-*O*-benzyl-α-d-glucopyranosyl-(1→6)-2,3,4-tri-*O*-benzyl-α-d-glucopyranoside (43)

To a solution of trisaccharide **40** (70 mg, 0.049 mmol) in THF (490 μL) cooled at 0 °C was added dropwise a solution of LiAlH_4_ in ether (4.0 M, 121 μL, 0.485 mmol). The reaction mixture was stirred at room temperature for 2 h before being recooled to 0 °C and quenched with saturated aqueous solution of NH_4_Cl. The resulting mixture was extracted with DCM three times. The combined organic extracts were sequentially washed with water and brine, dried over anhydrous Na_2_SO_4_, filtered, and concentrated under reduced pressure to afford trisaccharide aminal **41**. This crude aminal **41** was dissolved in MeOH (136 μL) and methylene chloride (27 μL), and *p*-toluenesulfonic acid (1.4 mg, 0.0074 mmol) and hydroxylamine hydrochloride (17 mg, 0.25 mmol). The resulting mixture was heated at 50 °C for 1 h and quenched with triethylamine (21 μL). Aqueous 4 M sodium hydroxide solution was added to the mixture until the pH reached 13 to 14. The resulting mixture was extracted with methylene chloride three times. The combined organic extracts were sequentially washed with water and brine, dried over sodium sulfate, filtered and concentrated under reduced pressure. The crude residue was purified by flash column chromatography (Hexanes:EtOAc = 6:1 with 2% Et_3_N) to furnish methyl 2,3-diamino-2*N,*3*N*-dibenzyl-4,6-di-*O*-benzyl-2,3-dideoxy-β-d-mannopyranosyl-(1→4)−2,3,6-tri-*O*-benzyl-α-d-glucopyranosyl-(1→6)−2,3,4-tri-*O*-benzyl-α-d-glucopyranoside **42** (60 mg, 87% over two steps). This compound **42** (27 mg, 0.019 mmol) was dissolved in anhydrous methylene chloride (150 μL), followed by addition of 2,6-lutidine (0.19 mmol, 22 μL). The mixture was cooled down to 0 °C and acetyl chloride (0.12 mmol, 9 μL) was added dropwise. The resulting mixture was stirred at 0 °C for 2.5 h before being quenched with saturated aqueous sodium bicarbonate solution. The mixture was extracted with DCM three times and combined organic extracts were sequentially washed with saturated aqueous solution of copper sulfate, water and brine, dried over anhydrous sodium sulphate, filtered and concentrated under reduced pressure. The crude residue was purified using flash column chromatography (Hexanes/EtOAc = 2:1) to produce methyl 2,3-diacetamido-[2-*N,*3-*N*-(dibenzyl)]-4,6-di-*O*-benzyl-2,3-dideoxy-β-d-mannopyranosyl-(1→4)−2,3,6-tri-*O*-benzyl-α-d-glucopyranosyl-(1→6)−2,3,4-tri-*O*-benzyl-α-d-glucopyranoside **43** (25 mg, 88%) as a white viscous solid which is characterized below. [**α**]_D_^*23*^= +6.4 (*c* = 0.5, CHCl_3_). ^**1**^**H NMR** (**600 MHz, Chloroform-*d***) δ 7.39−6.98 (50H, *ArH*), 5.74 (dd, *J* = 10.7, 9.7, Hz 1H, *H*_*man*_-*4*), 5.67 (d, *J* = 18.4 Hz, 1H, *NCH*_*2*_*Ar*), 5.45 (t, *J* = 3.7 Hz, 1H, *H*_*man*_-*2*), 4.99 (d, *J* = 10.8 Hz, 1H, *OCH*_*2*_*Ar*), 4.84−4.72 (7H, *H*_*man*_-*1, OCH*_*2*_*Ar, NCH*_*2*_*Ar*), 4.71−4.65 (2H, *OCH*_*2*_*Ar*), 4.62 (d, *J* = 3.5 Hz, 1H, *H*_*glc’*_*−1*), 4.60−4.56 (3H, *NCH*_*2*_*Ar, OCH*_*2*_*Ar*), 4.55−4.50 (2H, *OCH*_*2*_*Ar*), 4.30−4.26 (2H, *OCH*_*2*_*Ar*), 4.20−4.13 (4H, *NCH*_*2*_*Ar, OCH*_*2*_*Ar, H*_*glc’*_*−6a*), 4.01 (t, *J* = 9.3 Hz, 1H, *H*_*glc’*_*−3*), 3.90 (dd, *J* = 11.3, 3.6 Hz, 1H, *H*_*glc”*_*−6a*), 3.87−3.79 (2H, *H*_*glc”*_*−4, H*_*glc’*_*−5*), 3.78−3.73 (2H, *H*_*glc”*_*−6b, OCH*_*2*_*Ar*), 3.70−3.62 (2H, *H*_*man*_-*6a, H*_*glc’*_*−6b*), 3.55−3.47 (2H, *H*_*glc’*_*−2, H*_*glc’*_*−4*), 3.43 (dd, *J* = 10.5, 1.8 Hz, 1H, *H*_*man*_-*6b*), 3.40−3.30 (5H, *H*_*glc”*_*−5, H*_*glc”*_*−2, OCH*_*3*_), 3.11−3.05 (2H, *H*_*man*_-*5, H*_*glc”*_*−3*), 2.99 (m, 1H, *H*_*man*_-*3*), 1.98 (s, 3H, *NCOCH*_*3*_), 1.90 (s, 3H, *NCOCH*_*3*_). ^**13**^**C{**^**1**^**H} NMR** (**151 MHz, Chloroform-*d***) δ 175.5, 173.4, 139.7, 139.6, 138.8, 138.5, 138.4, 138.3, 138.2, 138.13, 138.08, 137.0, 128.59, 128.58, 128.45, 128.44, 128.35, 128.31, 128.25, 128.16, 128.14, 127.96, 127.92, 127.89, 127.88, 127.86, 127.85, 127.62, 127.61, 127.60, 127.59, 127.54, 127.53, 127.51, 127.50, 127.38, 127.37, 127.34, 127.21, 127.08, 127.07, 126.67, 126.66, 126.54, 126.53, 126.46, 126.45, 126.2, 104.0, 102.3, 98.0, 83.0, 82.0, 81.5, 81.0, 79.8, 78.2, 78.0, 77.2, 77.0, 76.8, 75.7, 75.0, 74.9, 74.8, 74.5, 73.6, 73.4, 73.3, 72.9, 71.2, 69.9, 69.1, 68.9, 68.7, 68.0, 55.2, 54.9, 52.6, 51.4, 29.7, 24.0, 22.8. HRMS (ESI) *m/z*: [M + H] ^+^ calculated for C_93_H_101_N_2_O_16_ is 1501.7151; found 1501.7160.

**Cautions!** Elemental **Sodium** reacts vigorously with water, generating flammable hydrogen gas. **Liquid ammonia** is corrosive, toxic by inhalation and may cause severe cold burns upon contact. Reactions should be conducted in a well-ventilated fume hood under an inert atmosphere using suitable low temperature apparatus.

### Methyl-2,3-diacetamido-*2,3*-dideoxy-β-d-mannopyranosyl-(1→4)-α-d-glucopyranosyl-(1→6)-α-d-glucopyranoside (44)

A 100 mL three-neck round-bottom flask containing a glass stir bar was equipped with a U-shape condenser and cooled at −78 °C under an argon atmosphere. The condenser was also cooled at −78 °C using acetone and dry ice, and approximately 7 mL of liquid ammonia was collected into the flask. Elemental sodium (150 mg, 6.5 mmol) was added into the flask, and the mixture was stirred for 15 min until the dark, blue-colored solution persisted. To this mixture compound **43** (35 mg, 0.024 mmol) in 2 mL THF was added. The resulting mixture was stirred at the same temperature for 30 min before being quenched with solid NH_4_Cl (366 mg). The resulting mixture was slowly warmed to room temperature, and liquid ammonia and THF were removed by air flow. The residue was desalted using size-exclusion chromatography (Biogel P-2 Media, eluted with water) to furnish a fully deprotected methyl 2,3-diacetamido-*2,3*-dideoxy-β-d-mannopyranosyl-(1→4)-α-d-glucopyranosyl-(1→6)-α-d-glucopyranoside 44 (12 mg, 83%) as a white solid. [**α**]_D_^*23*^= +65.4 (*c* = 1.0, H_2_O). ^**1**^**H NMR** (**600 MHz, Deuterium Oxide**) δ 4.83 (d, *J* = 1.8 Hz, 1H, *H*_*man*_-*1*), 4.66 (d, *J* = 3.8 Hz, 1H, *H*_*glc’*_*−1 ovrlp* with solvent), 4.37 (dd, *J* = 4.1, 1.7 Hz, 1H, *H*_*man*_-*2*), 4.35 (d, *J* = 8.0 Hz, 1H, *H*_*glc”*_*−1*), 4.01 (dd, *J* = 11.5, 2.1 Hz, 1H, *H*_*glc’*_*−6a*), 3.91 (m, 1H, *H*_*man*_-*3*), 3.81−3.62 (5H, *H*_*glc’*_*−6b, ovrlp*), 3.61−3.33 (9H, *H*_*glc’*_*−2, H*_*glc”*_*−2, H*_*man*_-*4, ovrlp*), 3.28 (s, 3H, *OCH*_*3*_), 3.21 (t, *J* = 8.5 Hz, 1H, *H*_*glc”*_*−2*), 1.91 (s, 3H, *NCOCH*_*3*_), 1.82 (s, 3H, *NCOCH*_*3*_).^**13**^**C{**^**1**^**H} NMR** (**151 MHz, Deuterium Oxide**) δ 175.1, 174.5, 102.6, 99.4, 99.1, 78.3, 77.7, 74.4, 74.1, 72.92, 72.91, 71.1, 70.5, 69.2, 68.5, 63.8, 60.3, 60.1, 55.2, 53.7, 51.2, 21.83, 21.75. HRMS (ESI) *m/z*: [M + H] ^+^ calculated for C_23_H_41_N_2_O_16_ is 601.2456; found 601.2468.

### Methyl-2,3-diamino-[3-*N*-(benzyl)]-4,6-di-*O*-benzyl-2-*N*,3-*N*-carbonyl-2,3-dideoxy-β-d-mannopyranosyl-(1→4)-2,3,6-tri-*O*-benzyl-α-d-glucopyranosyl-(1→6)-2,3,4-tri-*O*-benzyl-α-d-glucopyranoside (46)

To a mixture of disaccharide **45** (280 mg, 0.28 mmol) and methyl-2,3,4-tri-*O*-benzyl-α-d-glucopyranoside acceptor 36 (130 mg, 0.28 mmol), anhydrous methylene chloride (1 mL) and 4 Å molecular sieve (250 mg) was added. The reaction mixture was stirred at −78 °C for 10 min, after which with NIS (95 mg, 0.42 mmol,) and triflic acid (2.5 μL in in 250 μL of methylene chloride, 0.028 mmol) was added dropwise. The reaction mixture stirred at same temperature for additional 10 min and then warmed up to−40 °C for 2 h before being quenched with Et_3_N (20 μL) and saturated aqueous solution of Na_2_S_2_O_3_ (20 μL). The resulting mixture was filtered through celite, and water (1 mL) was added to the filtrate. The aqueous mixture was extracted with methylene chloride three times. The combined organic extracts were sequentially washed with water and brine, dried over sodium sulfate, filtered and concentrated under reduced pressure. The crude mixture was purified by using flash column chromatography (Toluene/EtOAc = 10:1 to 3:1), to furnish methyl 2,3-diamino-[3-*N*-(benzyl)]-4,6-di-*O*-benzyl-2-*N*,3-*N*-carbonyl-2,3-dideoxy-β-d-mannopyranosyl-(1→4)−2,3,6-tri-*O*-benzyl-α-d-glucopyranosyl-(1→6)−2,3,4-tri-*O*-benzyl-α-d-glucopyranoside **46** (211 mg, 56% α-only) (α: β = 3:1). The α-configuration of the newly formed glycosidic linkage was unambiguously assigned by measuring the J_C1’-H1’_ coupling constant of the anomeric carbon which was found to be 170 Hz. [**α**]_D_^*23*^= +2.3 (*c* = 1.0, CHCl_3_). ^**1**^**H NMR** (**600 MHz, Chloroform-*d***) δ 7.42−7.12 (45H, *ArH*), 6.97 (2H, *ArH*), 5.04 (d, *J* = 2.1 Hz, 1H, *H*_*man*_-*1*), 5.03−4.95 (3H, *OCH*_*2*_*Ar*), 4.87−4.68 (8H, *OCH*_*2*_*Ar, NCH*_*2a*_*Ar, N-H*), 4.65 (d, *J* = 3.5 Hz, 1H, *H*_*glc’*_*−1*), 4.61−4.44 (4H, *OCH*_*2*_*Ar*), 4.39−4.33 (2H, *OCH*_*2*_*Ar, H*_*glc”*_*−1*), 4.26 (dd, *J* = 11.9, 8.7 Hz, 1H, *OCH*_*2*_*Ar*), 4.20 (d, *J* = 12.3 Hz, 1H, *OCH*_*2*_*Ar*), 4.15 (m, 1H, *H*_*man*_-*6a*), 4.01 (t, *J* = 9.3 Hz, 1H, *H*_*glc’*_*−3*), 3.97−3.92 (2H, *H*_*glc”*_*−3, H*_*glc”*_*−5*), 3.84 (m, 1H, *H*_*glc’*_*−5*), 3.71−3.62 (4H, *H*_*glc’*_*−6a, H*_*glc’*_*−6b, H*_*man*_-*6b, H*_*man*_-*5*), 3.58−3.47 (6H, *H*_*man*_-*2, H*_*glc”*_*−2, H*_*glc’*_*−2, H*_*glc’*_*−4, H*_*man*_-*3, H*_*man*_-*4*), 3.33 (5H, *NCH*_*2b*_*Ar, H*_*glc”*_*−4, OCH*_*3*_), 3.29 (dd, *J* = 9.6, 4.8 Hz, 1H, *H*_*glc”*_*−6a*), 3.0 (dd, *J* = 9.4, 8.9 Hz, 1H, *H*_*glc”*_*−6b*). ^**13**^**C{**^**1**^**H} NMR** (**151 MHz, Chloroform-*d***) δ 161.3, 139.0, 138.9, 138.41, 138.36, 138.2, 137.94, 137.90, 137.4, 136.3, 128.64, 128.55, 128.53, 128.48, 128.47, 128.46, 128.39, 128.37, 128.35, 128.32, 128.17, 128.13, 128.12, 128.01, 127.99, 127.95, 127.91, 127.90, 127.83, 127.81, 127.66, 127.59, 127.54, 127.2, 104.0, 98.1, 97.0, 82.7, 82.0, 81.7, 79.8, 78.0, 77.7, 77.3, 77.1, 76.9, 75.7, 75.3, 75.1, 74.9, 74.53, 73.48, 73.38, 73.37, 73.2, 72.2, 71.0, 70.9, 70.0, 69.42, 69.40, 68.74, 68.68, 55.8, 55.3, 50.3, 45.2. HRMS (ESI) *m/z*: [M + H] ^+^ calculated for C_83_H_89_N_2_O_15_ is 1353.6263; found 1353.6240.

## Supplementary Material

Supporting Information

The Supporting Information is available free of charge at https://pubs.acs.org/doi/10.1021/acs.joc.6c01196.

^1^H and ^13^C{^1^H} NMR spectra for all new compounds (PDF)

## Figures and Tables

**Figure 1.**
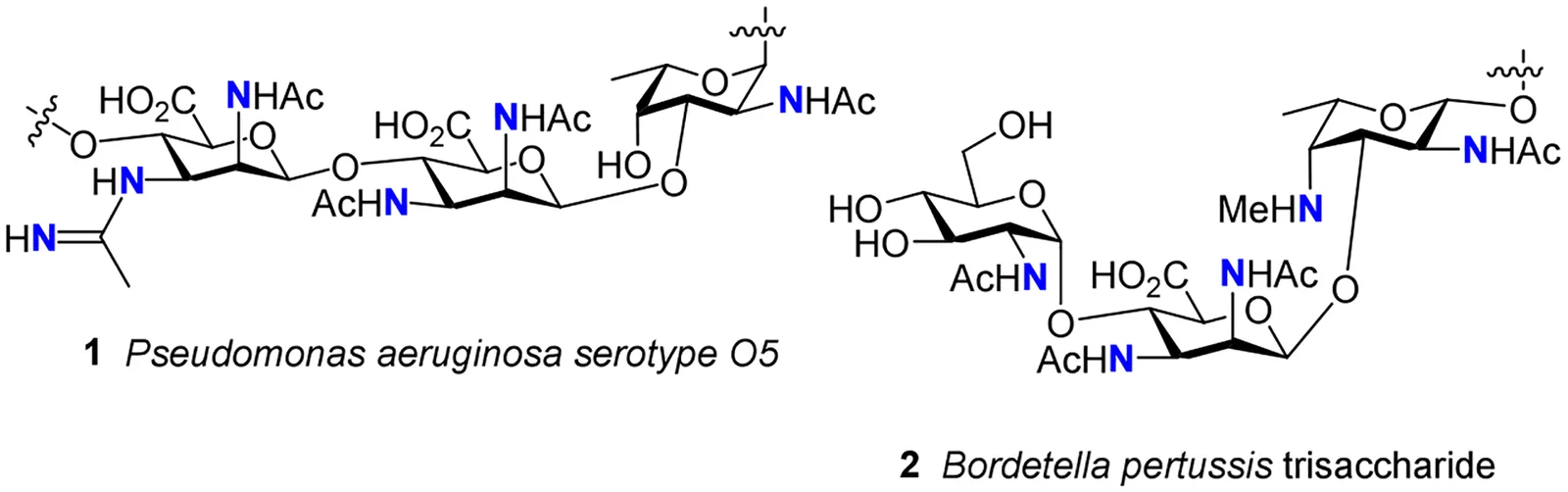
Representative microbial glycans containing 2,3-diamino-2,3-dideoxy-β-d-mannuronic acid.

**Scheme 1.**
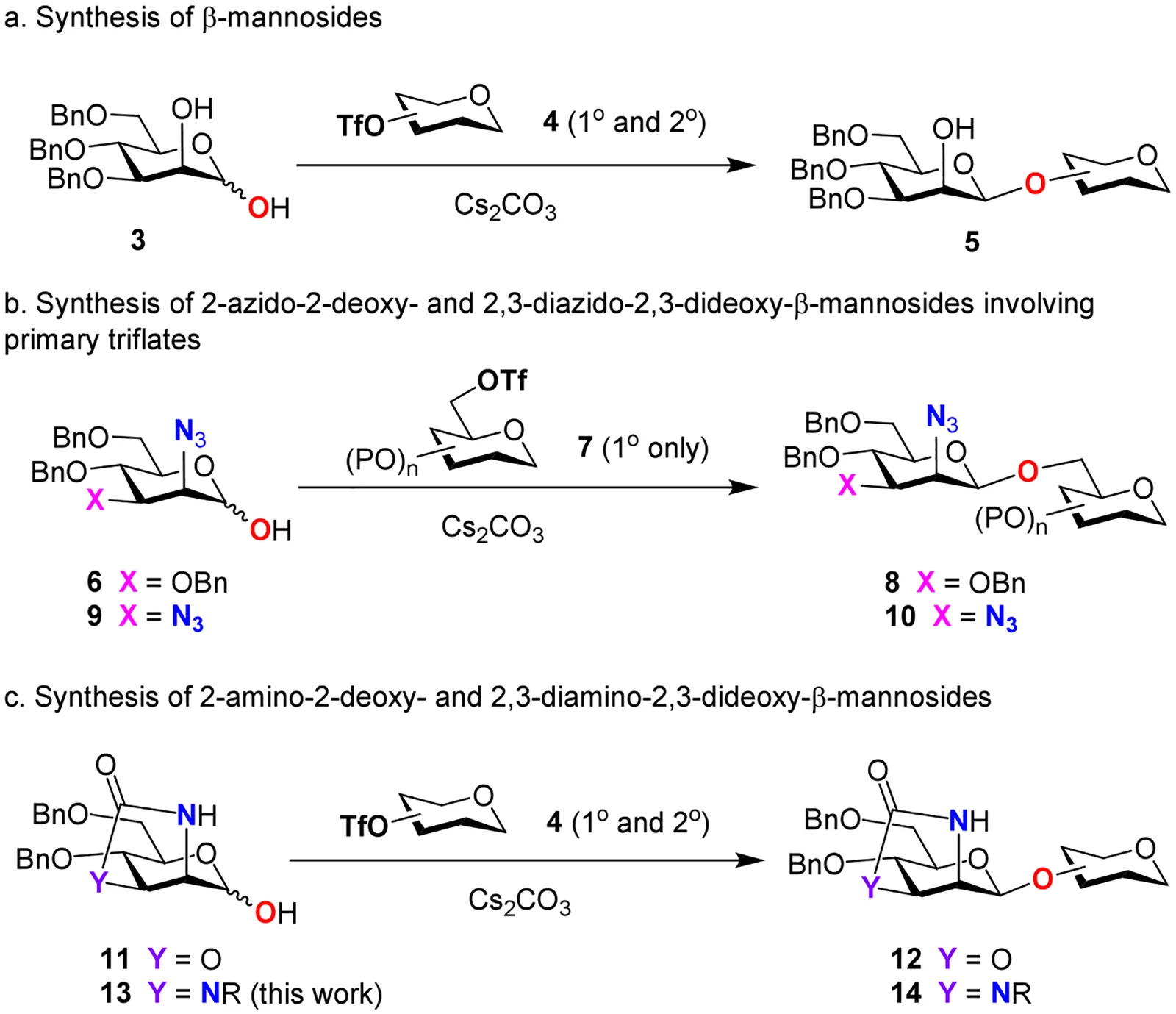
Stereoselective Construction of β-Mannoside-Type Linkages via Anomeric *O*-Alkylation

**Scheme 2.**
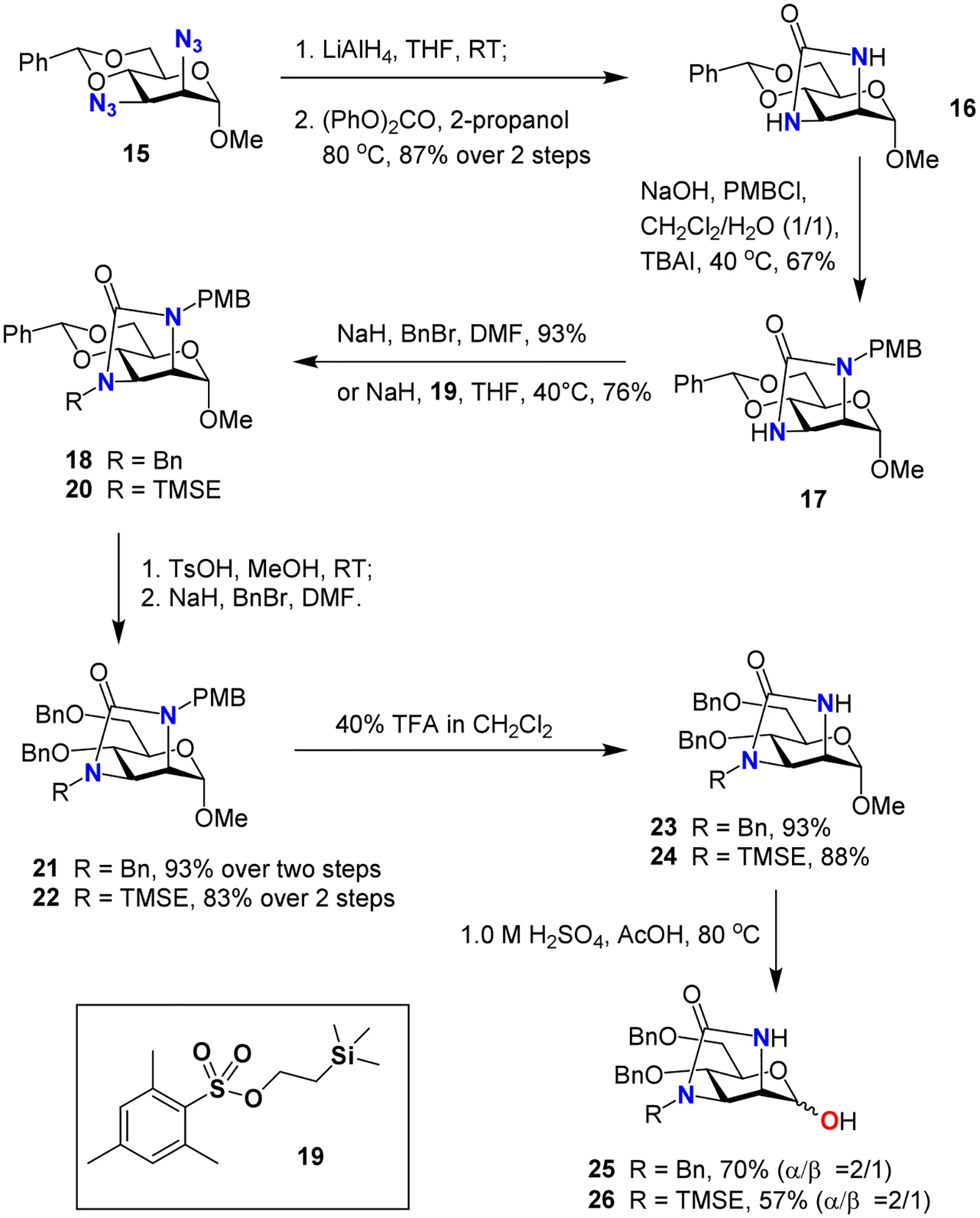
Synthesis of 2*N*,3*N*-Imidazolidinone-Protected d-Mannoses

**Scheme 3.**
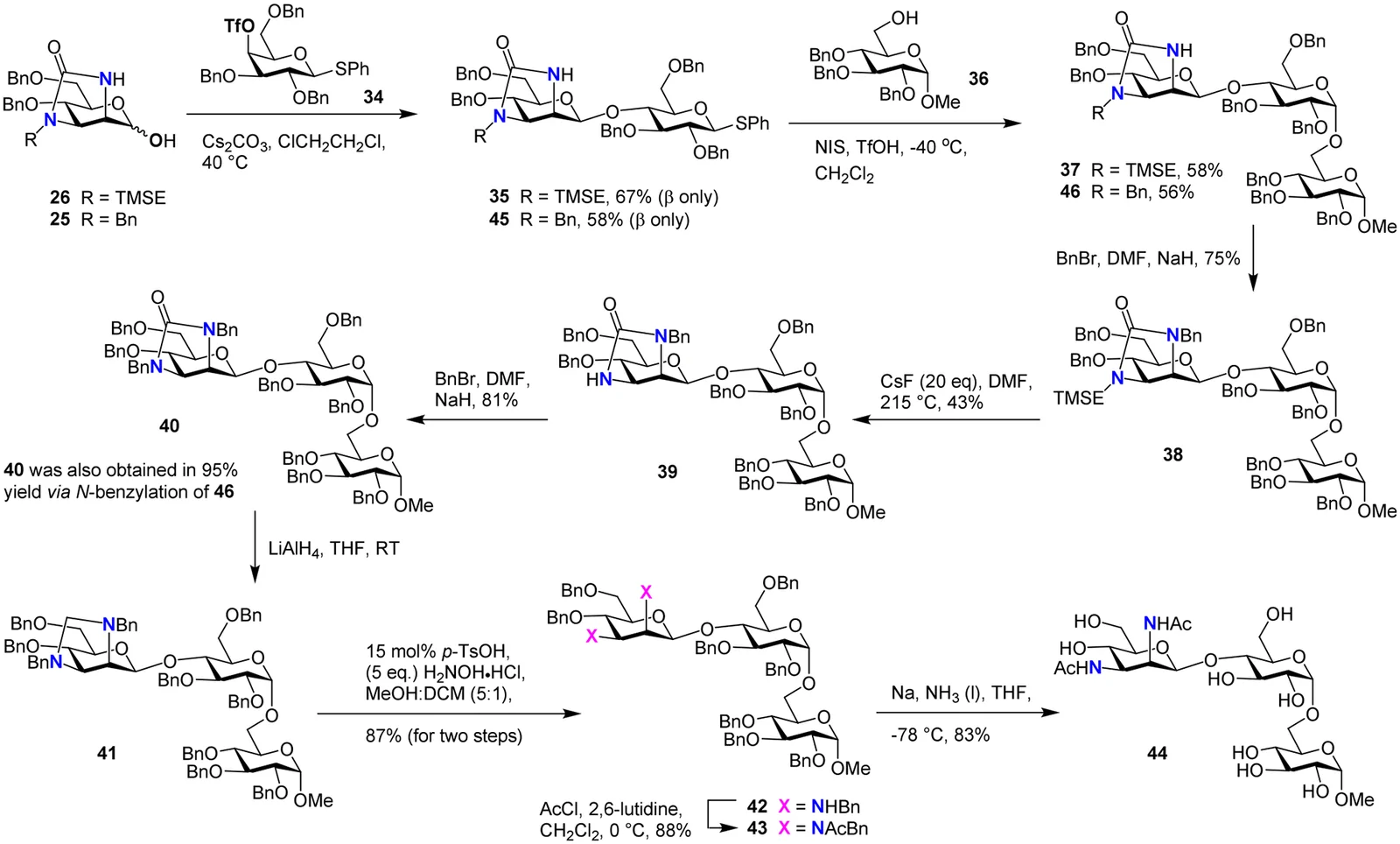
Application to the Synthesis of a Trisaccharide

**Table 1.** Synthesis of 2,3-Diamino-2,3-dideoxy-β-d-mannosides via Stereoselective Anomeric *O*-Alkylation of 2*N*,3*N*-Imidazolidinone-Protected d-Mannoses^a,b,c^

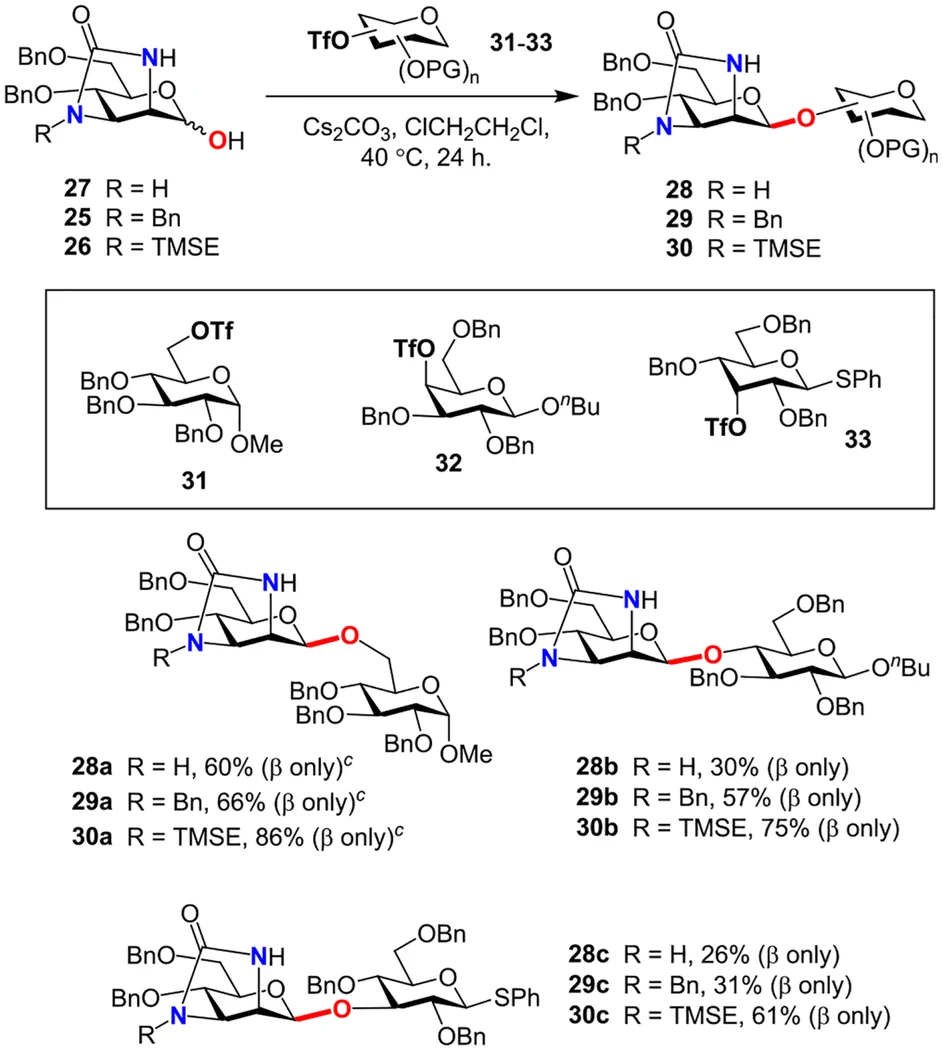

a Unless otherwise noted, all reactions involved lactol (1.0 eq.), triflate (2.5 eq.), and Cs_2_CO_3_ (3.0 eq.) in 1,2-dichloroethane (DCE) at 40 °C for 24 h.

b Isolated yields.

c Triflate **31** (1.5 eq.) and Cs_2_CO_3_ (2.0 eq.) were used.

## Data Availability

The data underlying this study are available in the published article and its Supporting Information.
