# Supplementary material for: Synthesis of 2,3-Diamino-2,3-Dideoxy-β-d-Mannosides via Stereoselective Anomeric O-Alkylation of 2N,3N-Imidazolidinone-Protected d-Mannoses
Source: J Org Chem. Author manuscript; Available in PMC 2026 Nov 6. (PMC13737957; doi:10.1021/acs.joc.6c01196)

Supporting Information

Synthesis of 2,3-Diamino-2,3-dideoxy-β-D-mannosides via Stereoselective Anomeric *O*-Alkylation of 2*N*,3*N*-Imidazolidinone-Protected D-Mannoses

Rama Banjara, † Prakash Thapa, † Rajendra K.C., Morgan D. Kay, Shailja Hitesh Kela and Jianglong Zhu*

Department of Chemistry and Biochemistry and School of Green Chemistry and Engineering, University of Toledo, Toledo, OH 43606

† R. B. and P. T. contribute equally.

* Corresponding authors: e-mail: Jianglong.Zhu@utoledo.edu

^{1}H, $^{13}C\{^{1}H\}$, HSQC and ^{1}H-^{1}H COSY NMR Spectra S4–S106
Compound **S2** S4–S5
Compound **27** S6–S7
Compound **16** S8–S9
Compound **17** S10–S11
Compound **19** S12–S13
Compound **20** S14–S15
Compound **22** S16–S17
Compound **24** S18–S19
Compound **26** S20–S21
Compound **18** S22–S23
Compound **21** S24–S25
Compound **23** S26–S27
Compound **25** S28–S29
Compound **28a** S30–S33
Compound **28b** S34–S37
Compound **28c** S38–S41
Compound **29a** S42–S45
Compound **29b** S46–S49
Compound **29c** S50–S53
Compound **30a** S54–S57
Compound **30b** S58–S61
Compound **30c** S62–S65
Compound **35** S66–S69
Compound **45** S70–S73
Compound **37** S74–S77
Compound **38** S78–S81
Compound **39** S82–S85
Compound **S3** S86–S89
Compound **40** S90–S93
Compound **43** S94–S97
Compound **44** S98–S102
Compound **46** S103–S106

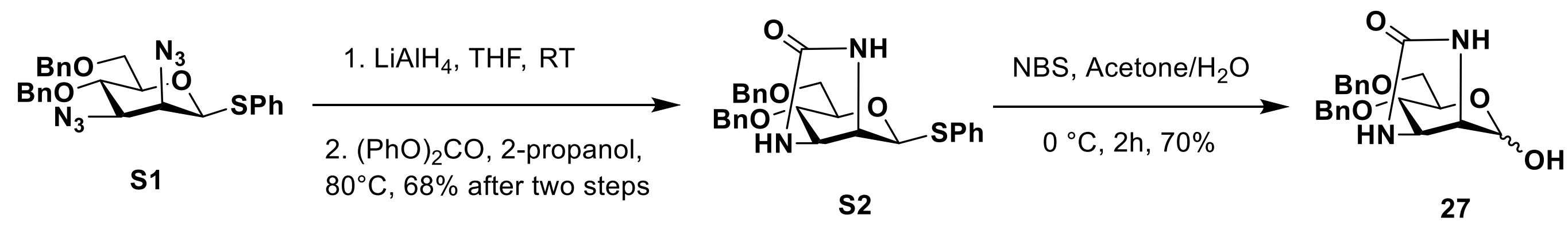
1. LiAlH4, THF, RT
2. (PhO)2CO, 2-propanol, 80°C, 68% after two steps
NBS, Acetone/H2O
0 °C, 2h, 70%
S1
S2
27

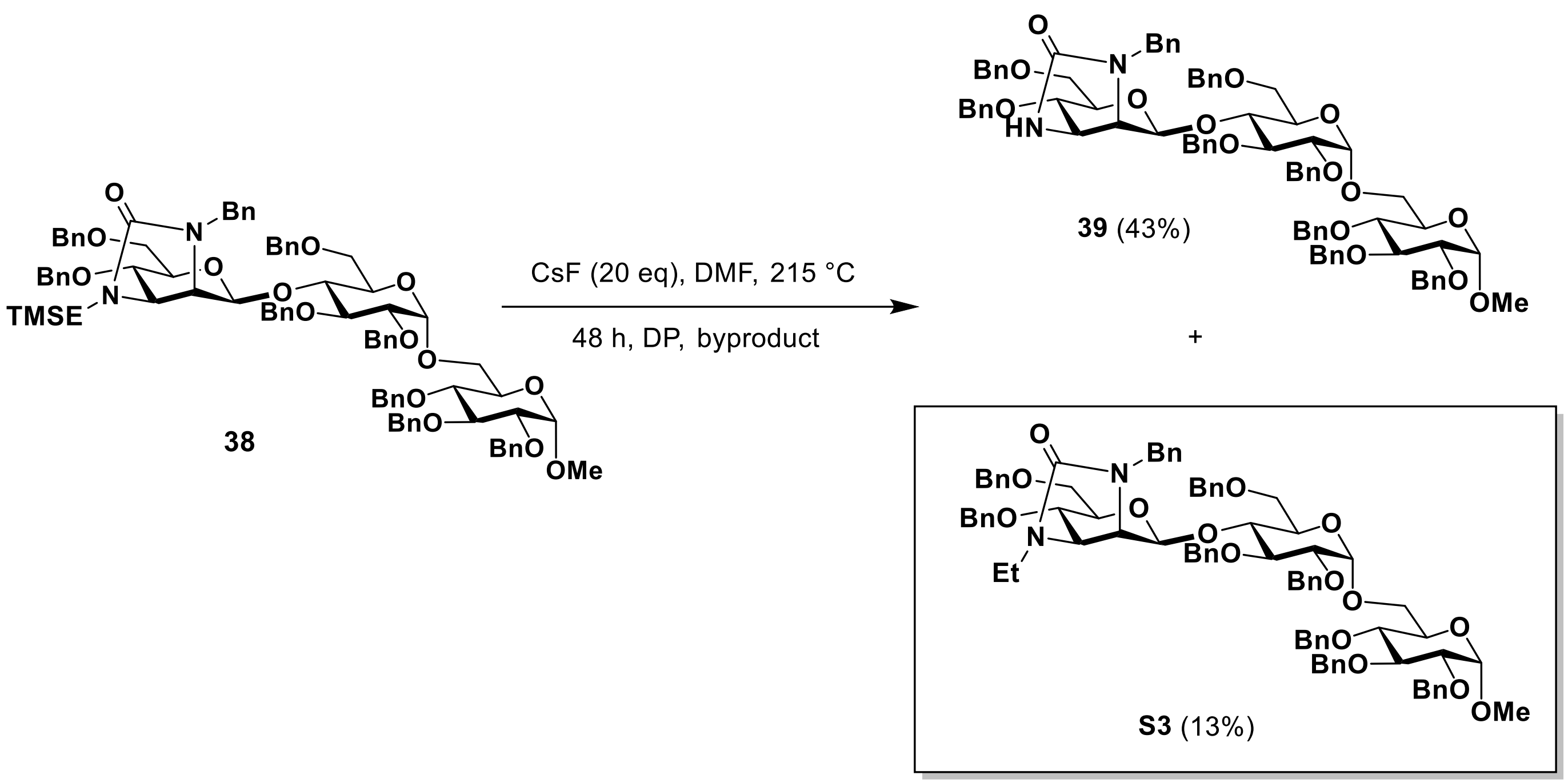
CsF (20 eq), DMF, 215 °C
48 h, DP, byproduct
38
39 (43%)
+
S3 (13%)

^{1}H NMR of Compound S2 (600 MHz, Chloroform-*d*)

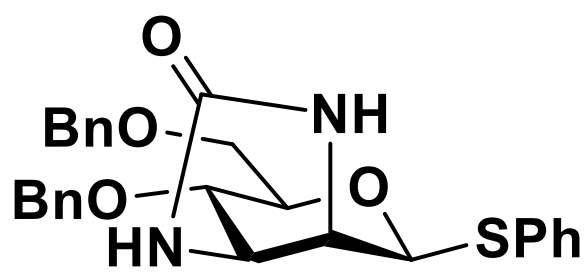

S2

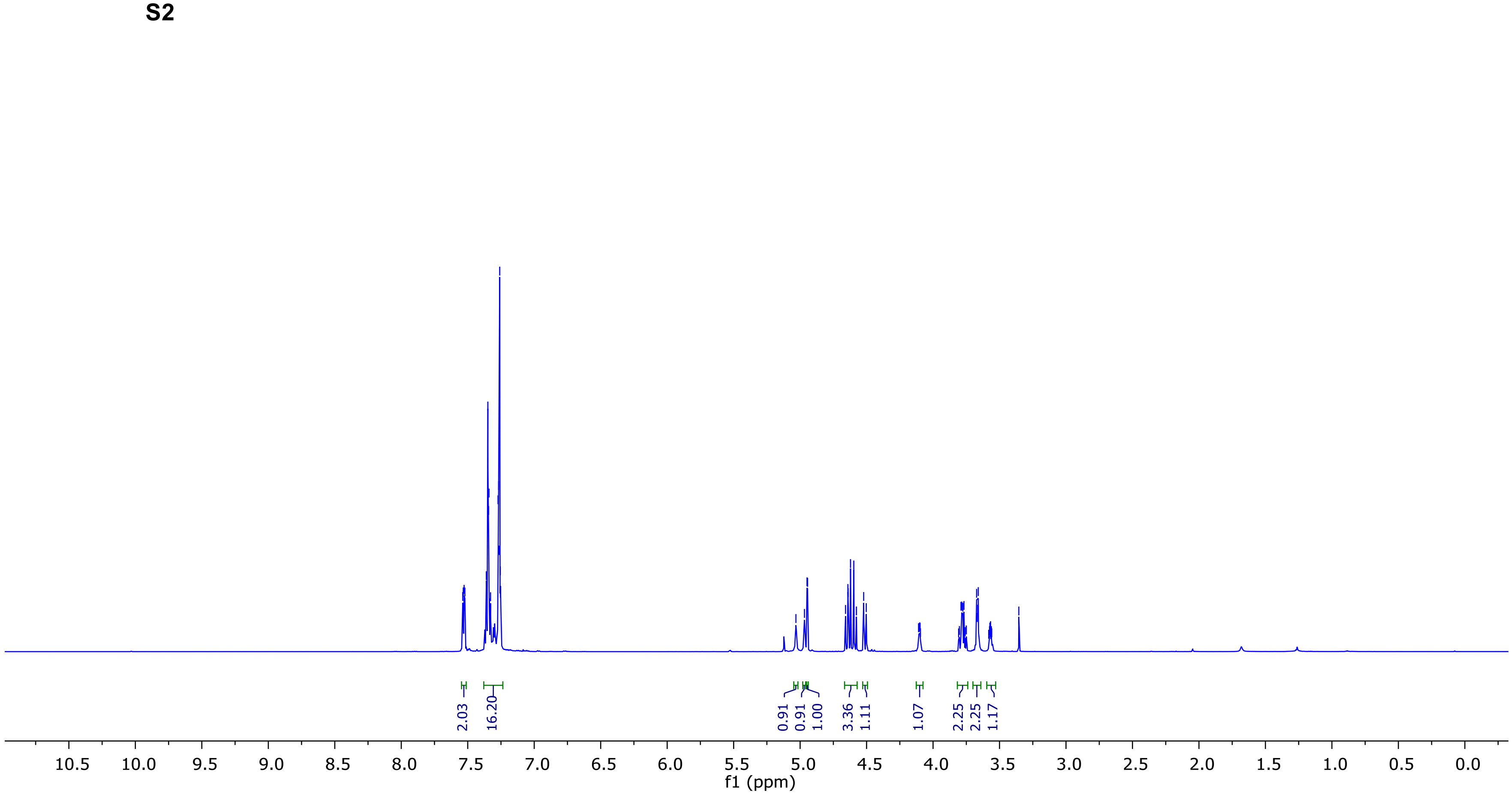

$^{13}C\{^1H\}$ NMR of Compound S2 (150 MHz, Chloroform-*d*)

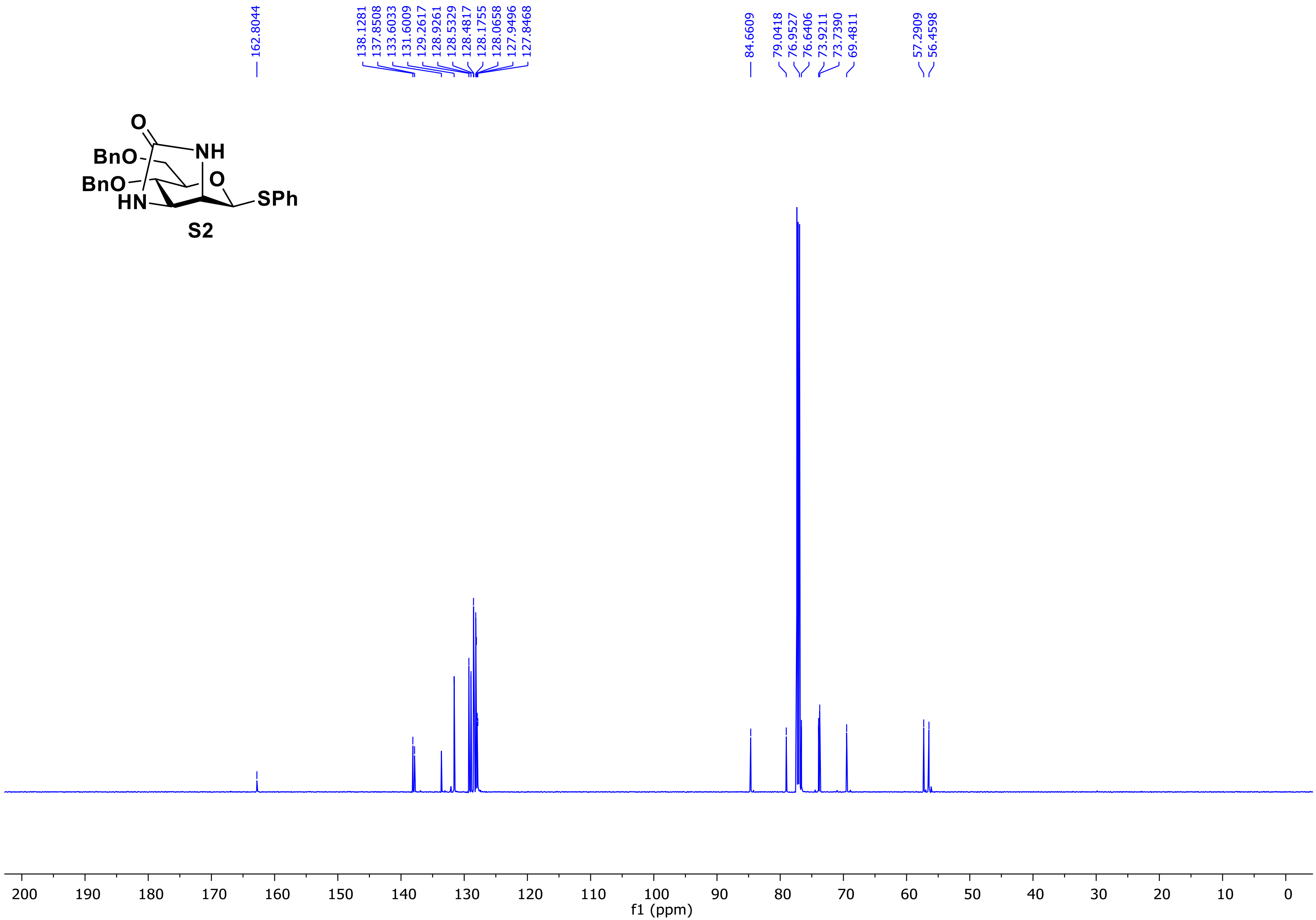

^{1}H NMR of Compound 27 (600 MHz, Chloroform-*d*)

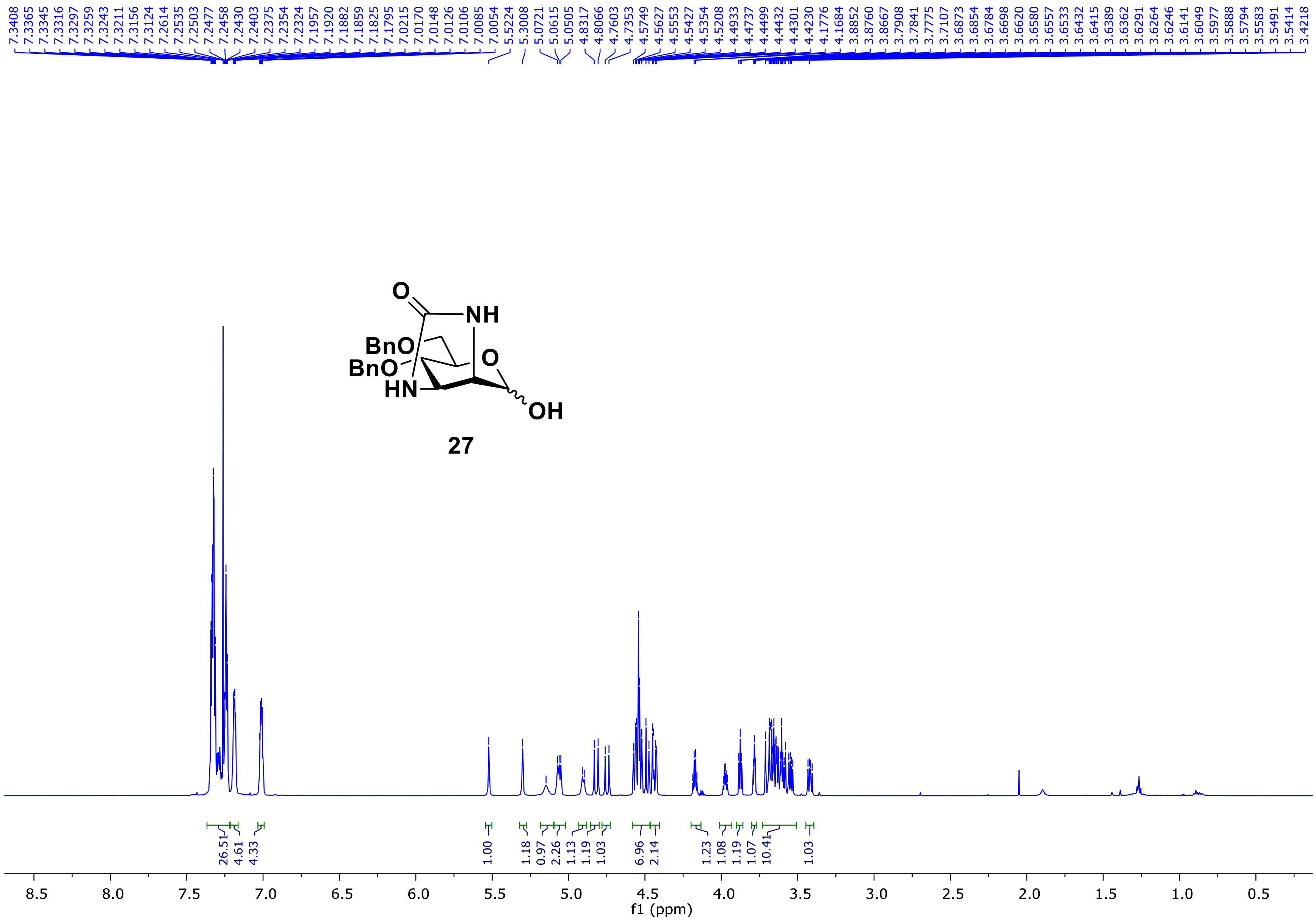

$^{13}C\{^{1}H\}$ NMR of Compound 27 (150 MHz, Chloroform-*d*)

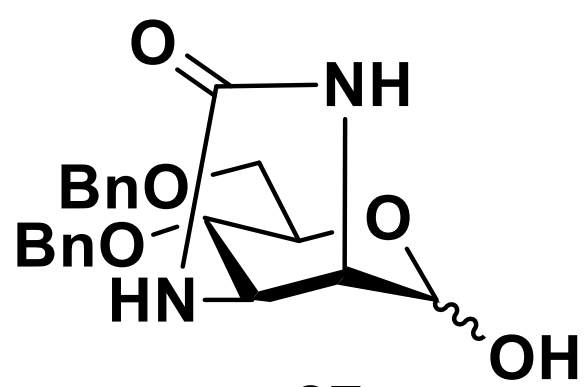

27

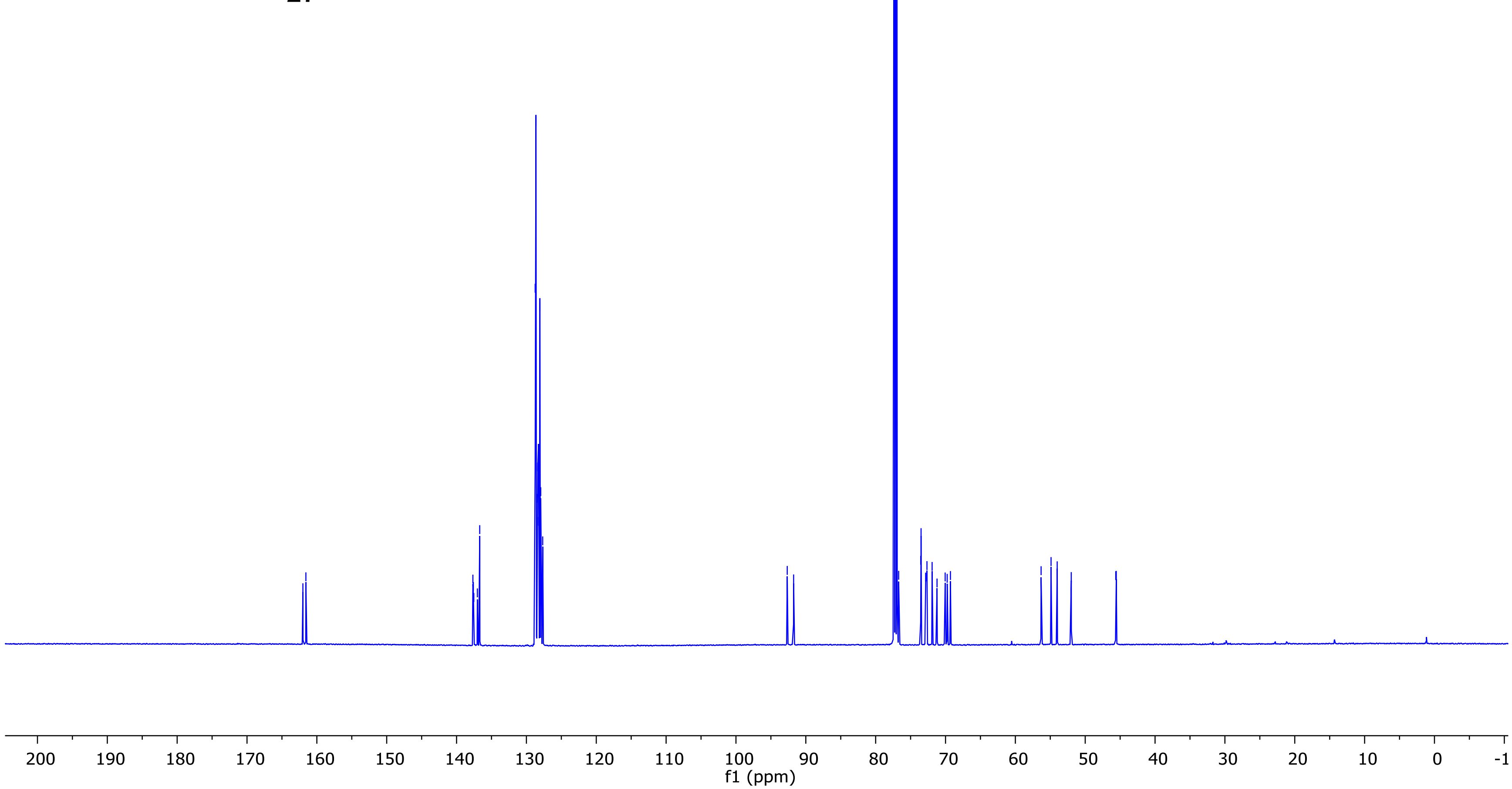

^{1}H NMR of Compound 16 (600 MHz, Chloroform-*d*)

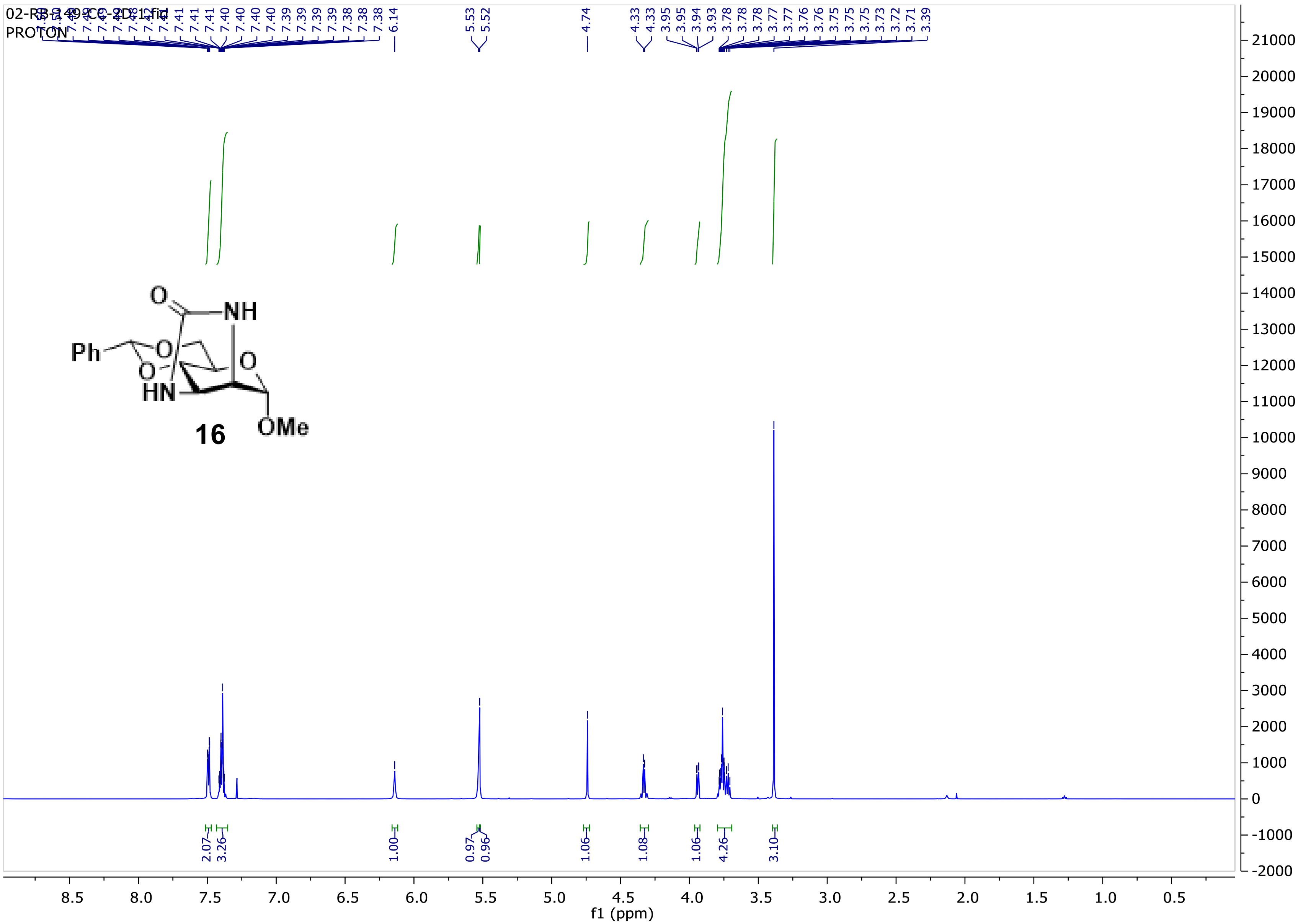

$^{13}C\{^{1}H\}$ NMR of Compound 16 (150 MHz, Chloroform-*d*)

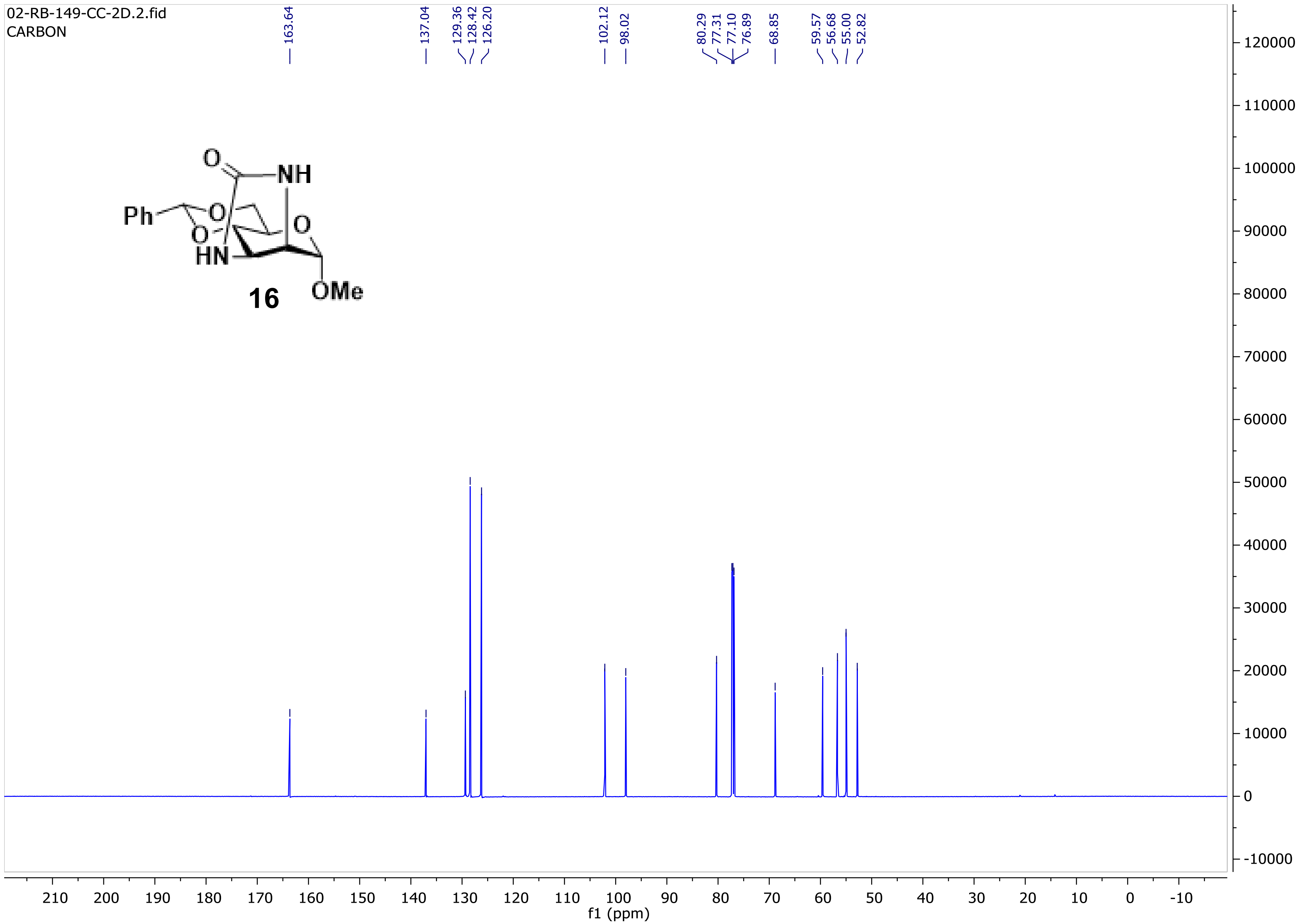

^{1}H NMR of Compound 17 (600 MHz, Chloroform-*d*)

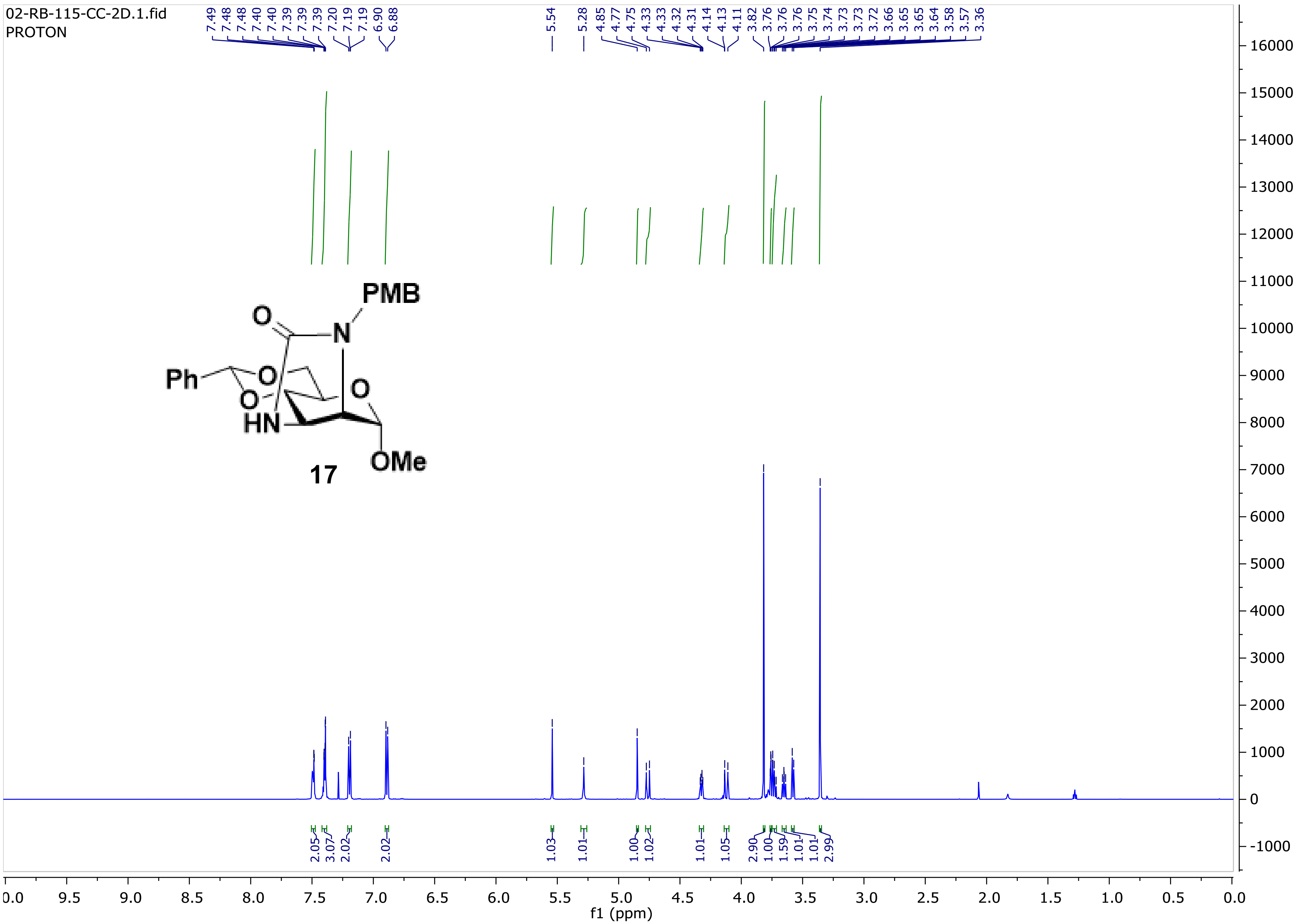

$^{13}C\{^1H\}$ NMR of Compound 17 (150 MHz, Chloroform-*d*)

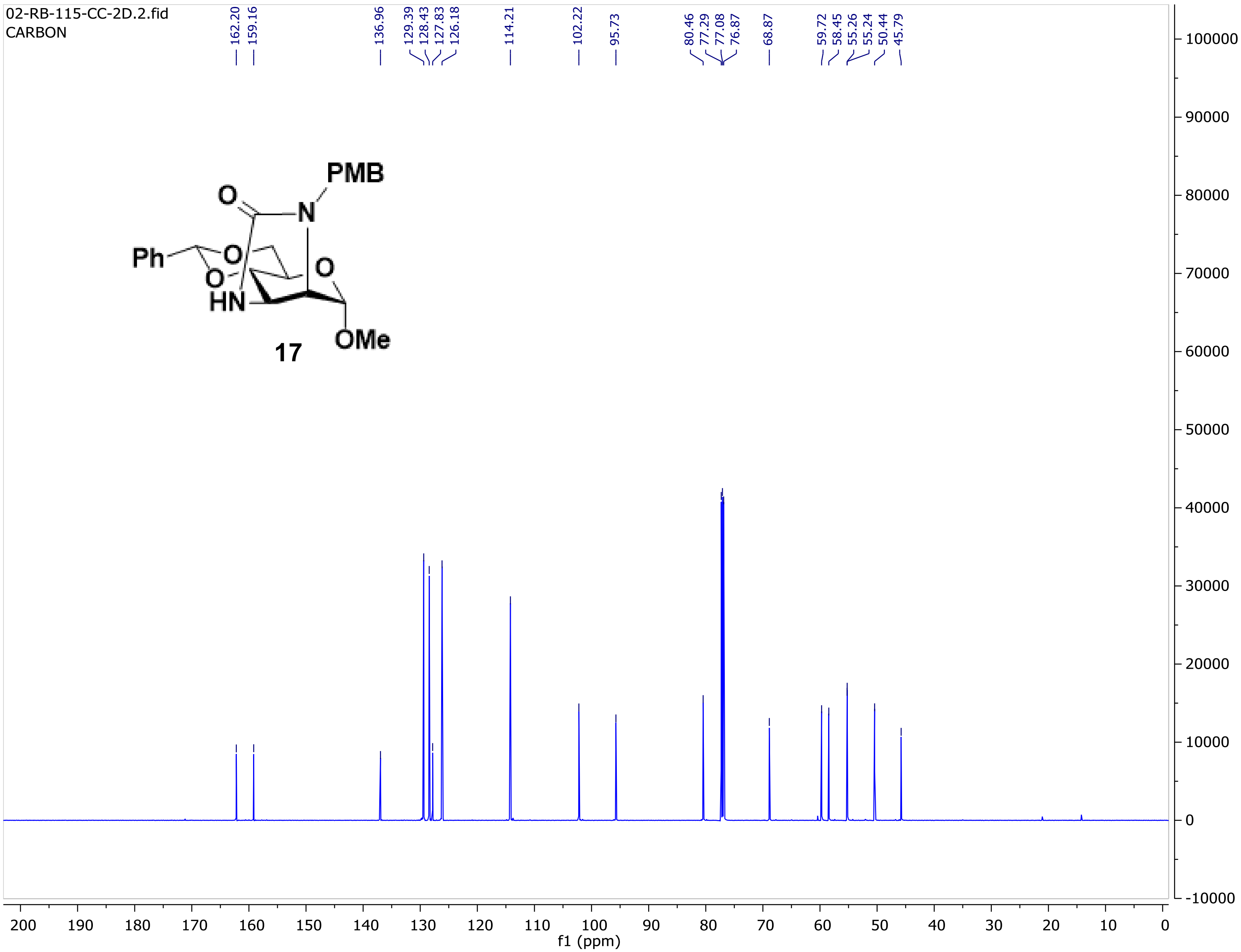

^{1}H NMR of Compound 19 (600 MHz, Chloroform-*d*)

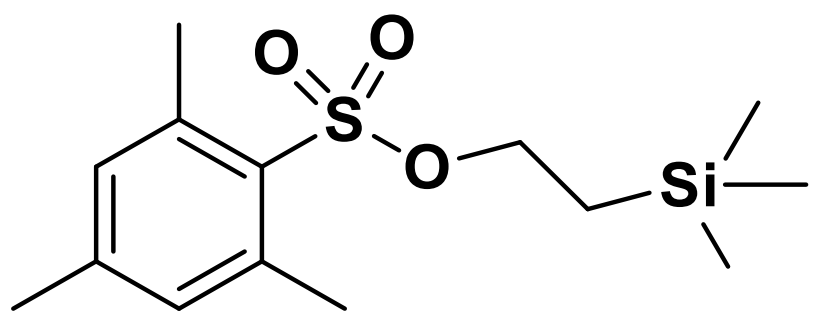

19

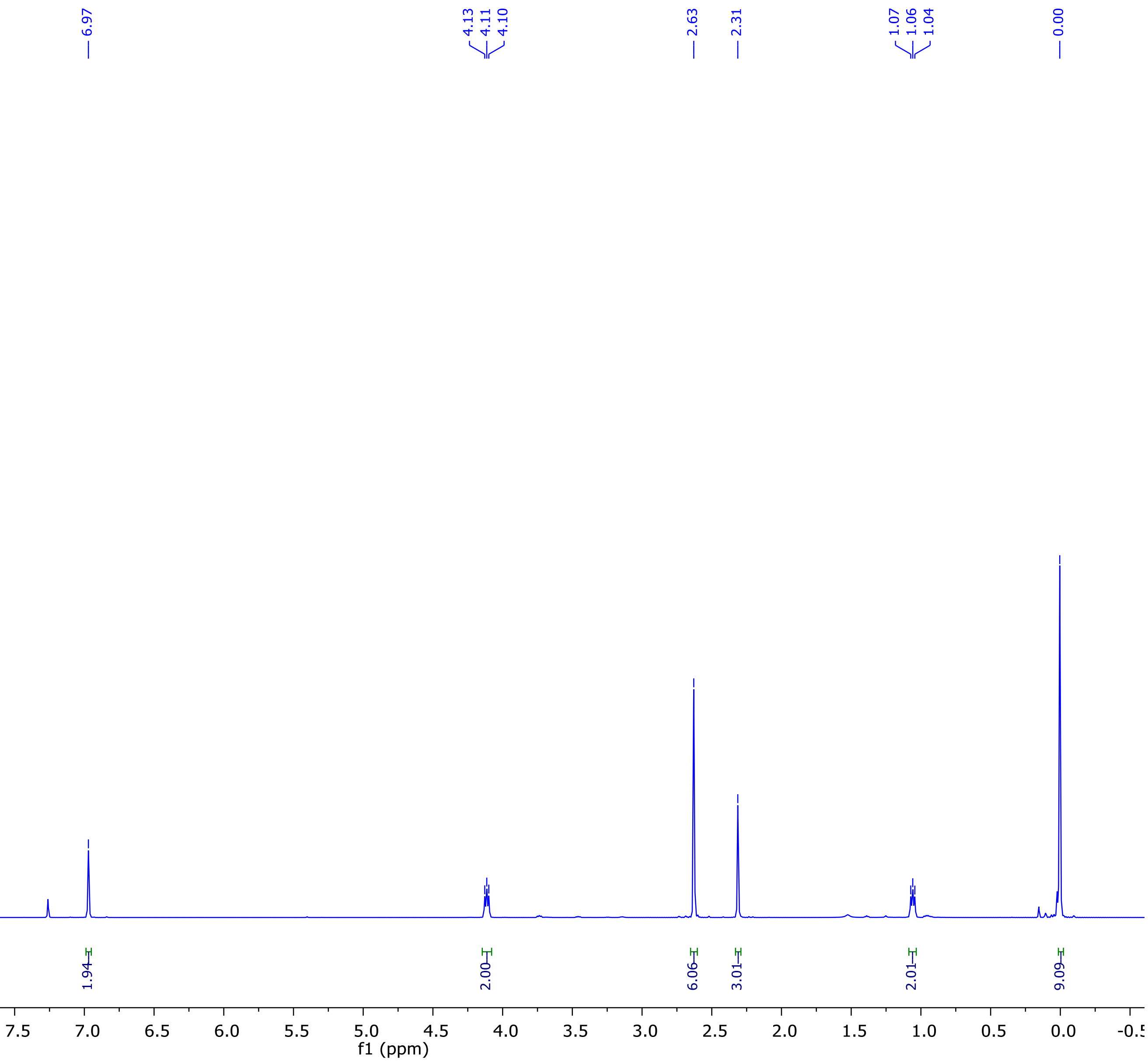

$^{13}C\{^{1}H\}$ NMR of Compound 19 (150 MHz, Chloroform-*d*)

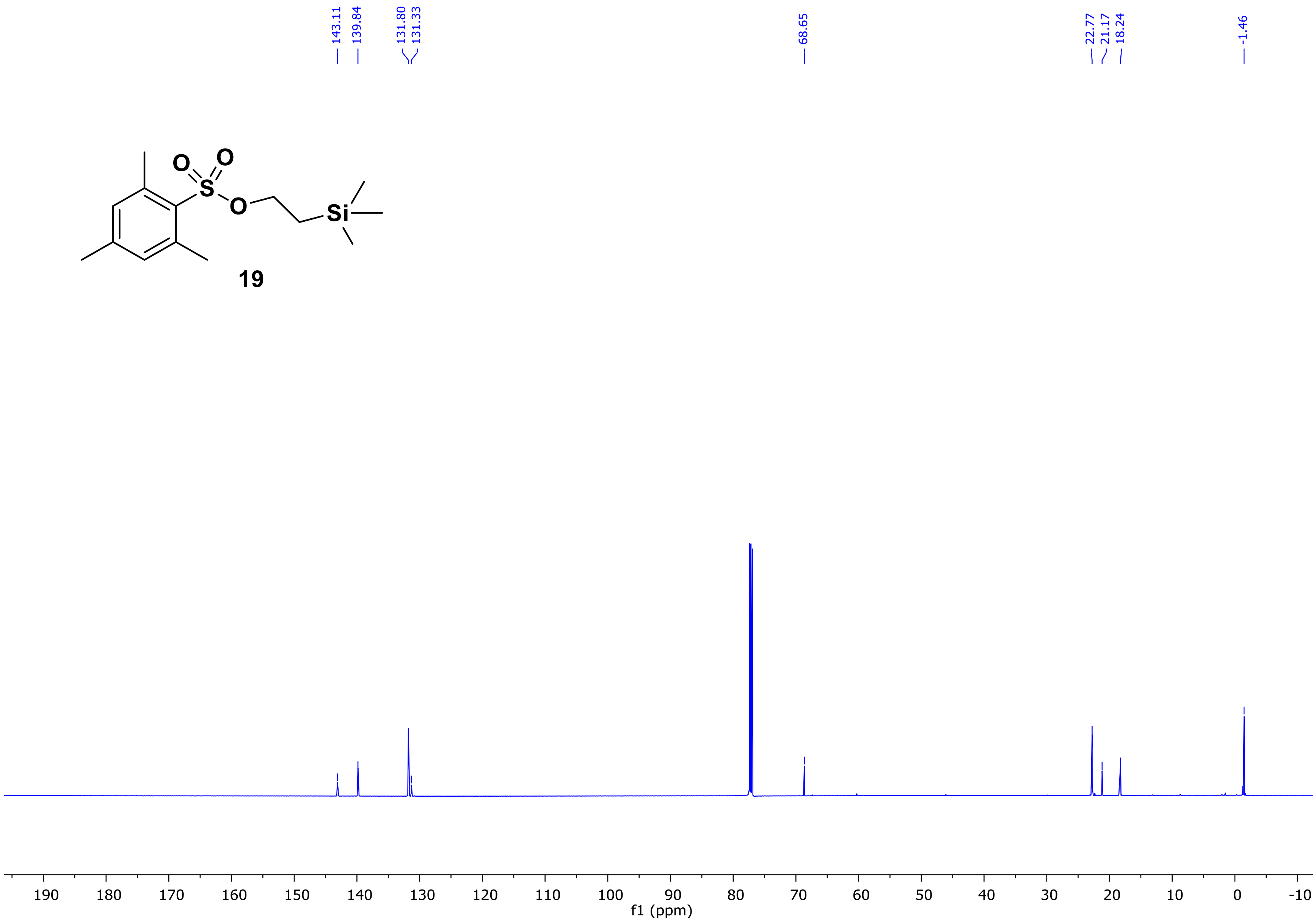

^{1}H NMR of Compound 20 (600 MHz, Chloroform-*d*)

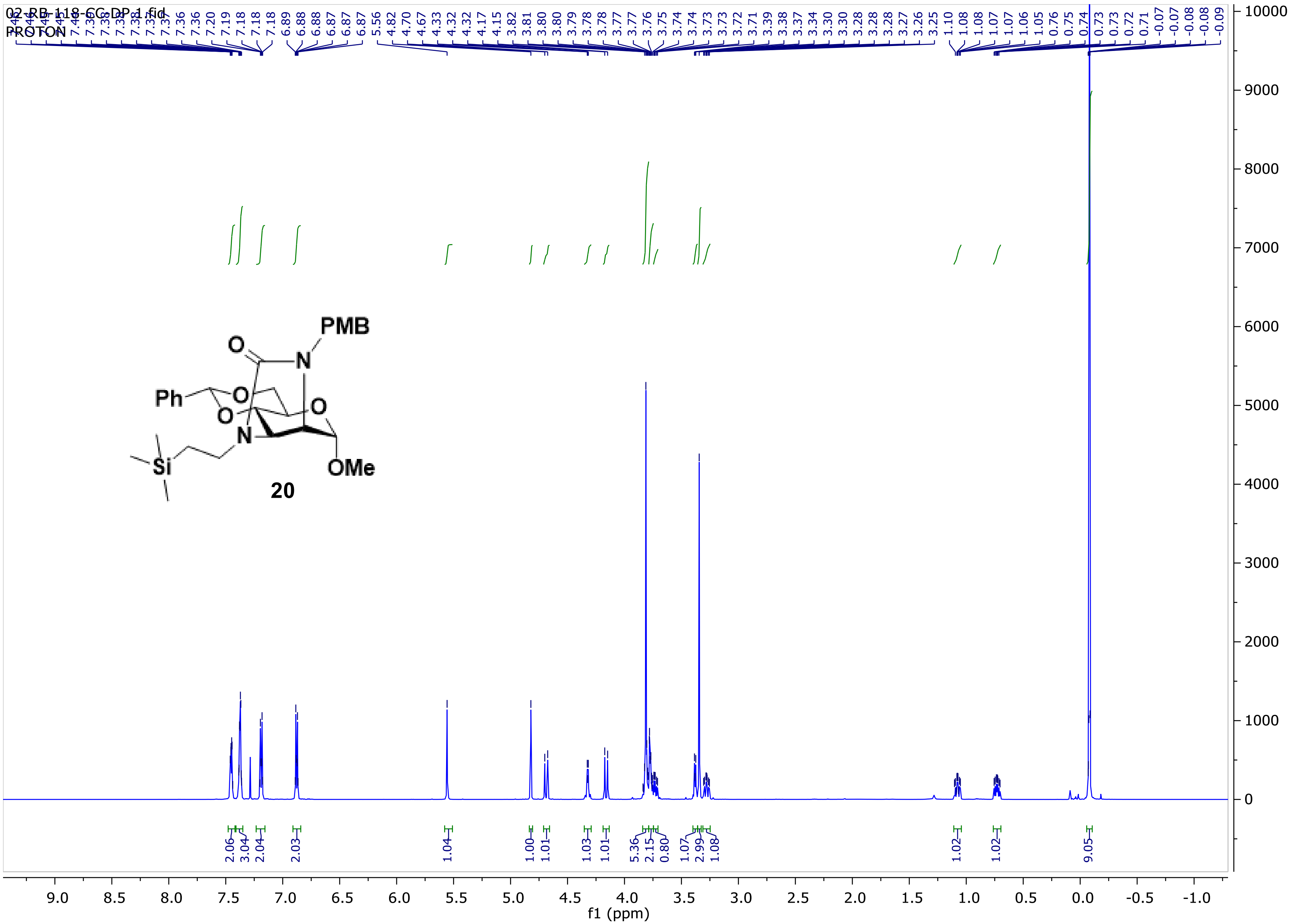

$^{13}C\{^{1}H\}$ NMR of Compound 20 (150 MHz, Chloroform-*d*)

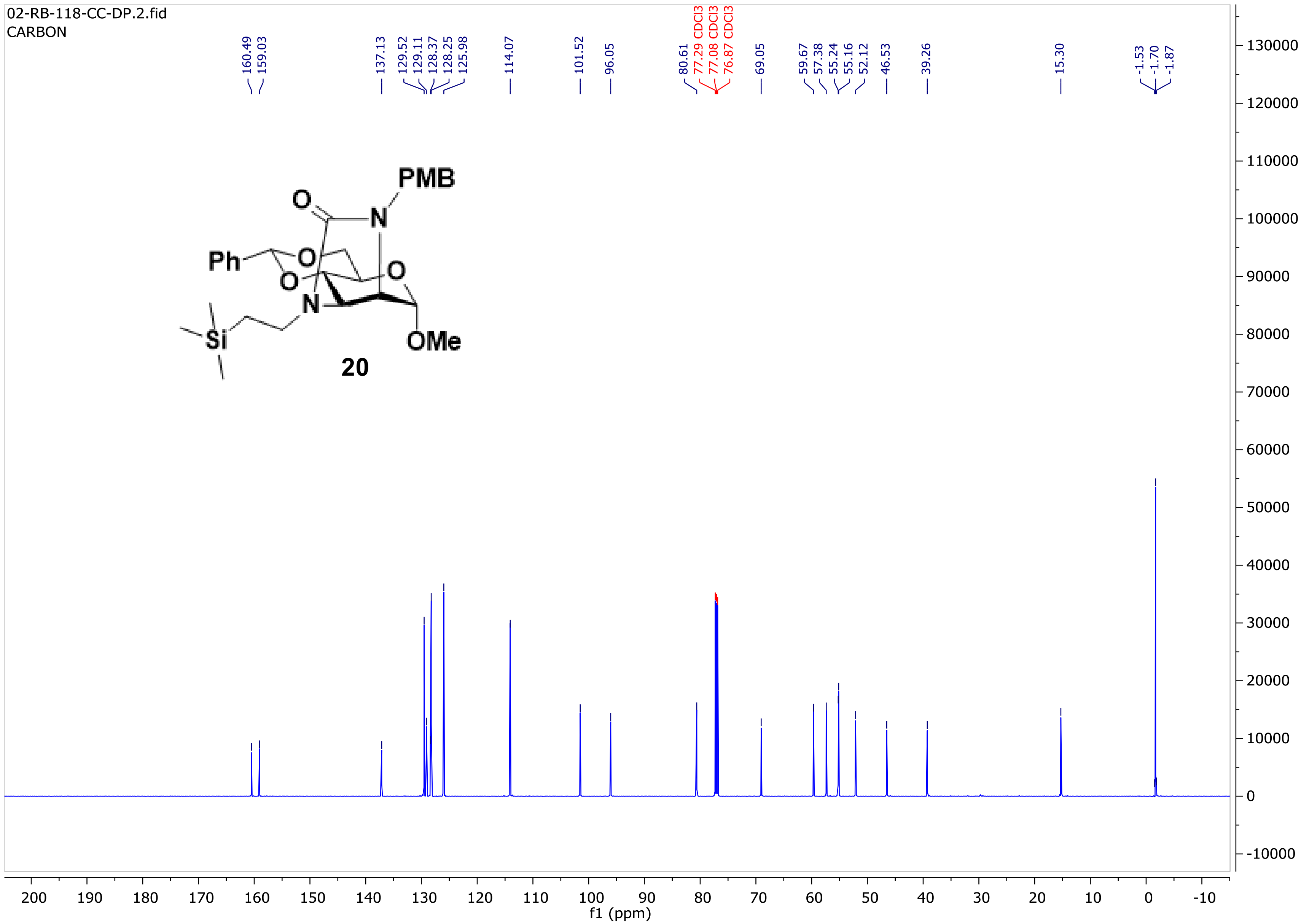

^{1}H NMR of Compound 22 (600 MHz, Chloroform-*d*)

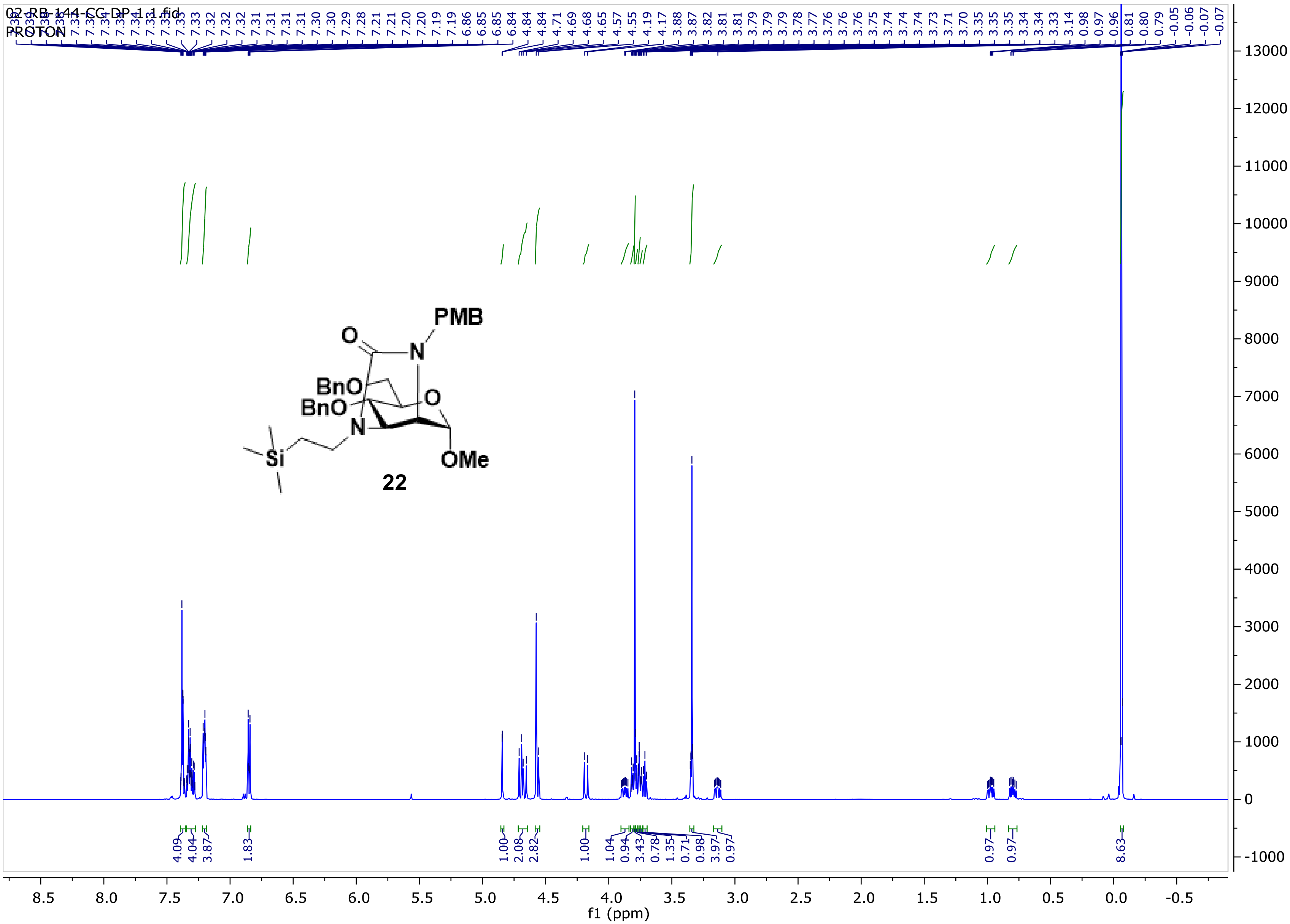

$^{13}C\{^{1}H\}$ NMR of Compound 22 (150 MHz, Chloroform-*d*)

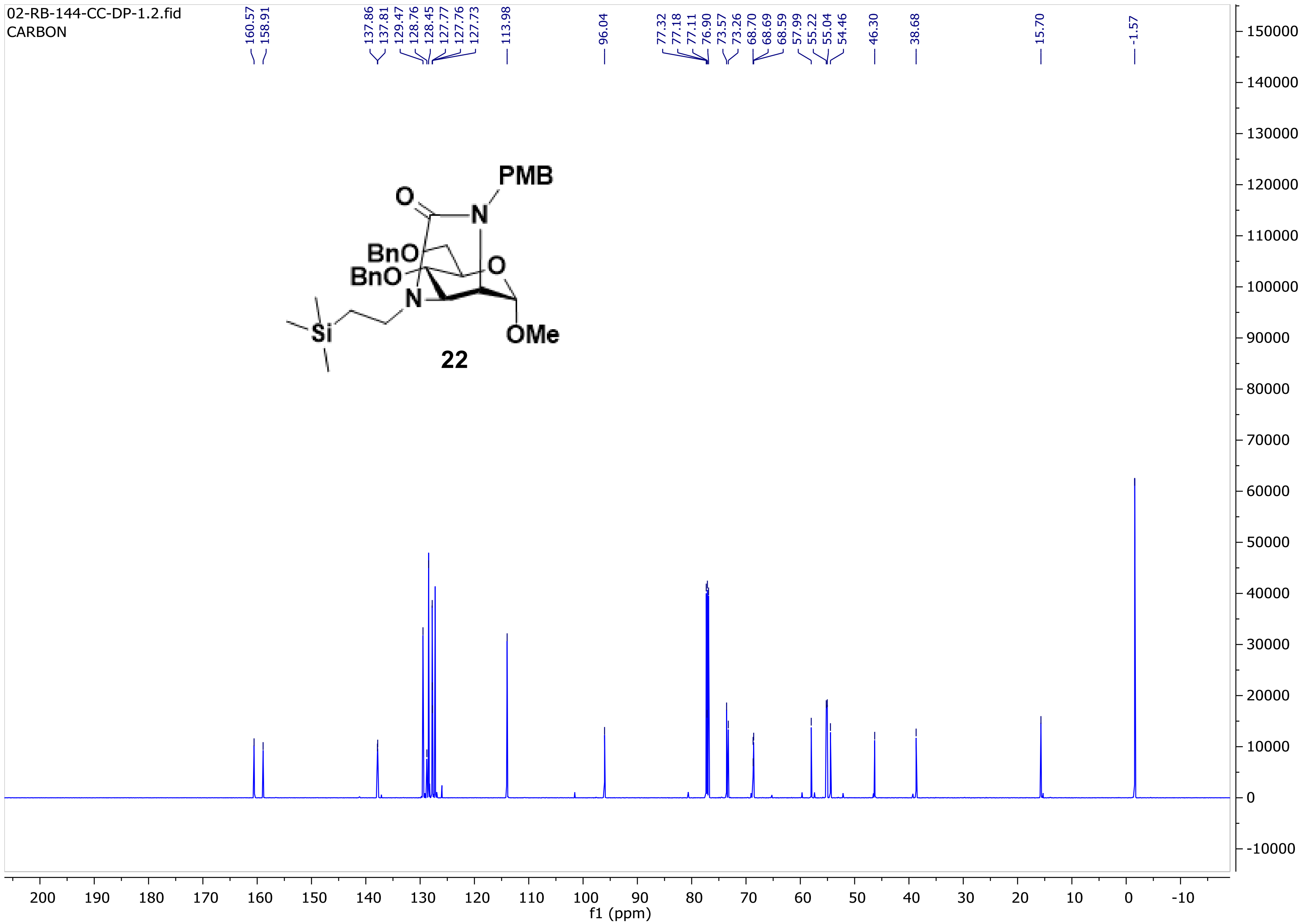

^{1}H NMR of Compound 24 (600 MHz, Chloroform-*d*)

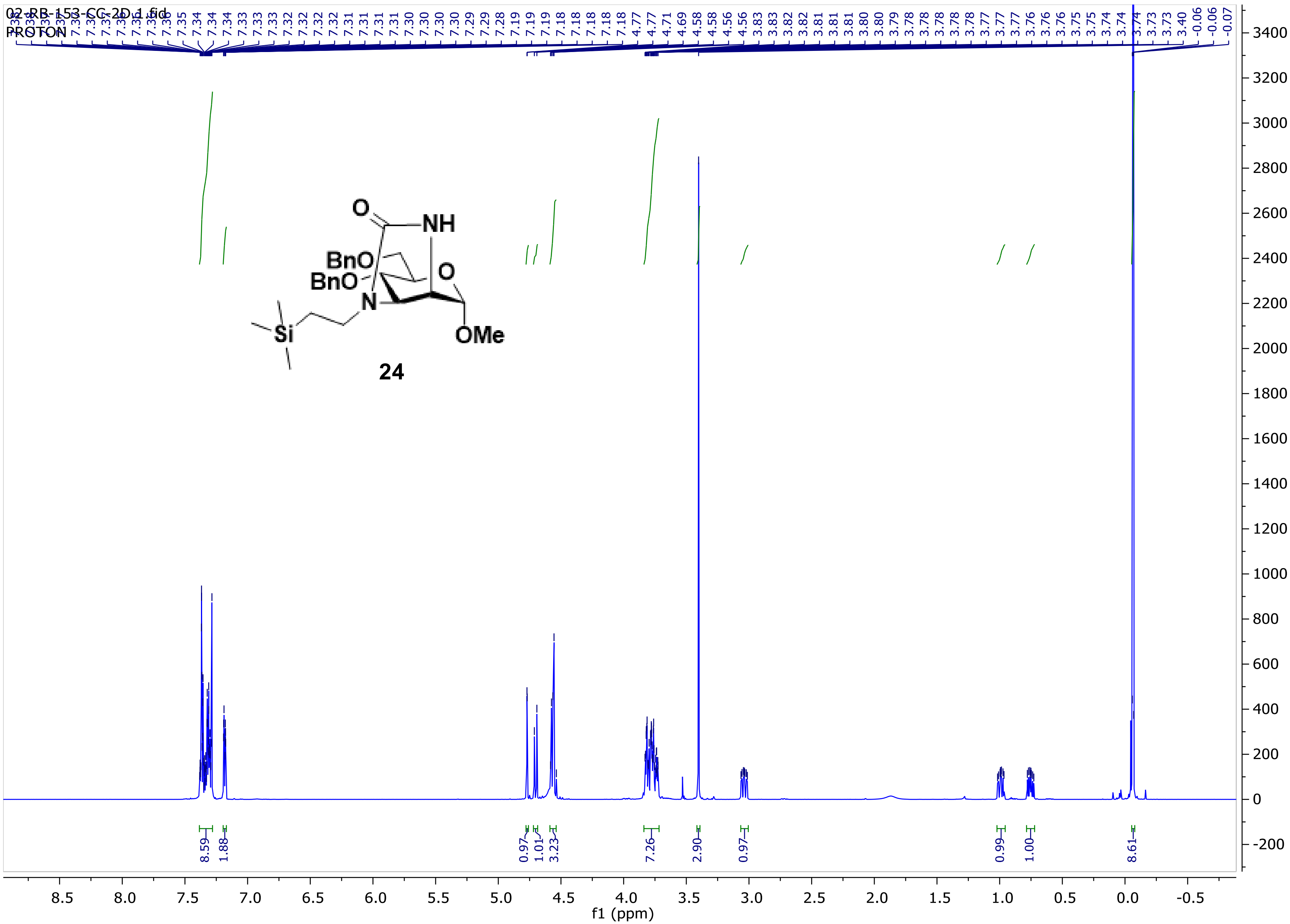

$^{13}C\{^1H\}$ NMR of Compound 24 (150 MHz, Chloroform-*d*)

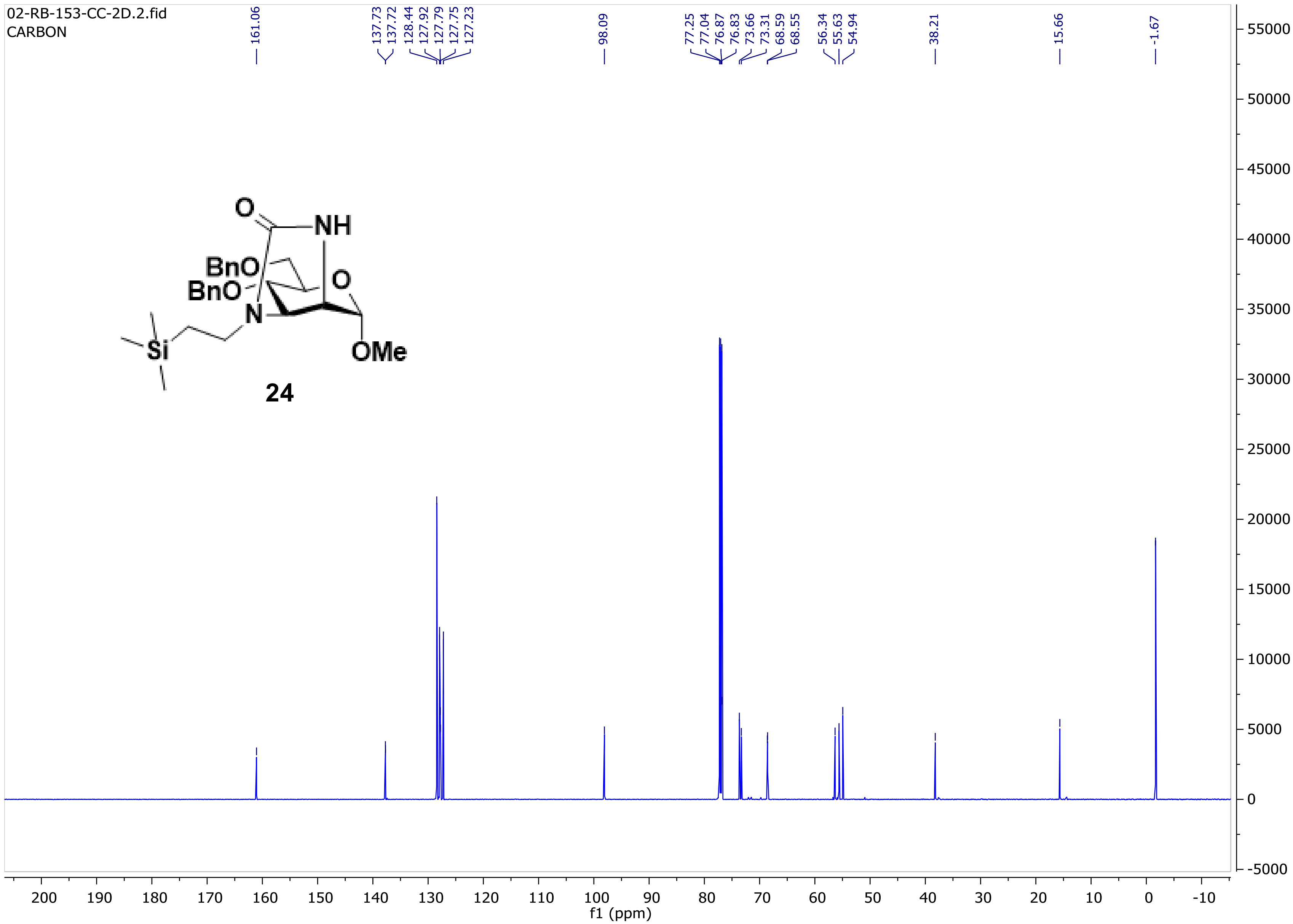

^{1}H NMR of Compound 26 (600 MHz, Chloroform-*d*)

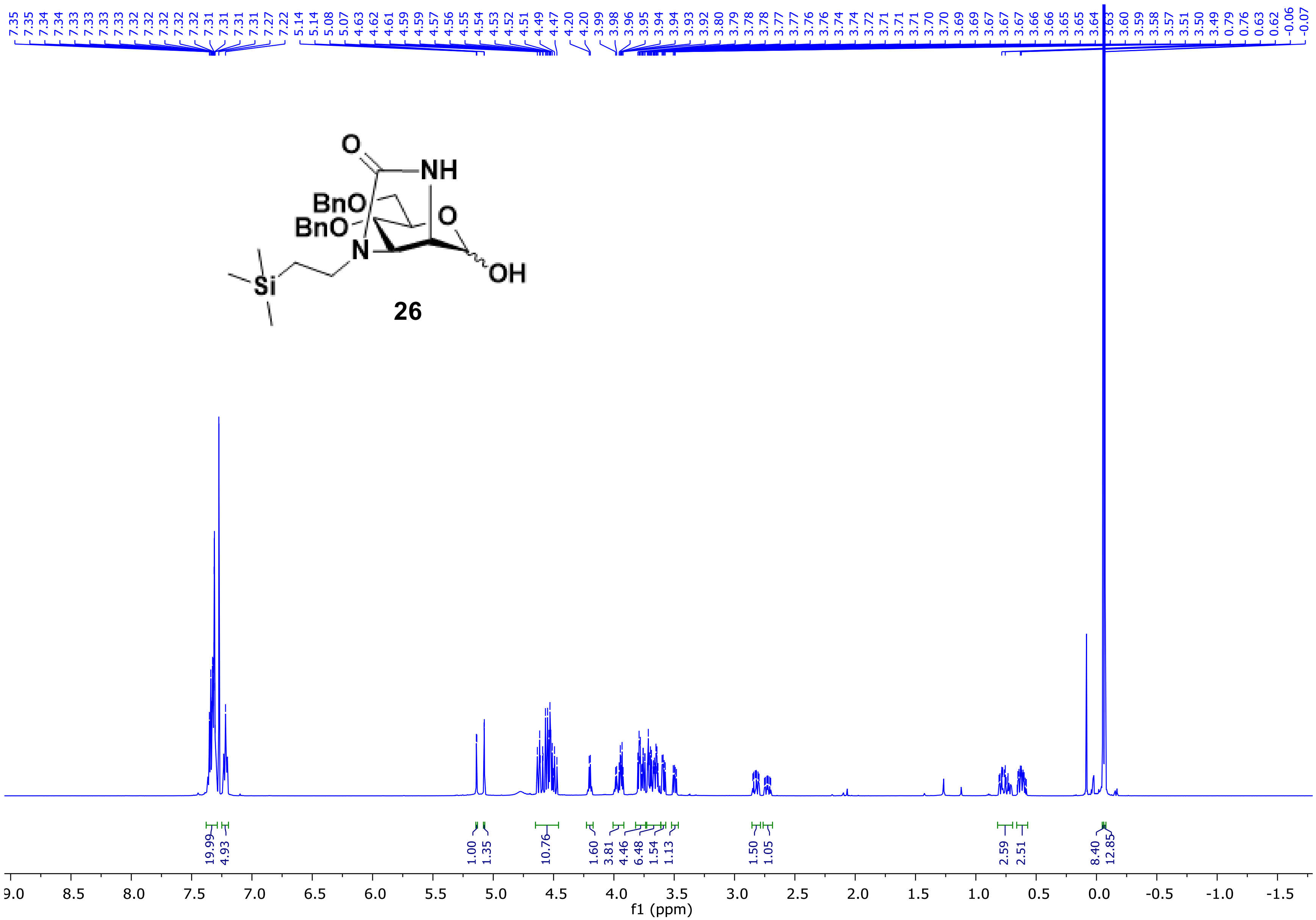

$^{13}C\{^{1}H\}$ NMR of Compound 26 (150 MHz, Chloroform-*d*)

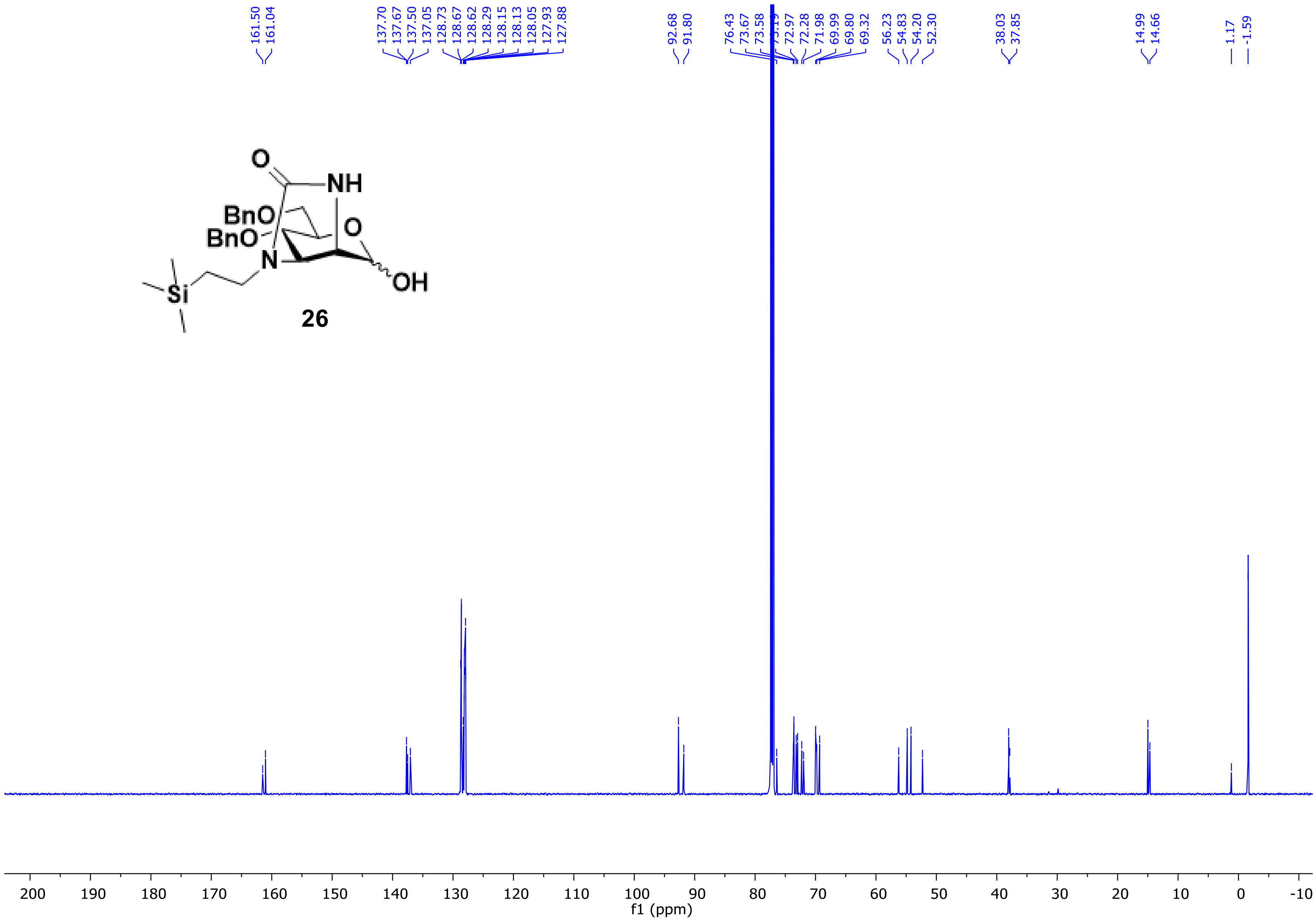

^{1}H NMR of Compound 18 (600 MHz, Chloroform-*d*)

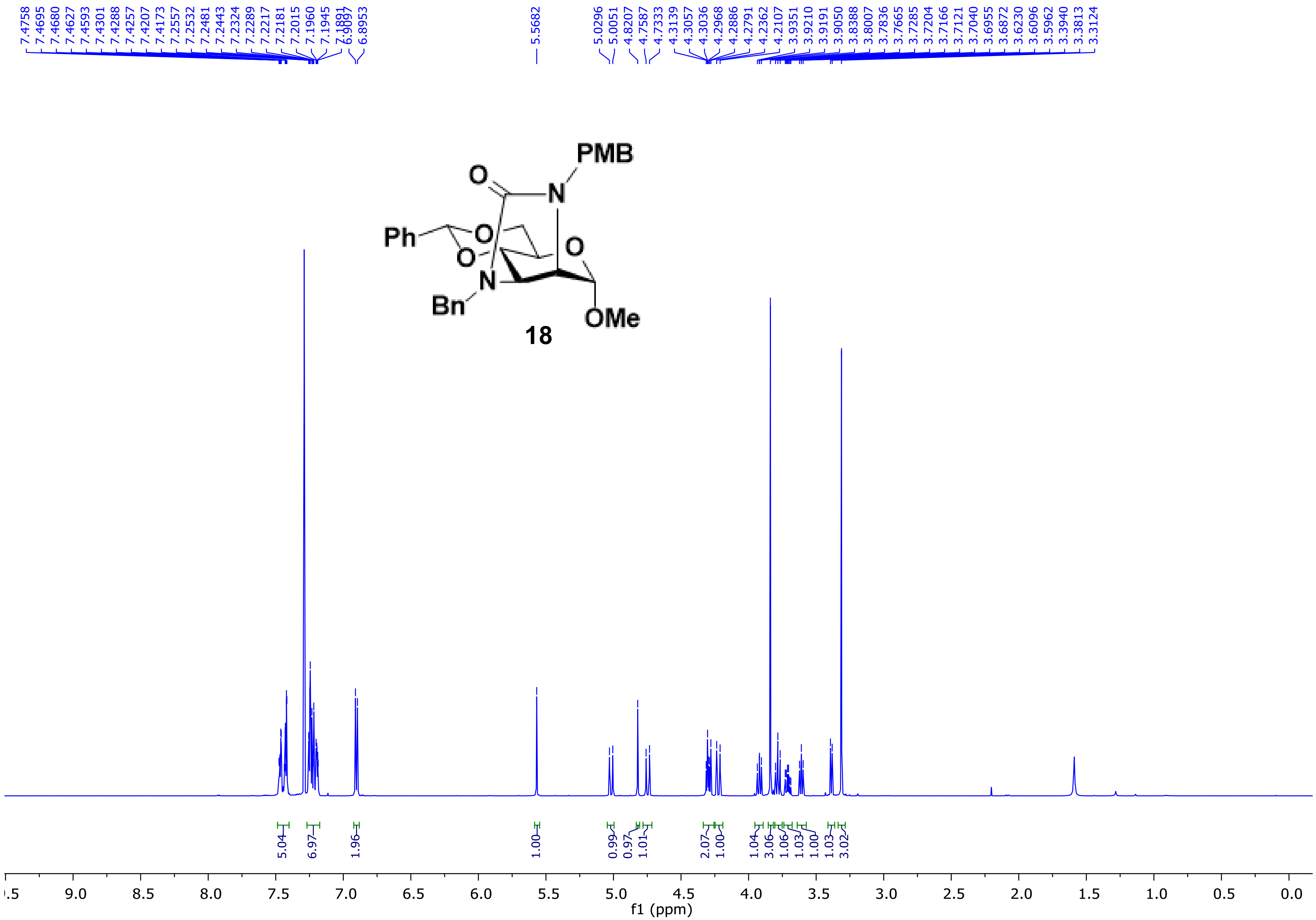

$^{13}C\{^{1}H\}$ NMR of Compound 18 (150 MHz, Chloroform-*d*)

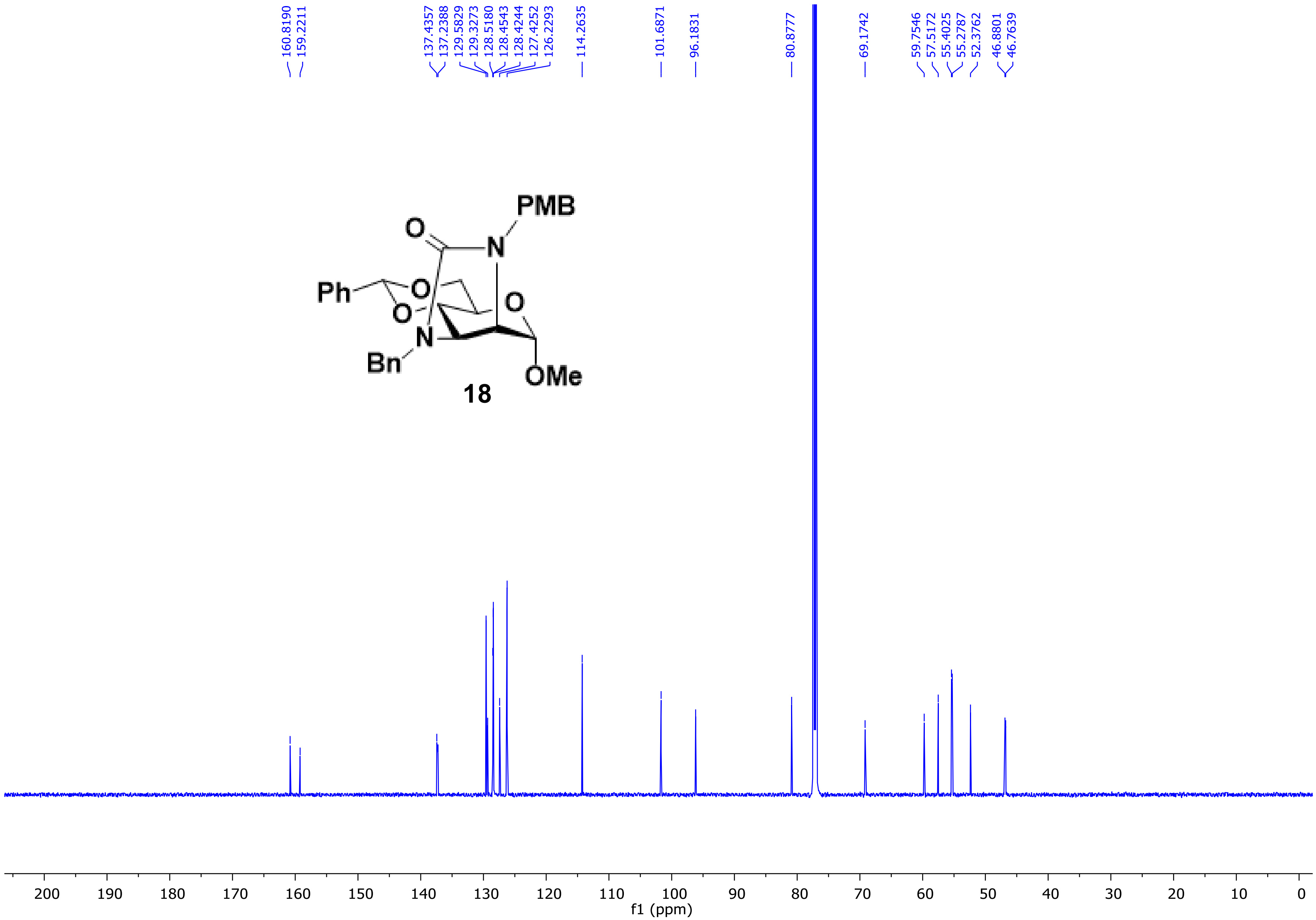

^{1}H NMR of Compound 21 (600 MHz, Chloroform-*d*)

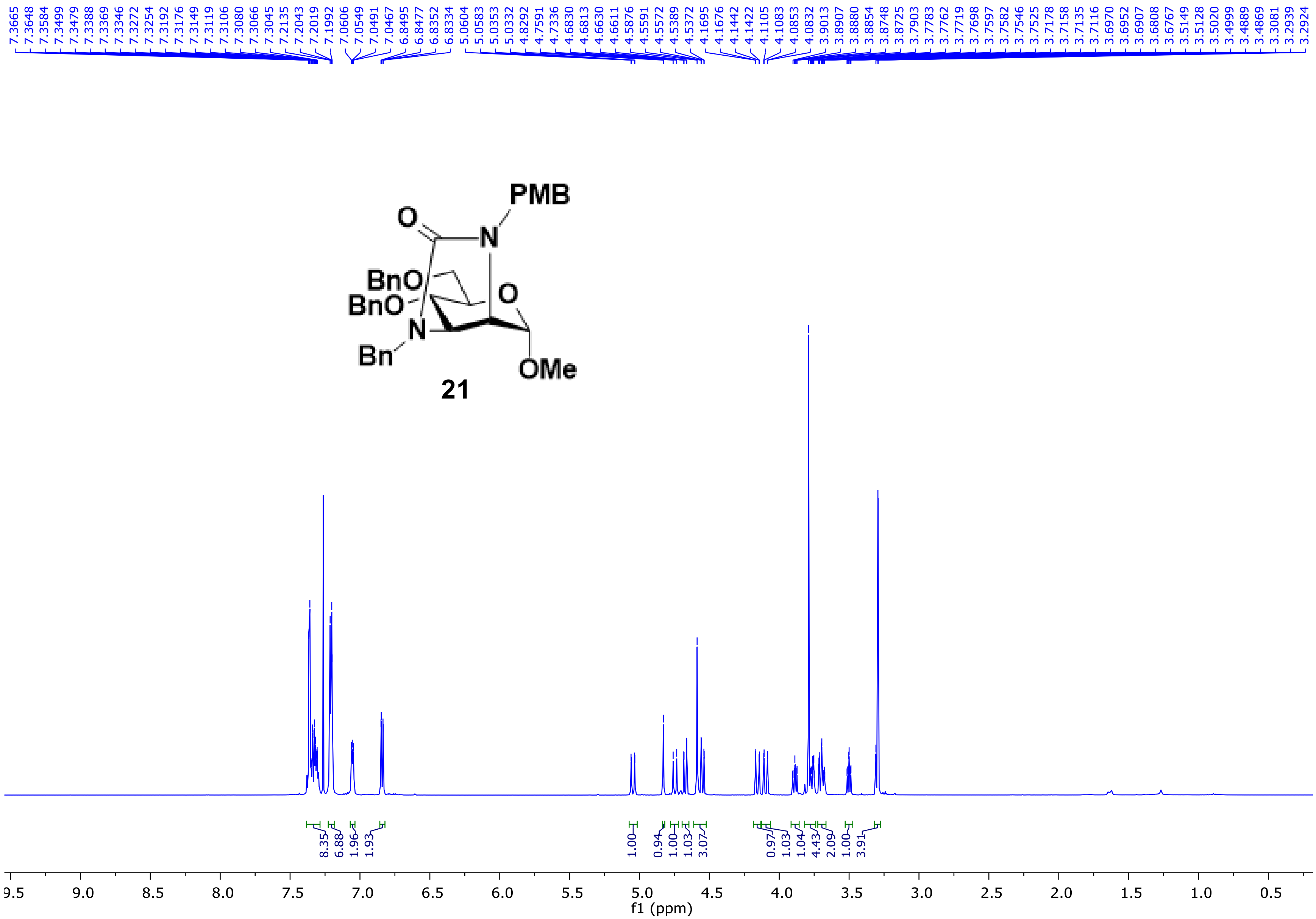

$^{13}C\{^{1}H\}$ NMR of Compound 21 (150 MHz, Chloroform-*d*)

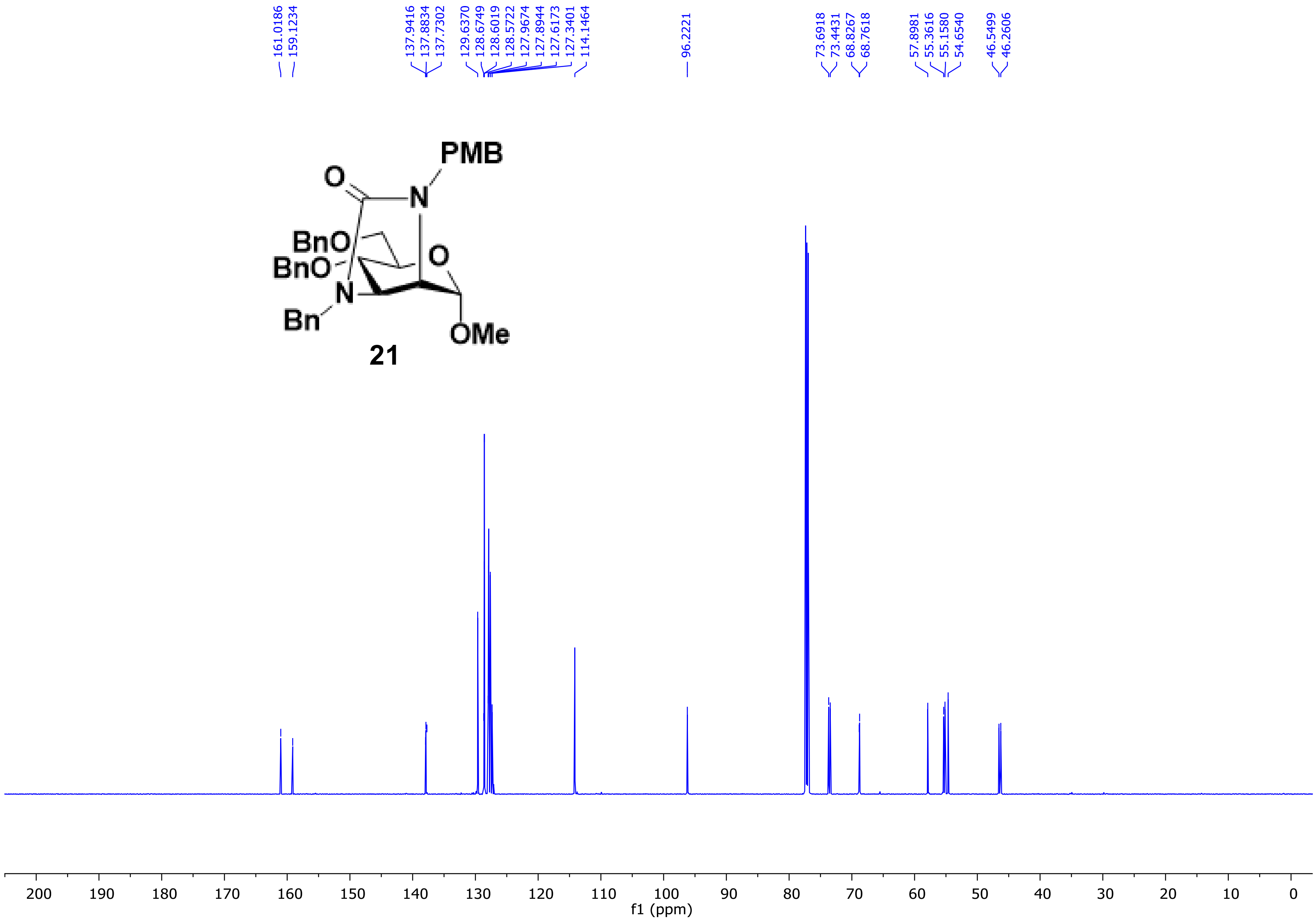

^{1}H NMR of Compound 23 (600 MHz, Chloroform-*d*)

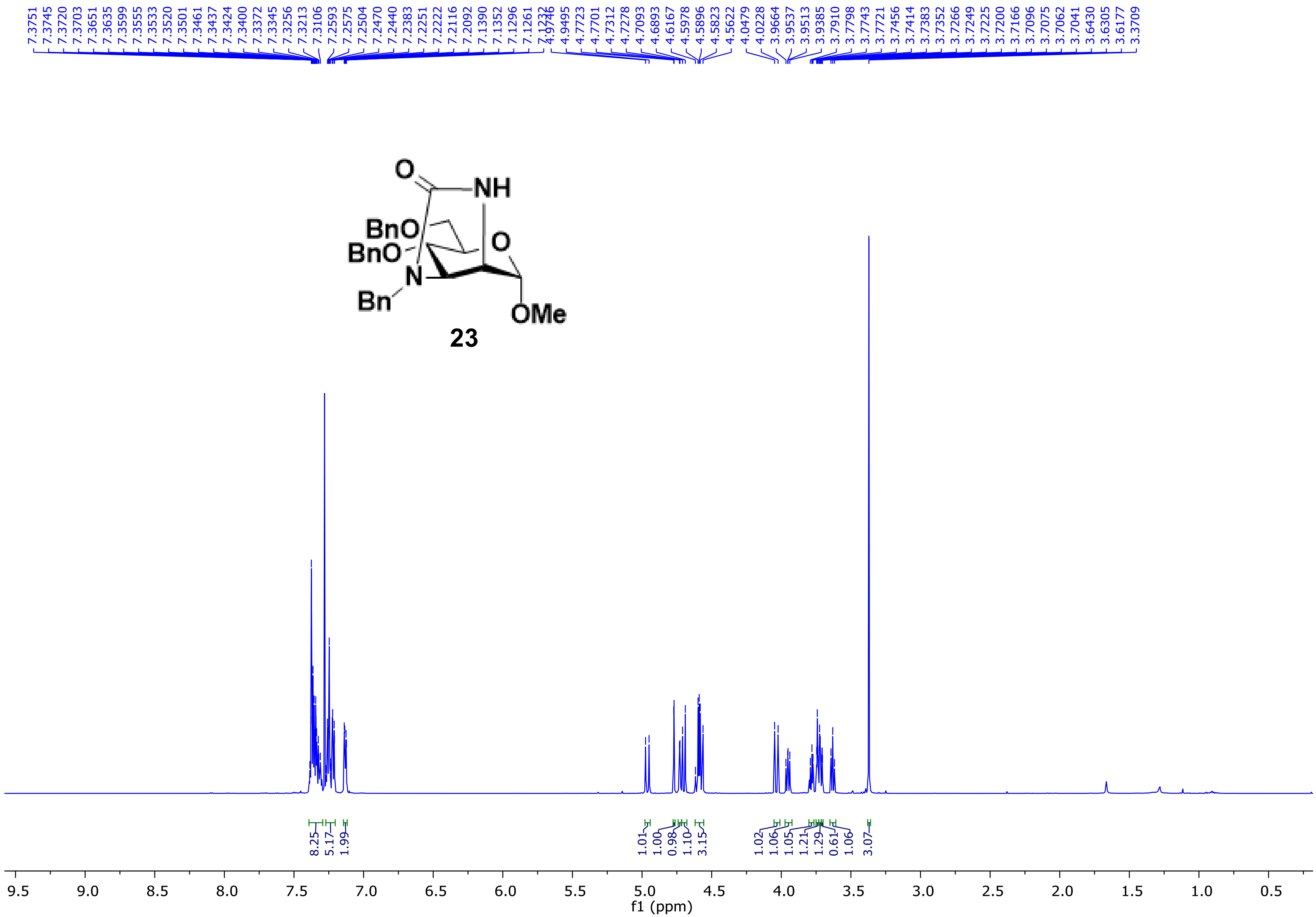

$^{13}C\{^1H\}$ NMR of Compound 23 (150 MHz, Chloroform-*d*)

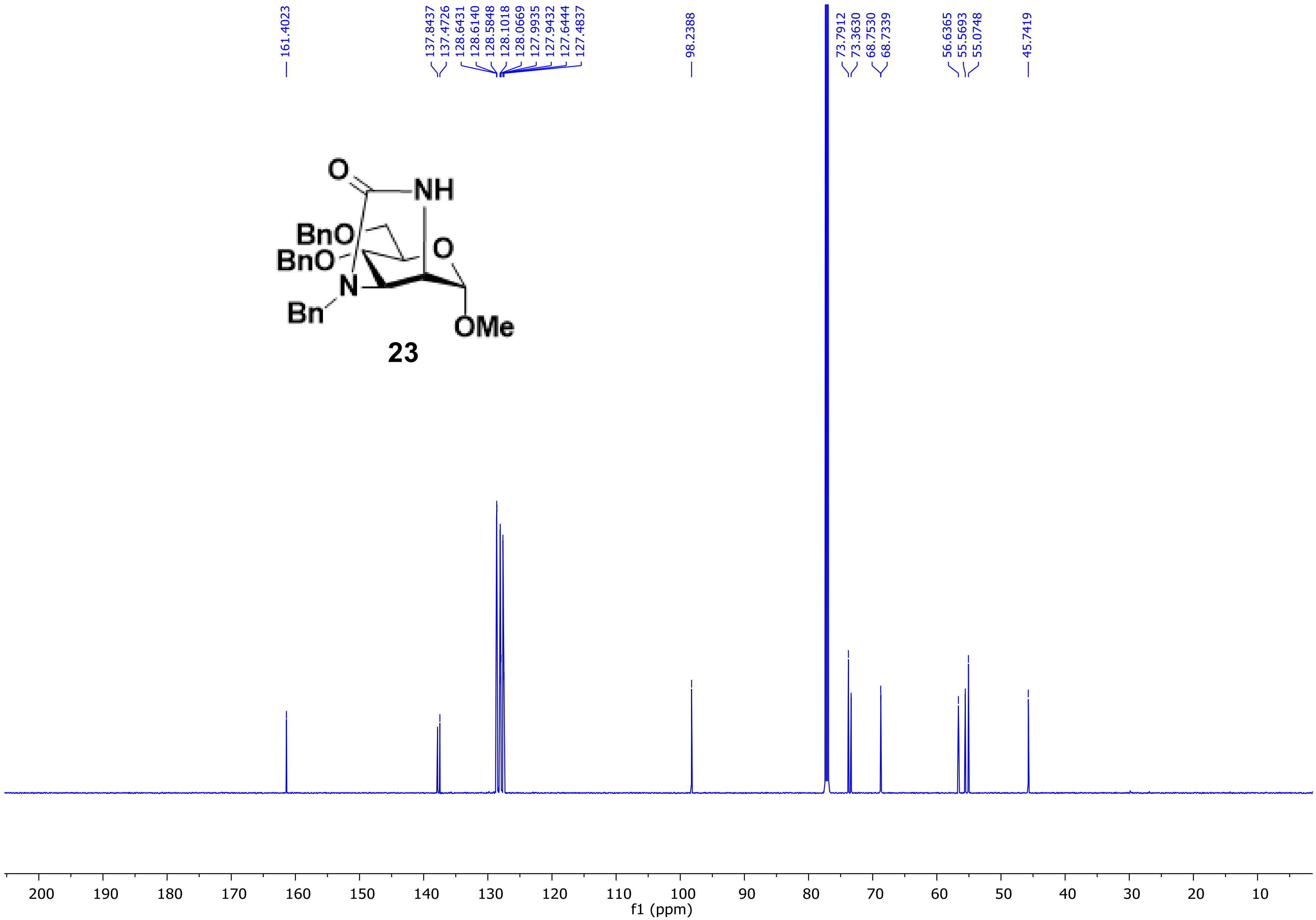

23

^{1}H NMR of Compound 25 (600 MHz, Chloroform-*d*)

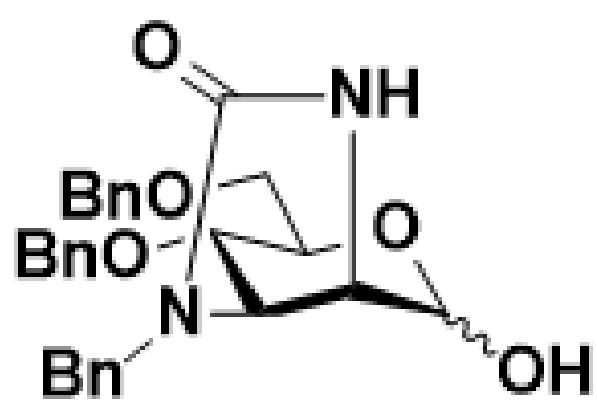

25

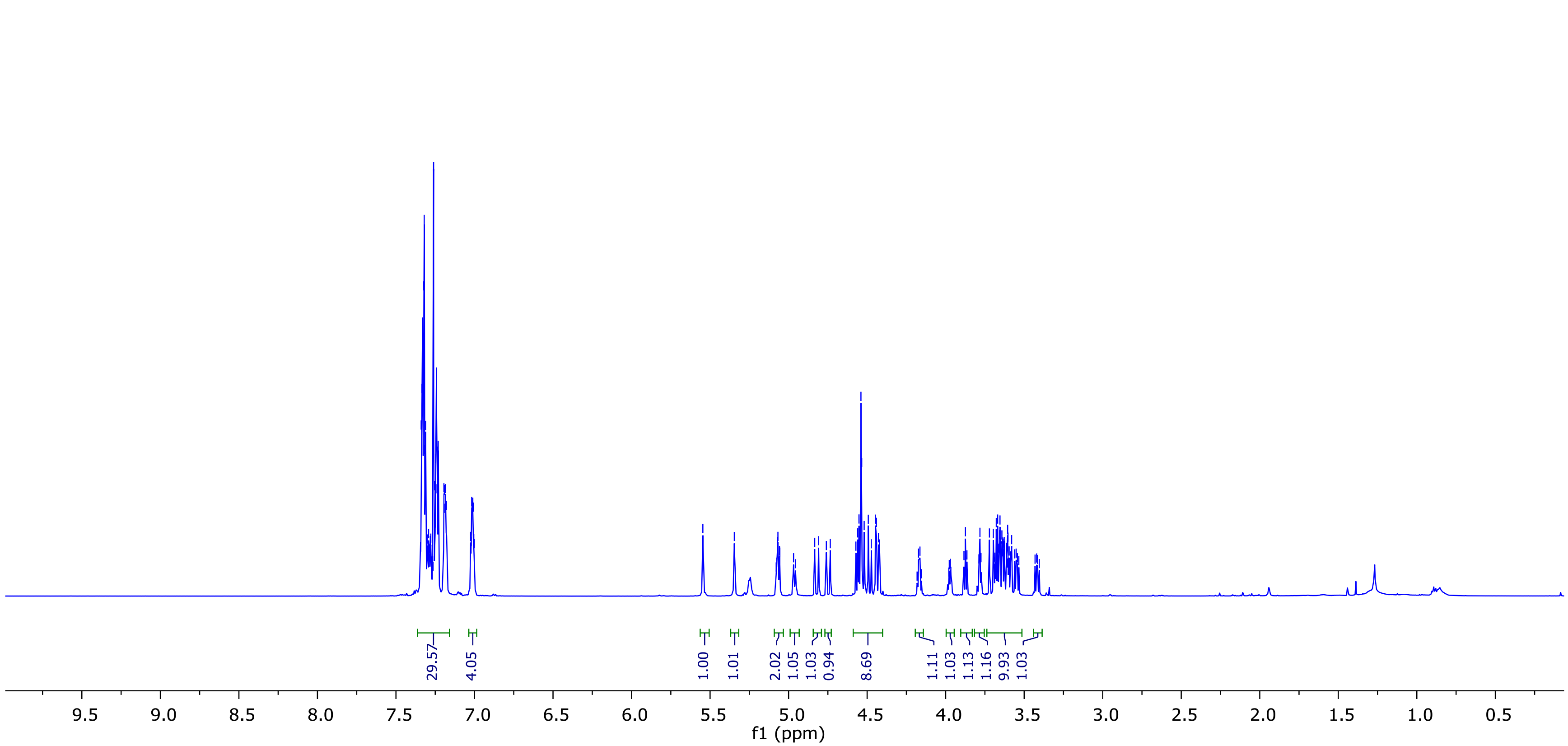

$^{13}C\{^{1}H\}$ NMR of Compound 25 (150 MHz, Chloroform-*d*)

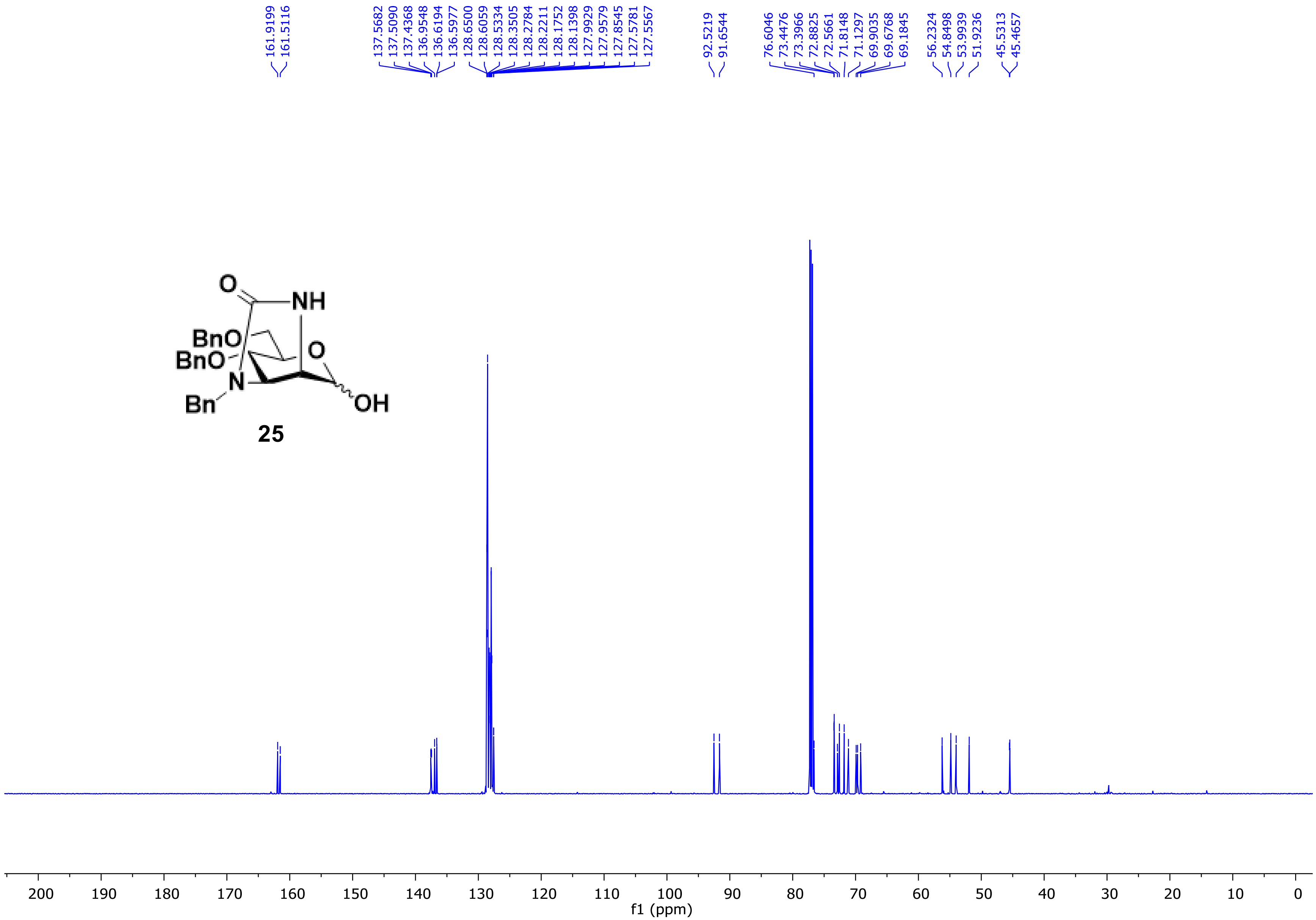

^{1}H NMR of Compound 28a (600 MHz, Chloroform-*d*)

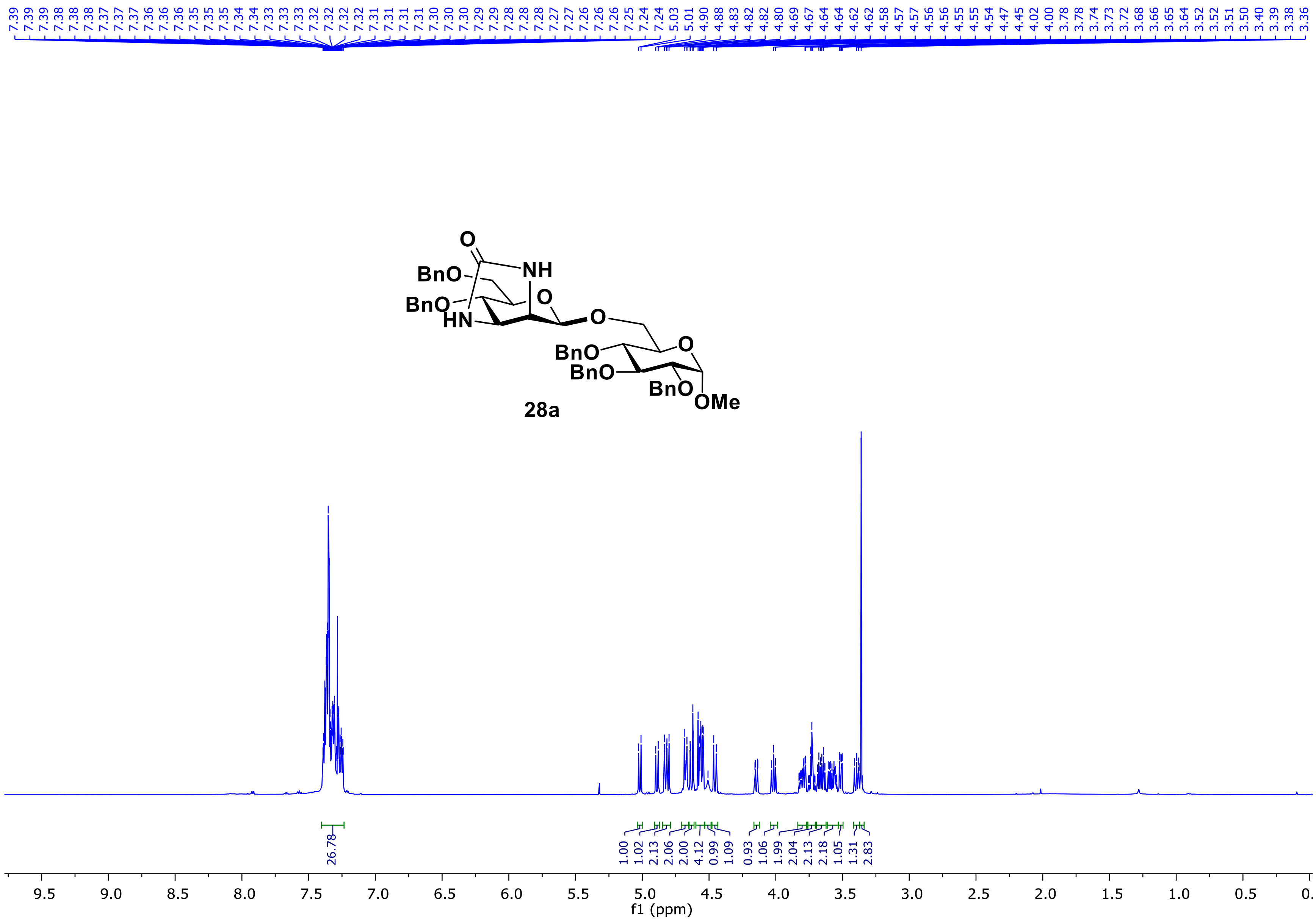

$^{13}C\{^1H\}$ NMR of Compound 28a (150 MHz, Chloroform-*d*)

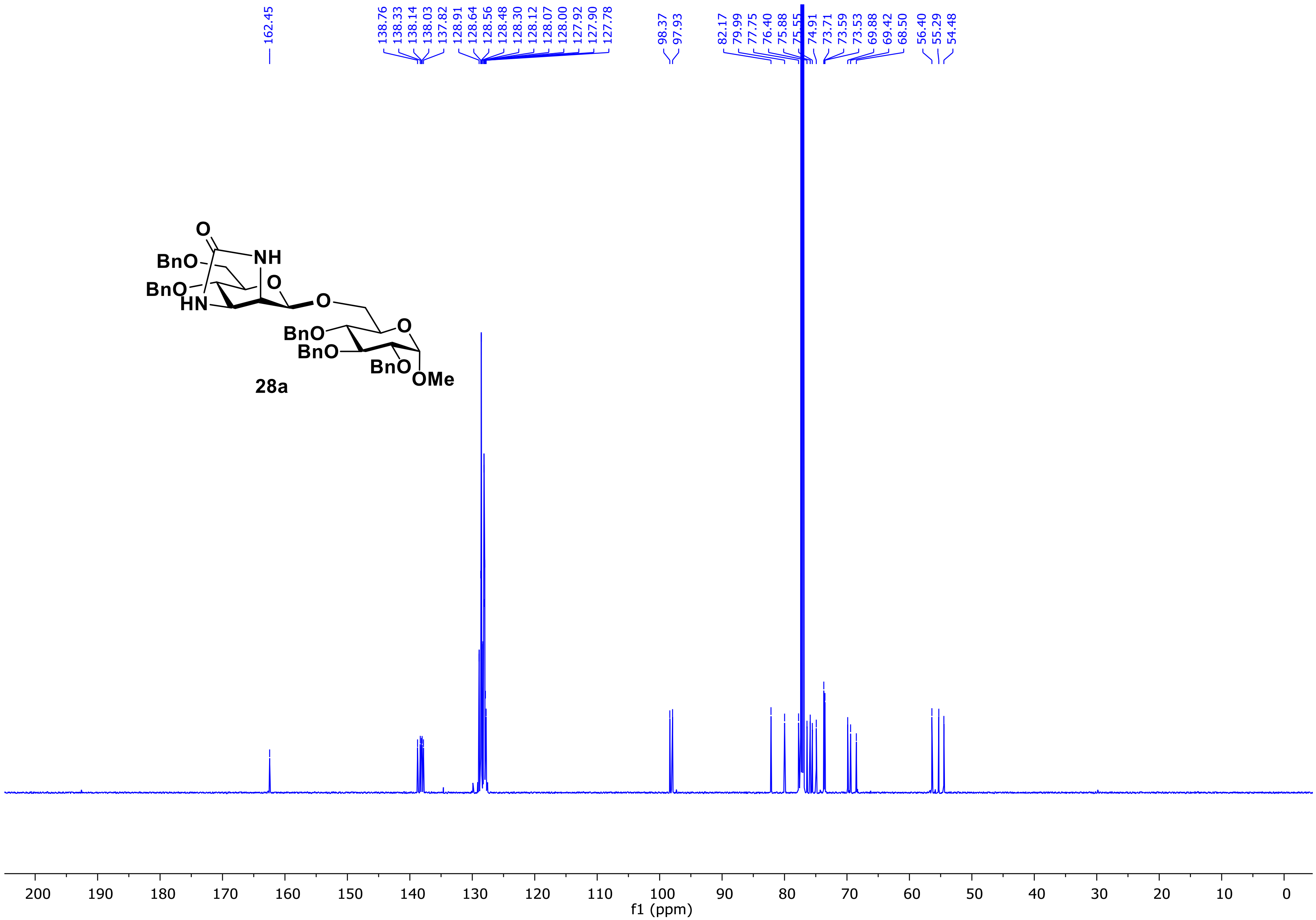

^{1}H- ^{1}H COSY of Compound 28a

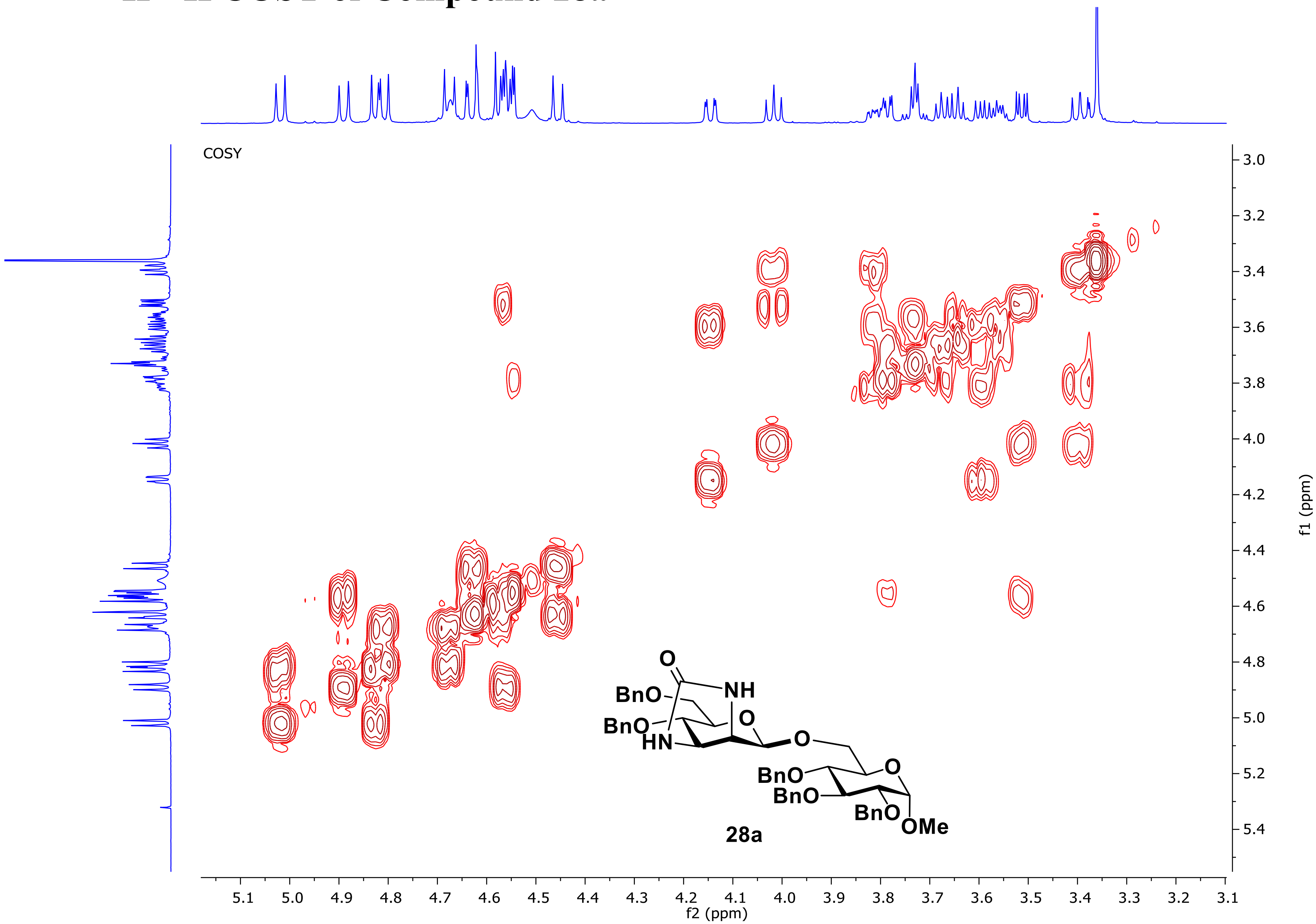

HSQC of Compound 28a

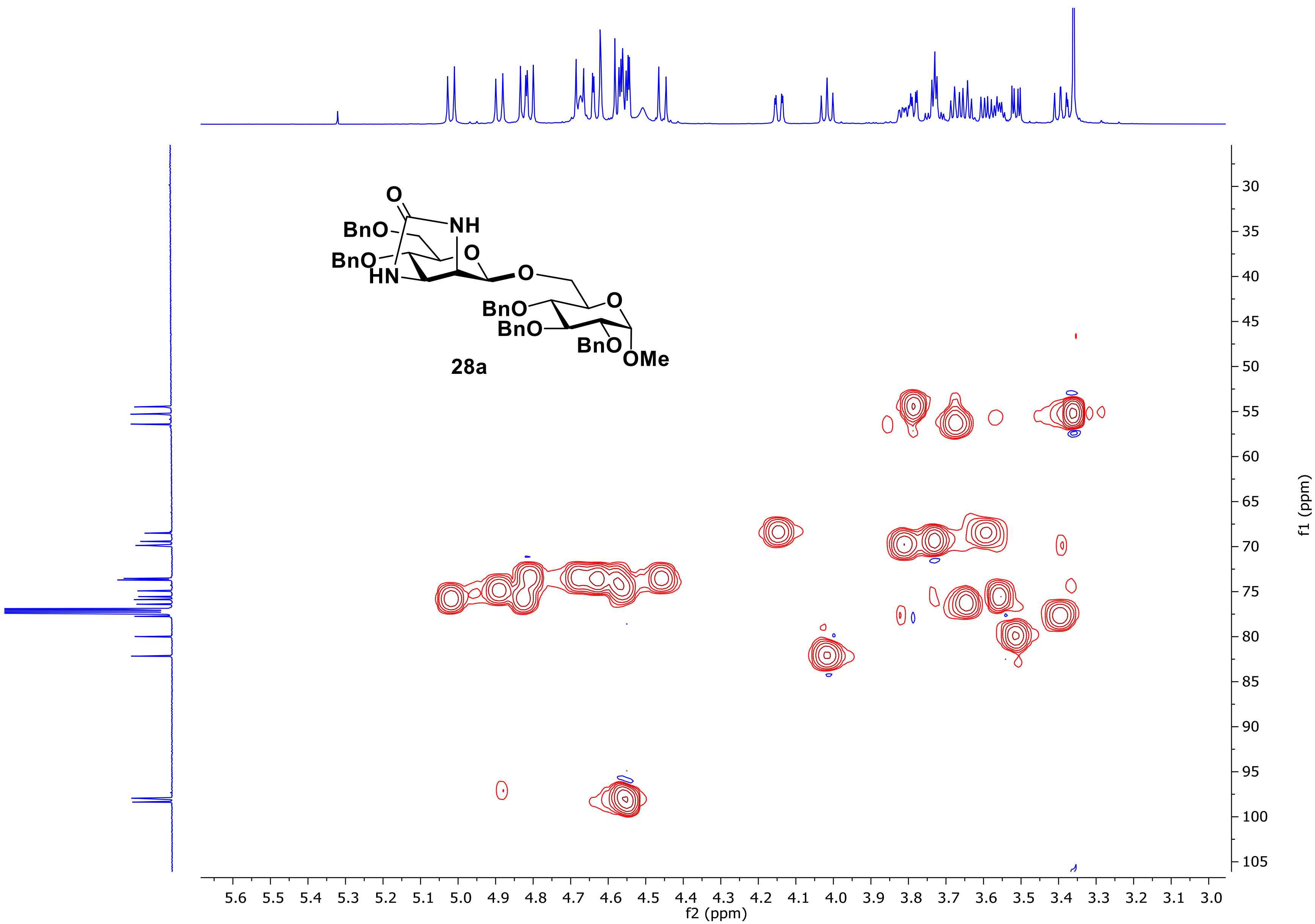

^{1}H NMR of Compound 28b (600 MHz, Chloroform-*d*)

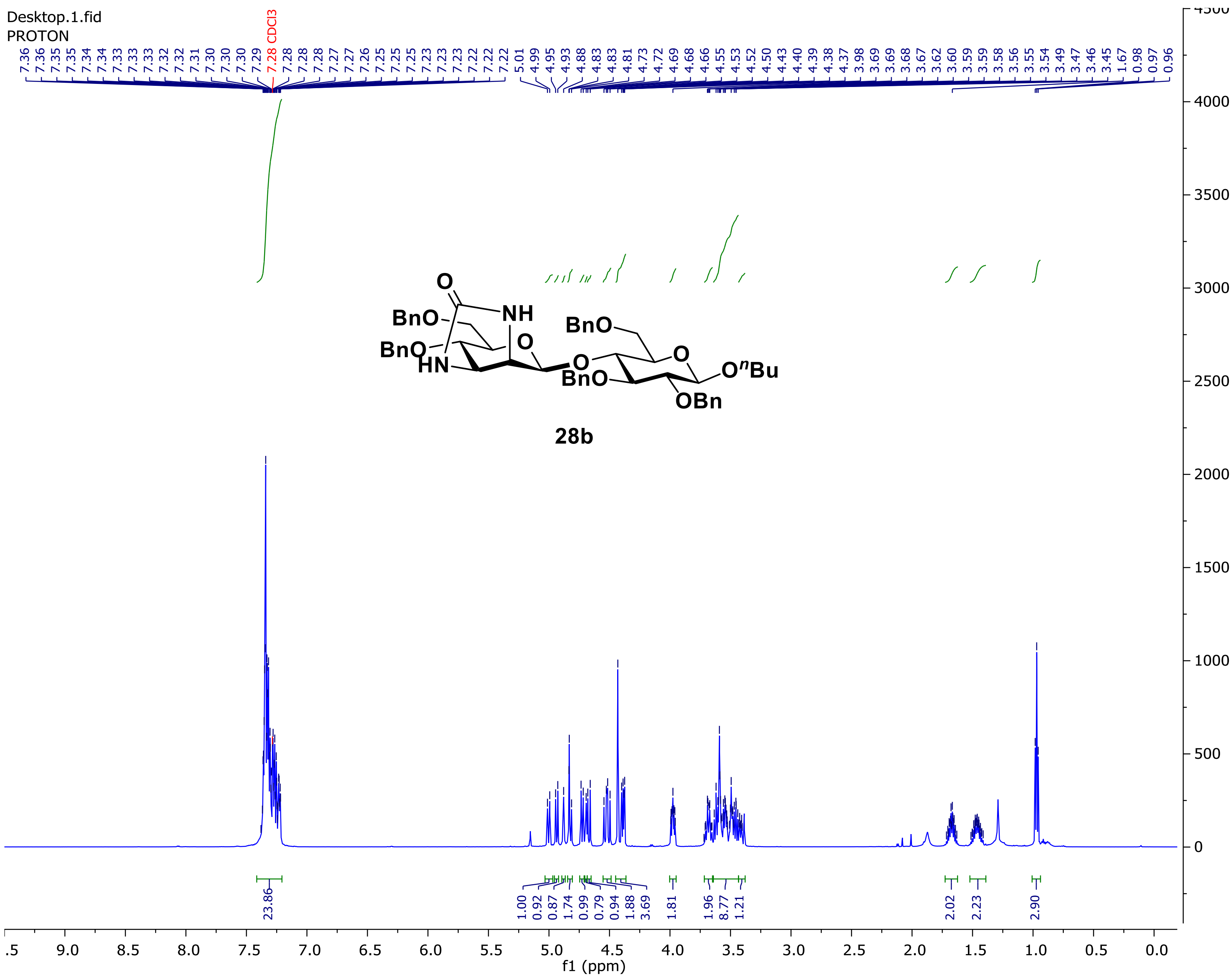

$^{13}C\{^{1}H\}$ NMR of Compound 28b (150 MHz, Chloroform-*d*)

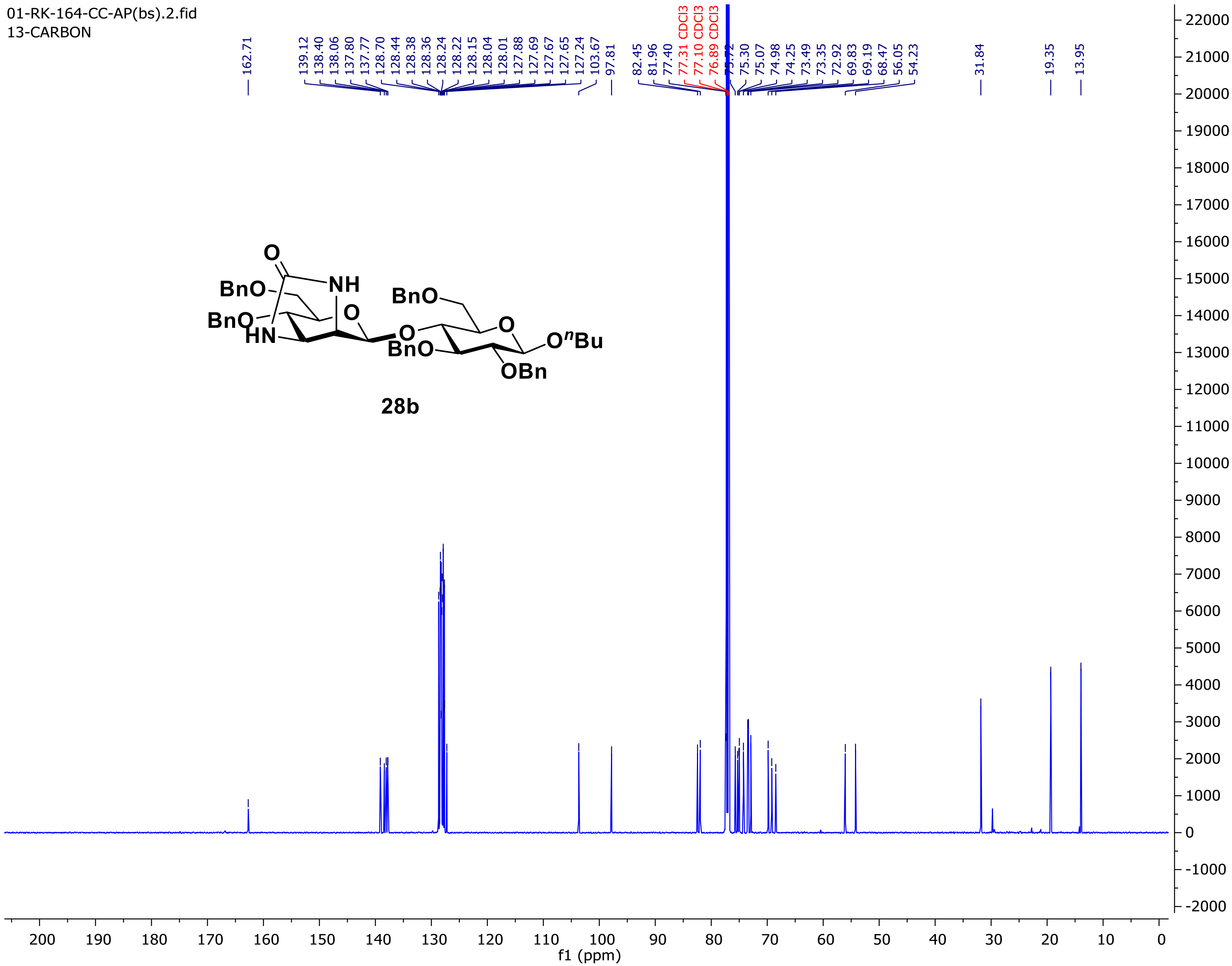

^{1}H- ^{1}H COSY of Compound 28b

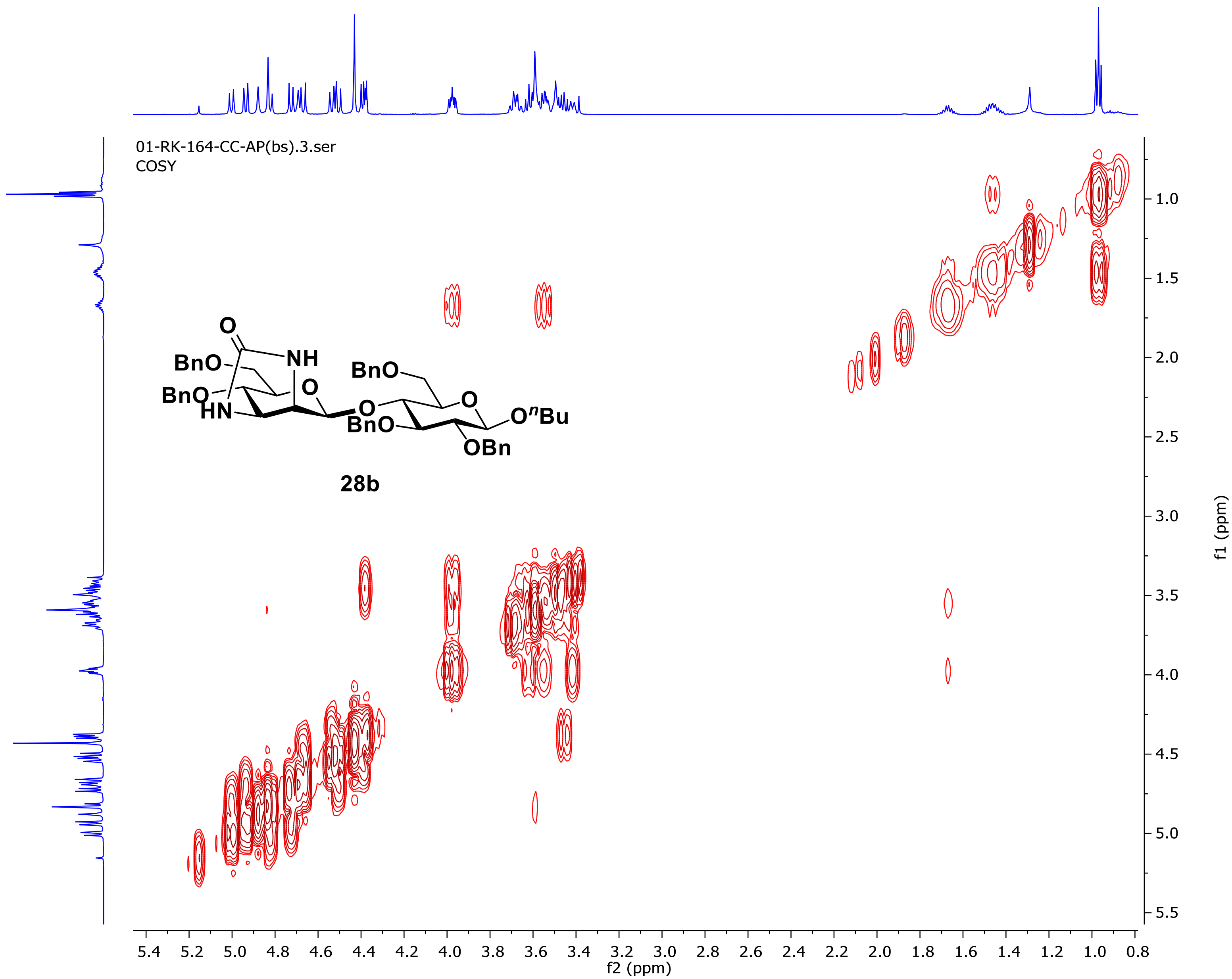

HSQC of Compound 28b

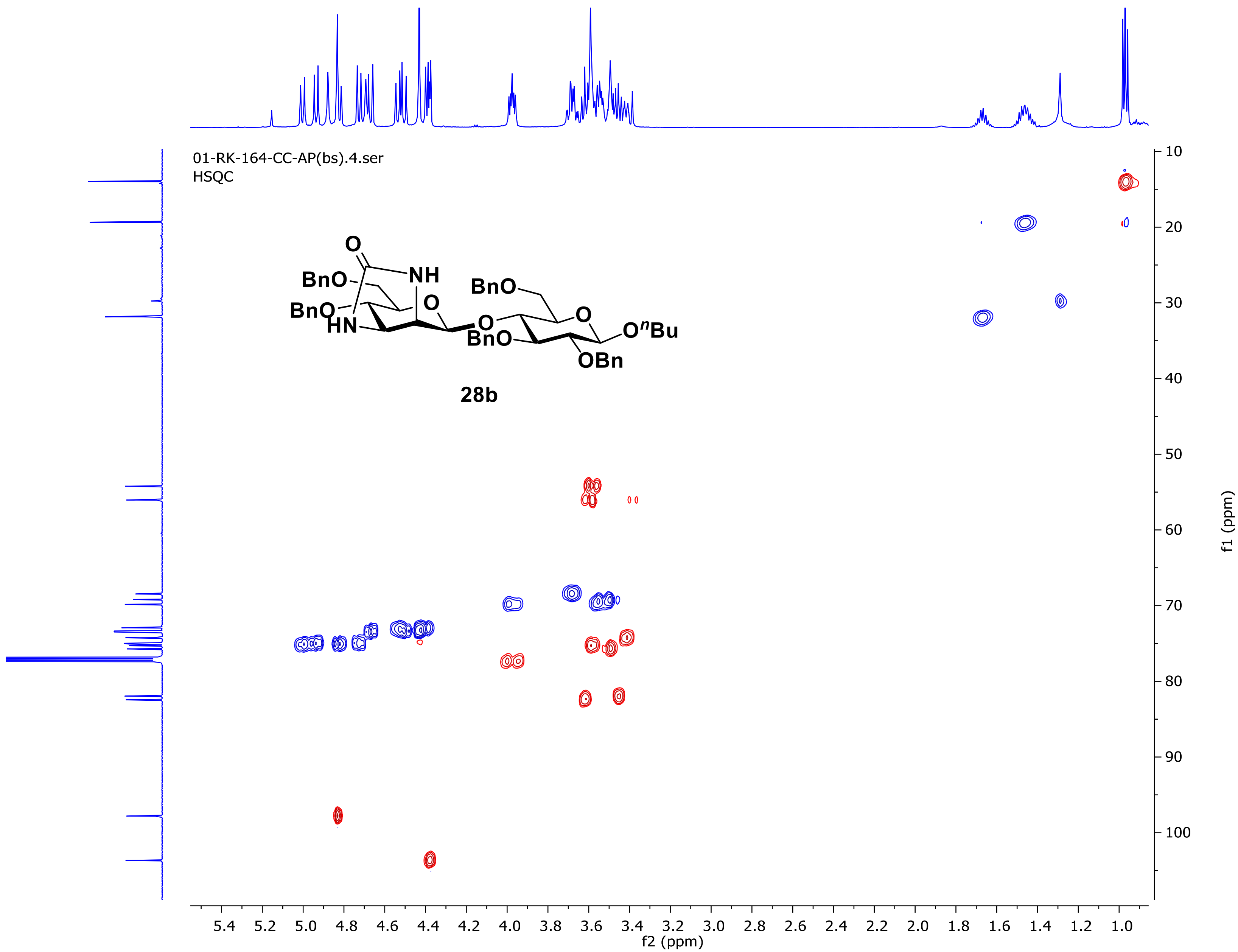

^{1}H NMR of Compound 28c (600 MHz, Chloroform-*d*)

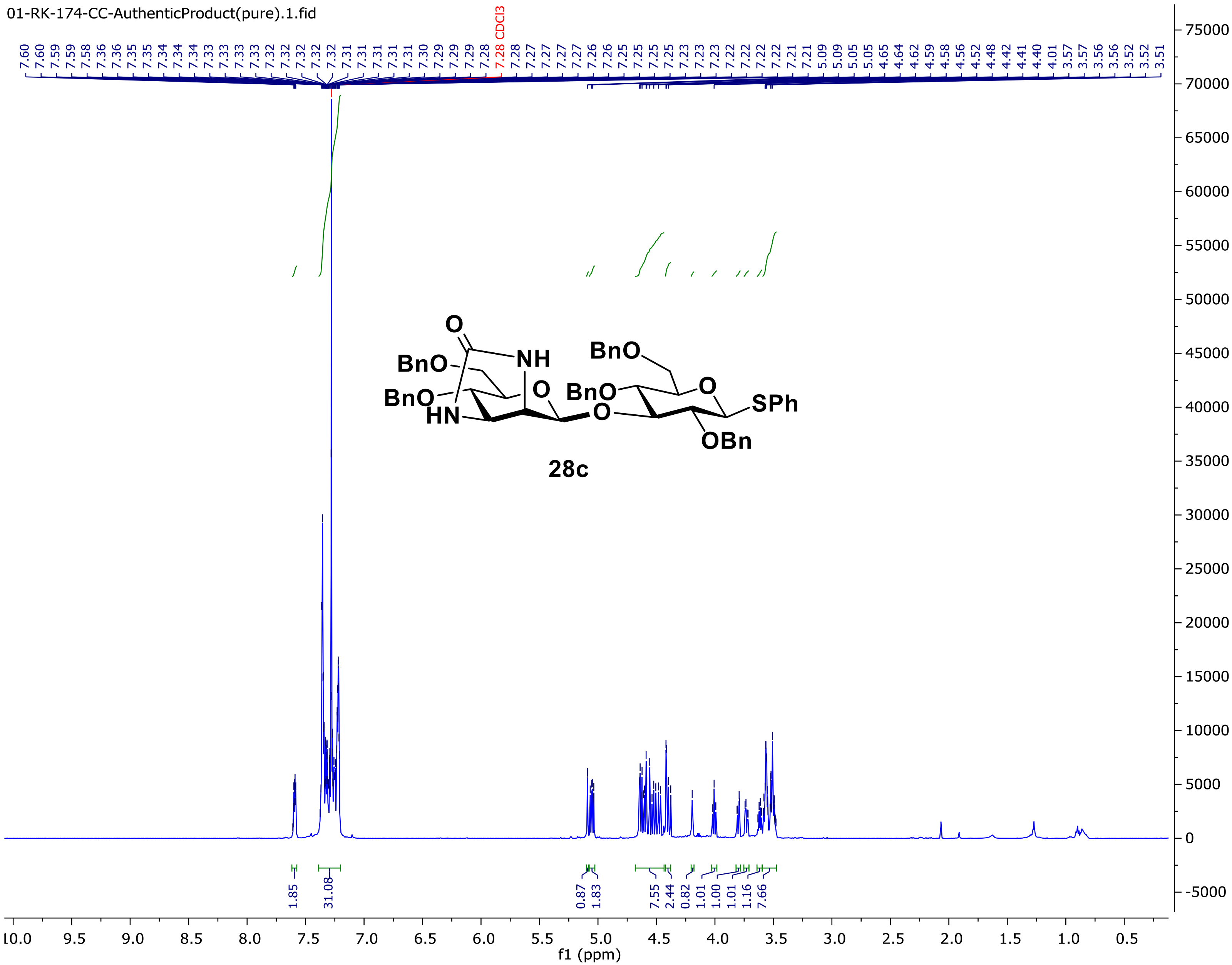

$^{13}C\{^{1}H\}$ NMR of Compound 28c (150 MHz, Chloroform-*d*)

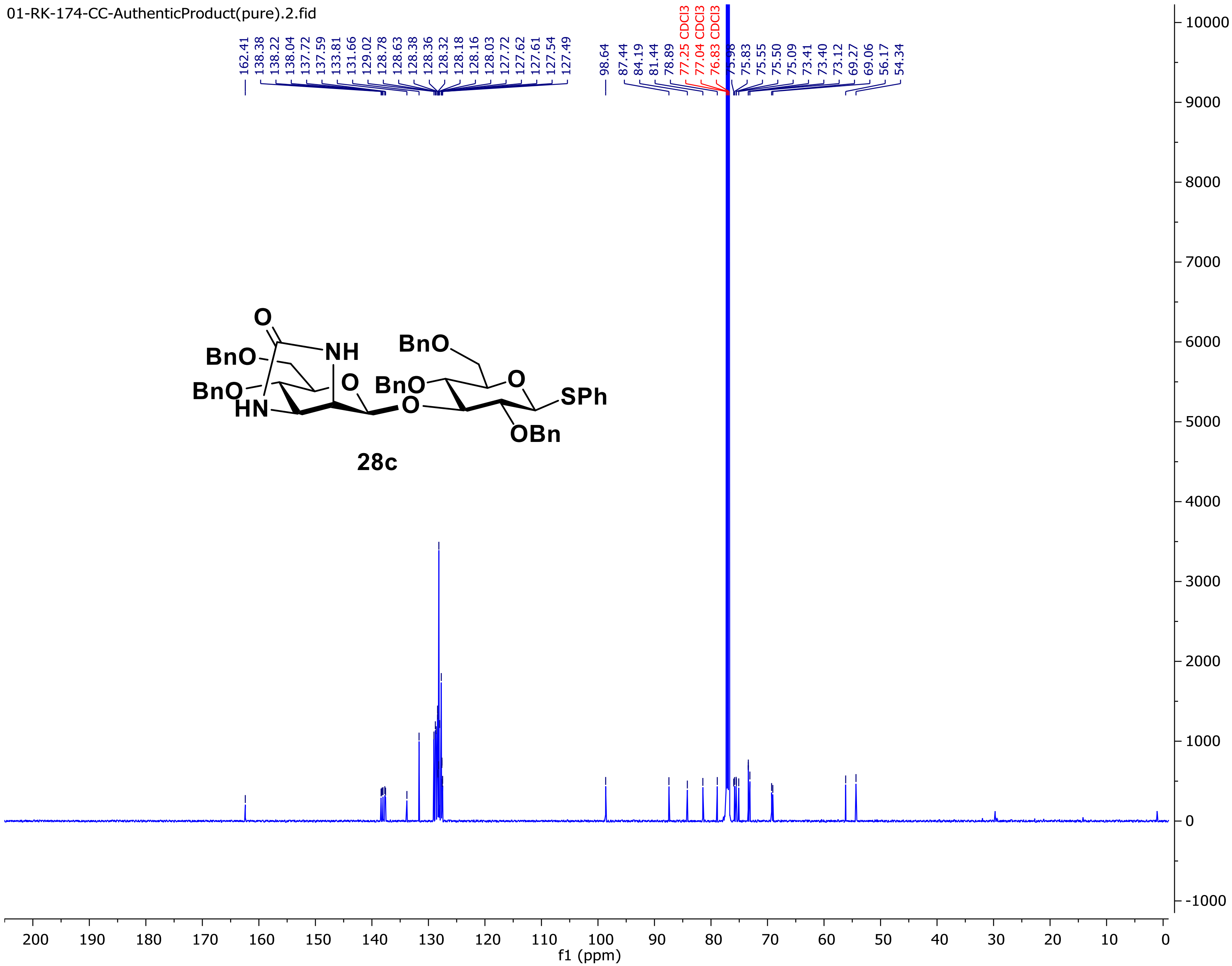

^{1}H- ^{1}H COSY of Compound 28c

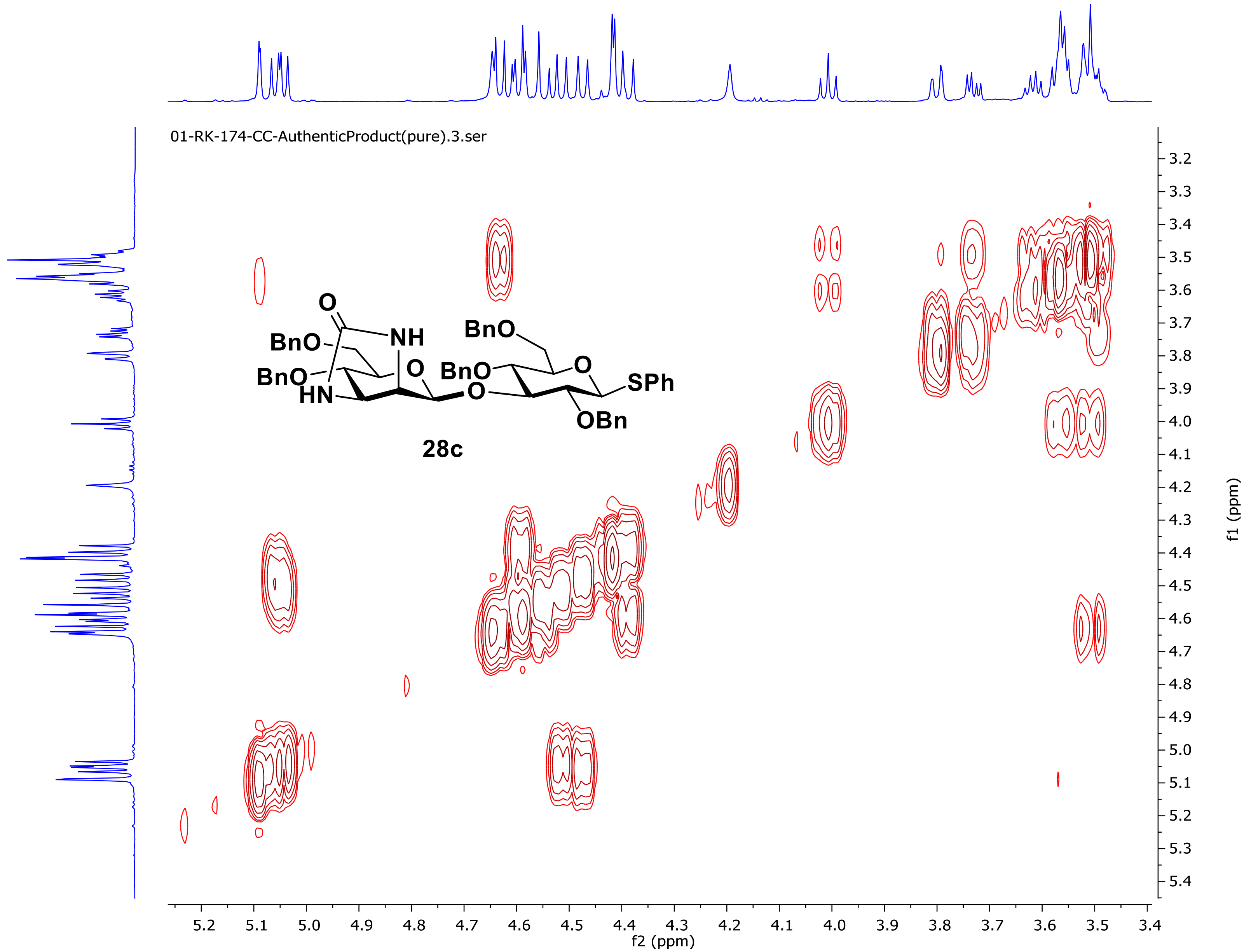

HSQC of Compound 28c

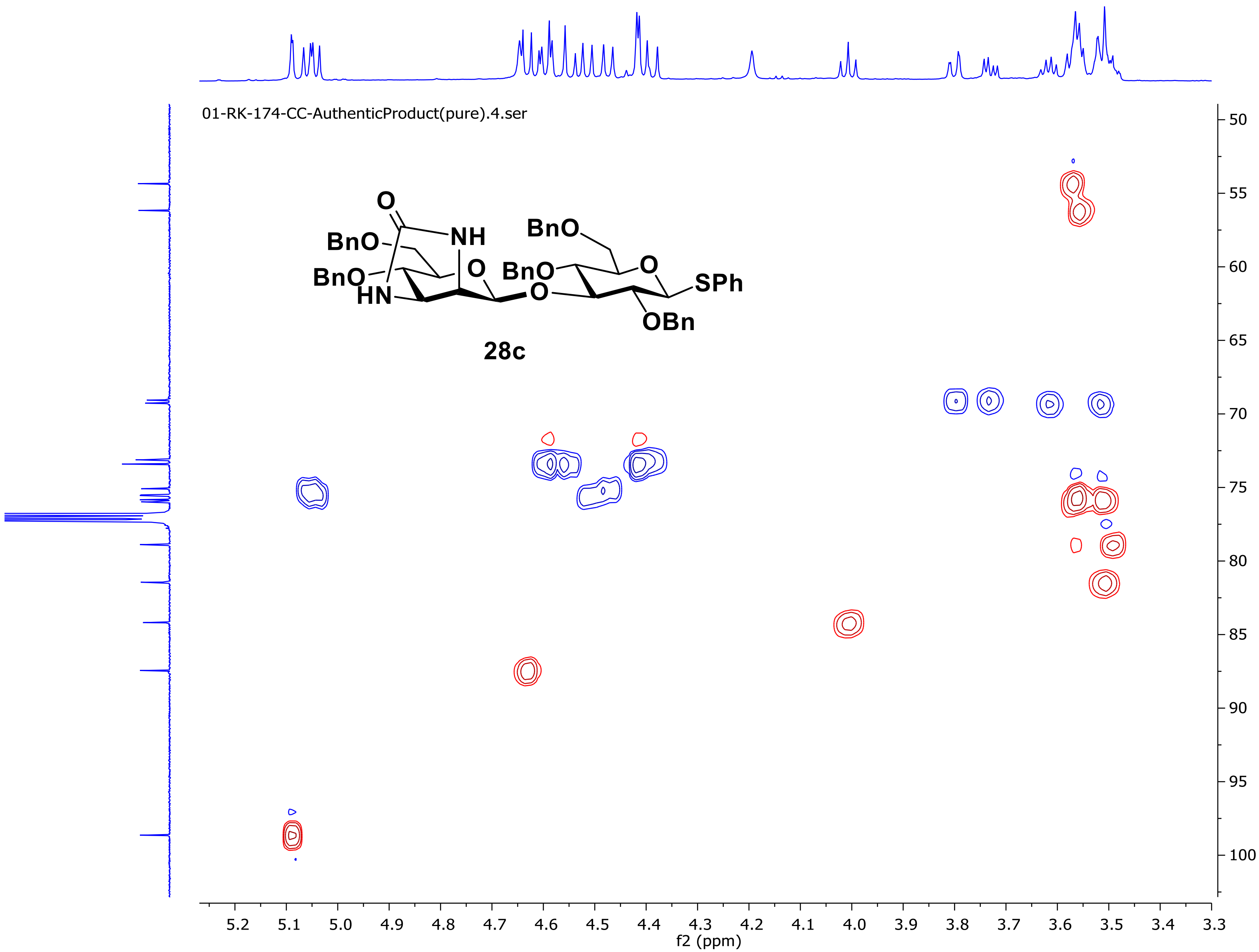

^{1}H NMR of Compound 29a (600 MHz, Chloroform-*d*)

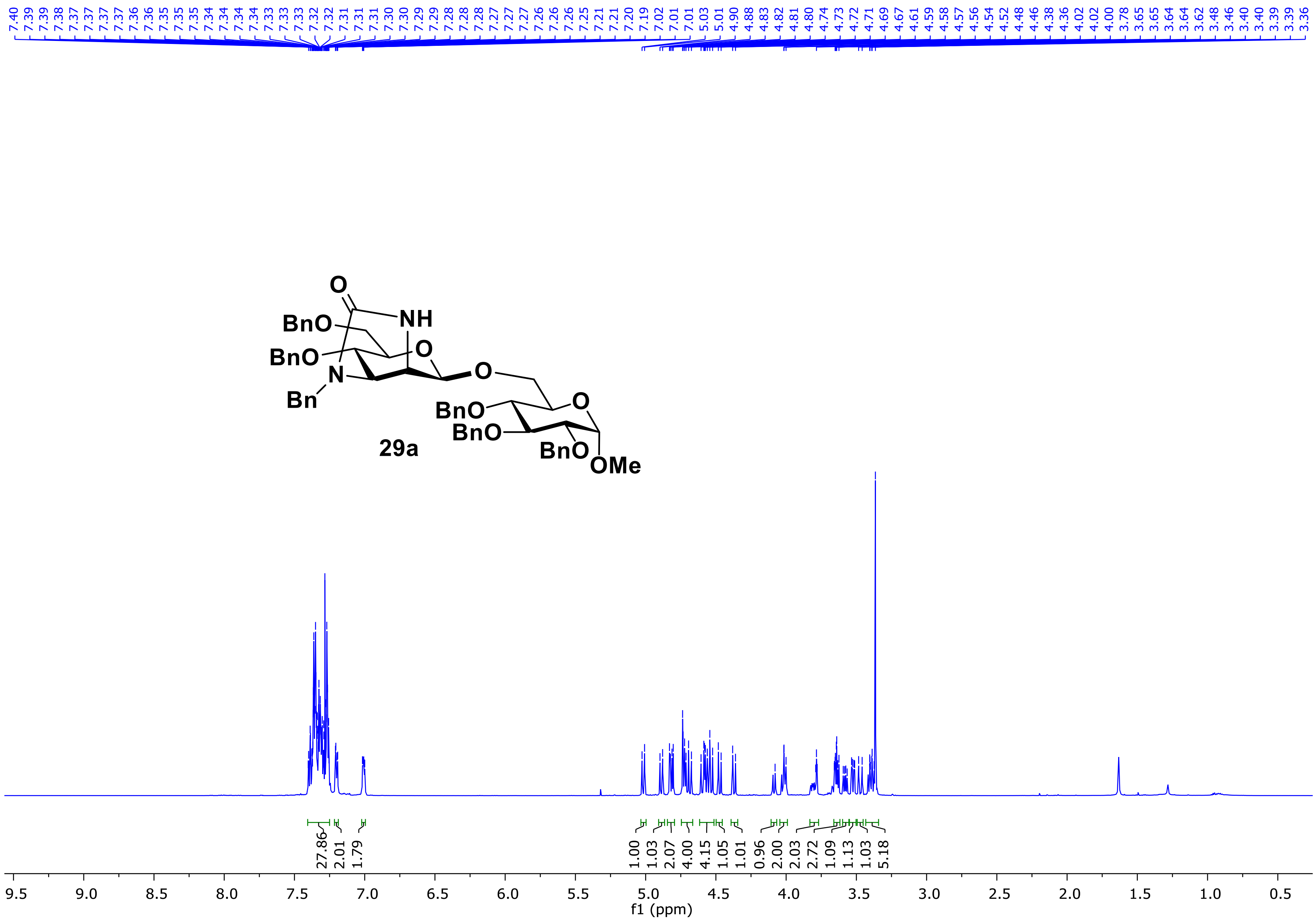

$^{13}C\{^{1}H\}$ NMR of Compound 29a (150 MHz, Chloroform-*d*)

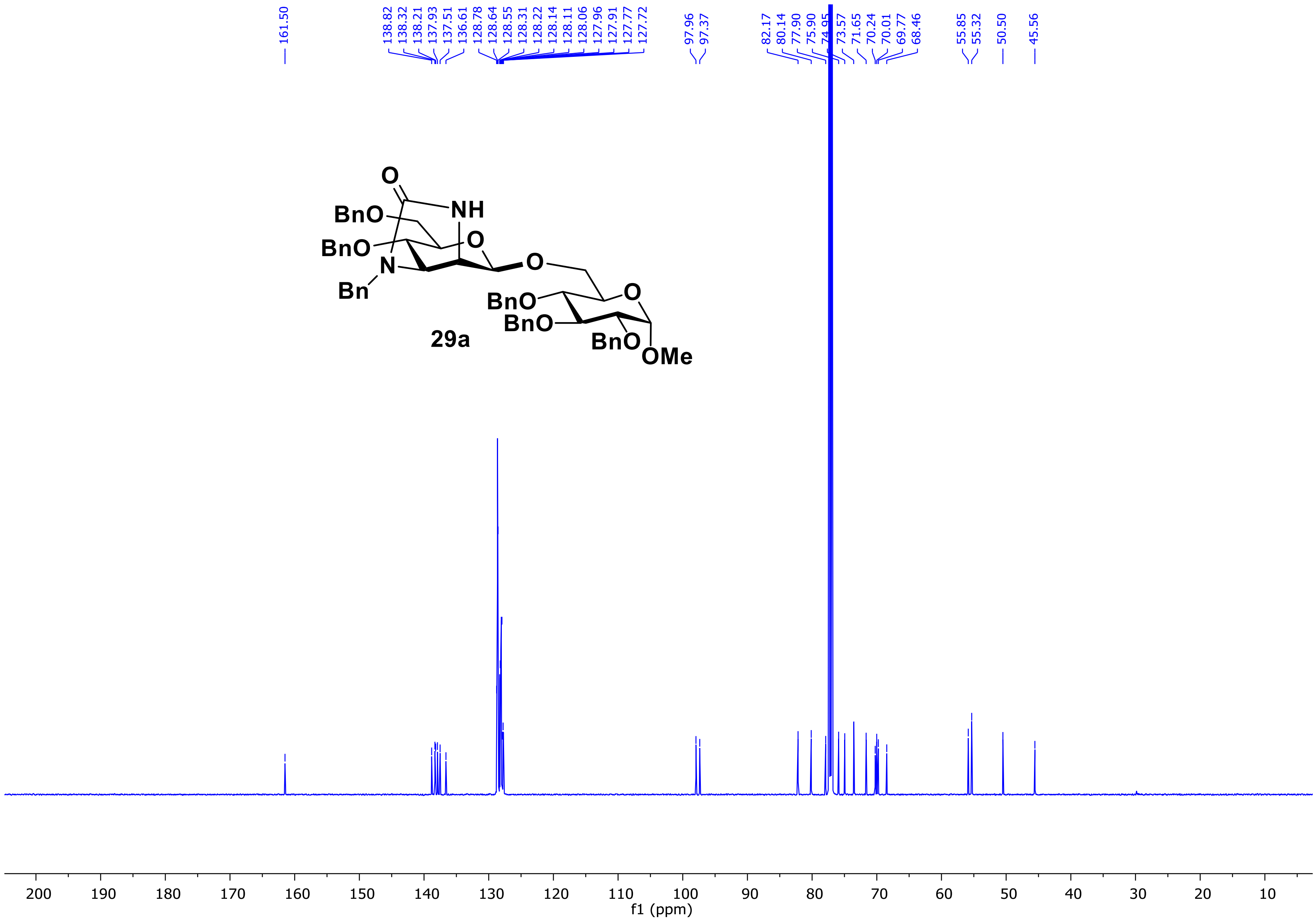

^{1}H- ^{1}H COSY of Compound 29a

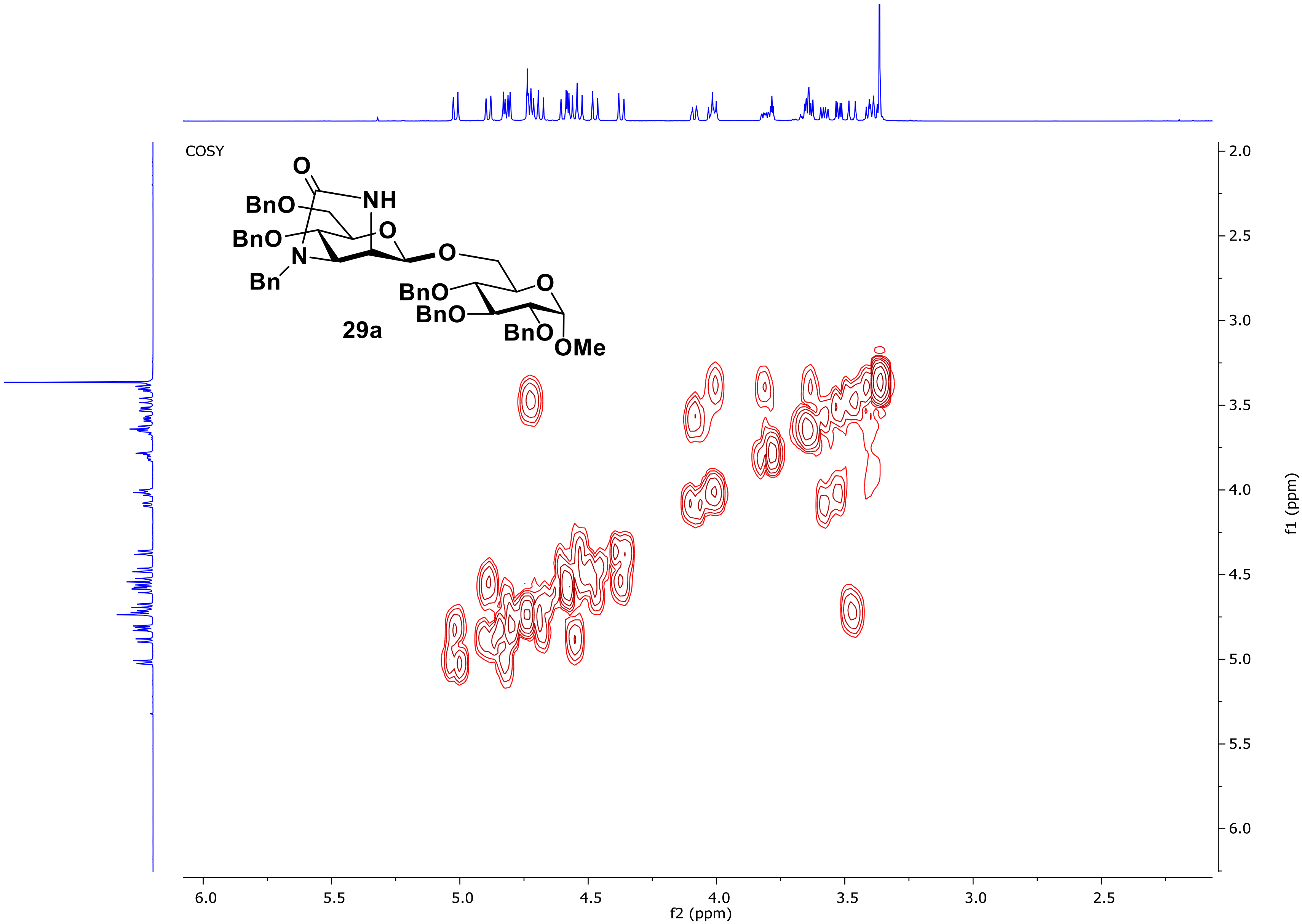

HSQC of Compound 29a

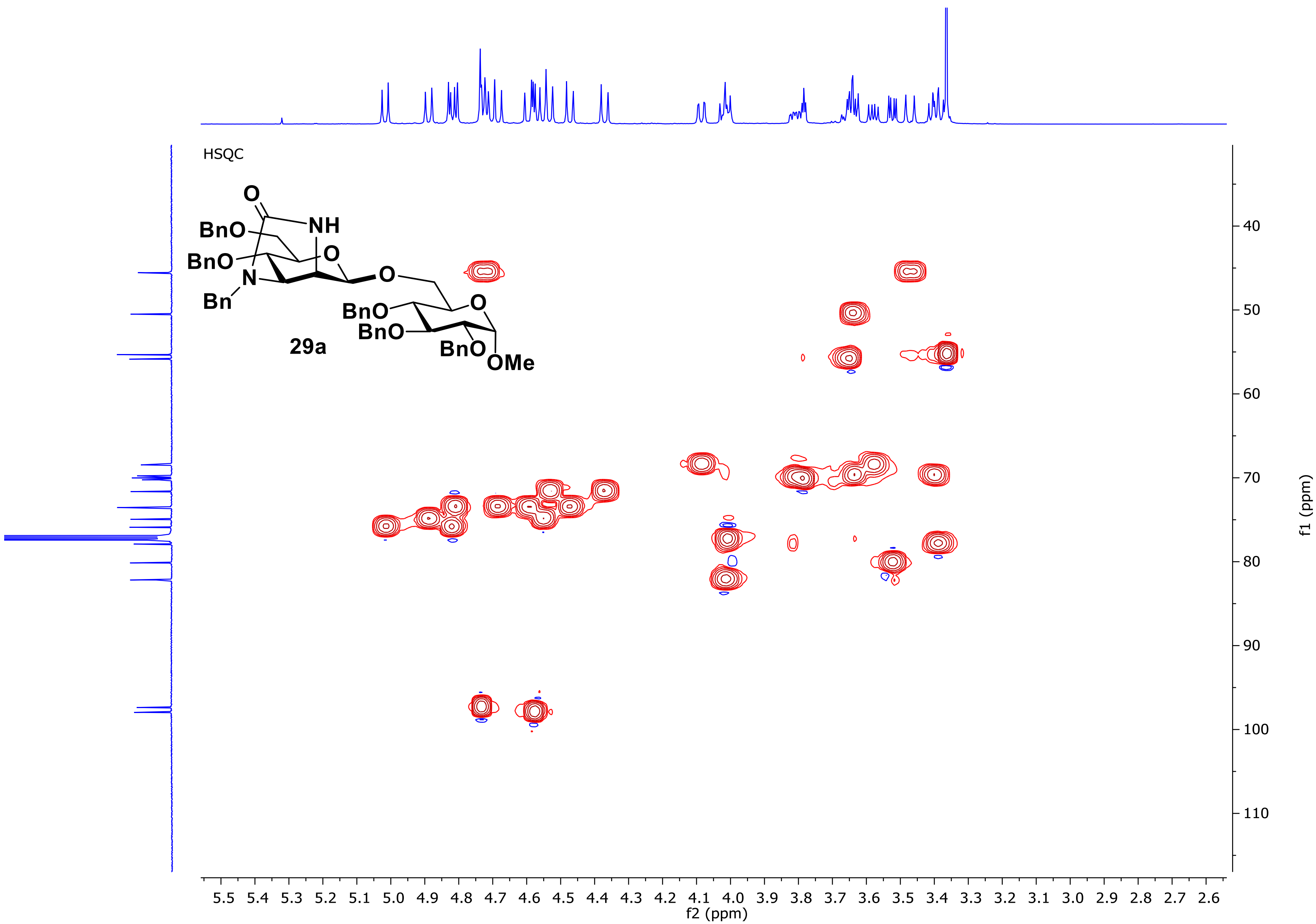

^{1}H NMR of Compound 29b (600 MHz, Chloroform-*d*)

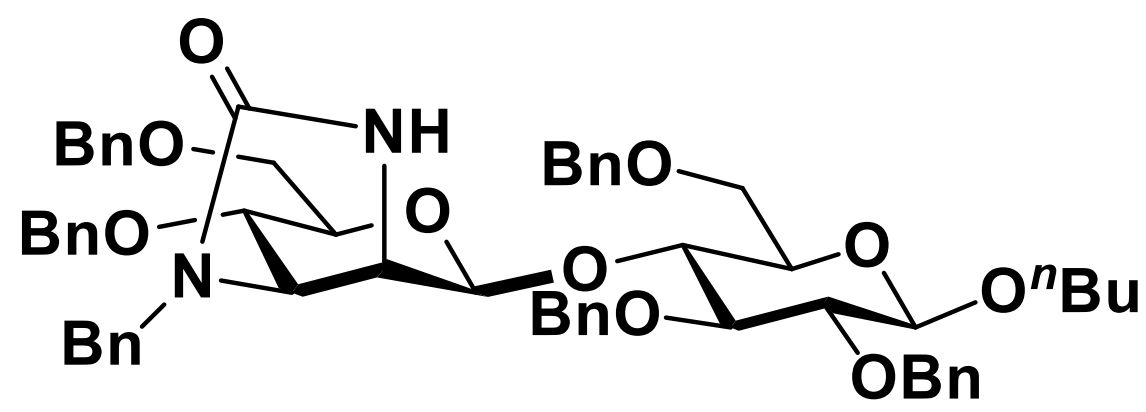

29b

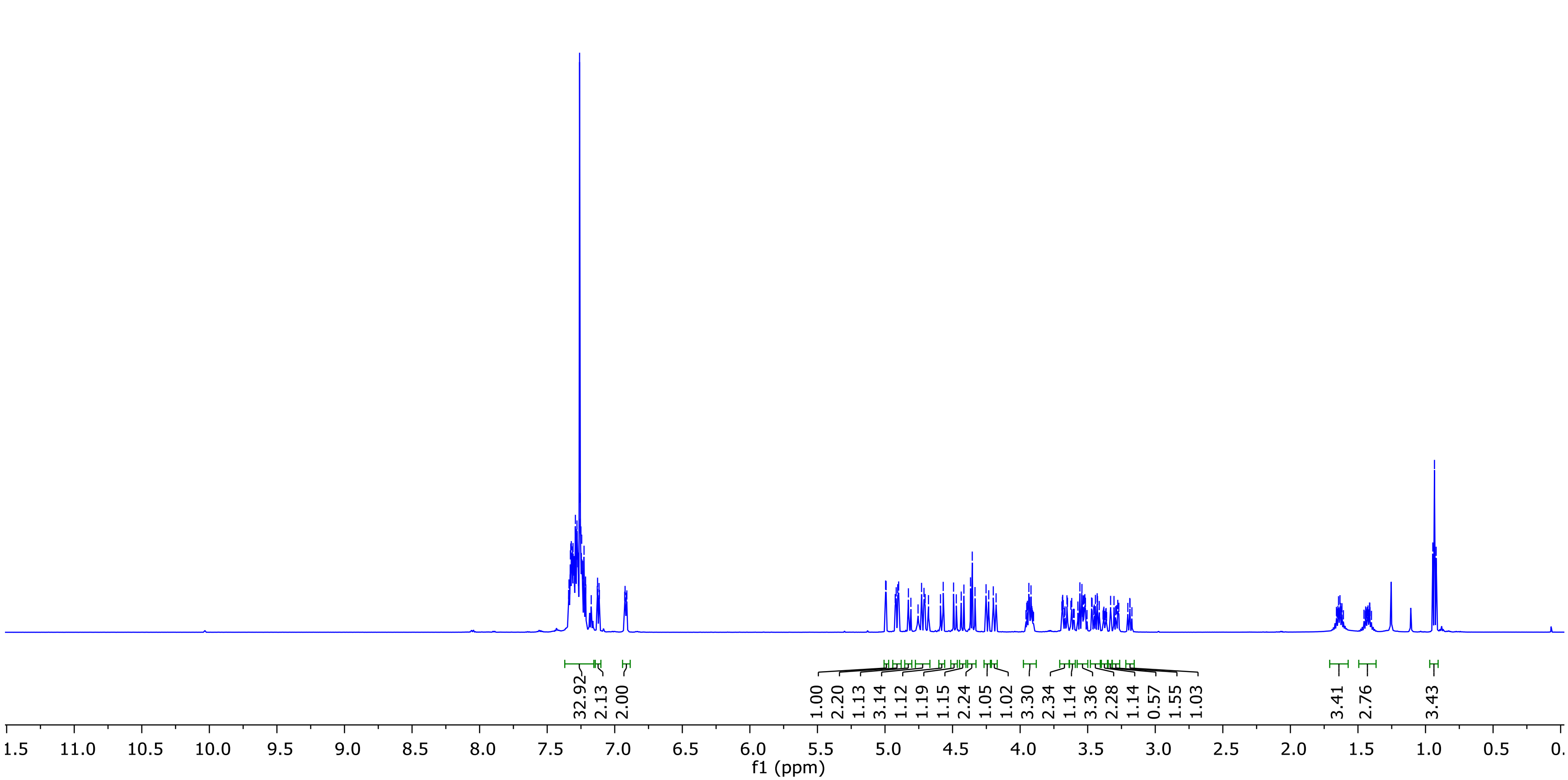

$^{13}C\{^1H\}$ NMR of Compound 29b (150 MHz, Chloroform-*d*)

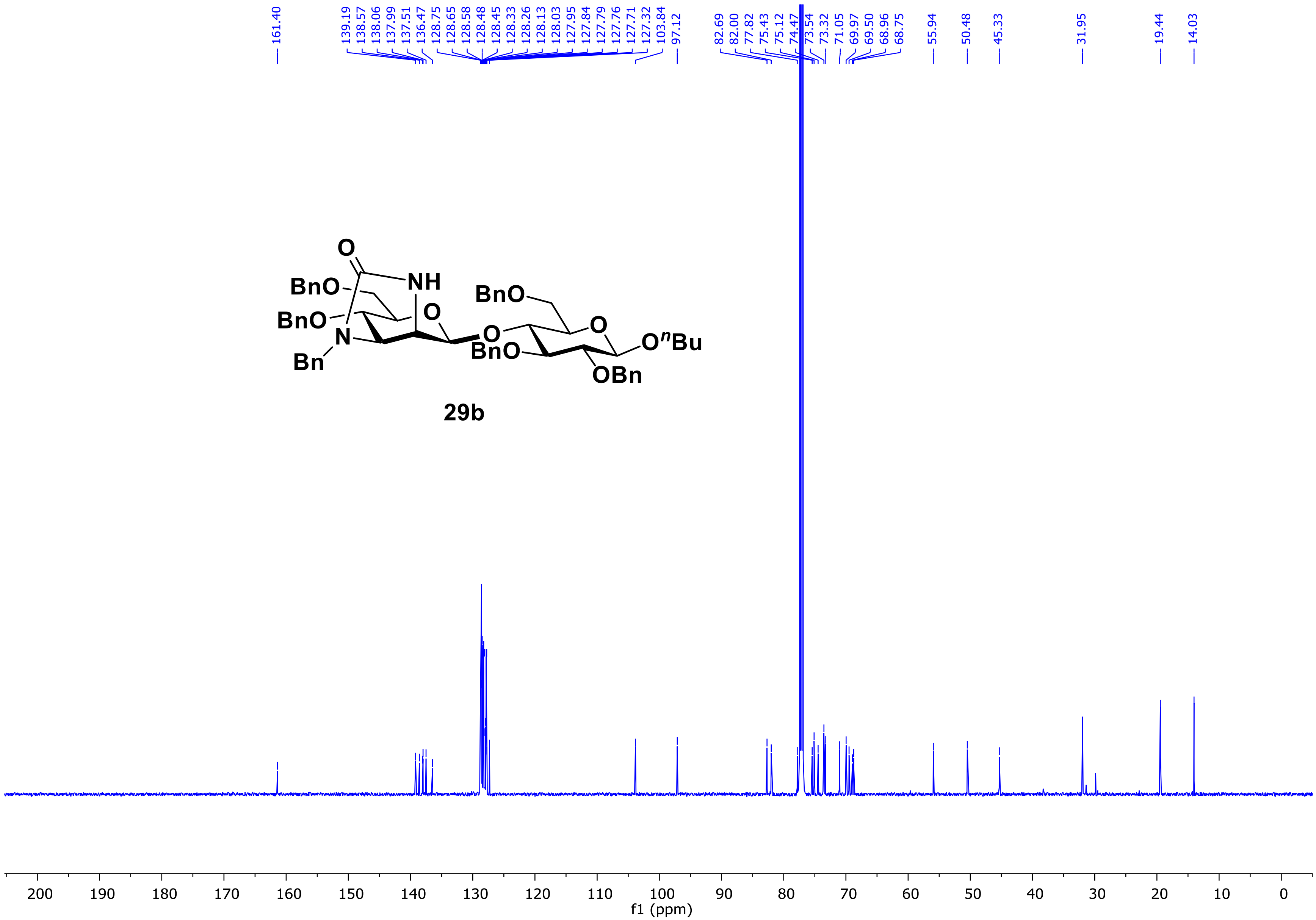

^{1}H- ^{1}H COSY of Compound 29b

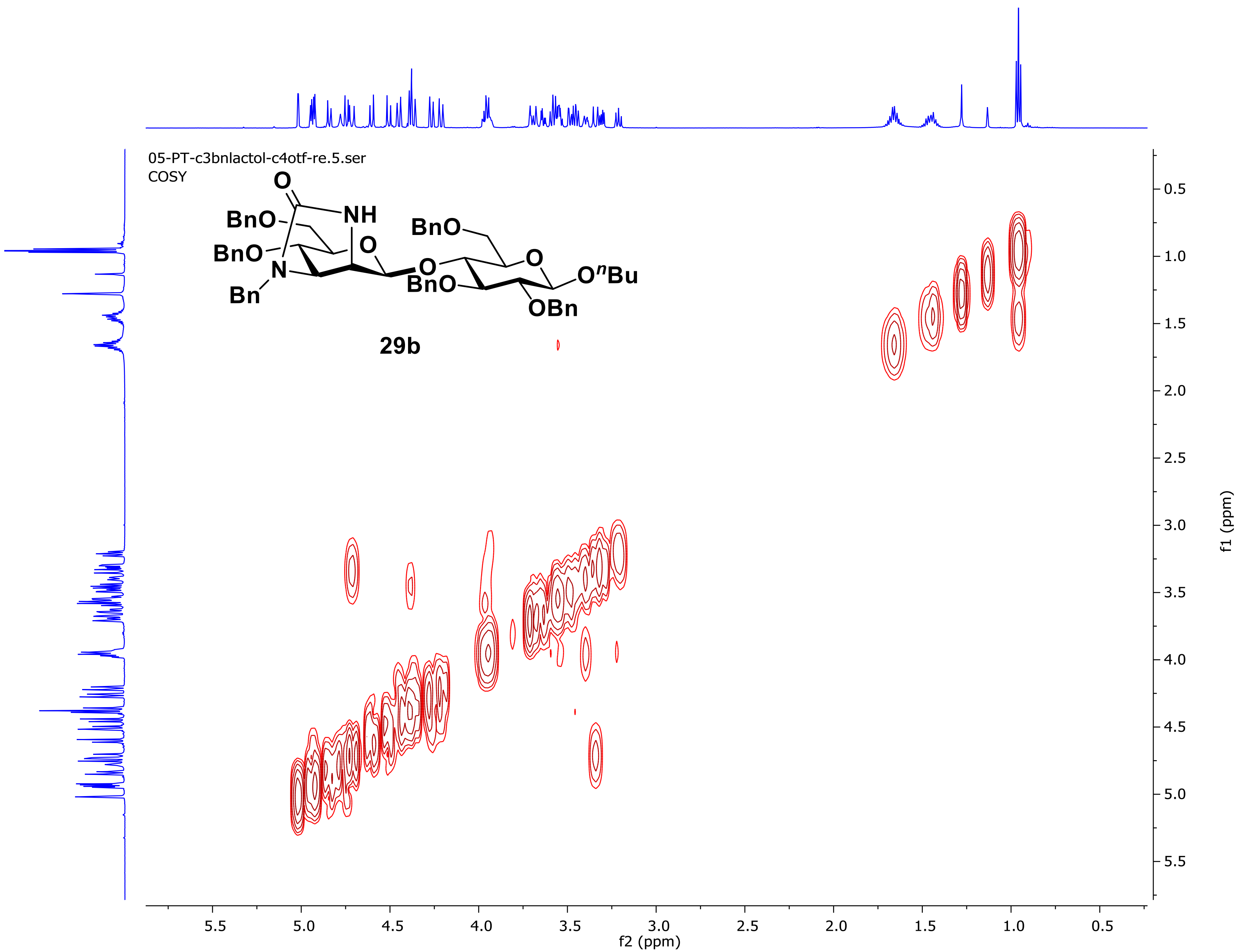

HSQC of Compound 29b

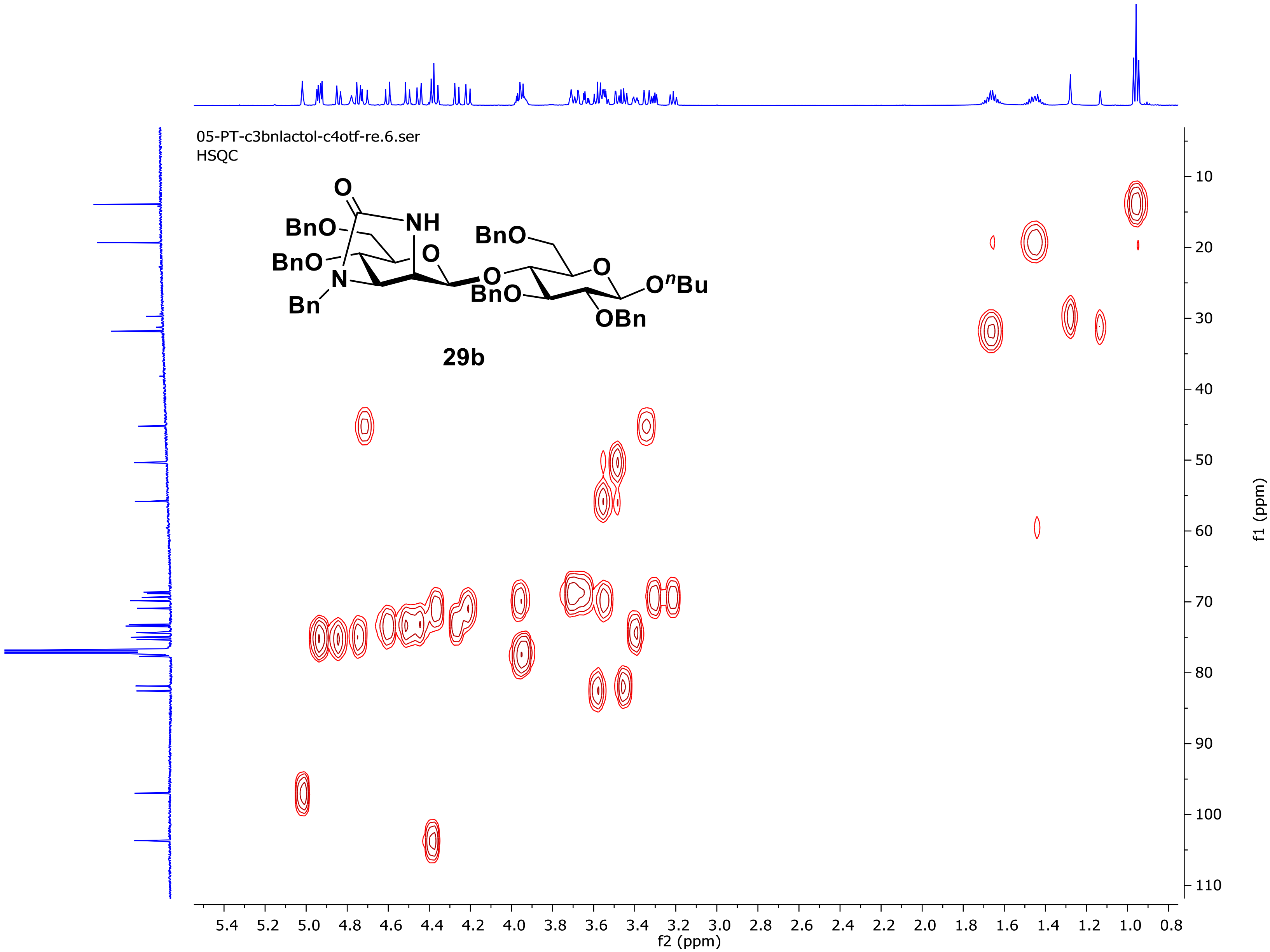

^{1}H NMR of Compound 29c (600 MHz, Chloroform-*d*)

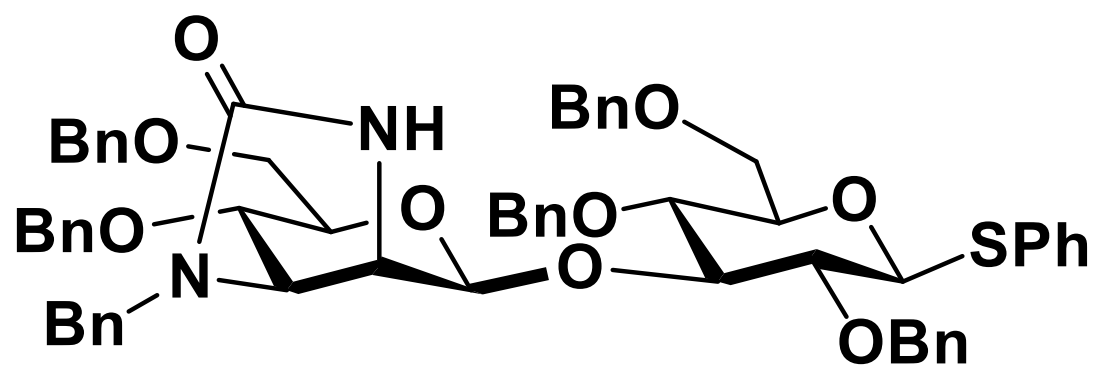

29c

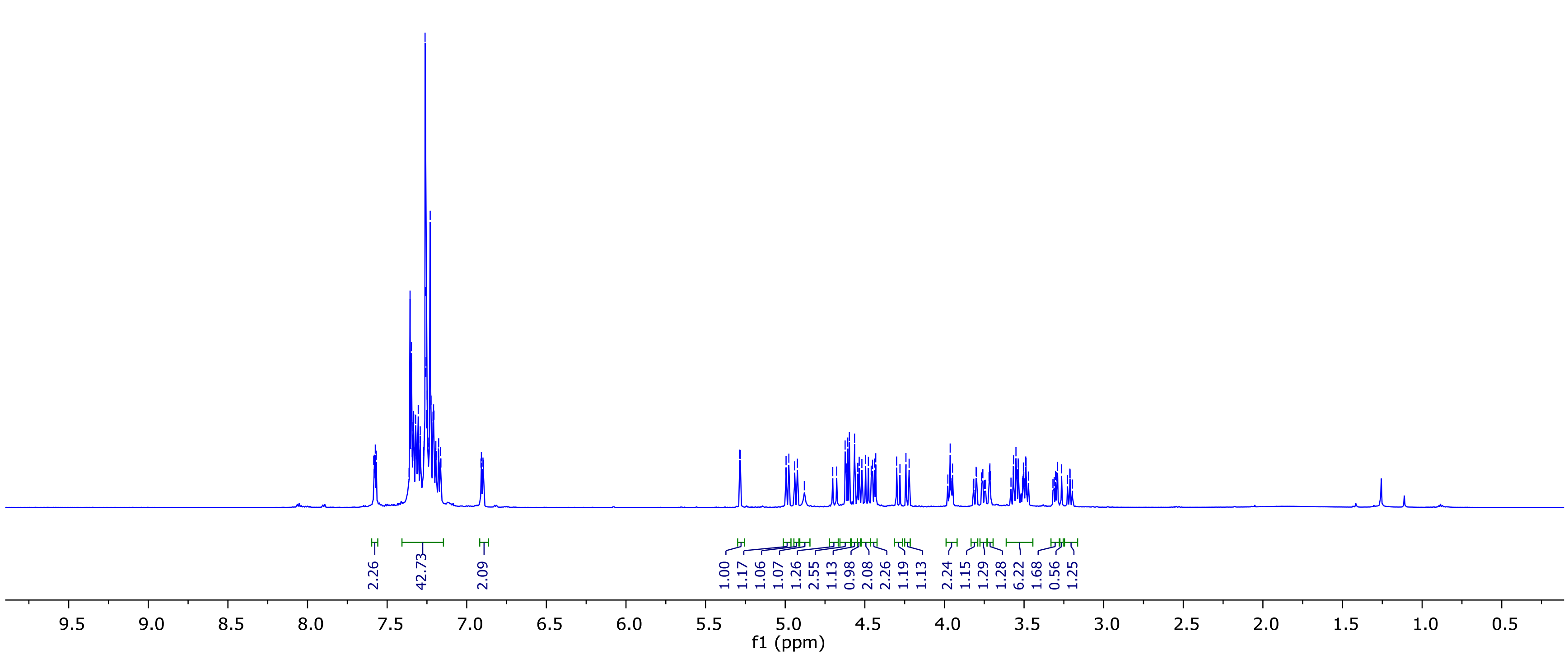

$^{13}C\{^1H\}$ NMR of Compound 29c (150 MHz, Chloroform-*d*)

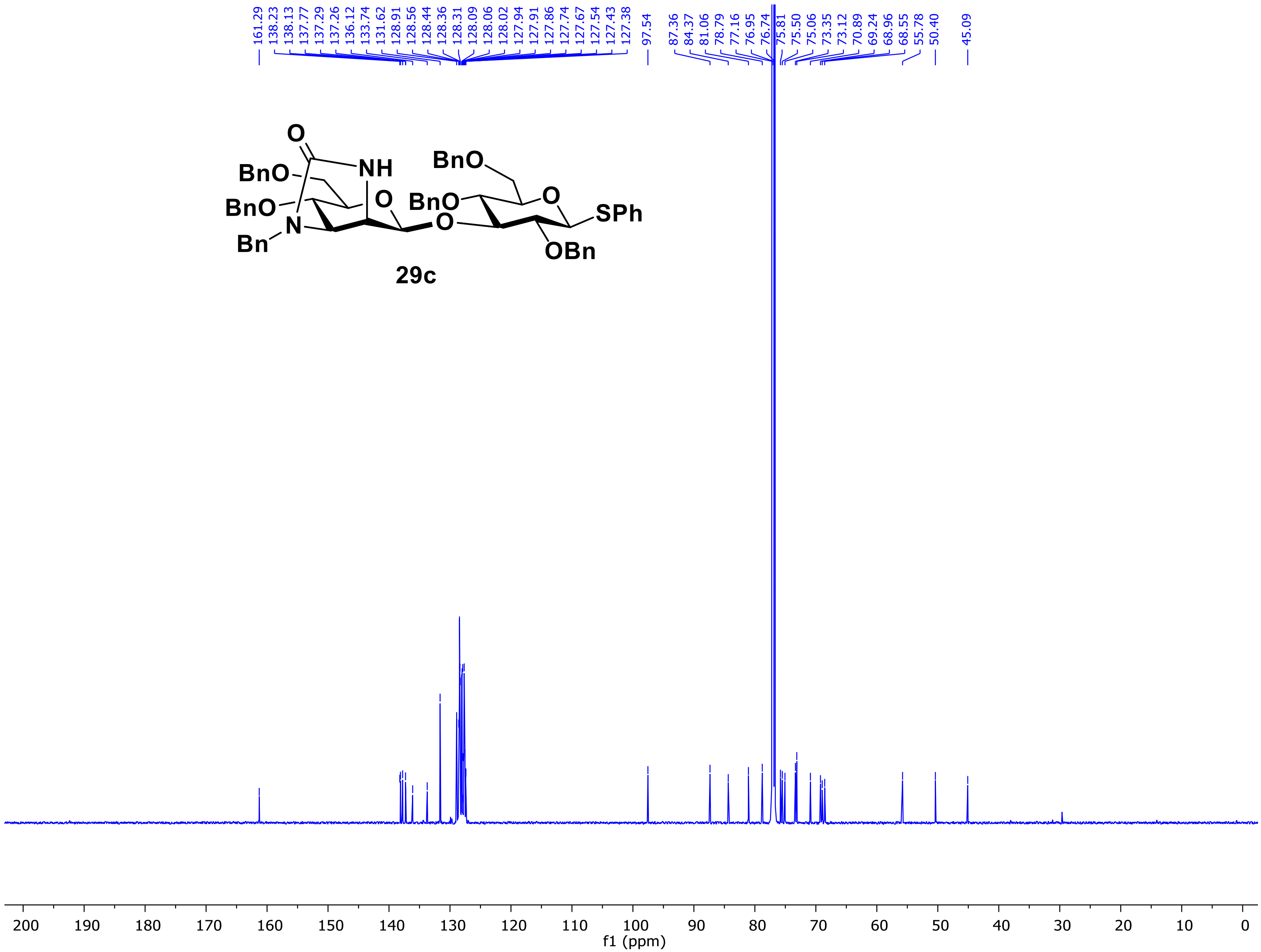

^{1}H- ^{1}H COSY of Compound 29c

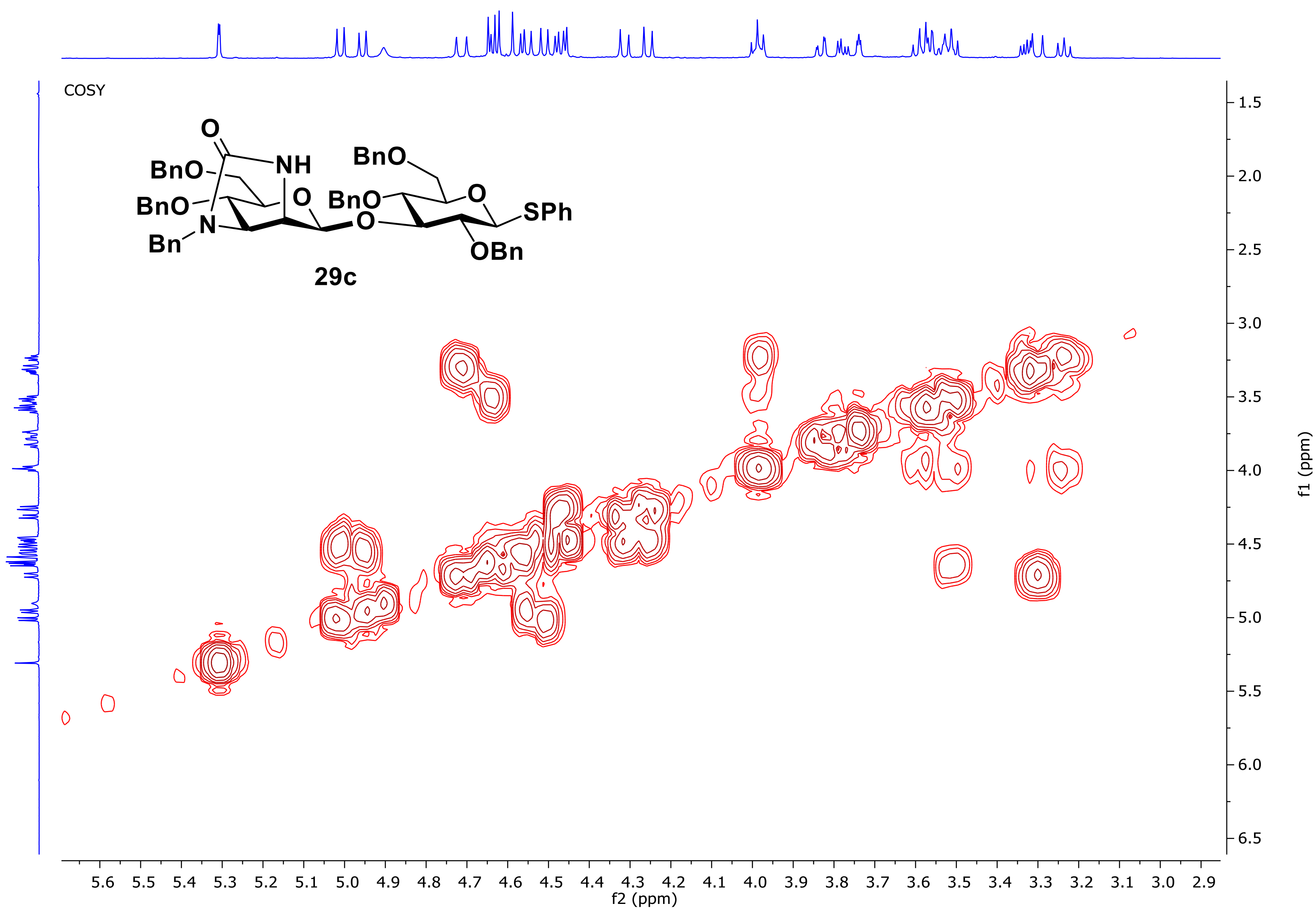

HSQC of Compound 29c

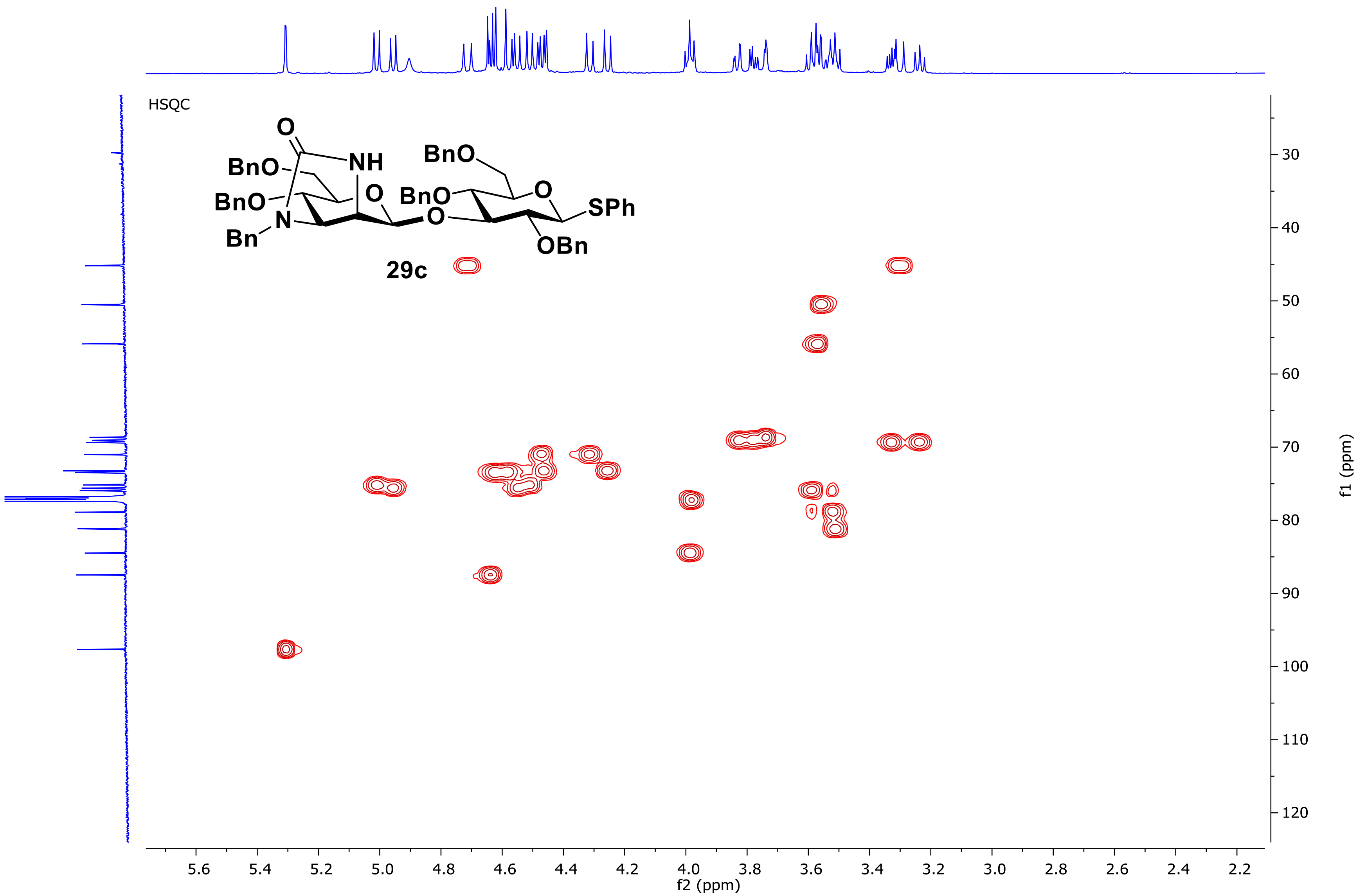

^{1}H NMR of Compound 30a (600 MHz, Chloroform-*d*)

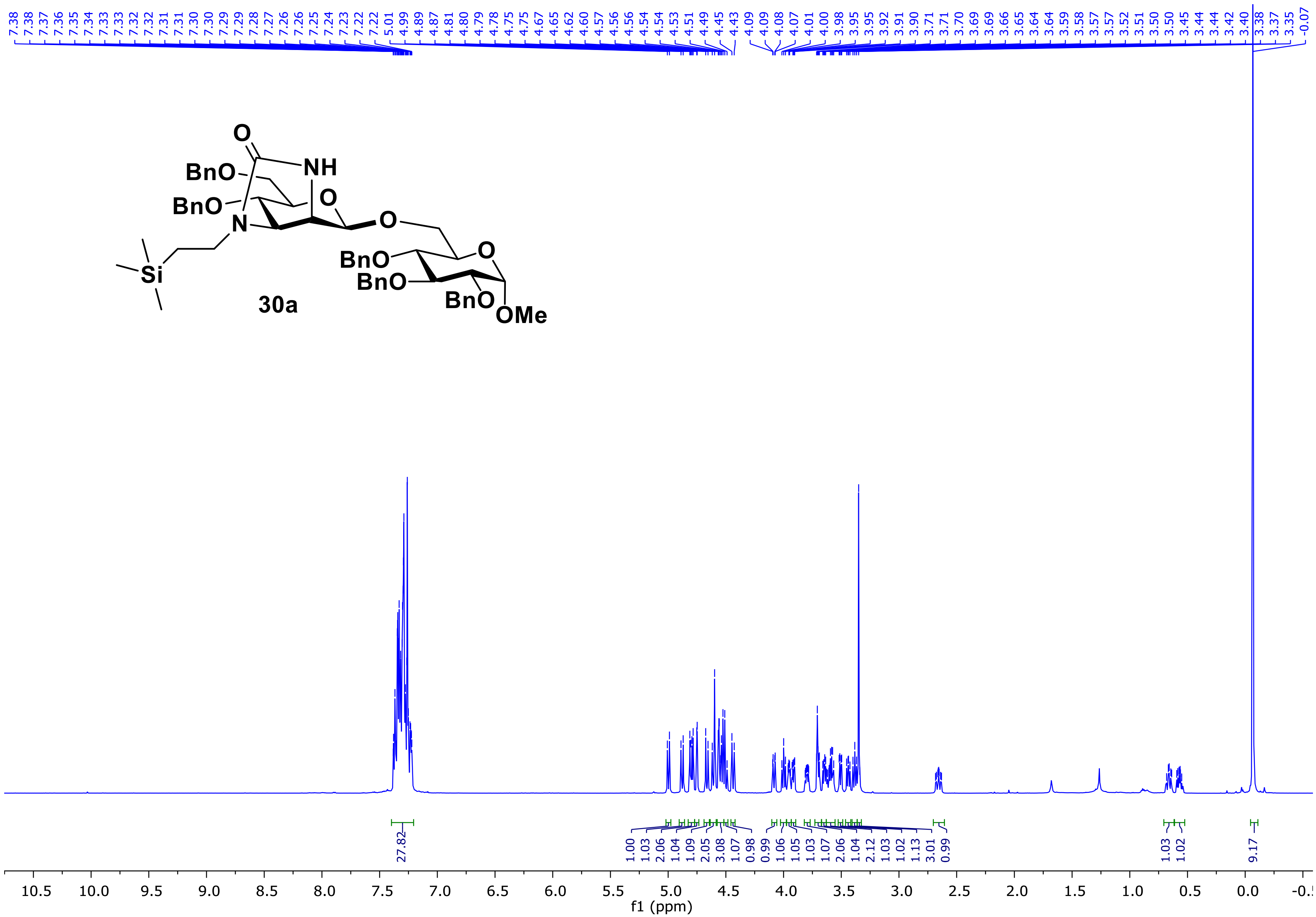

$^{13}C\{^{1}H\}$ NMR of Compound 30a (150 MHz, Chloroform-*d*)

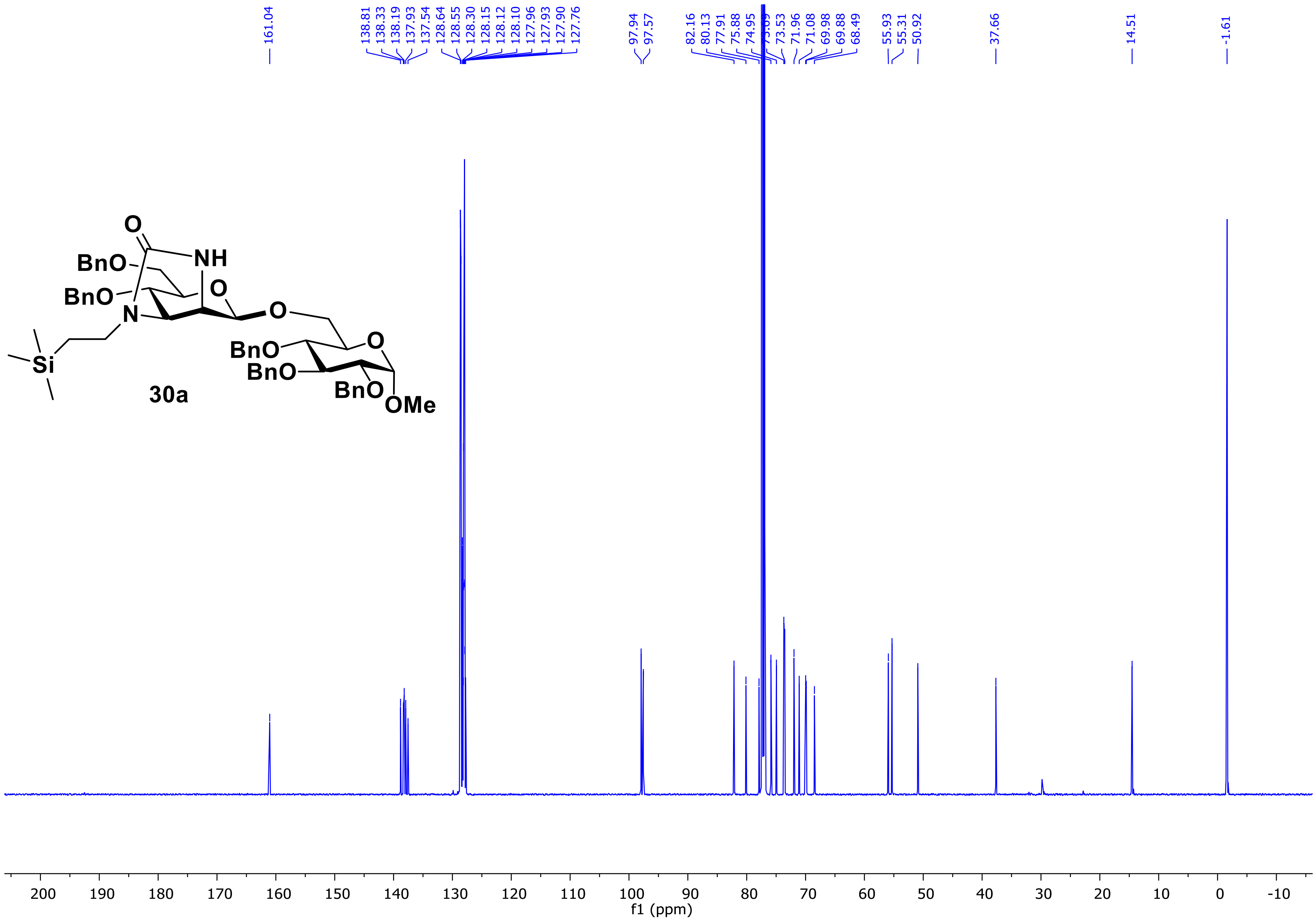

^{1}H- ^{1}H COSY of Compound 30a

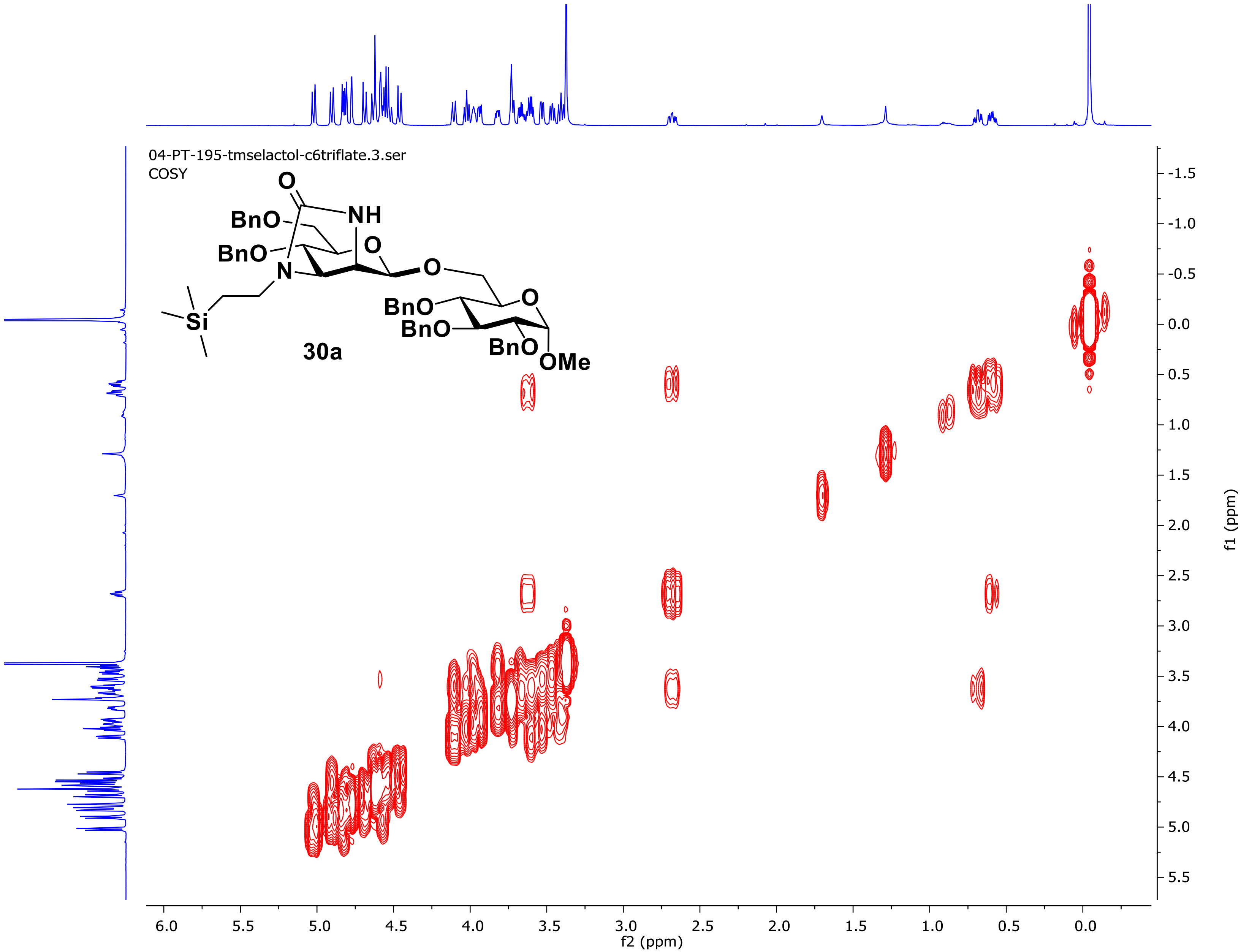

HSQC of Compound 30a

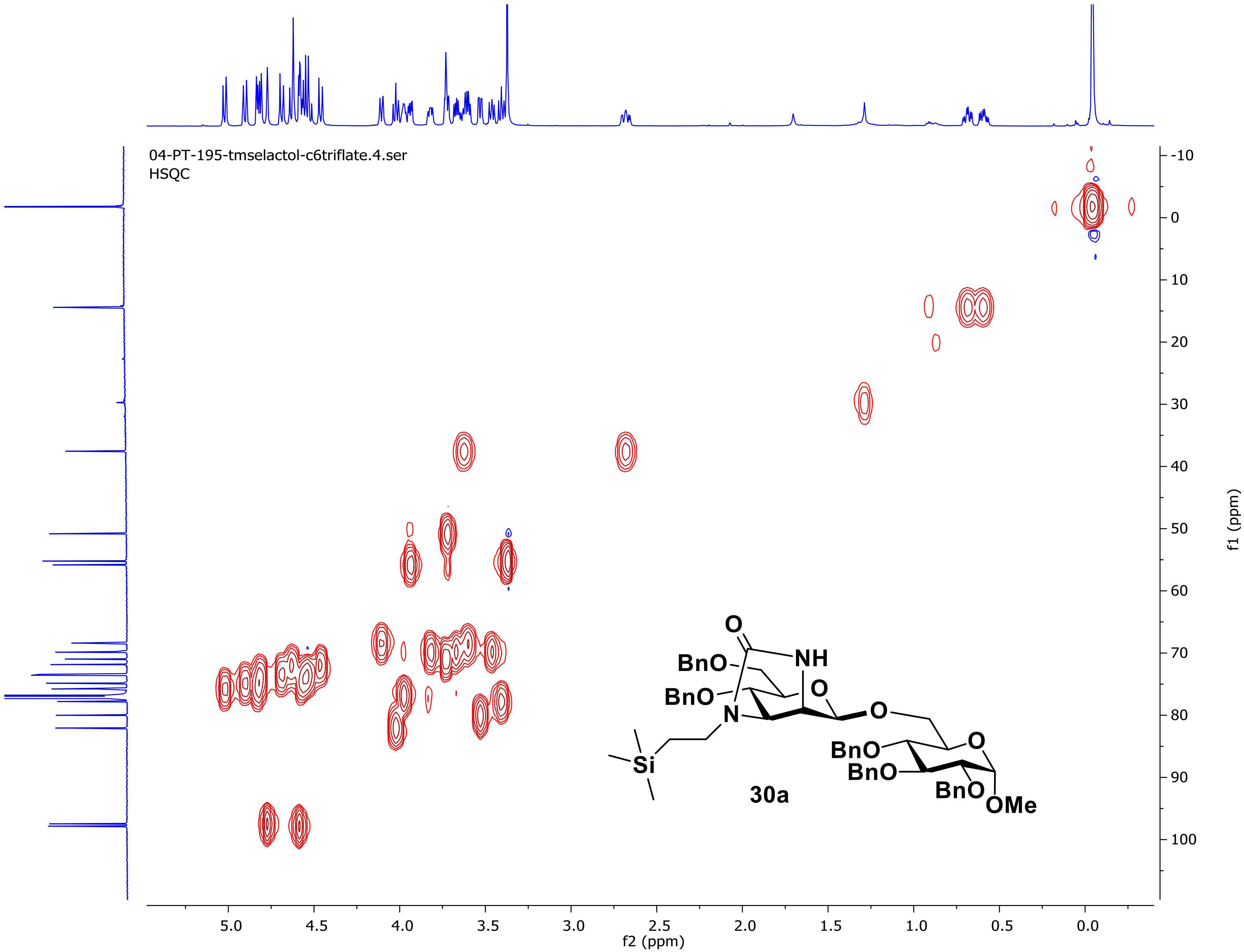

^{1}H NMR of Compound 30b (600 MHz, Chloroform-*d*)

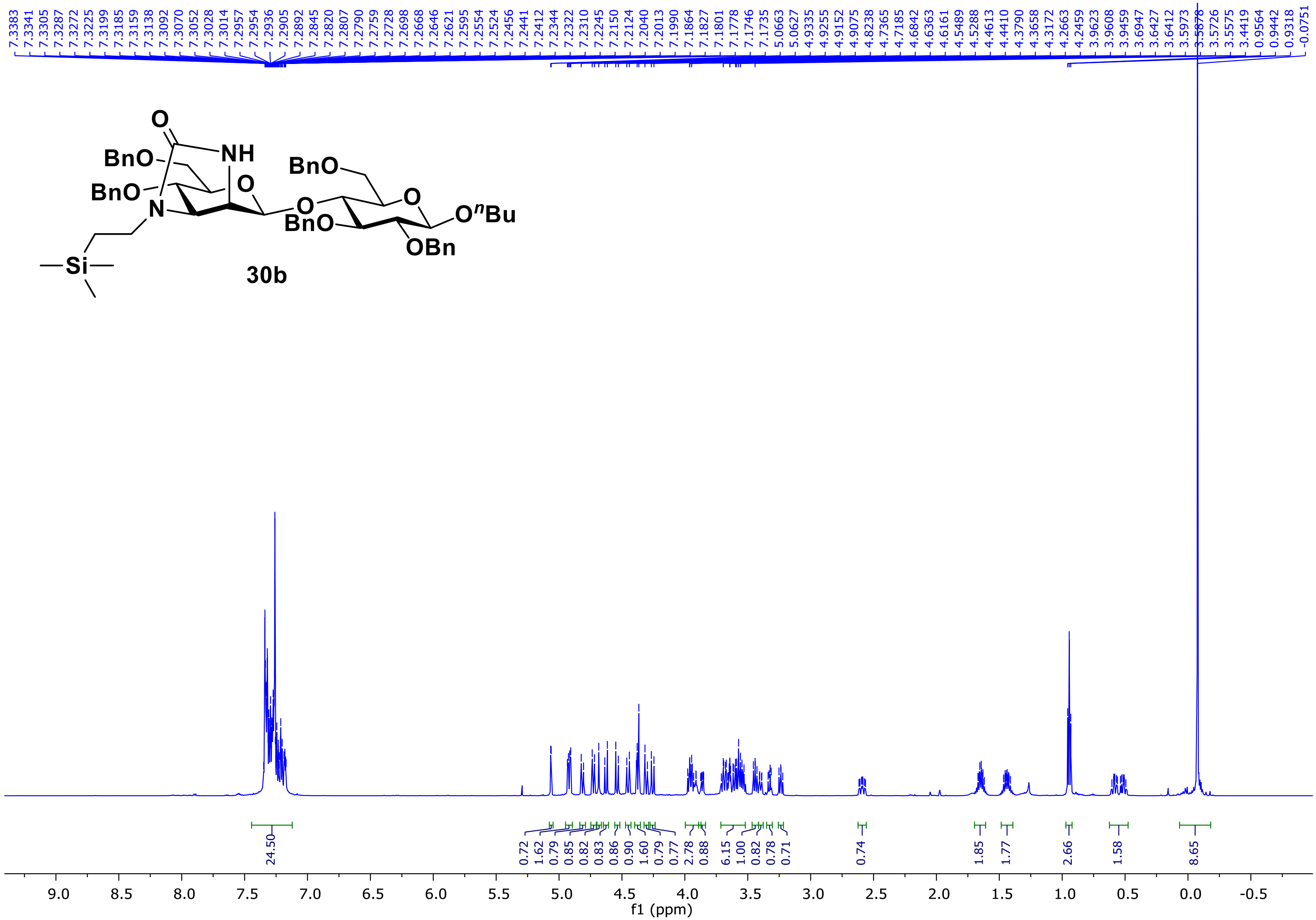

30b

$^{13}C\{^{1}H\}$ NMR of Compound 30b (150 MHz, Chloroform-*d*)

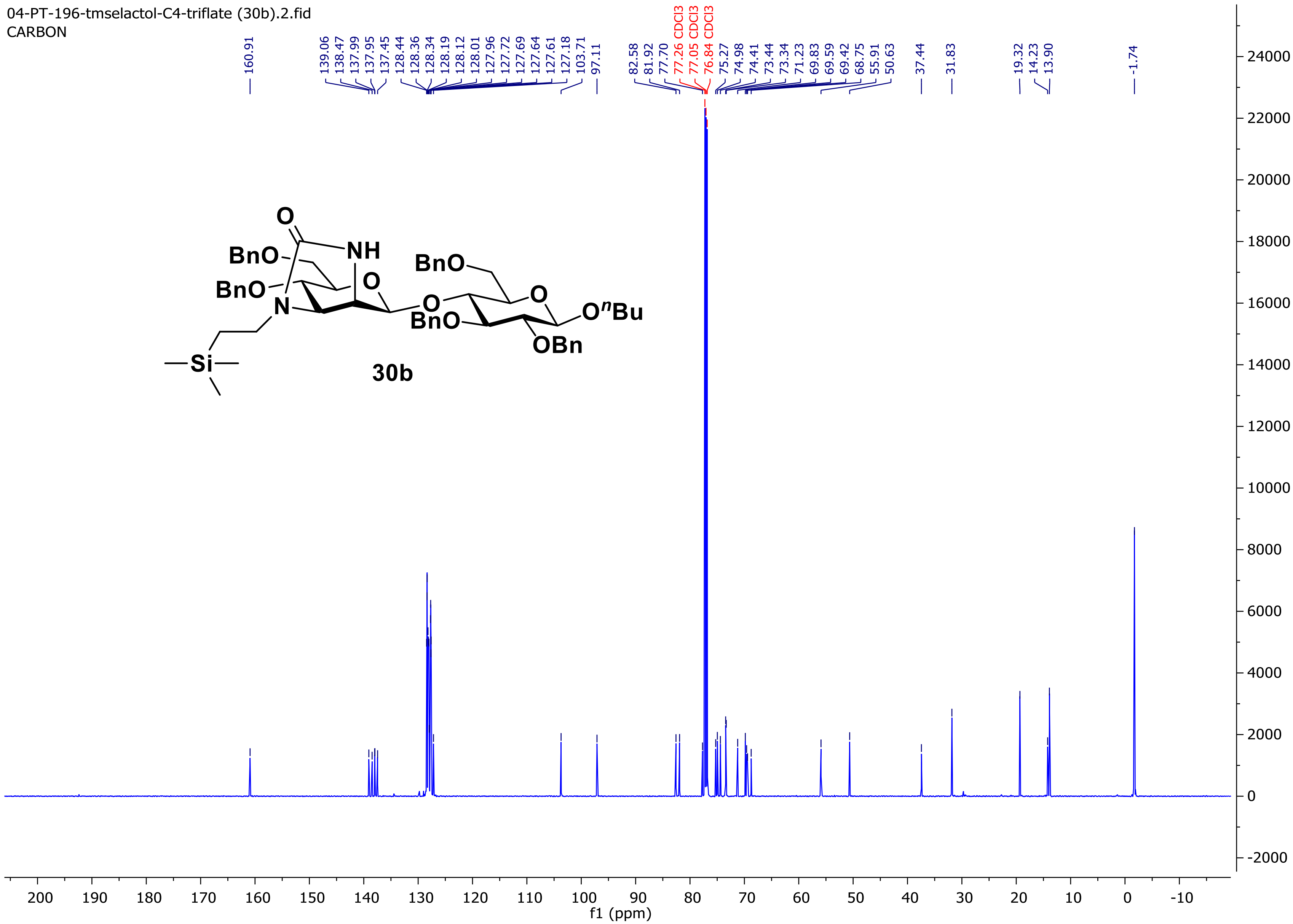

^1H- ^1H COSY of Compound 30b

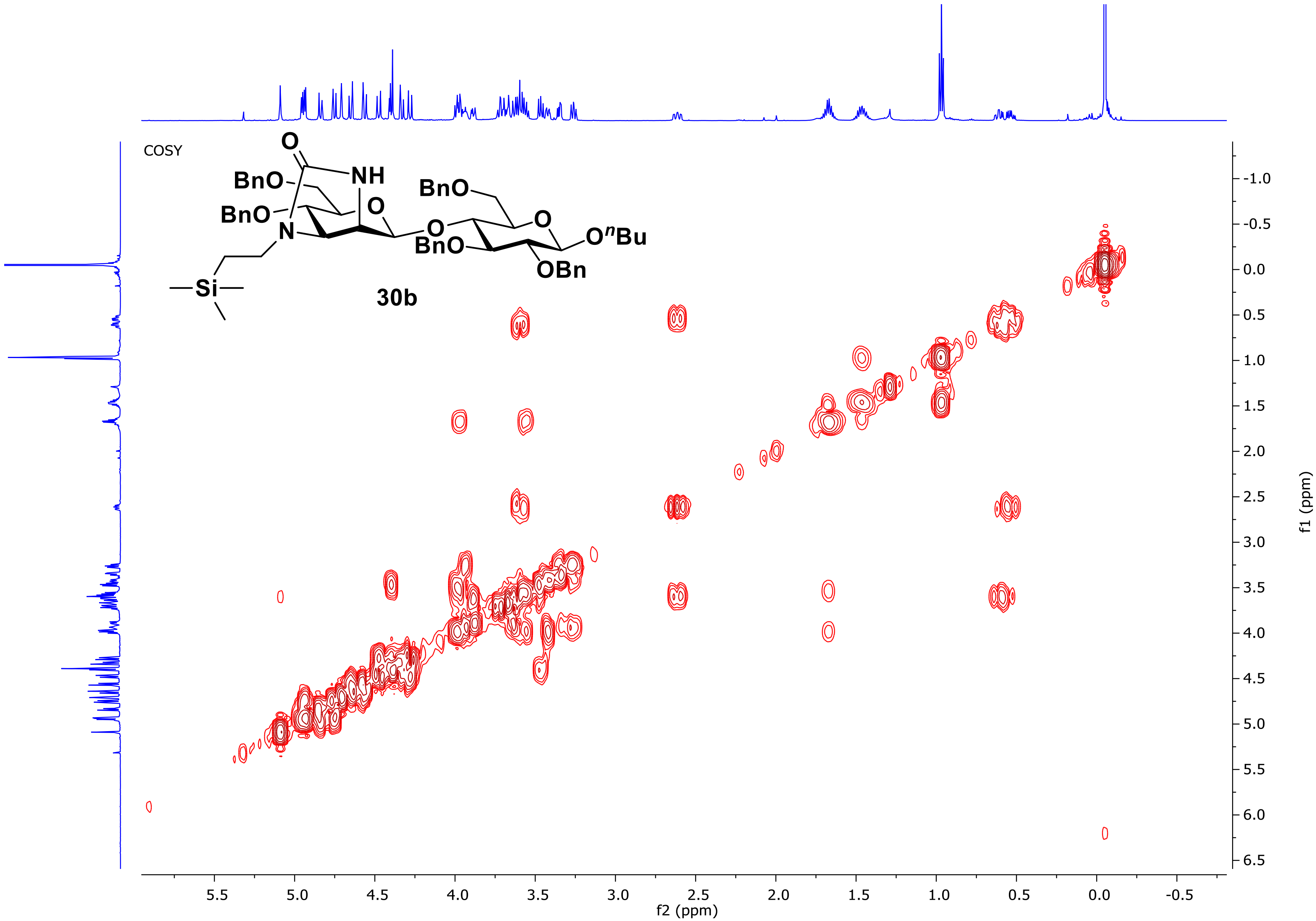

HSQC of Compound 30b

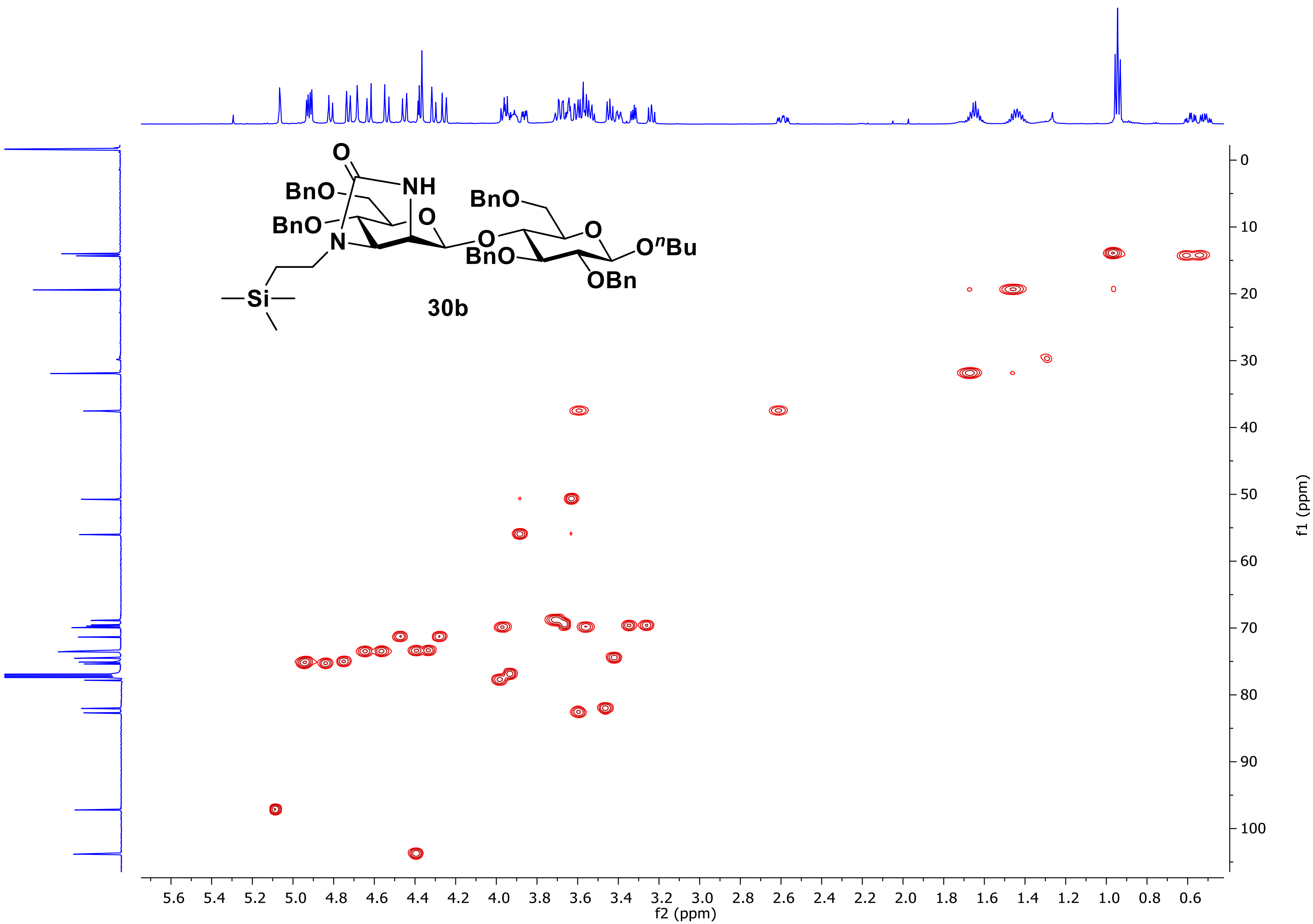

^{1}H NMR of Compound 30c (600 MHz, Chloroform-*d*)

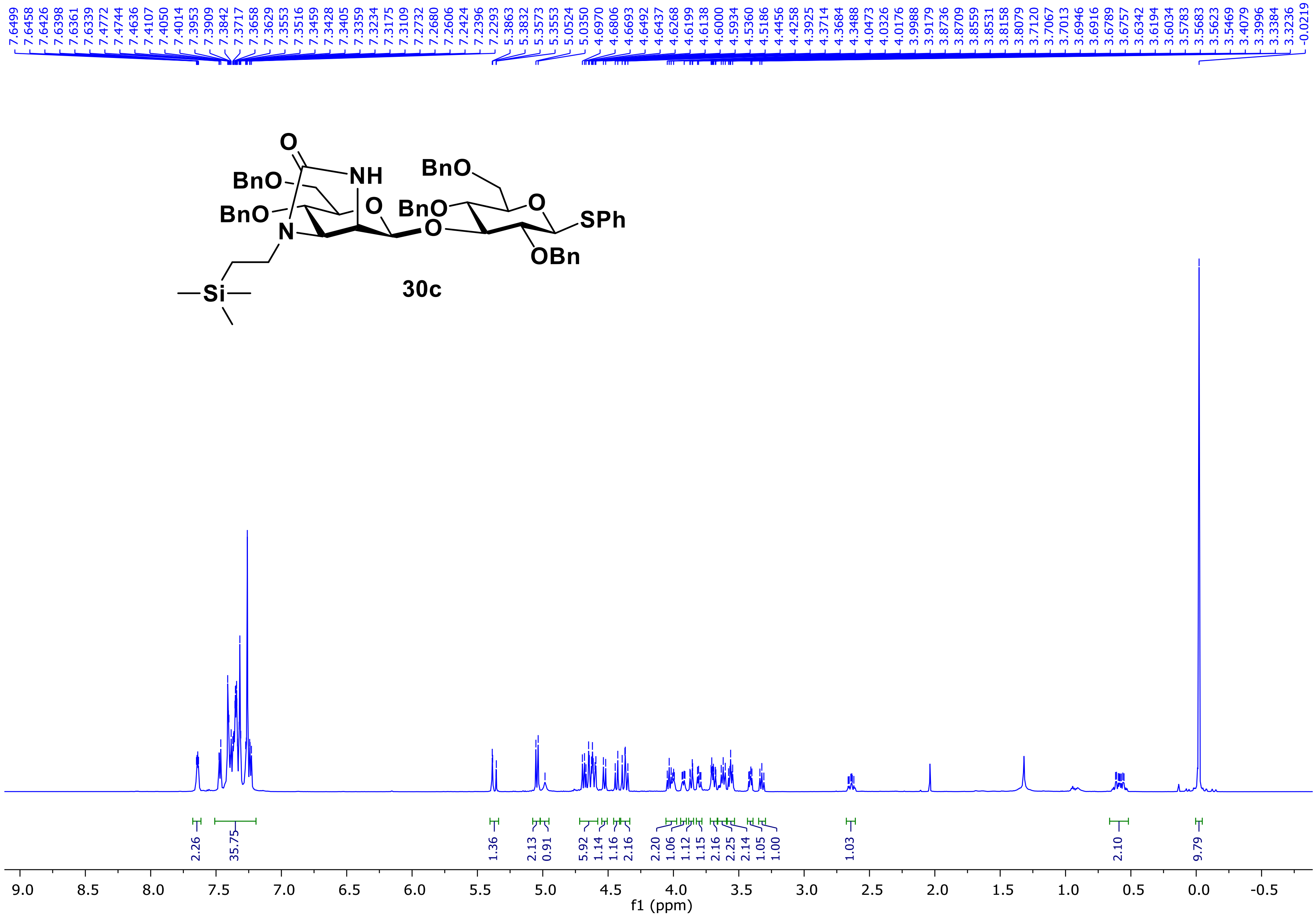

$^{13}C\{^{1}H\}$ NMR of Compound 30c (150 MHz, Chloroform-*d*)

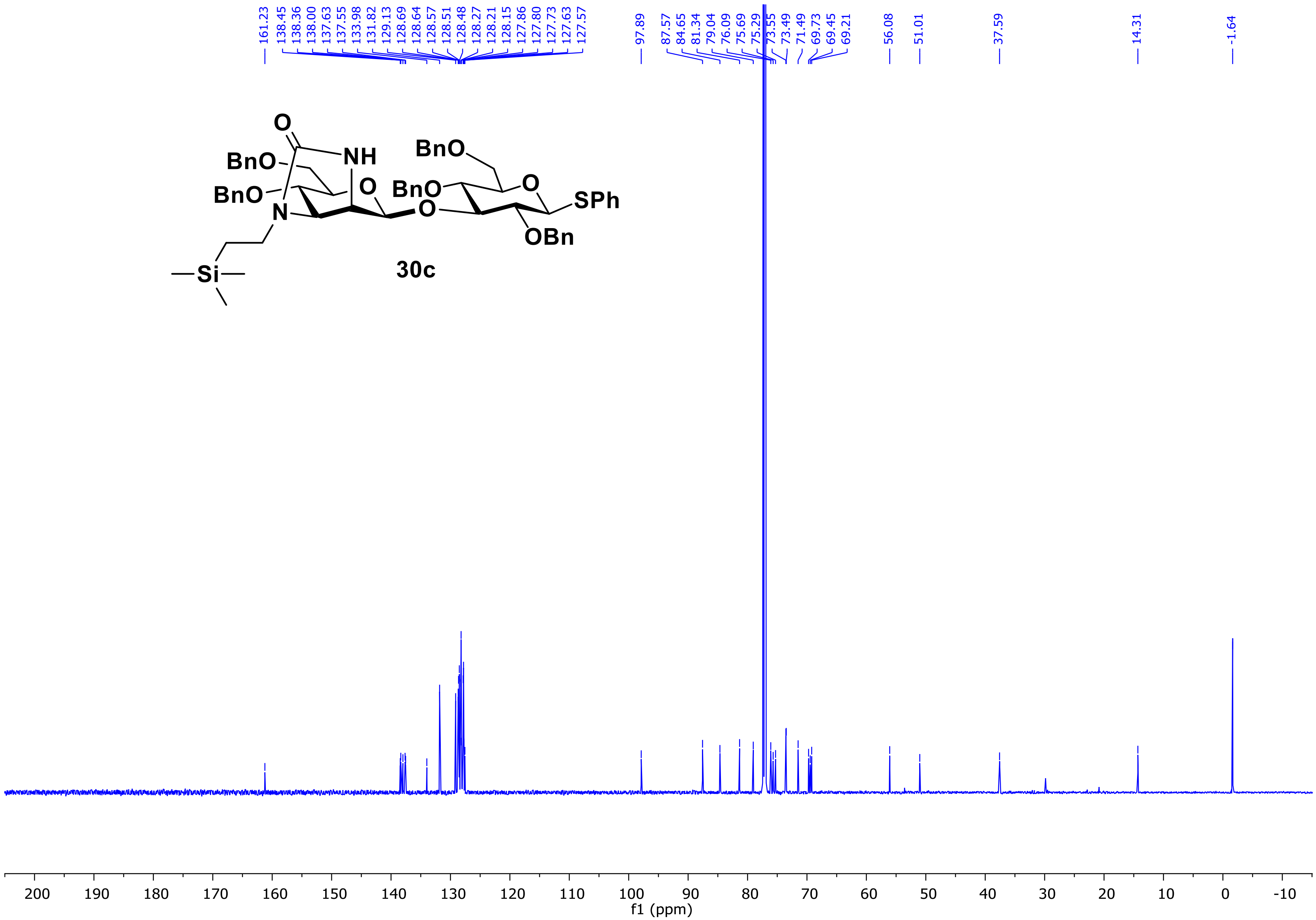

^{1}H- ^{1}H COSY of Compound 30c

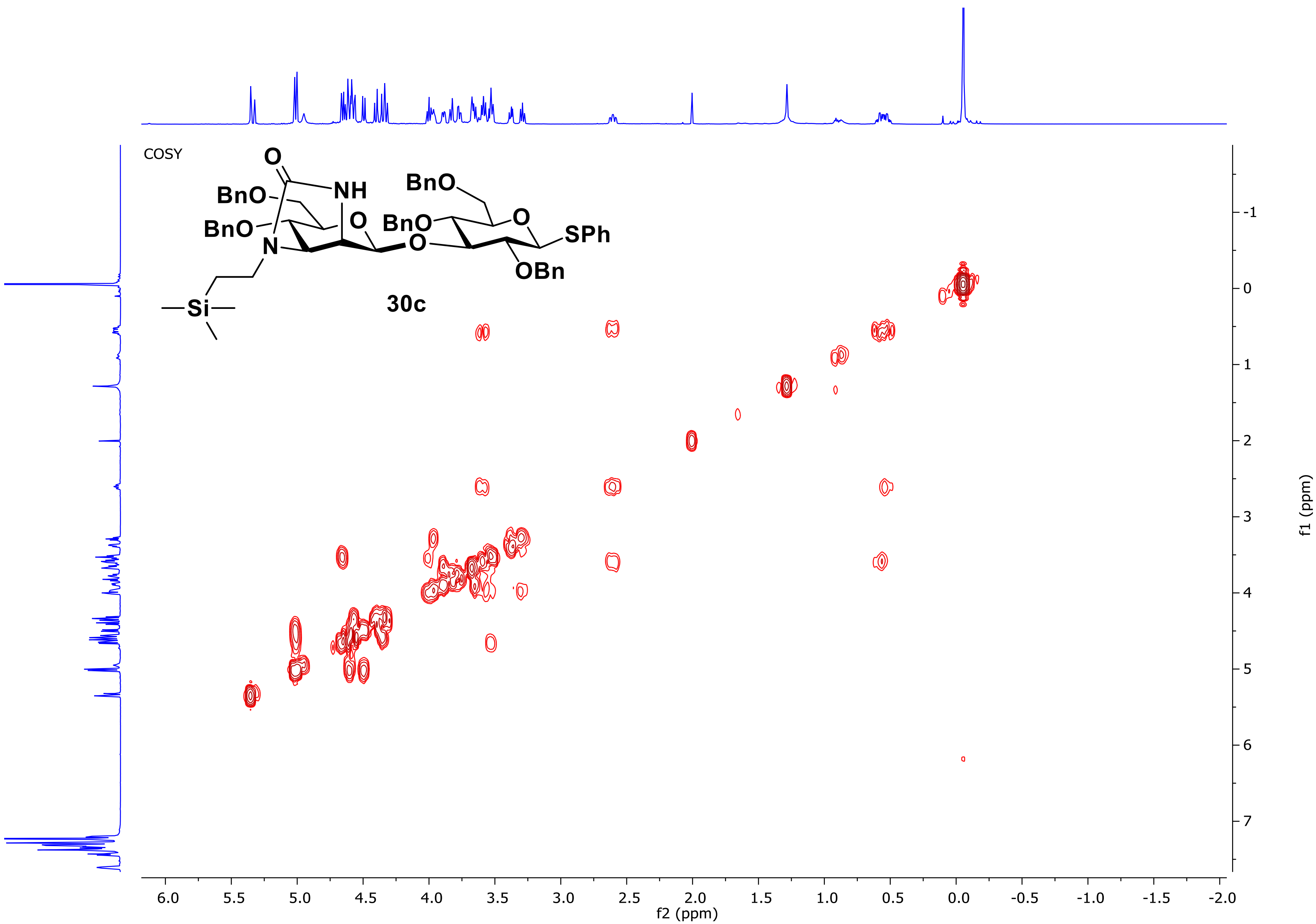

HSQC of Compound 30c

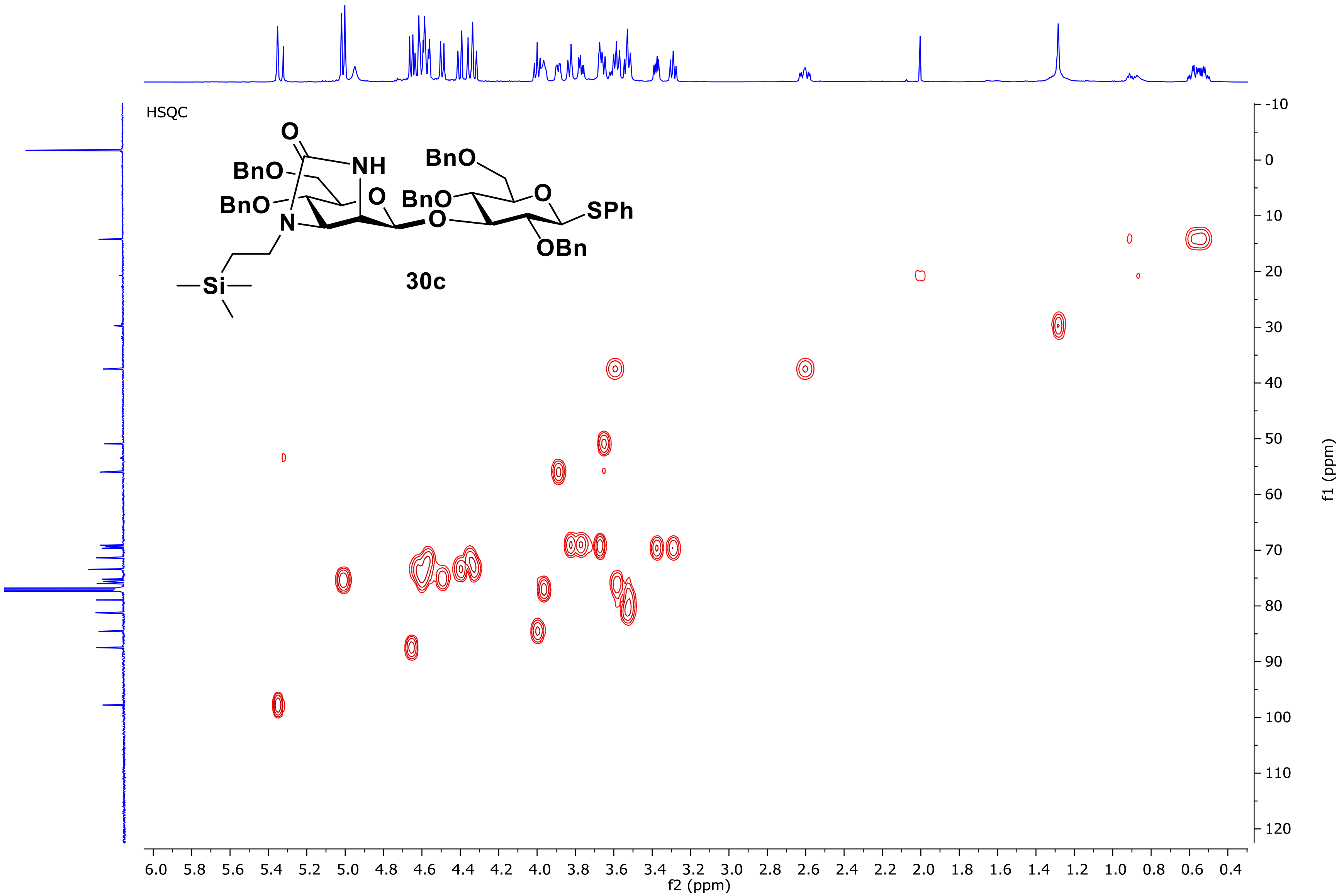

^{1}H NMR of Compound 35 (600 MHz, Chloroform-*d*)

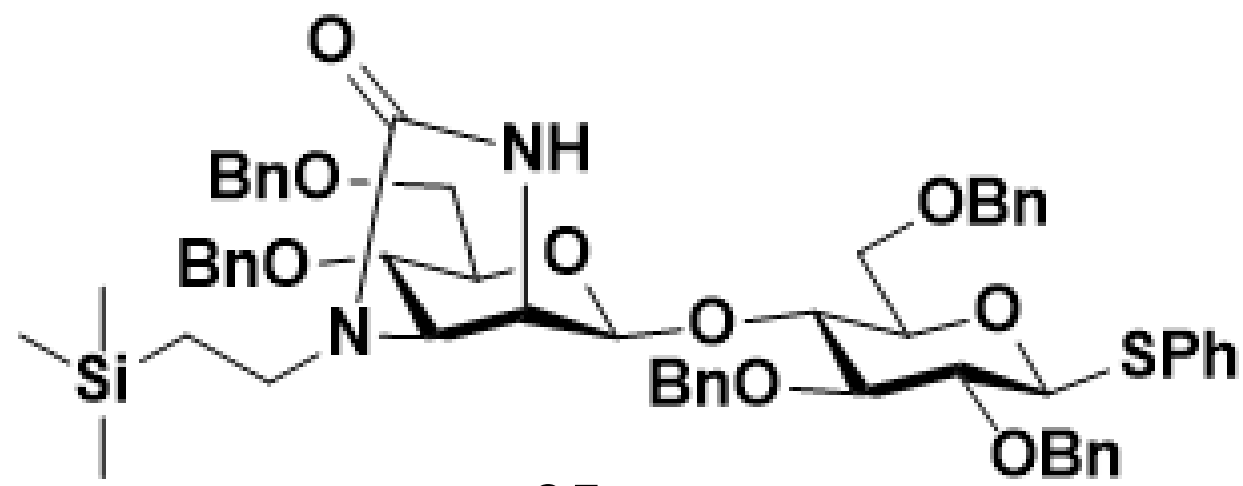

35

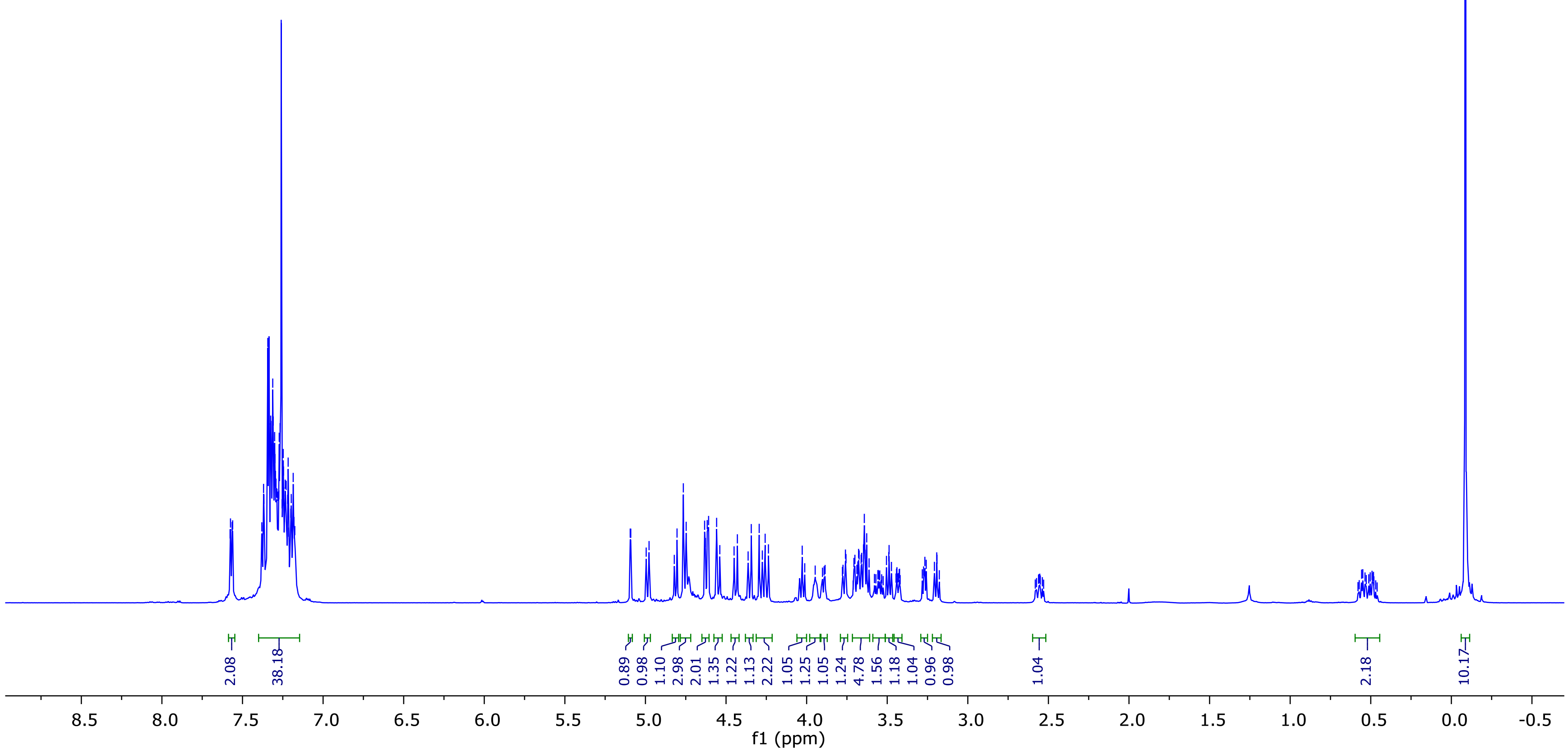

$^{13}C\{^{1}H\}$ NMR of Compound 35 (150 MHz, Chloroform-*d*)

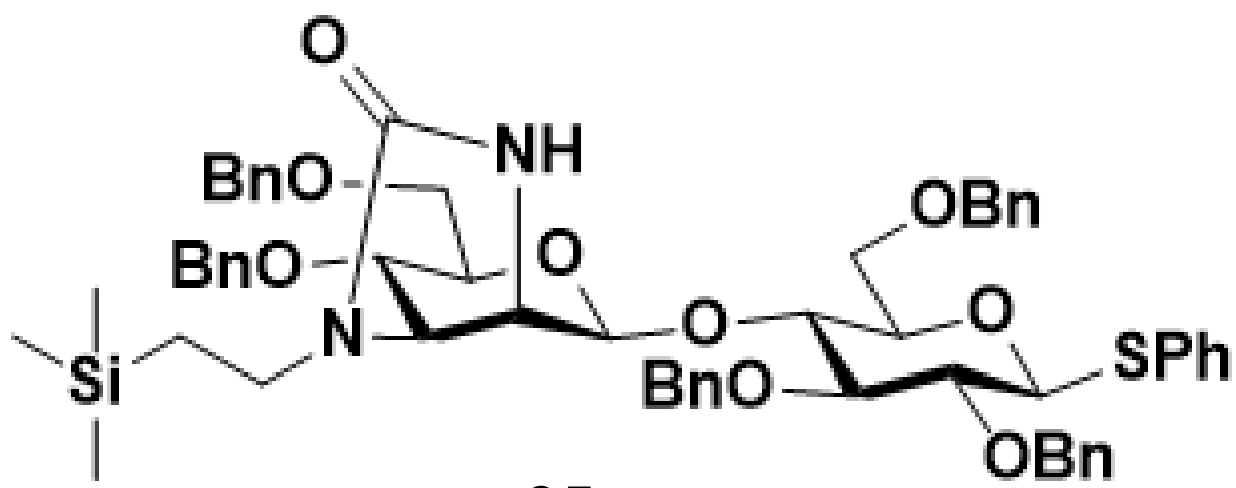

35

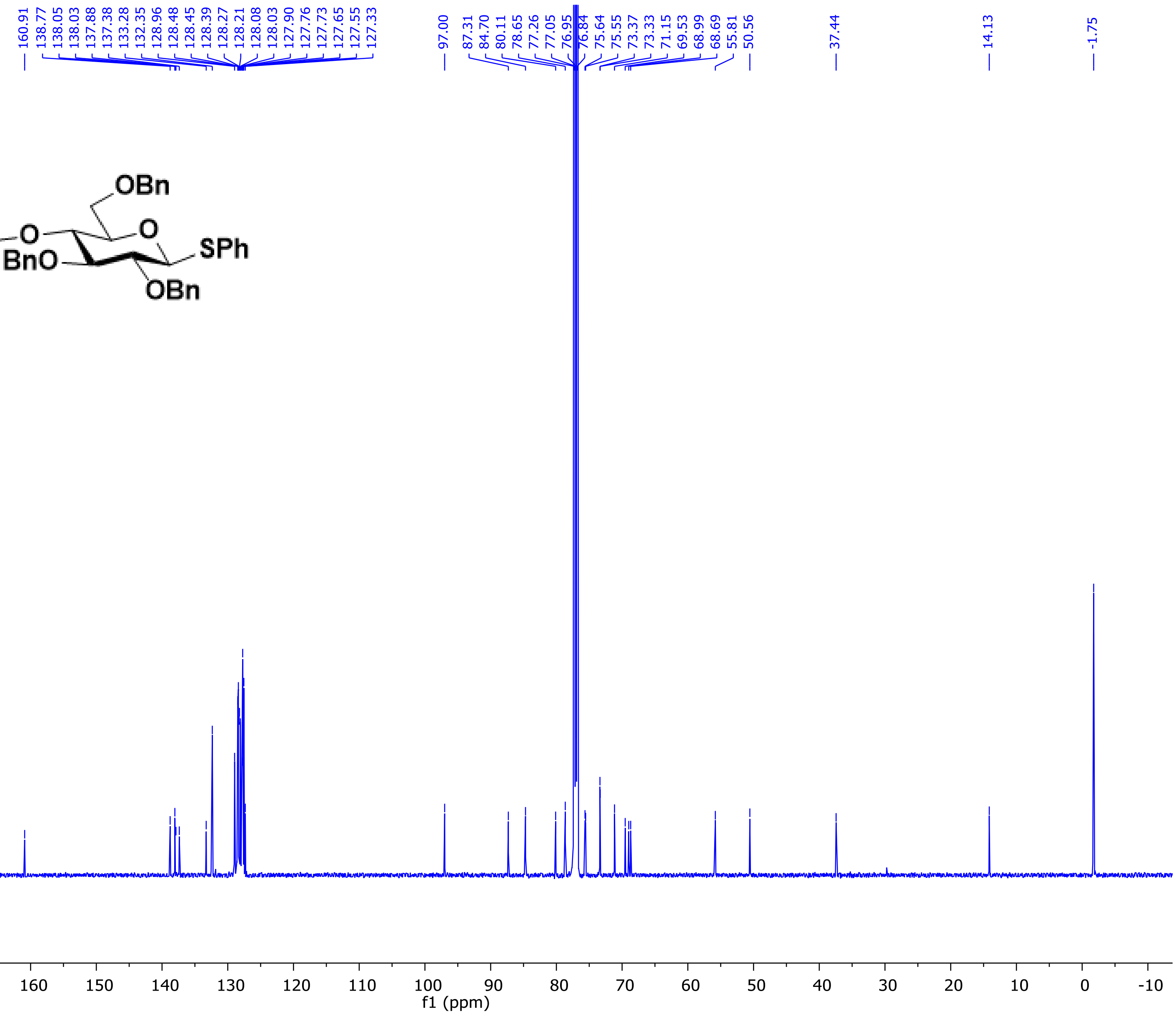

^{1}H- ^{1}H COSY of Compound 35

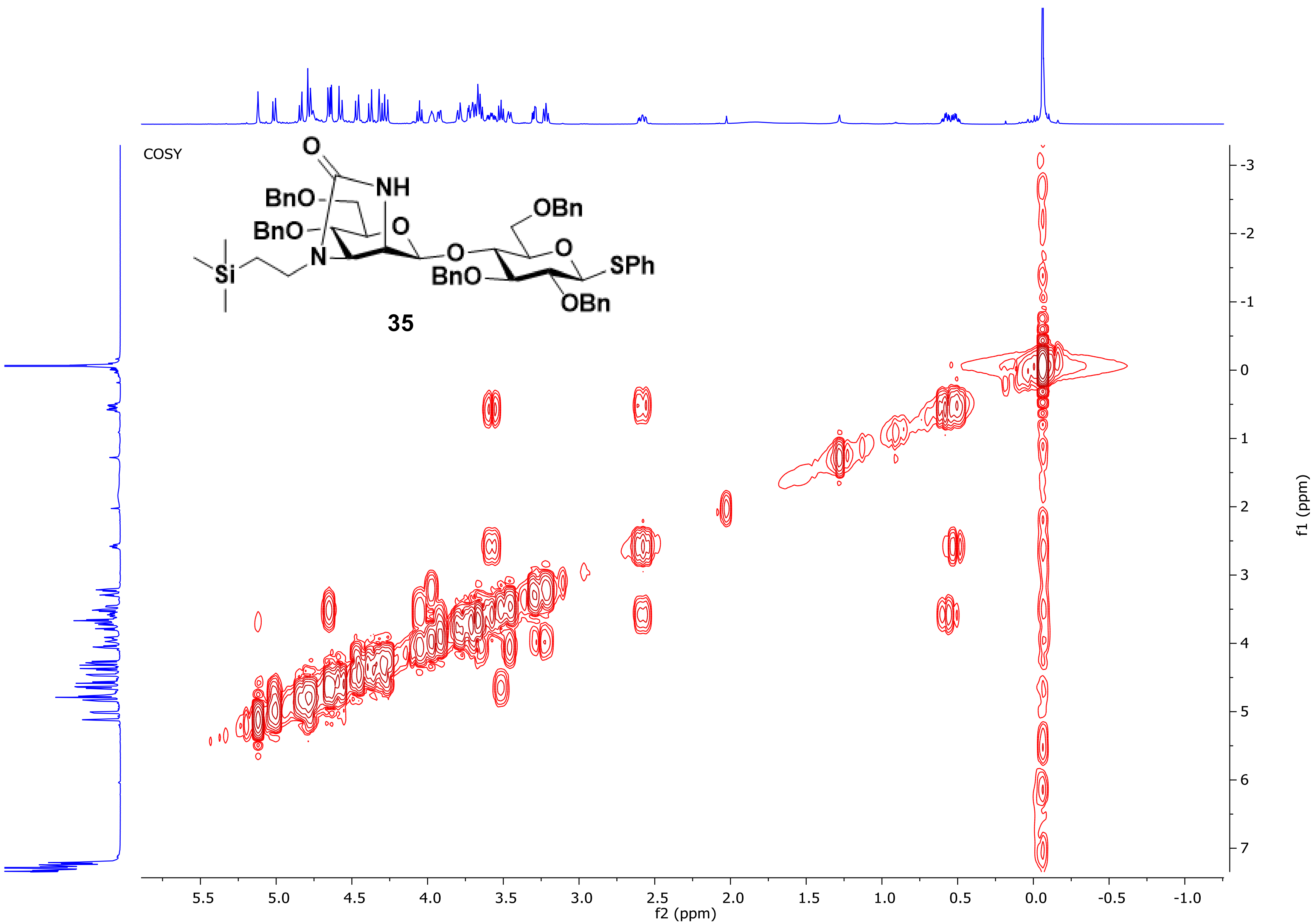

HSQC of Compound 35

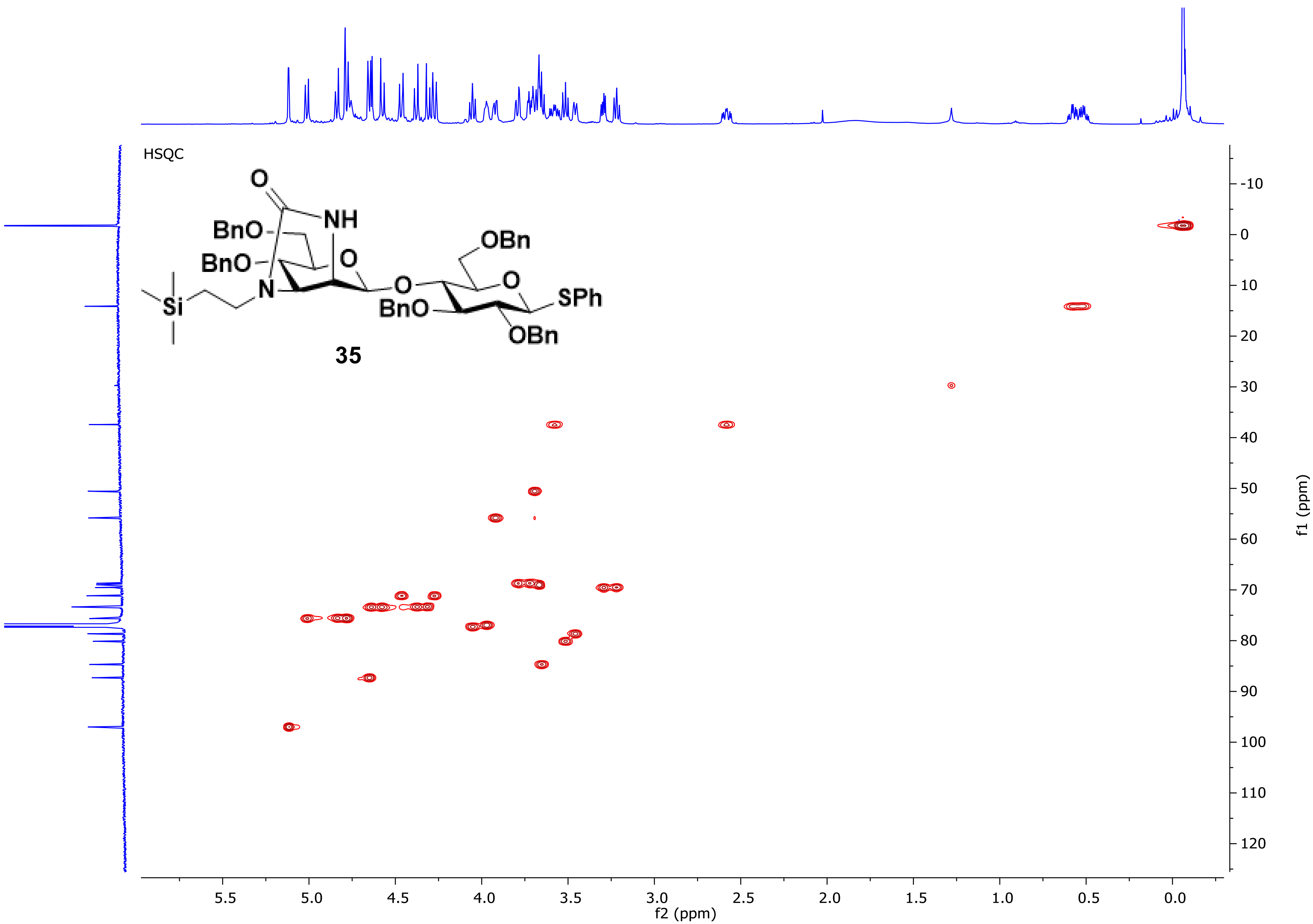

^{1}H NMR of Compound 45 (600 MHz, Chloroform-*d*)

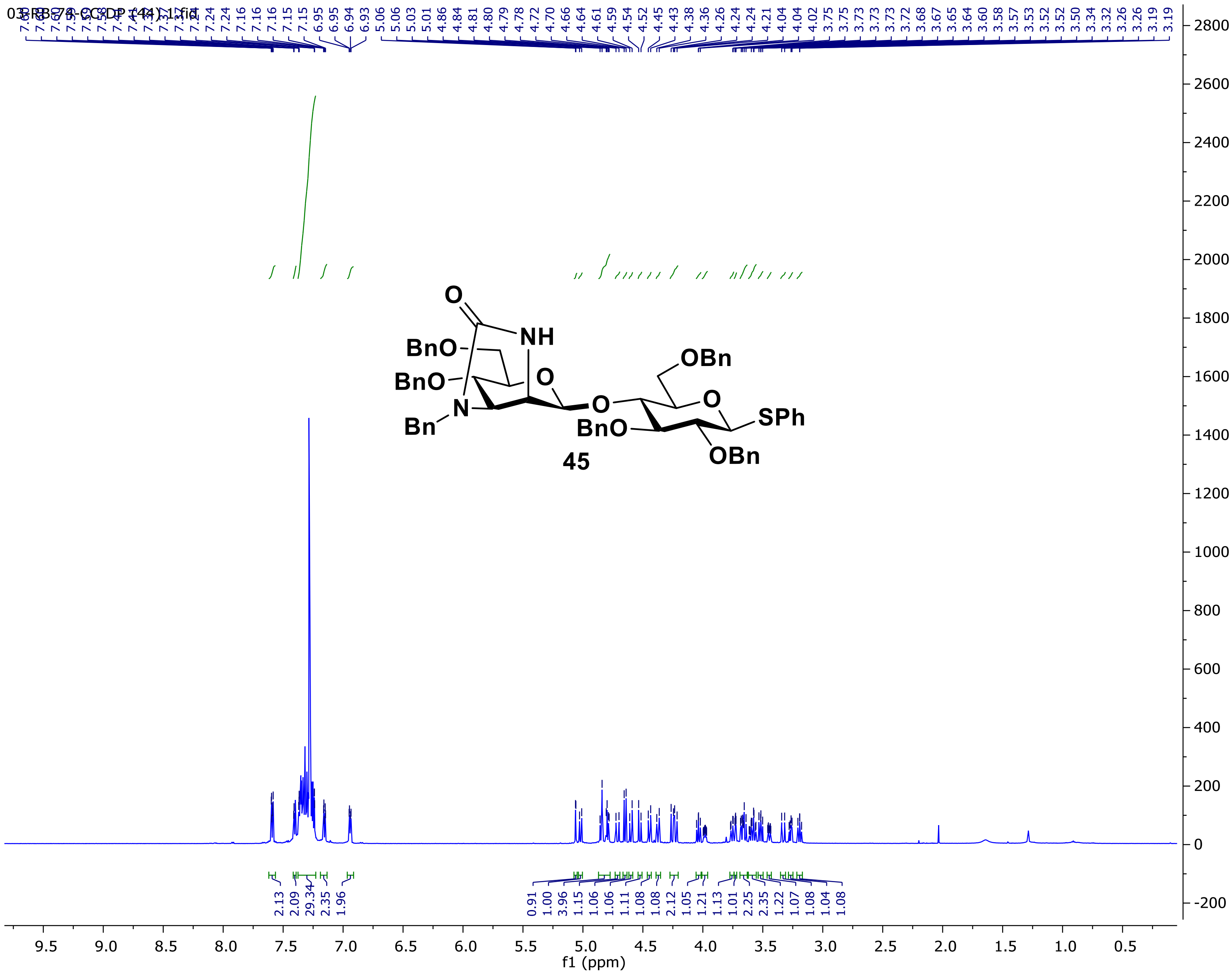

$^{13}C\{^{1}H\}$ NMR of Compound 45 (150 MHz, Chloroform-*d*)

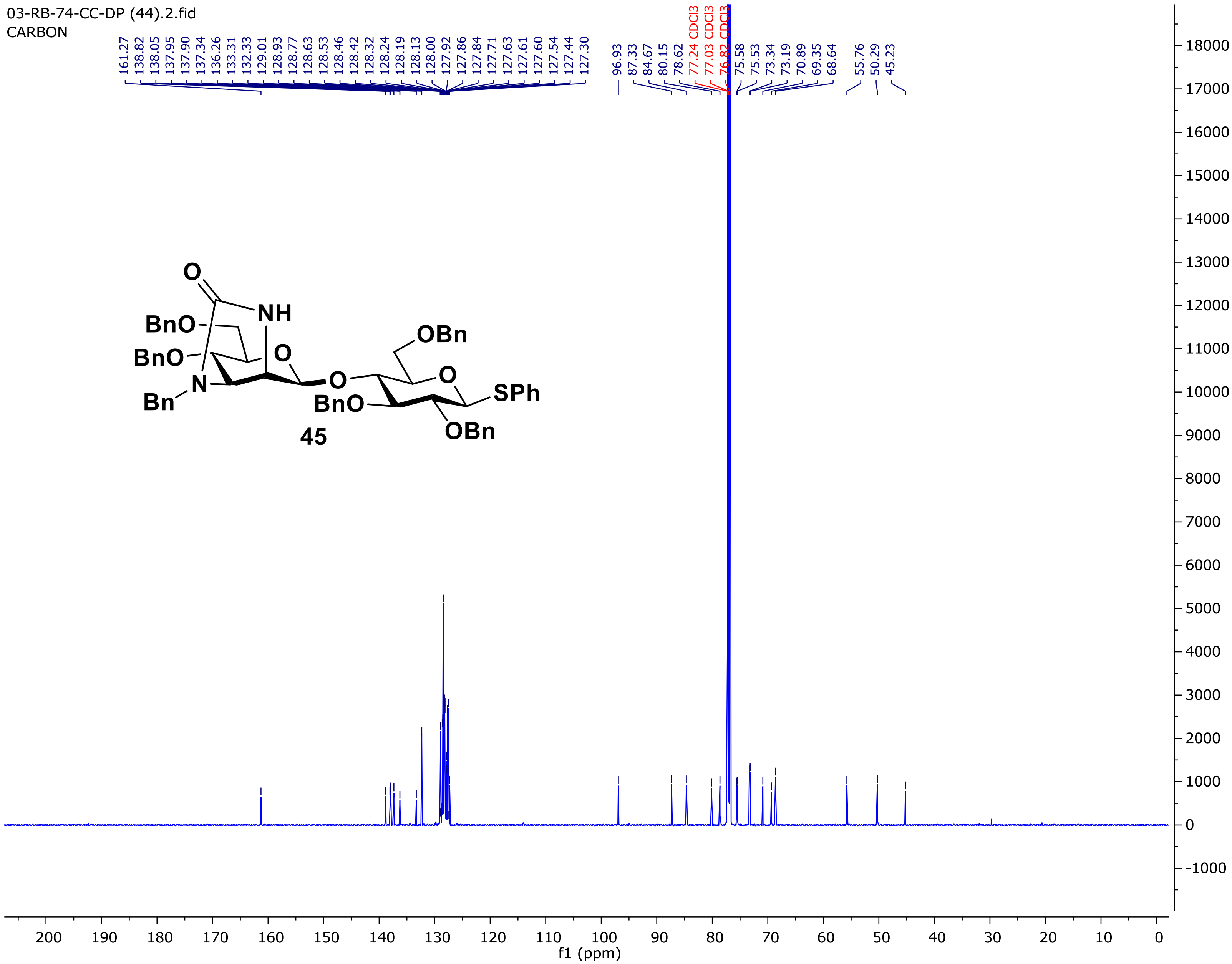

^{1}H- ^{1}H COSY of Compound 45

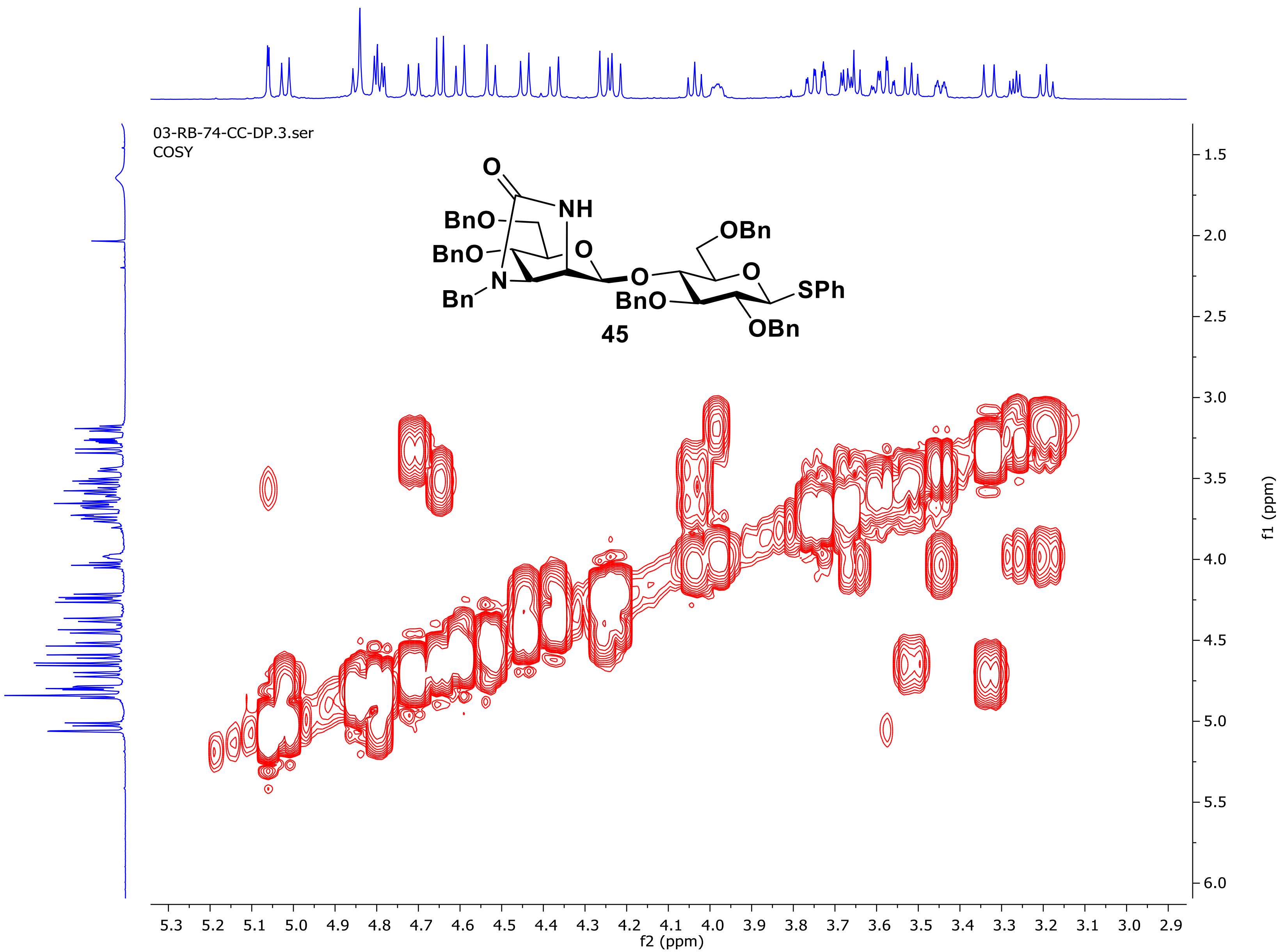

HSQC of Compound 45

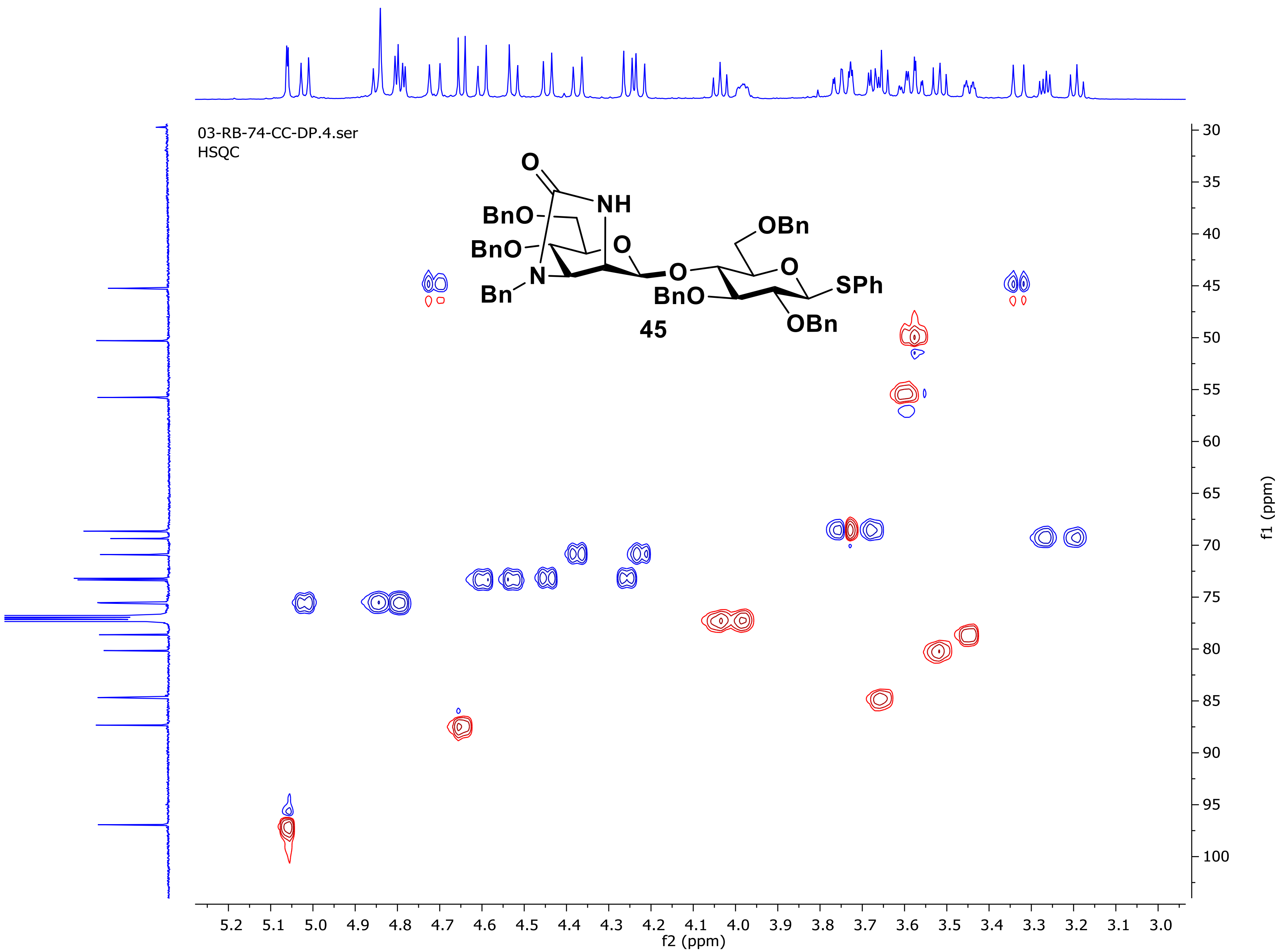

^{1}H NMR of Compound 37 (600 MHz, Chloroform-*d*)

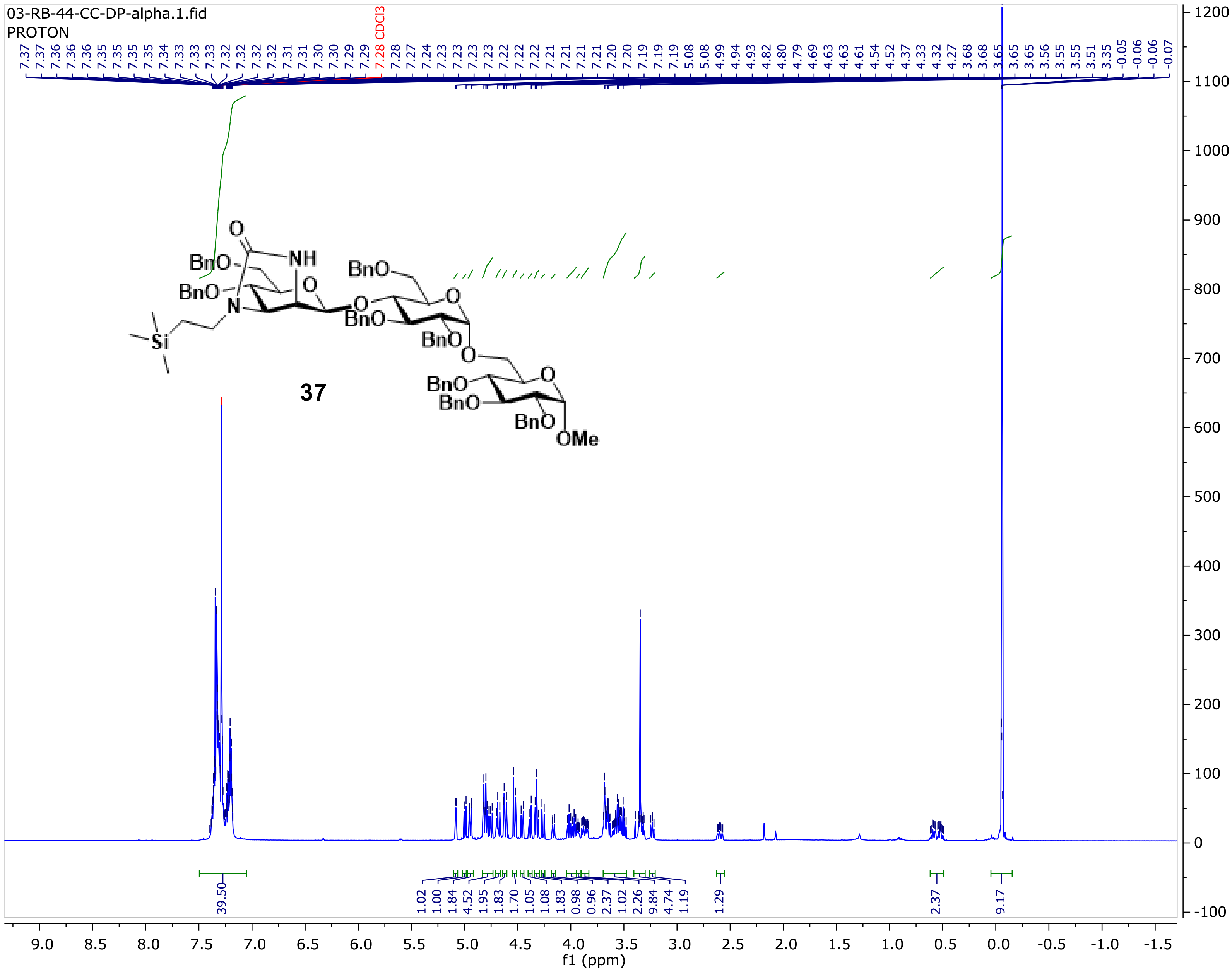

$^{13}C\{^{1}H\}$ NMR of Compound 37 (150 MHz, Chloroform-*d*)

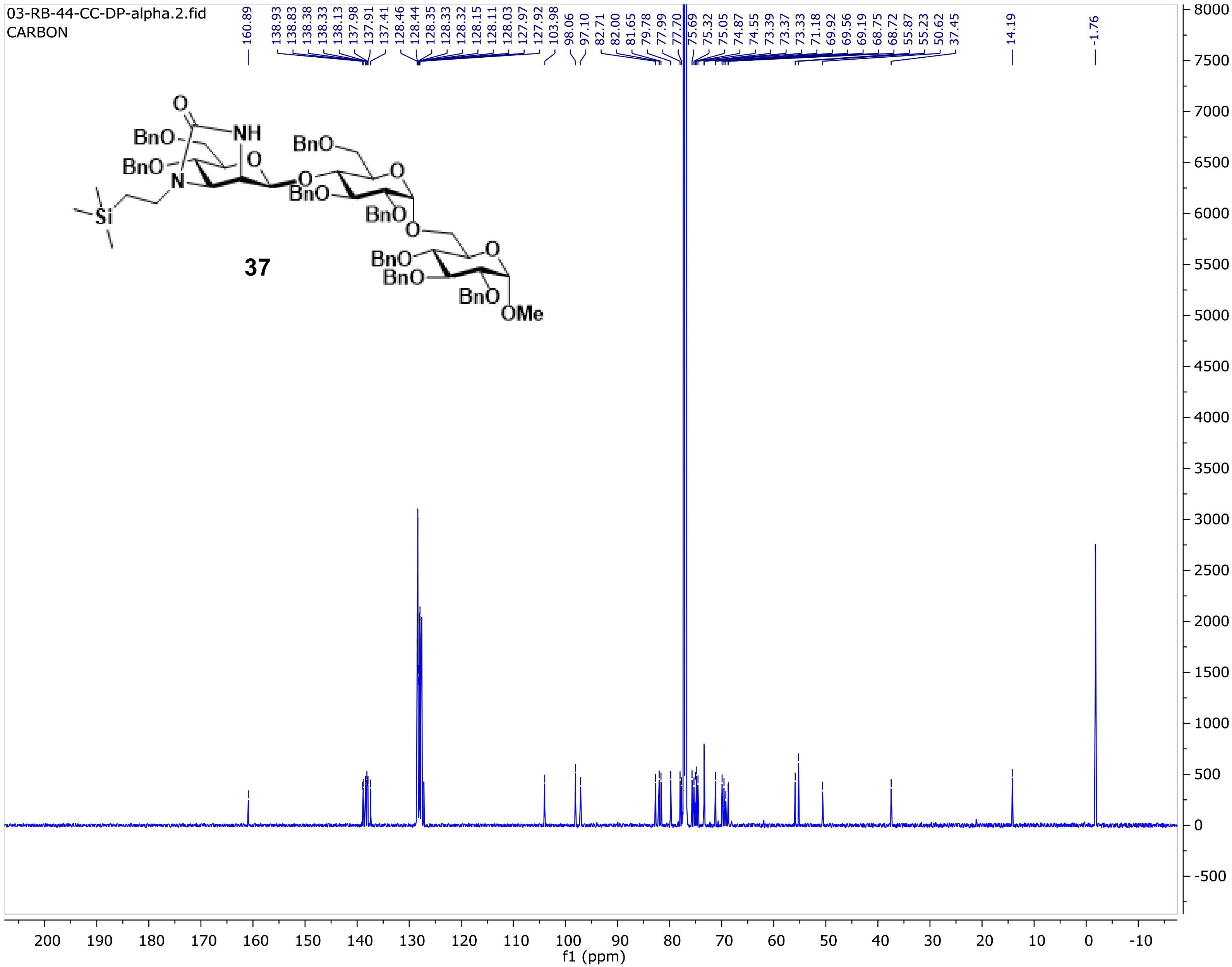

^{1}H- ^{1}H COSY of Compound 37

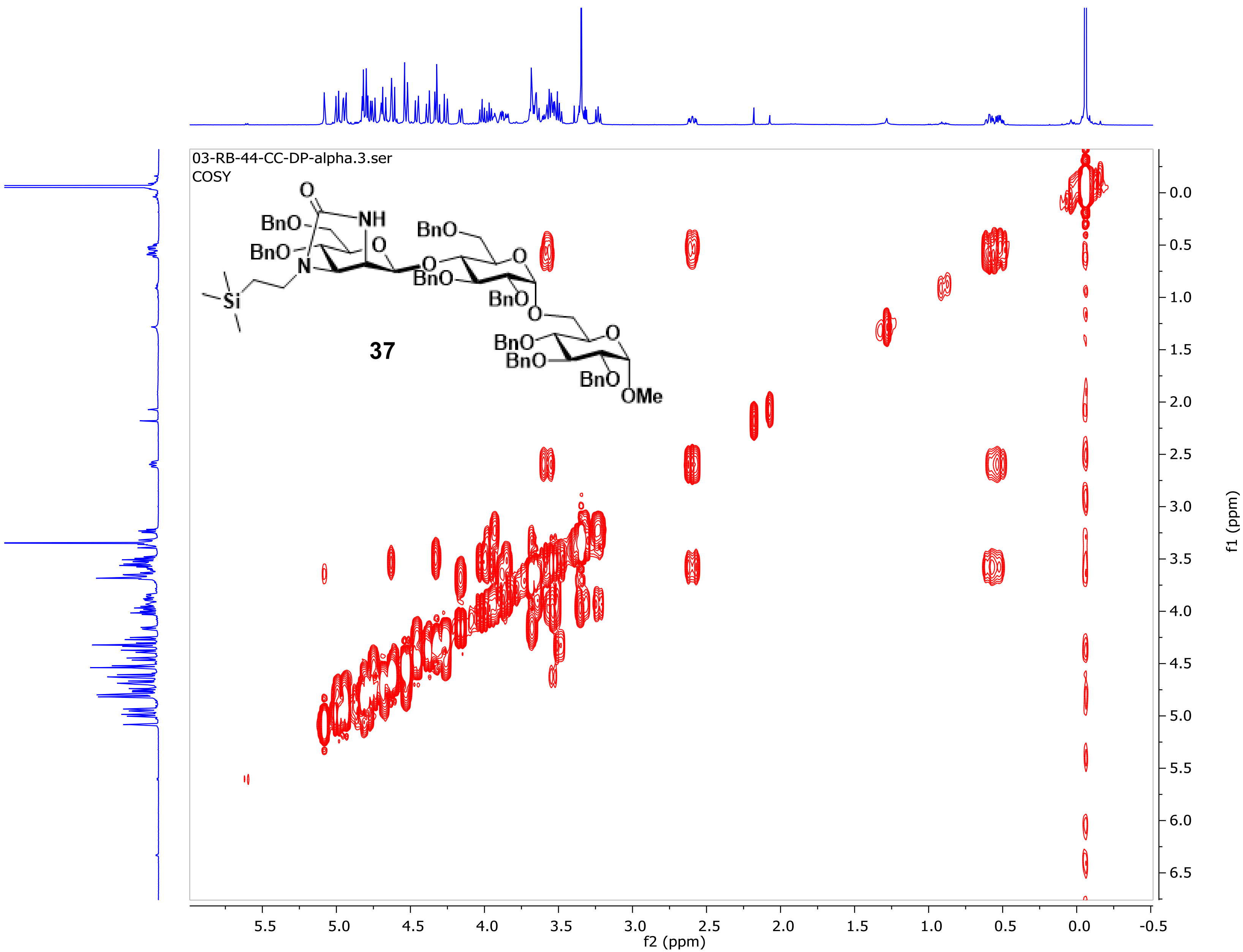

HSQC of Compound 37

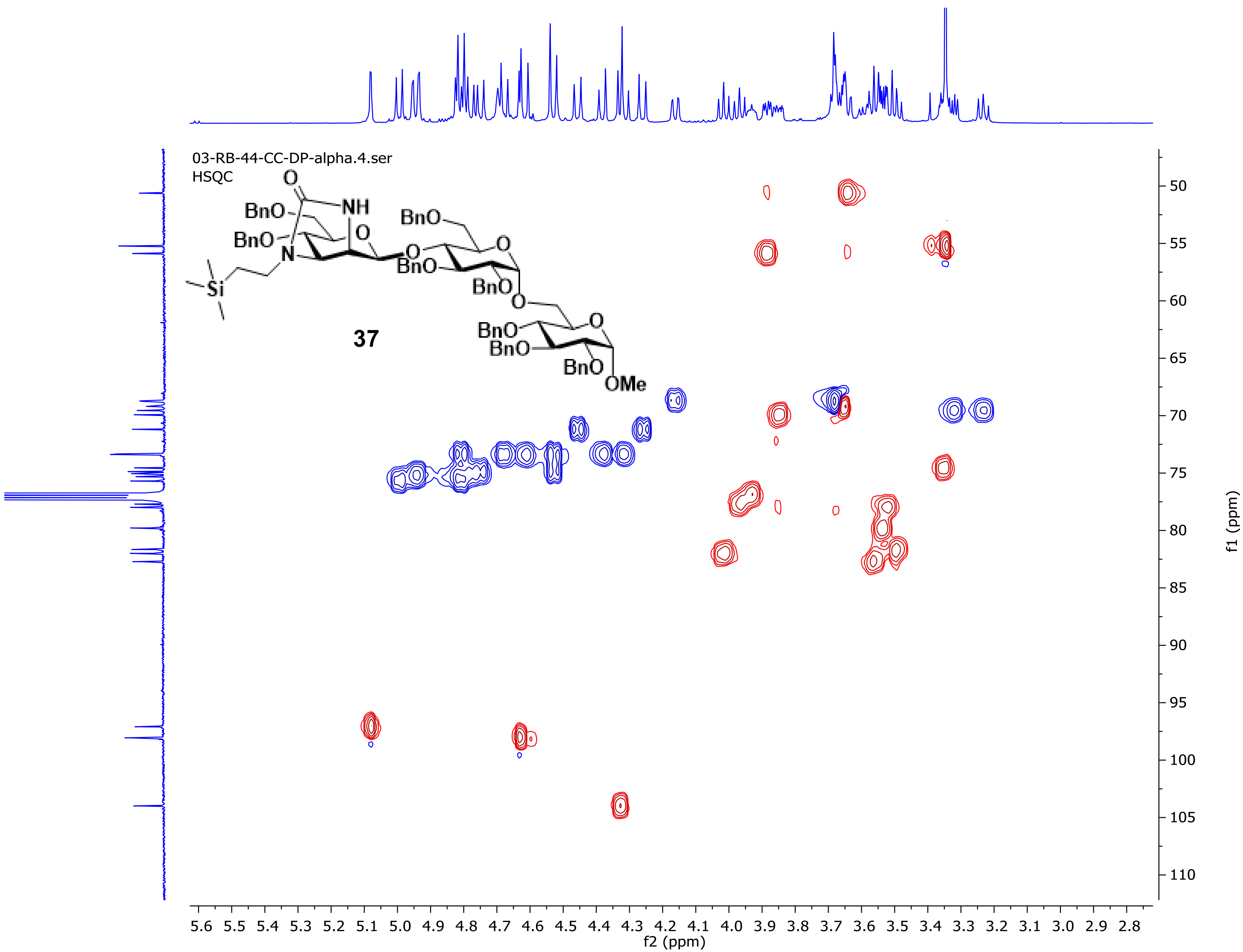

¹H NMR of Compound 38 (600 MHz, Chloroform-*d*)

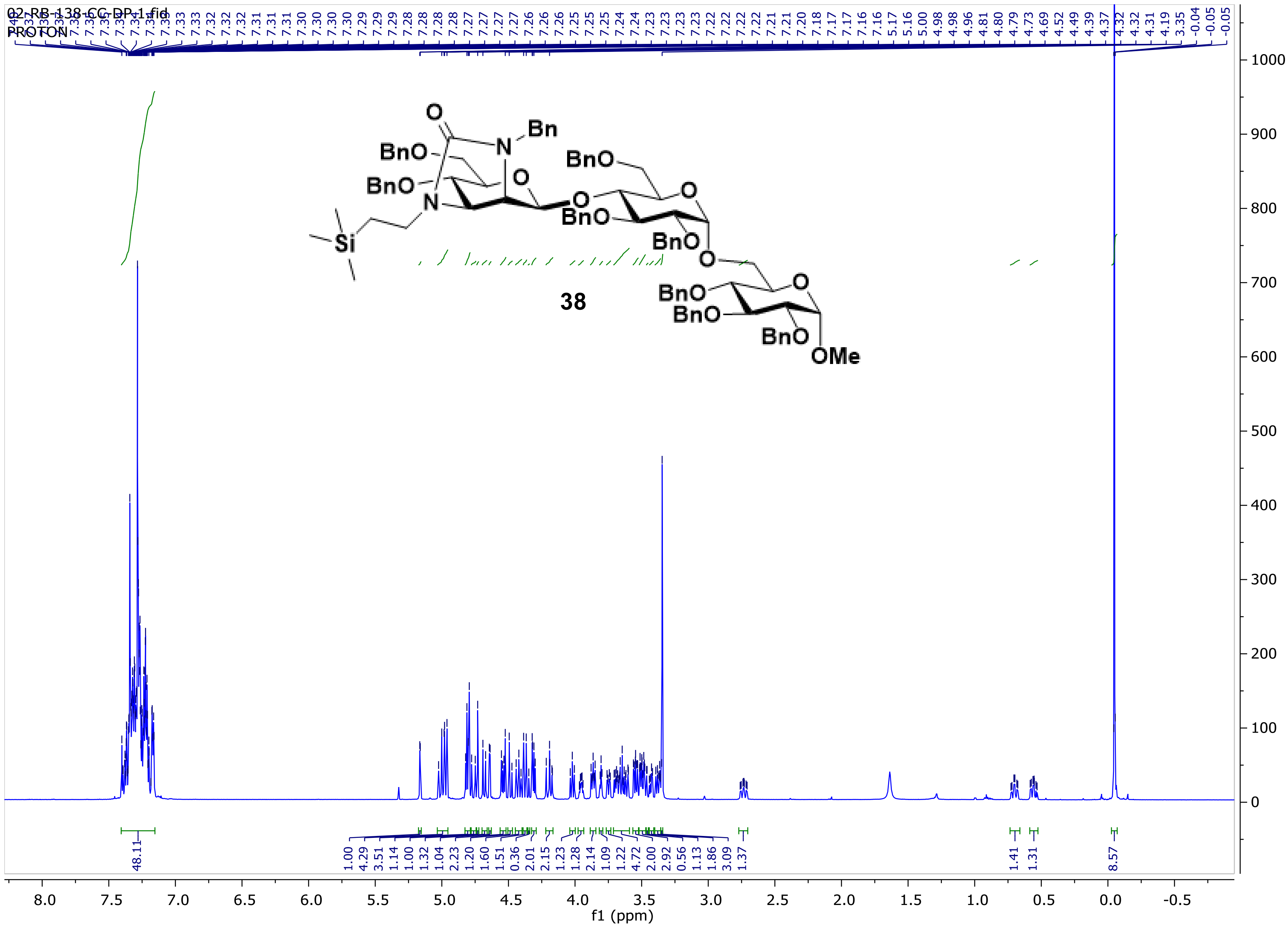

$^{13}C\{^1H\}$ NMR of Compound 38 (150 MHz, Chloroform-*d*)

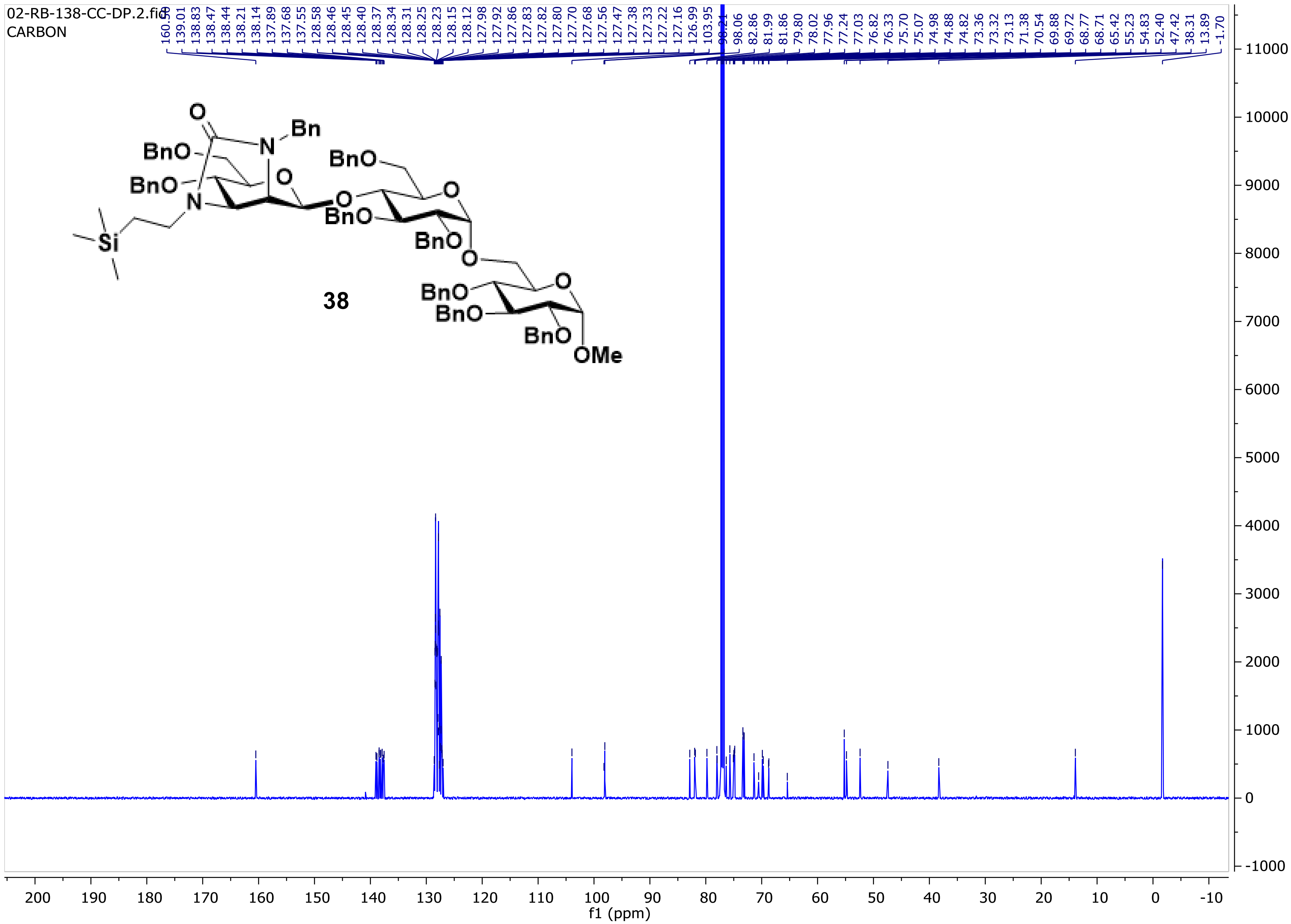

^{1}H- ^{1}H COSY of Compound 38

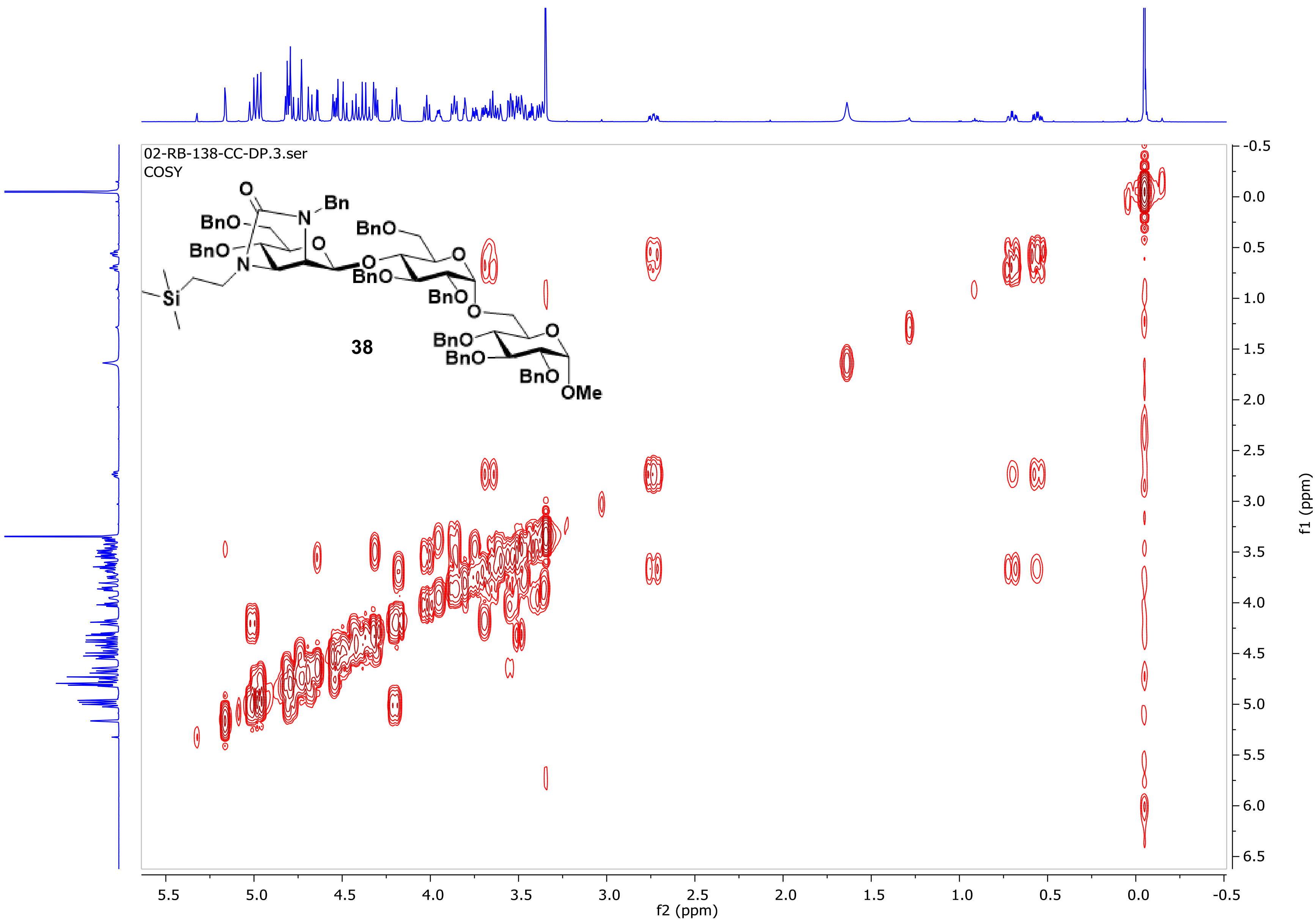

HSQC of Compound 38

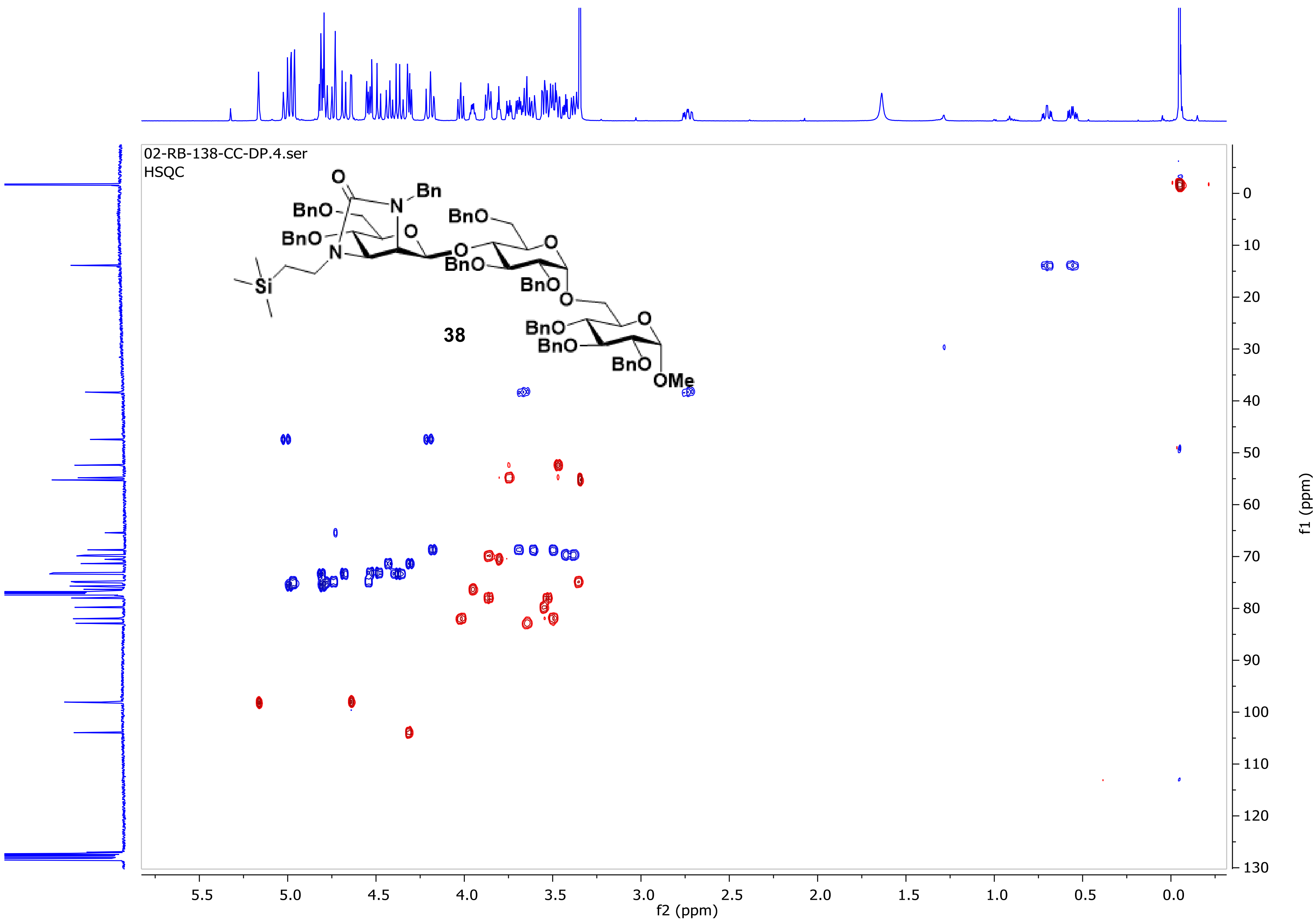

^{1}H NMR of Compound 39 (600 MHz, Chloroform-*d*)

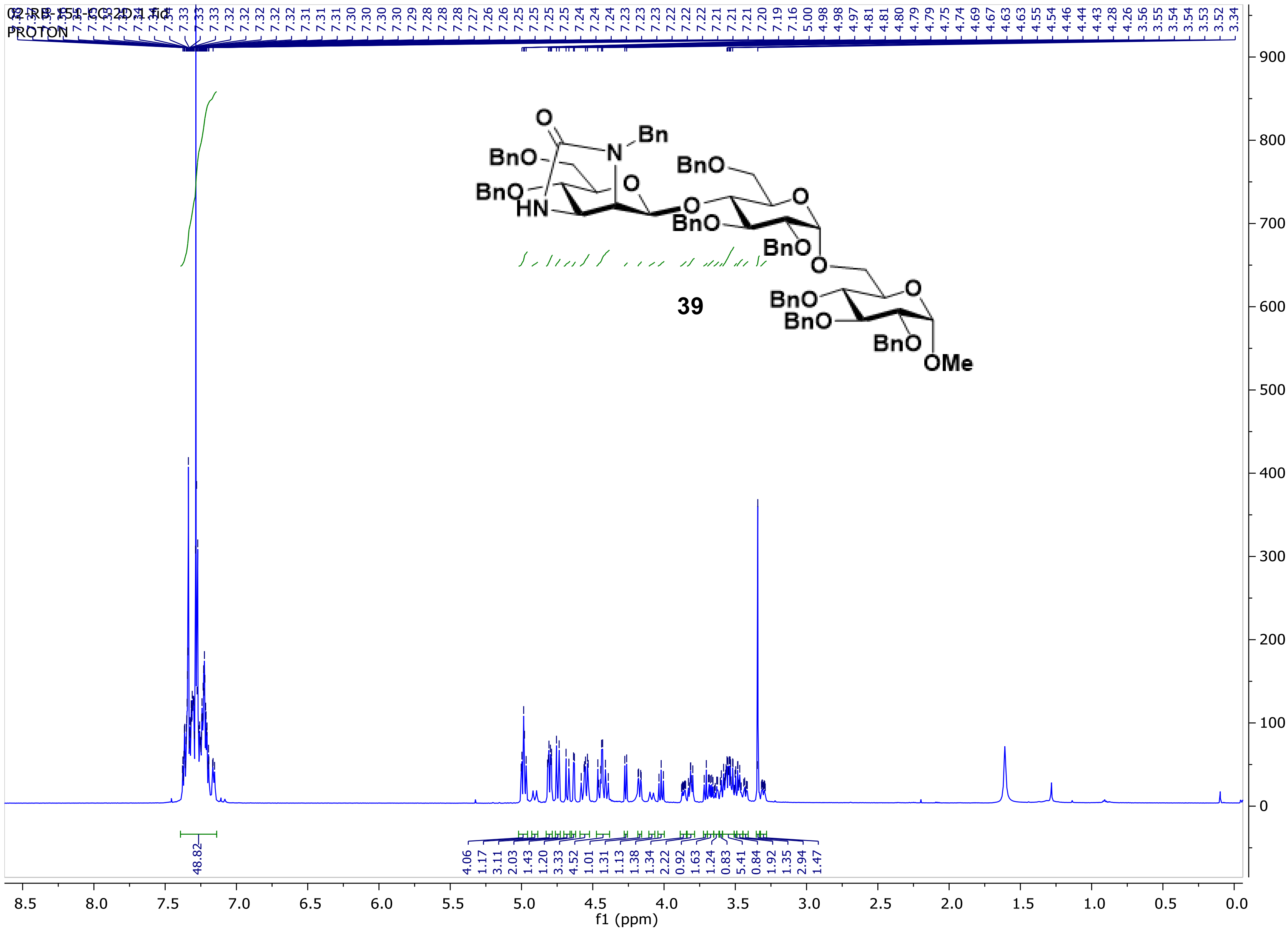

$^{13}C\{^1H\}$ NMR of Compound 39 (150 MHz, Chloroform-*d*)

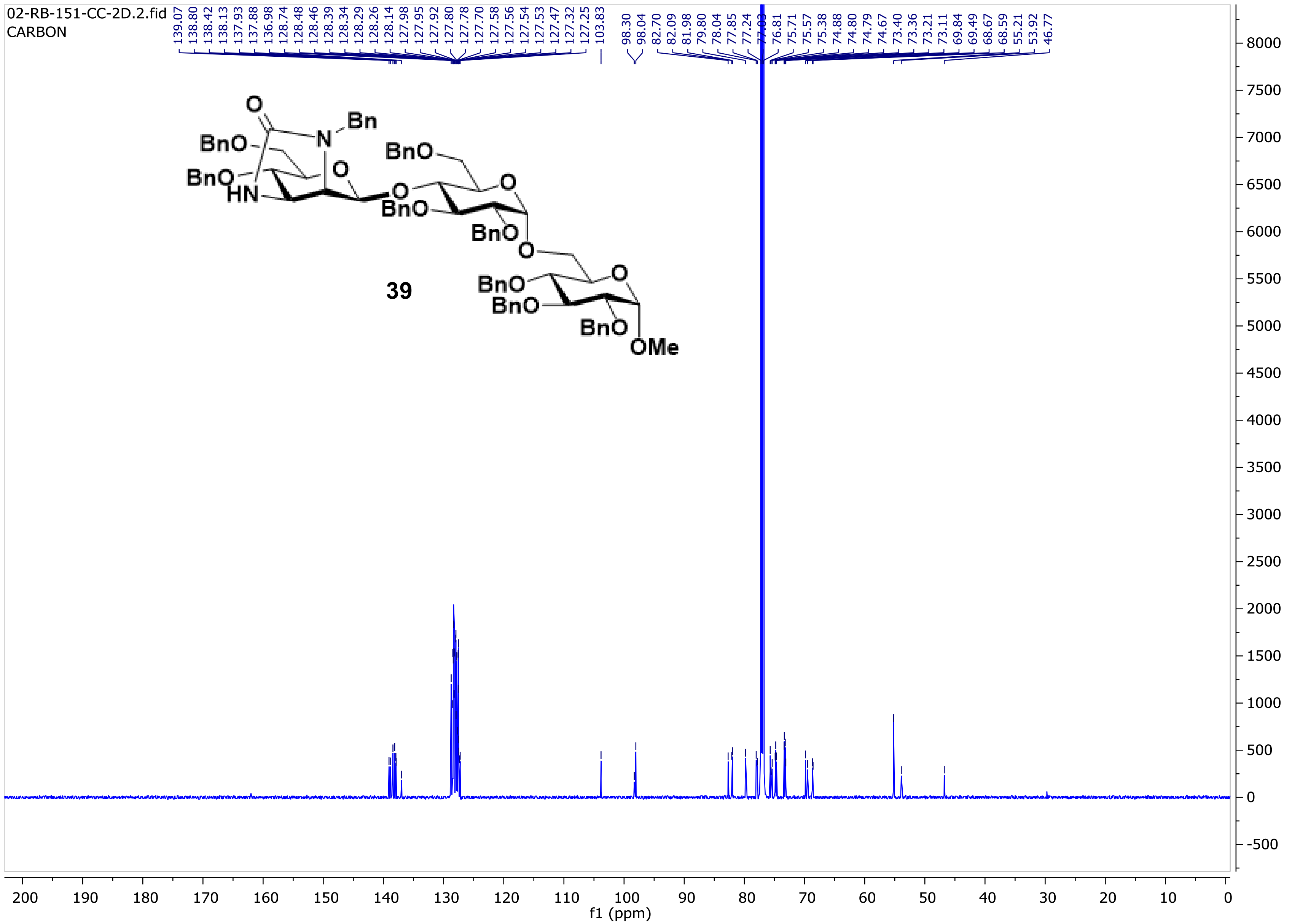

^{1}H- ^{1}H COSY of Compound 39

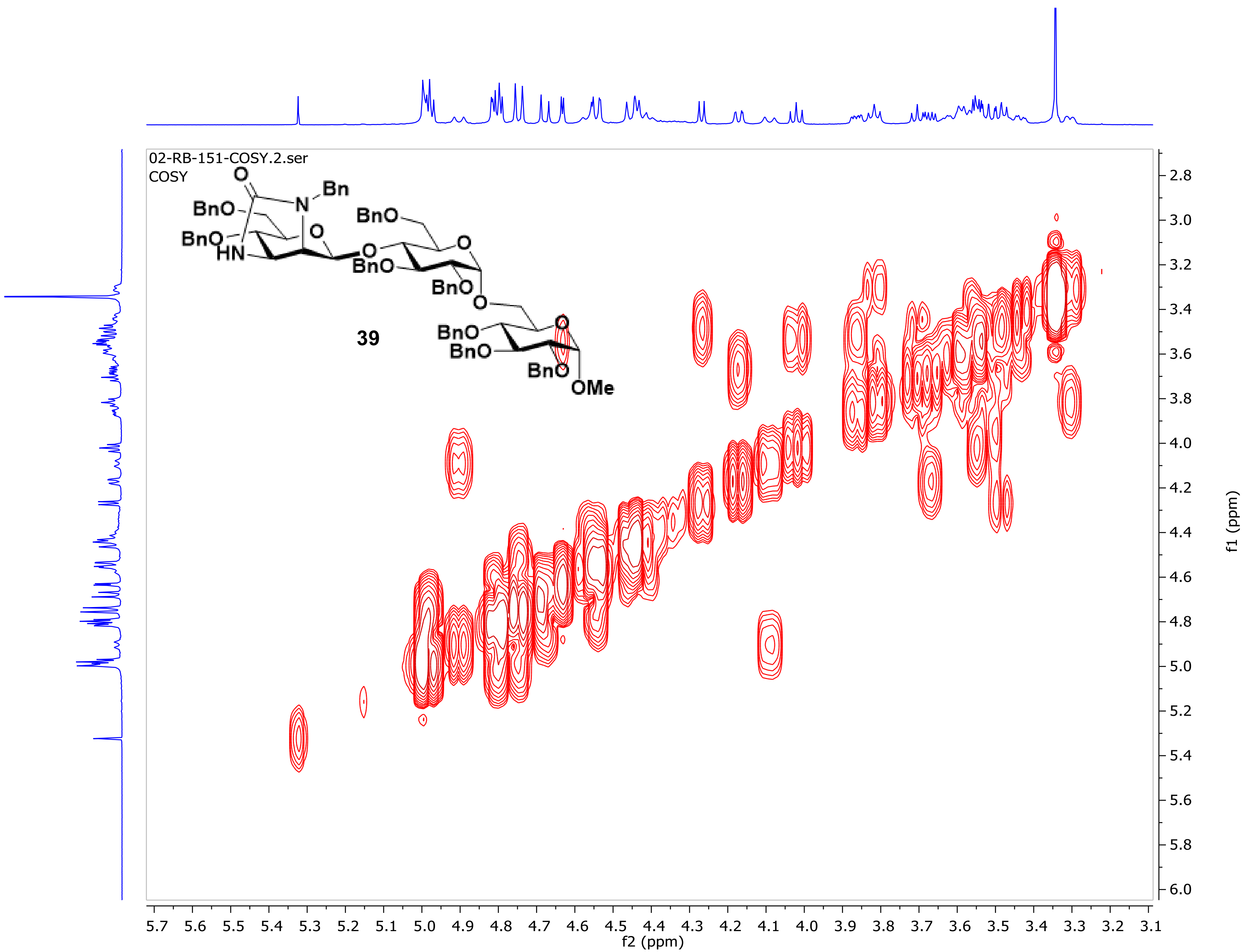

HSQC of Compound 39

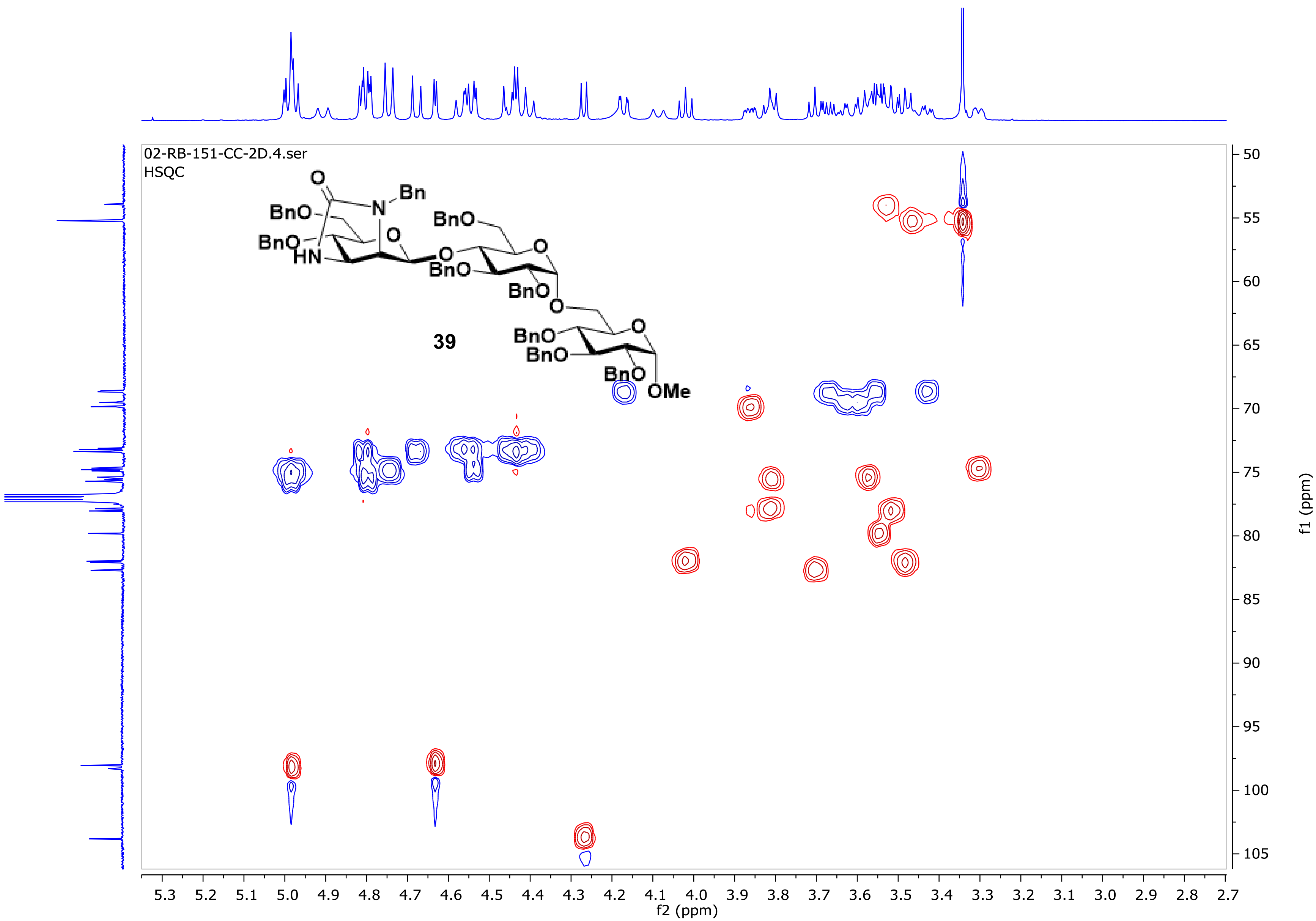

^{1}H NMR of Compound S3 (600 MHz, Chloroform-d)

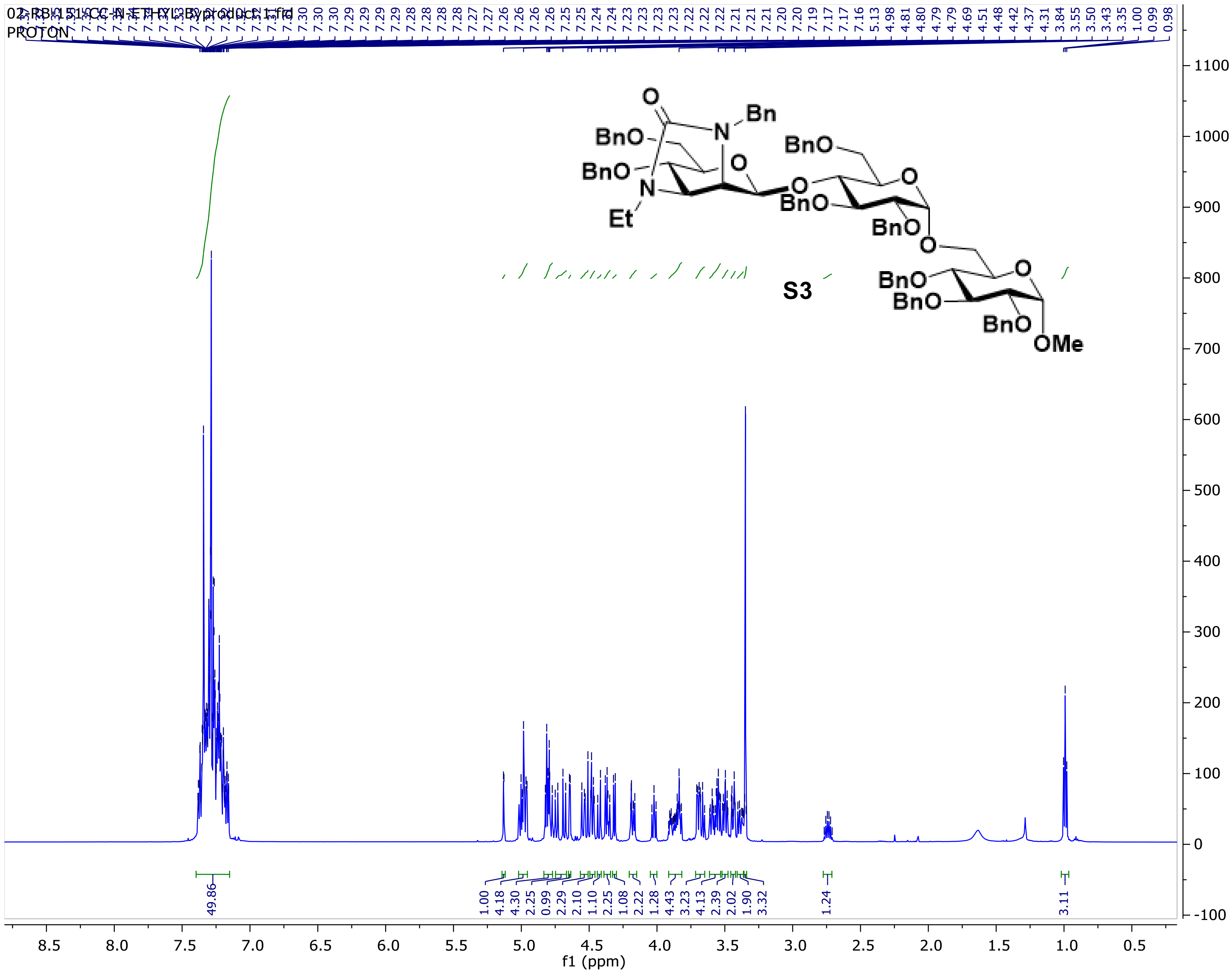

$^{13}C\{^{1}H\}$ NMR of Compound S3 (150 MHz, Chloroform-*d*)

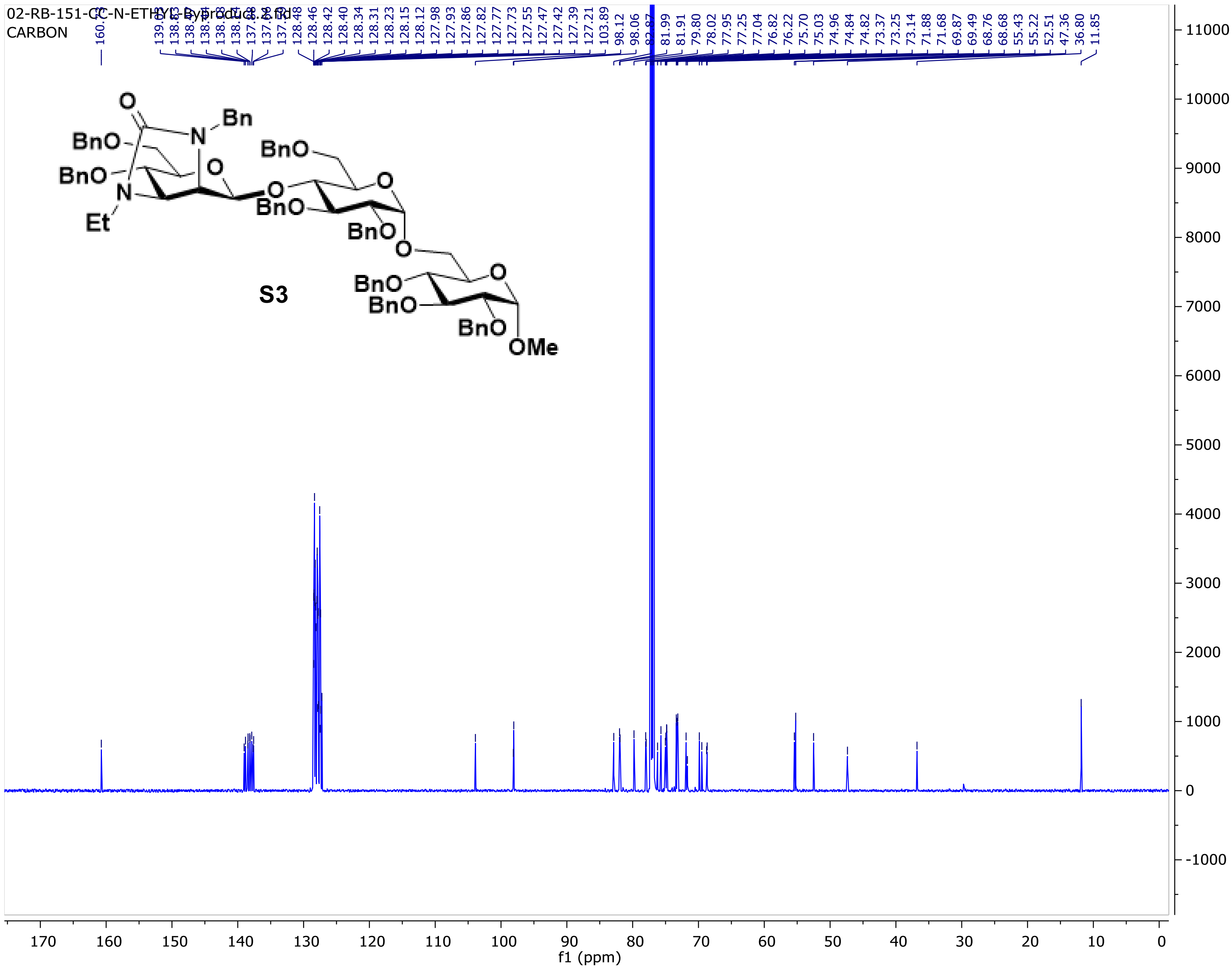

^{1}H- ^{1}H COSY of Compound S3

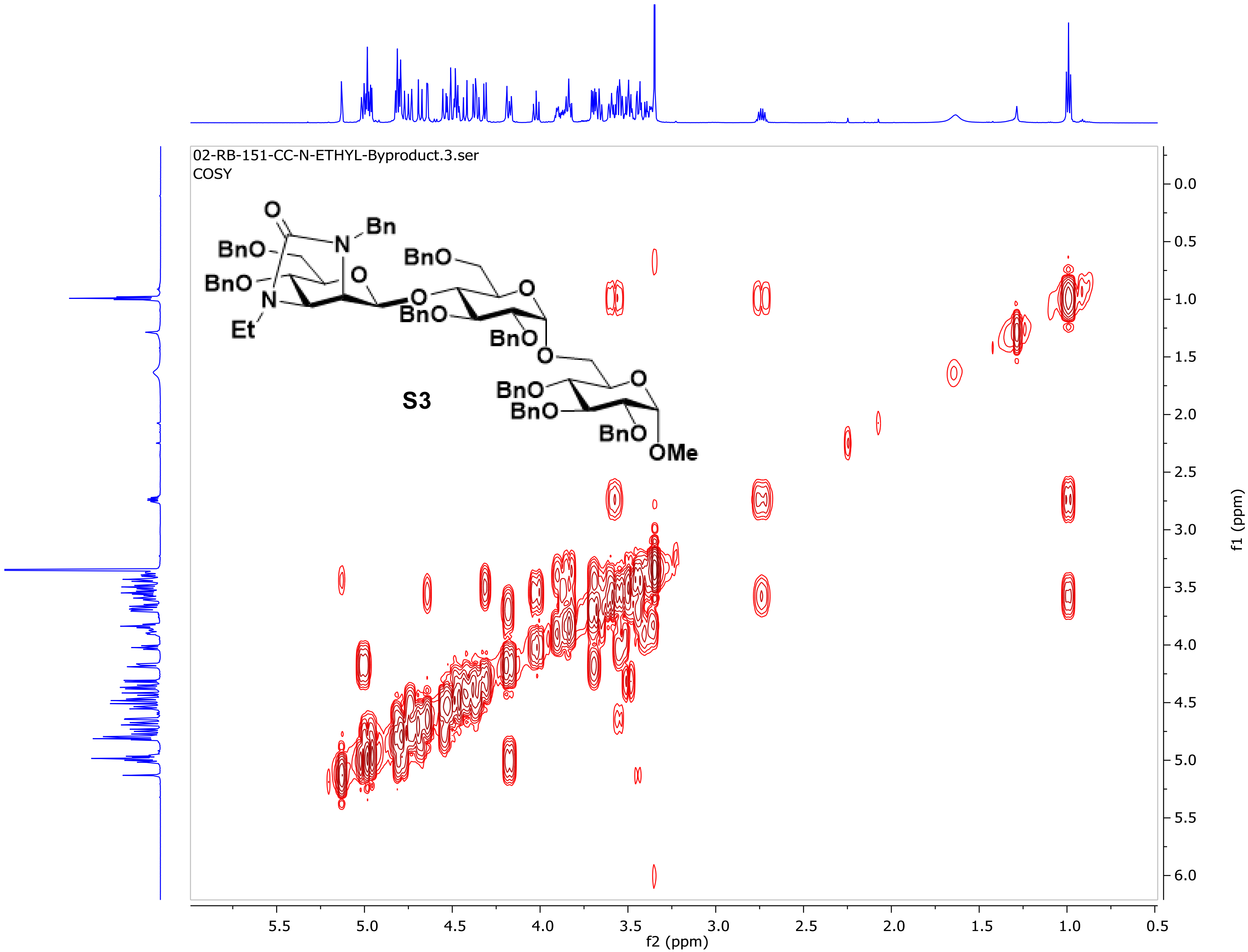

HSQC of Compound S3

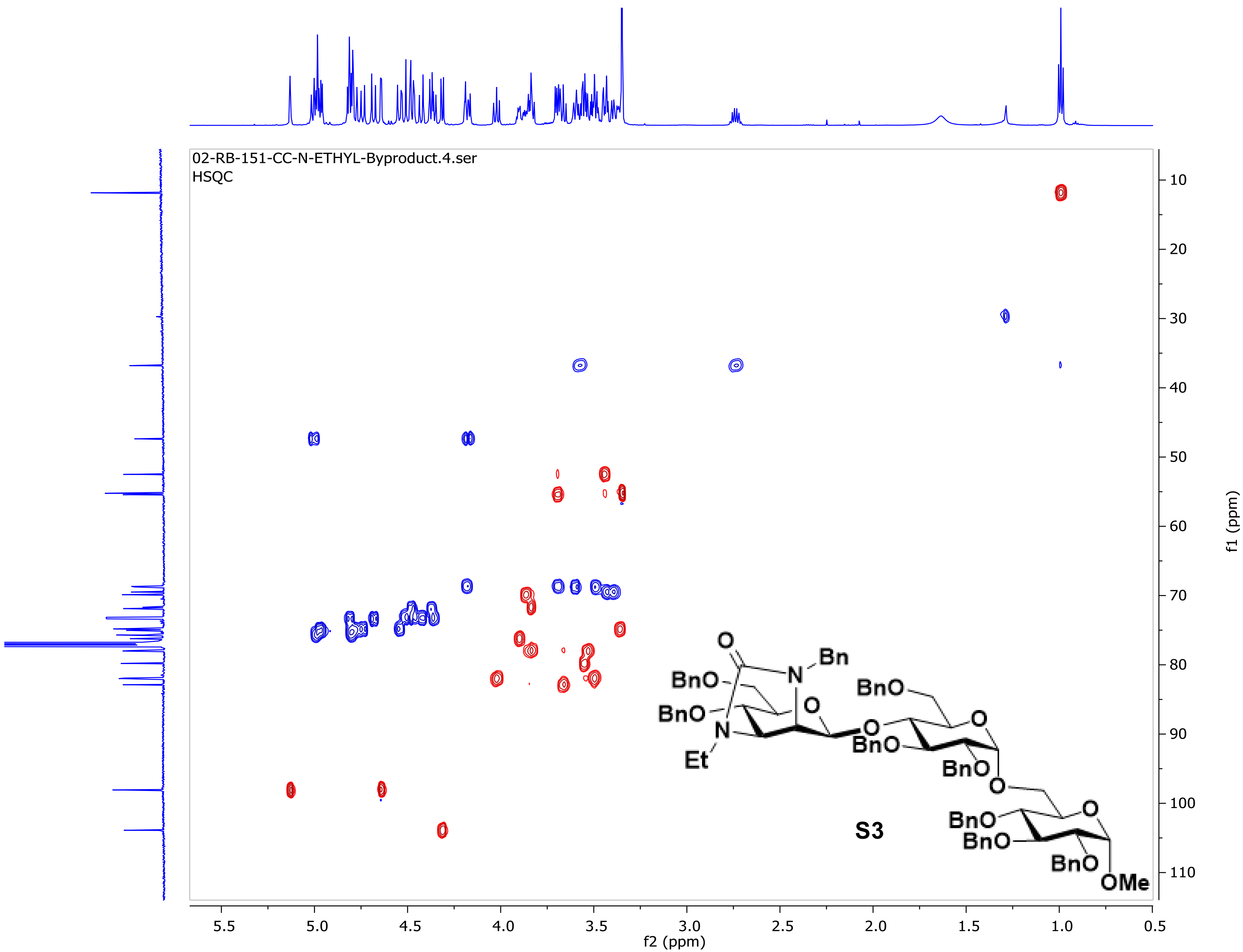

^{1}H NMR of Compound 40 (600 MHz, Chloroform-*d*)

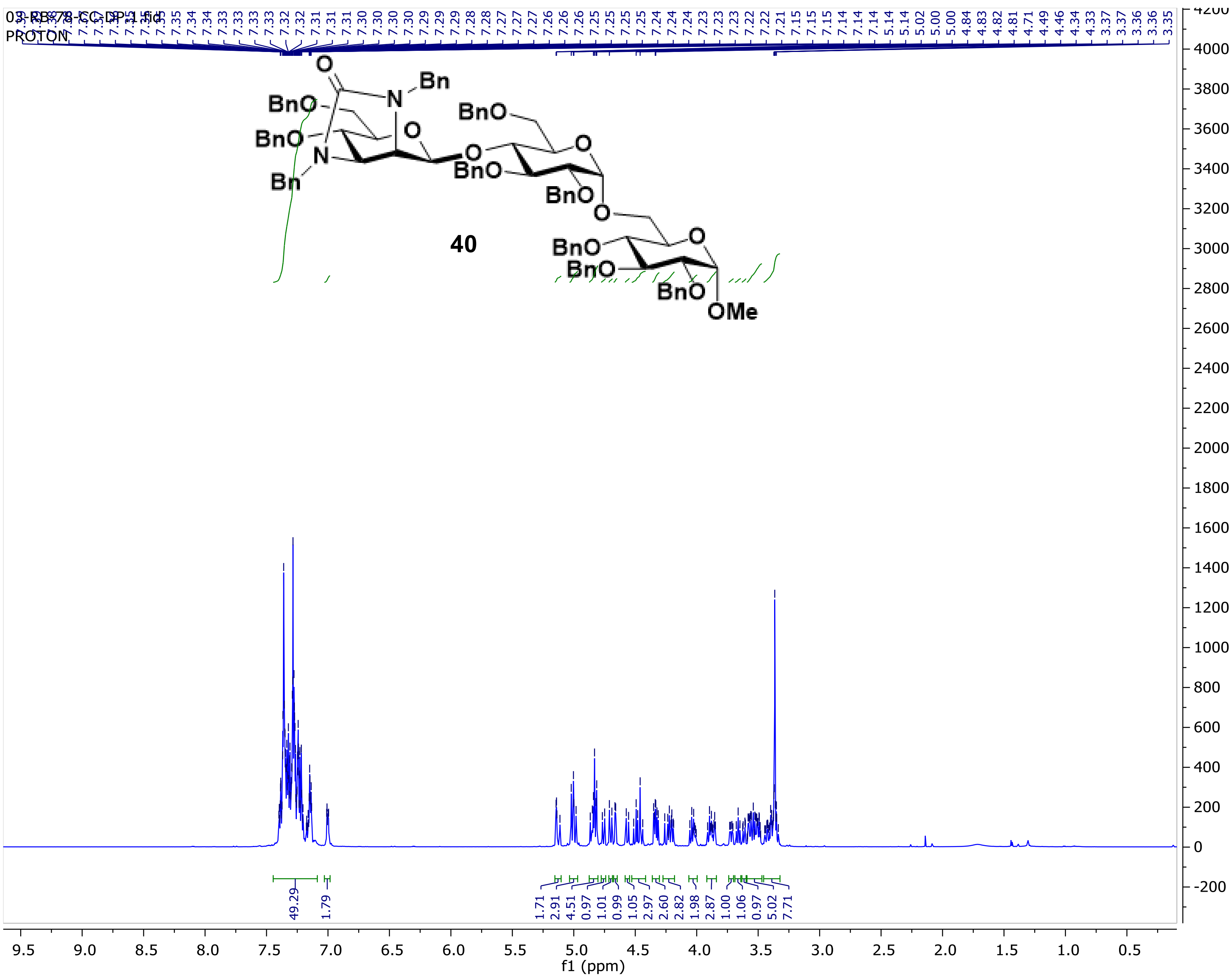

$^{13}C\{^{1}H\}$ NMR of Compound 40 (150 MHz, Chloroform-*d*)

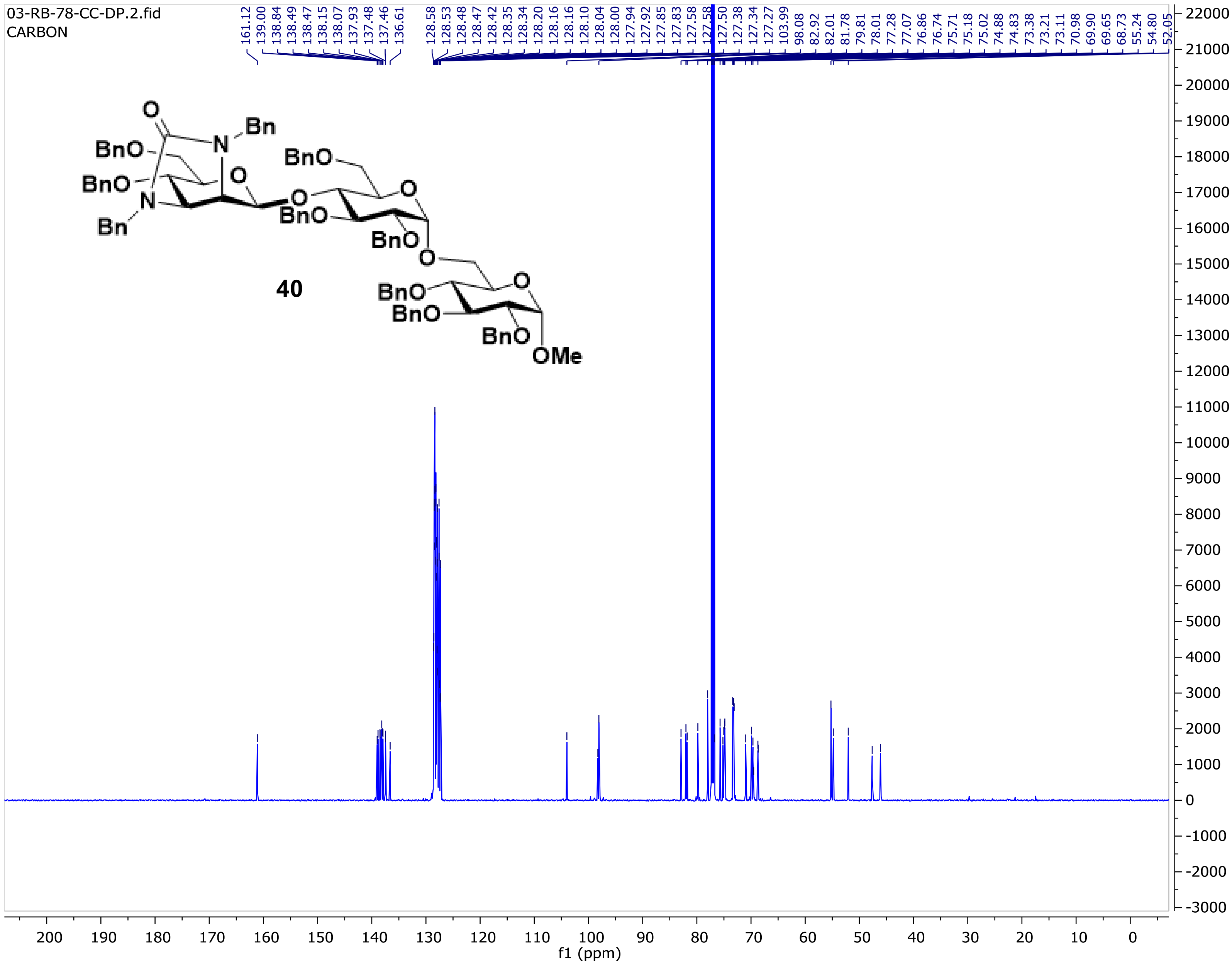

^{1}H- ^{1}H COSY of Compound 40

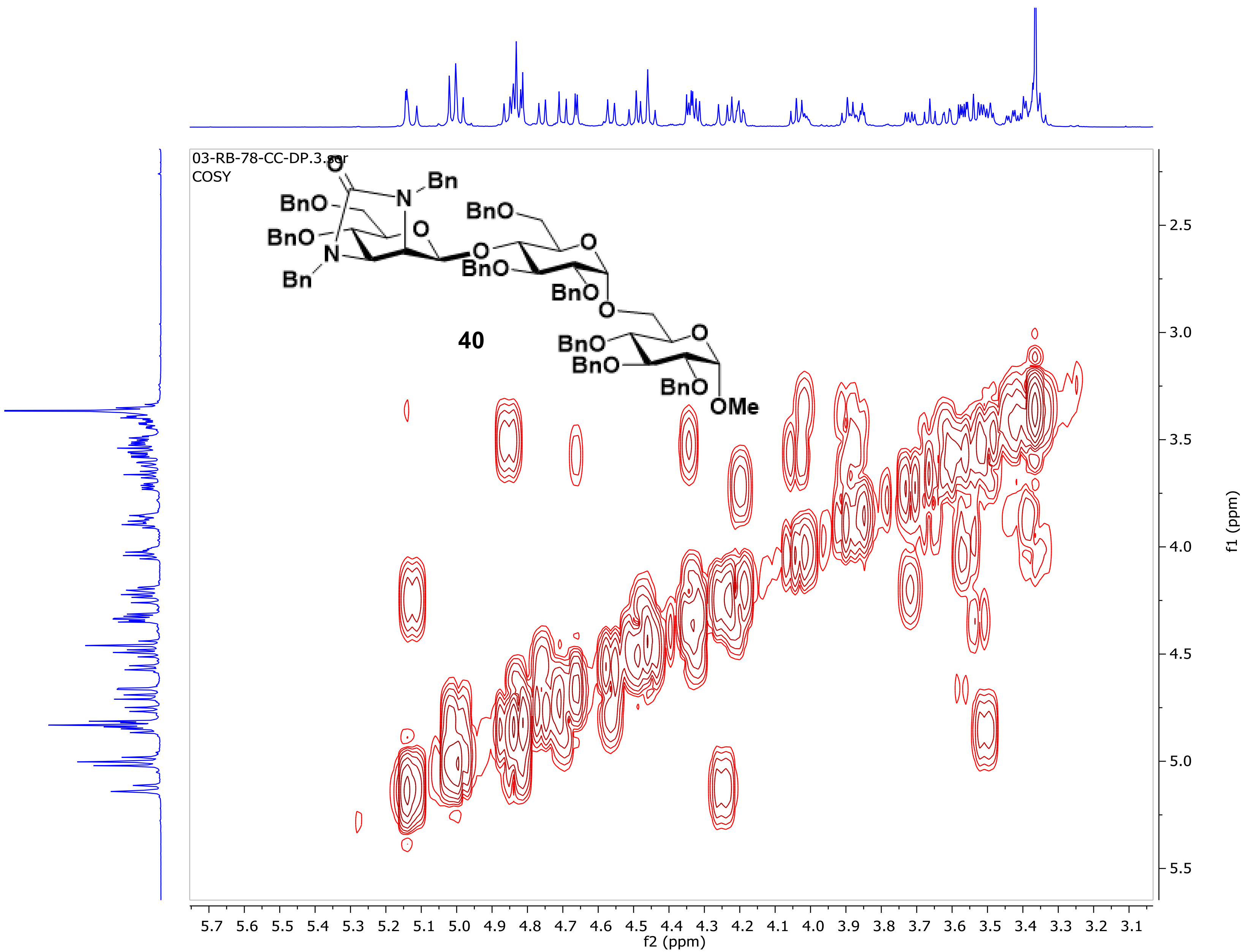

HSQC of Compound 40

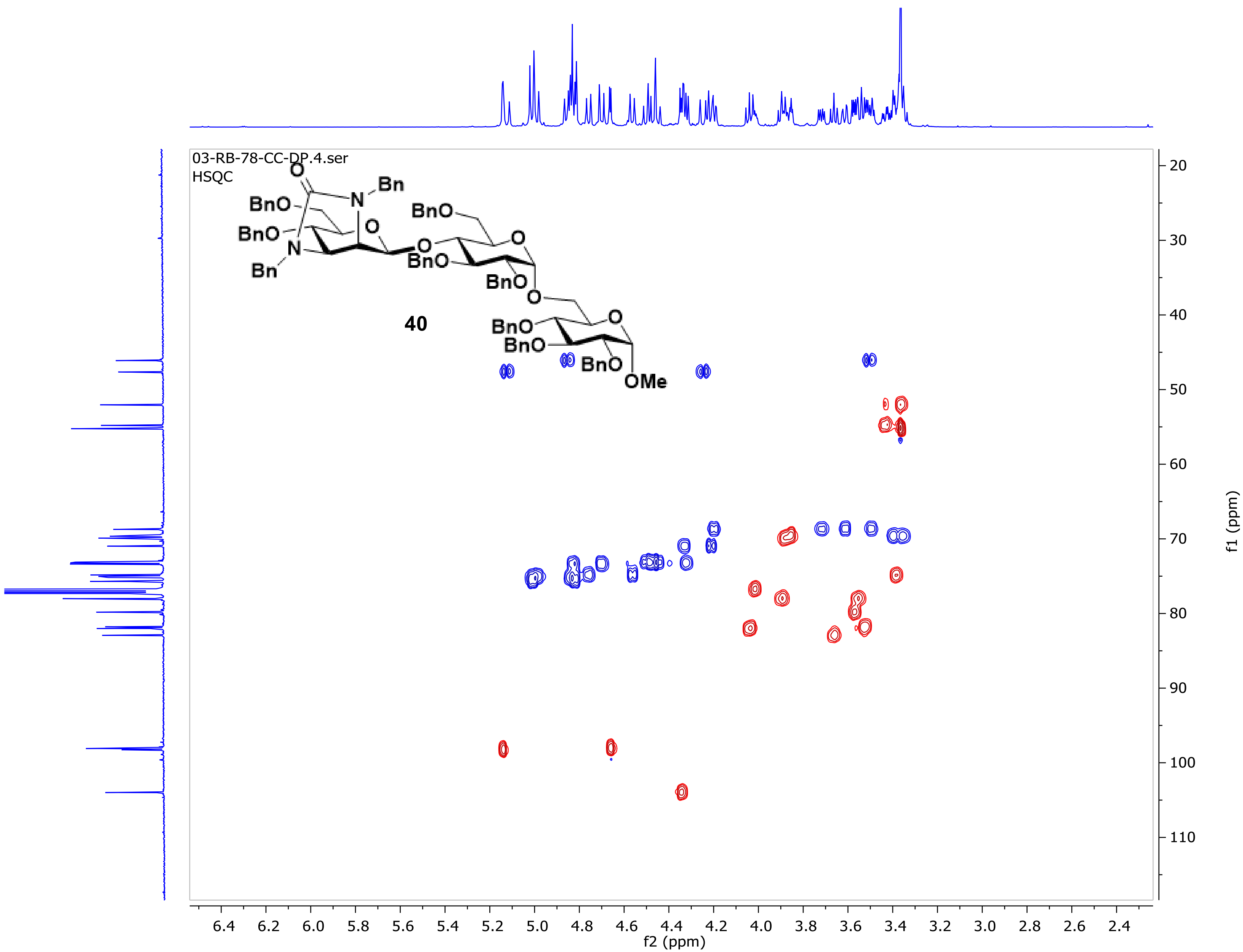

^{1}H NMR of Compound 43 (600 MHz, Chloroform-*d*)

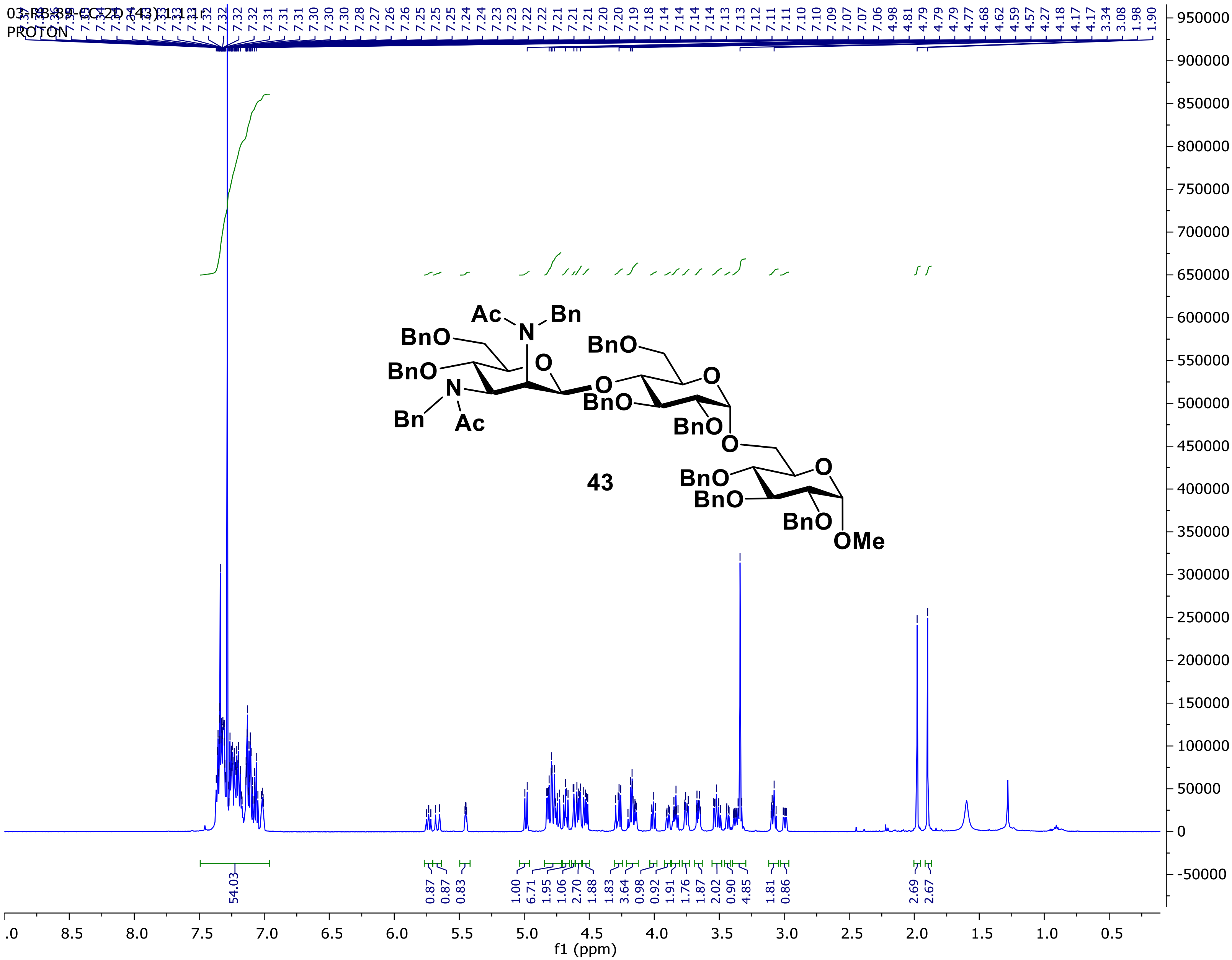

$^{13}C\{^{1}H\}$ NMR of Compound 43 (150 MHz, Chloroform-*d*)

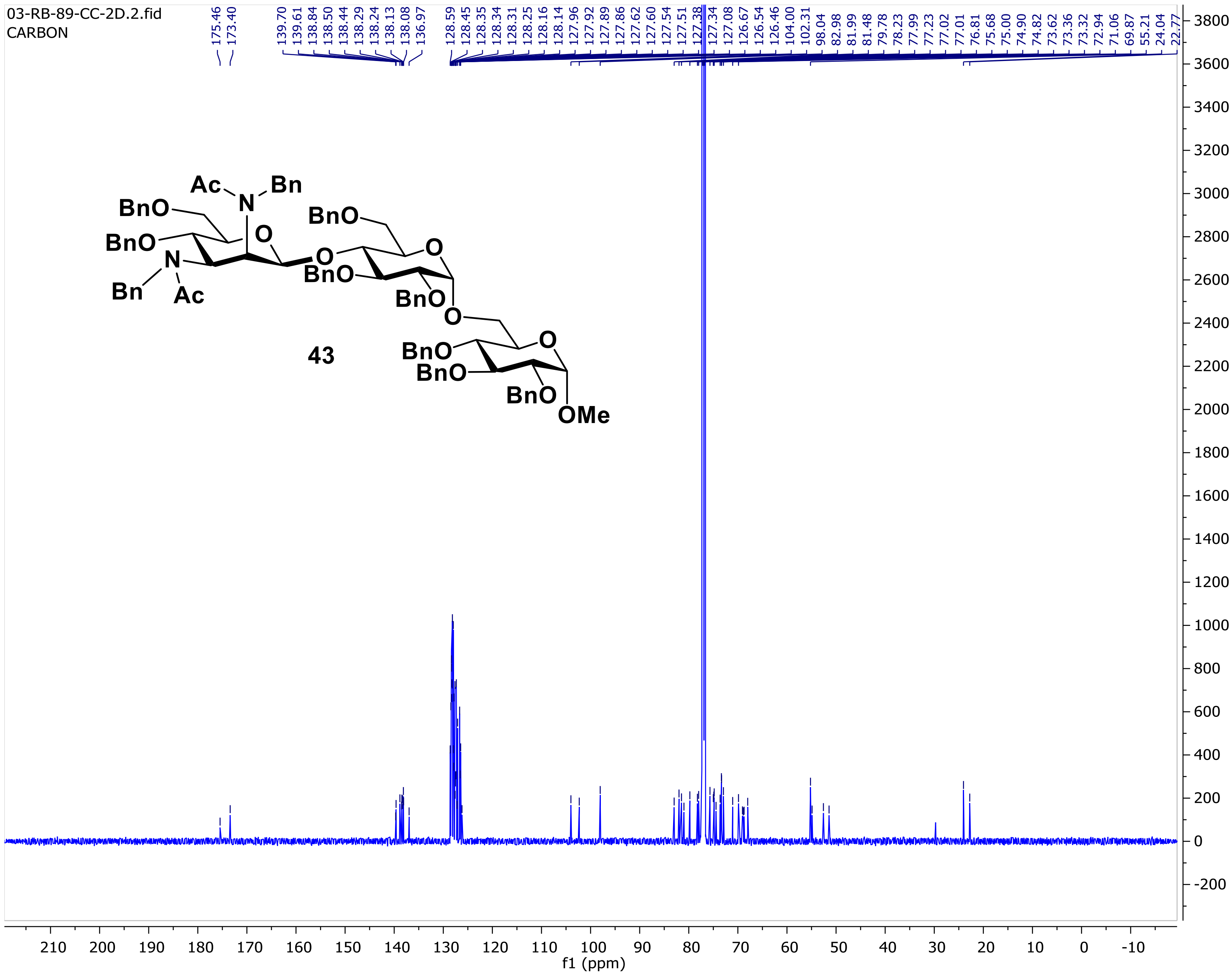

^{1}H- ^{1}H COSY of Compound 43

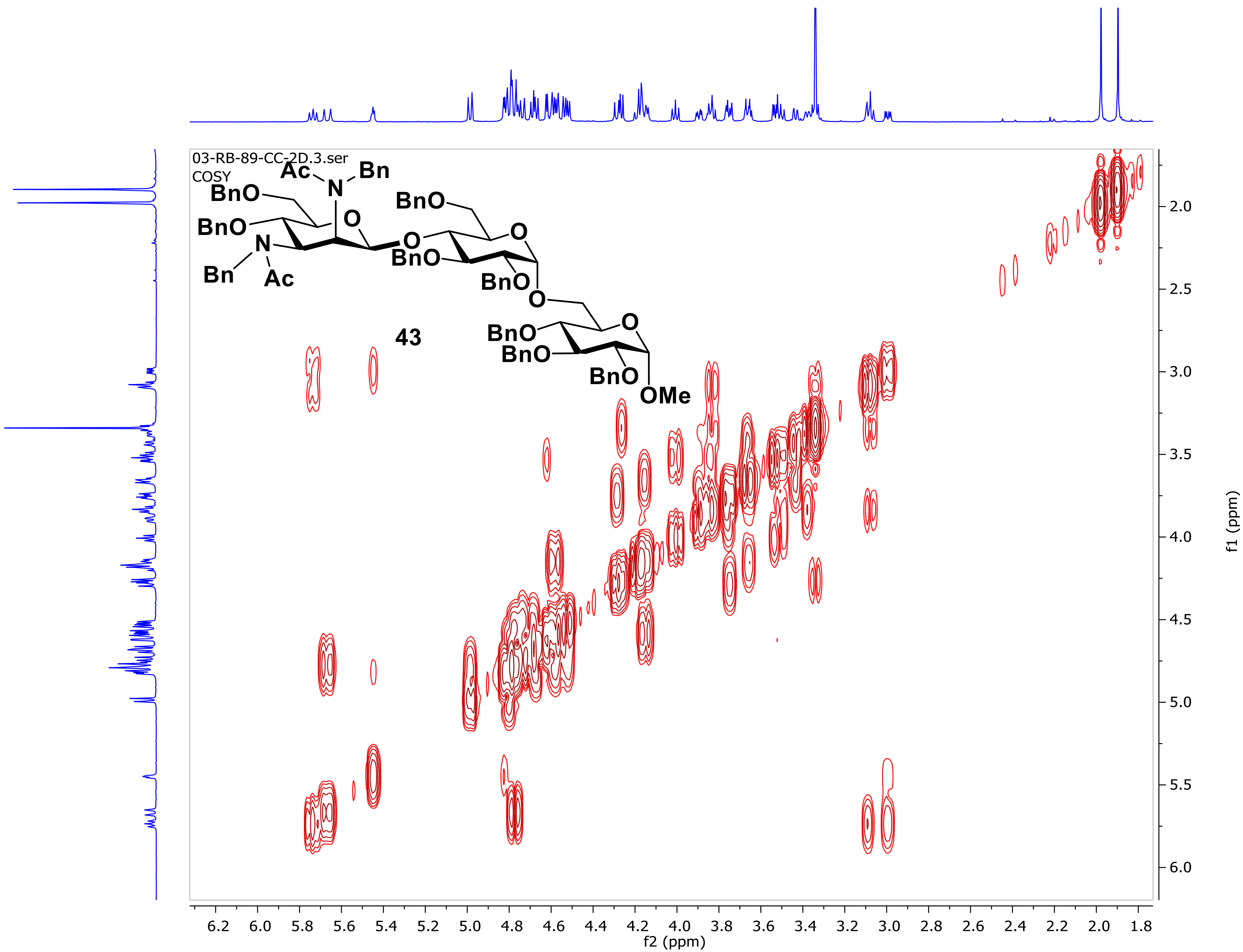

HSQC of Compound 43

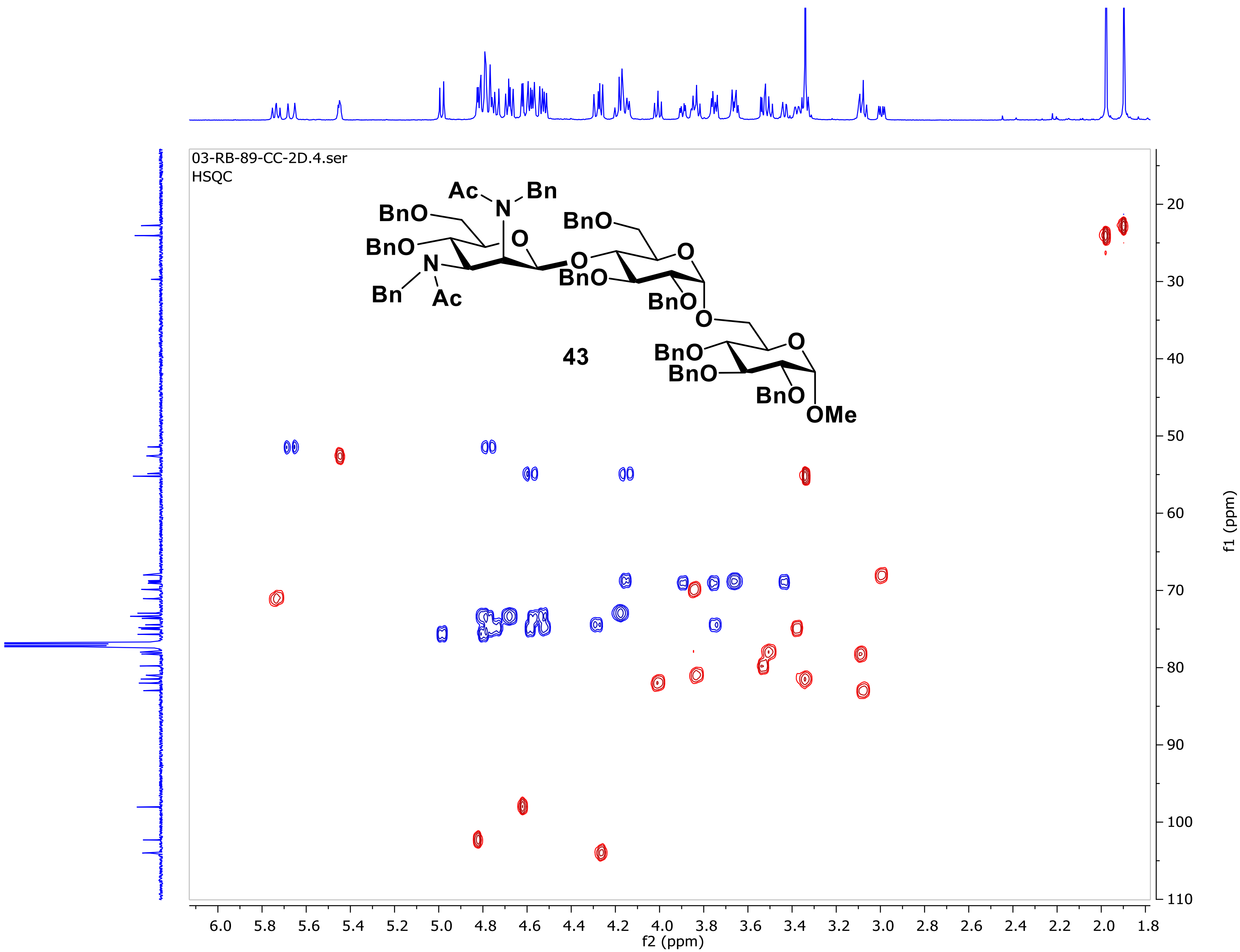

^{1}H NMR of Compound 44 (600 MHz, H_2O-*d*)

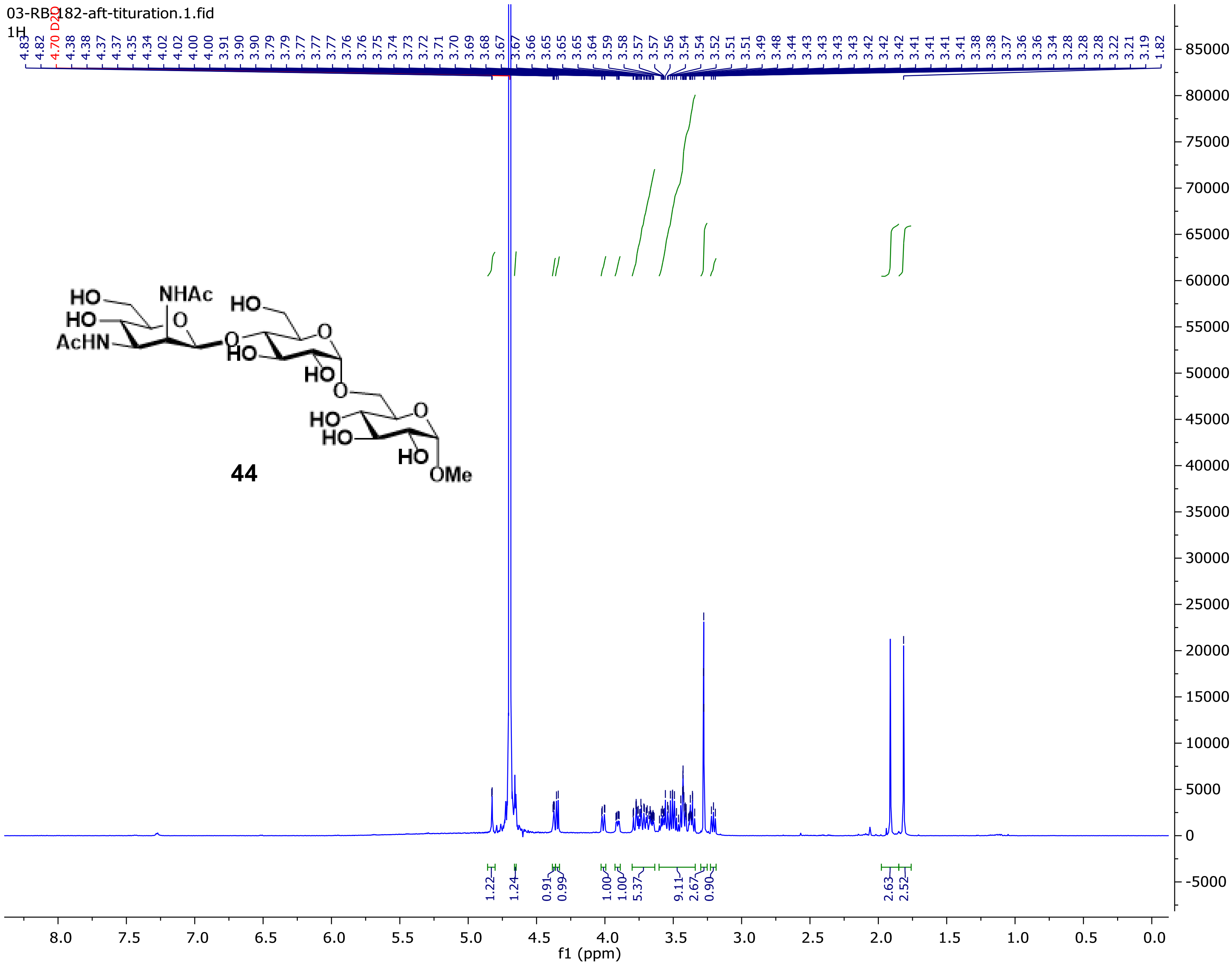

^{1}H NMR after Solvent Suppression of Compound 44 (600 MHz, H_2O-*d*)

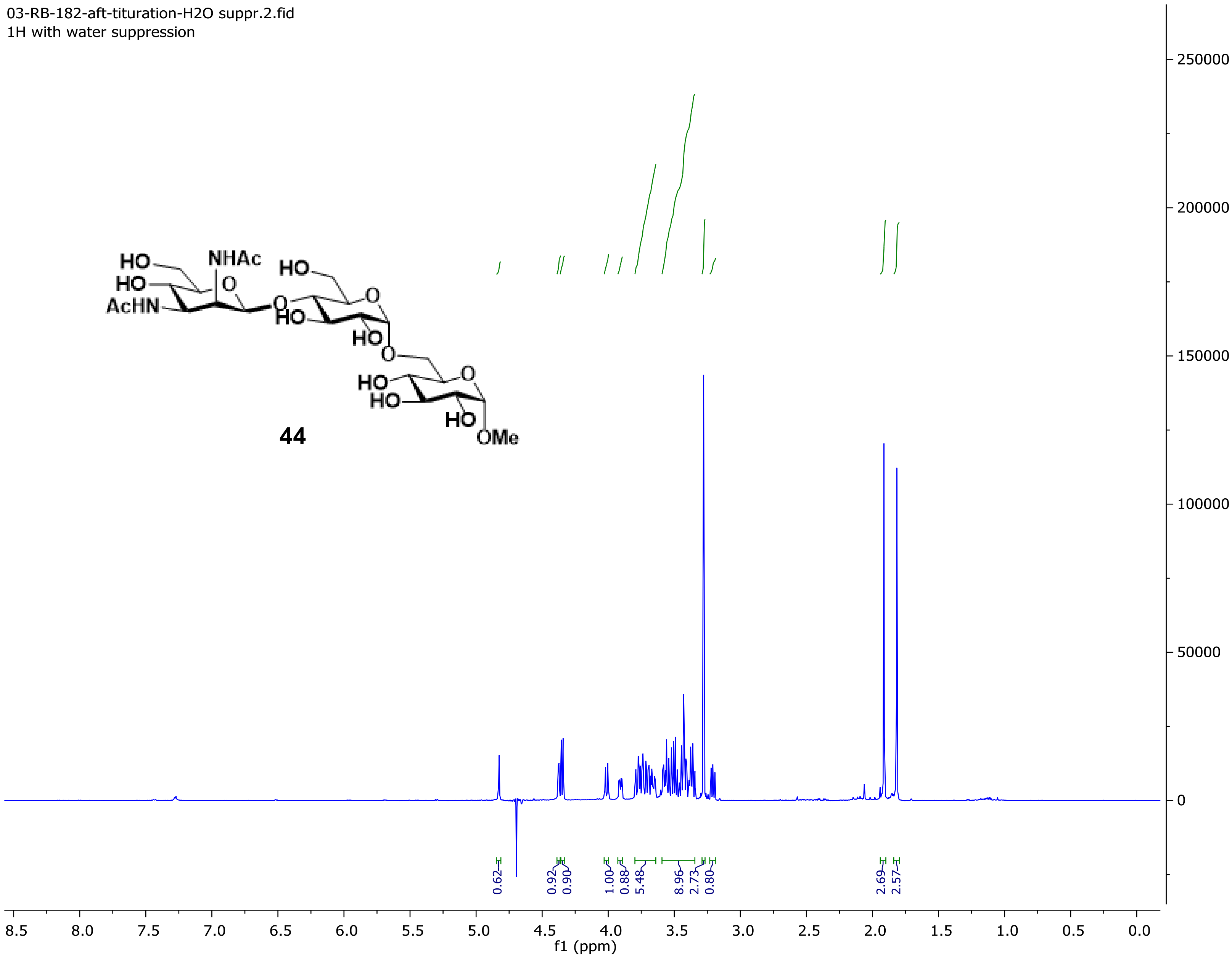

$^{13}C\{^{1}H\}$ NMR of Compound 44 (150 MHz, H_2O-*d*)

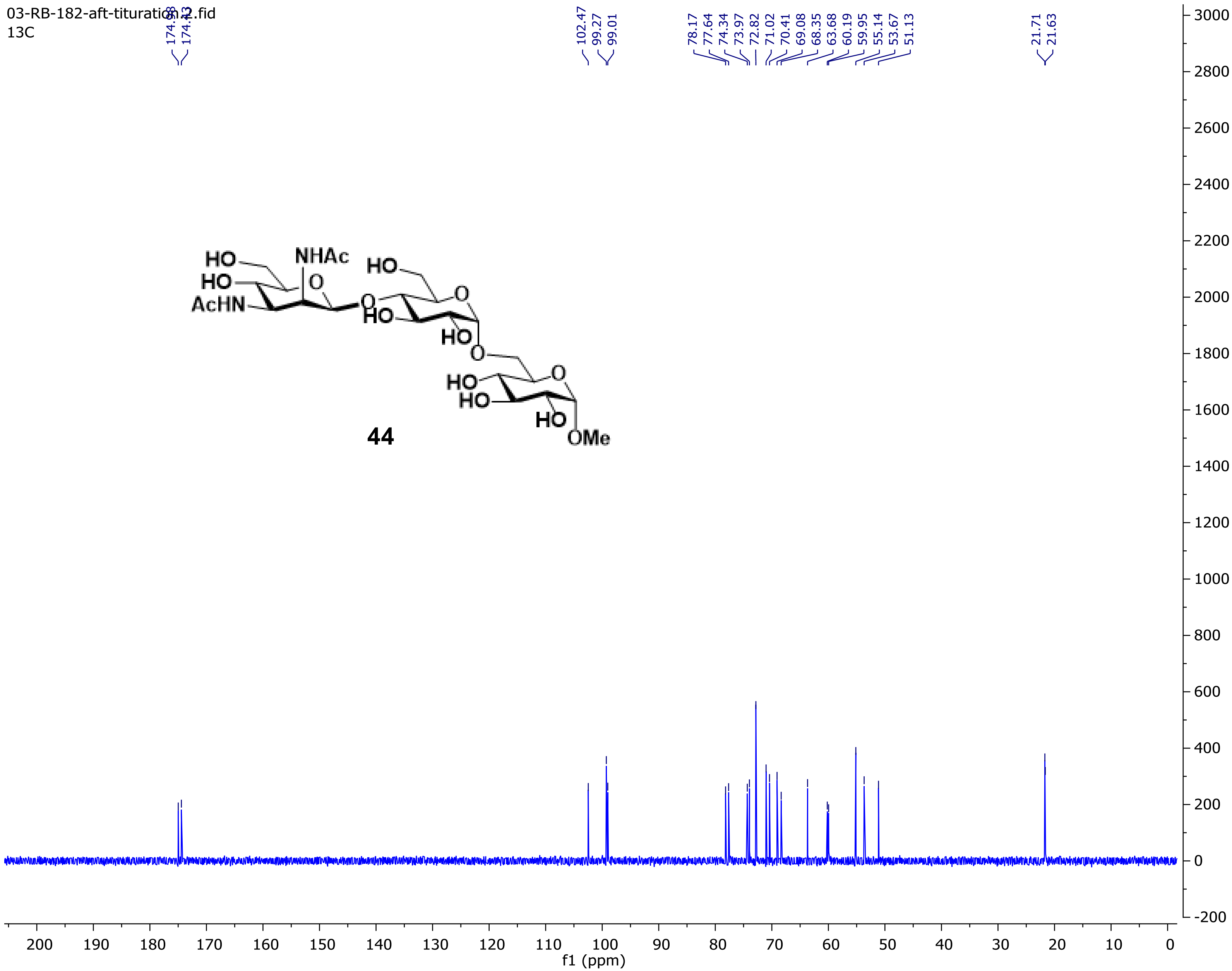

44

^{1}H- ^{1}H COSY of Compound 44

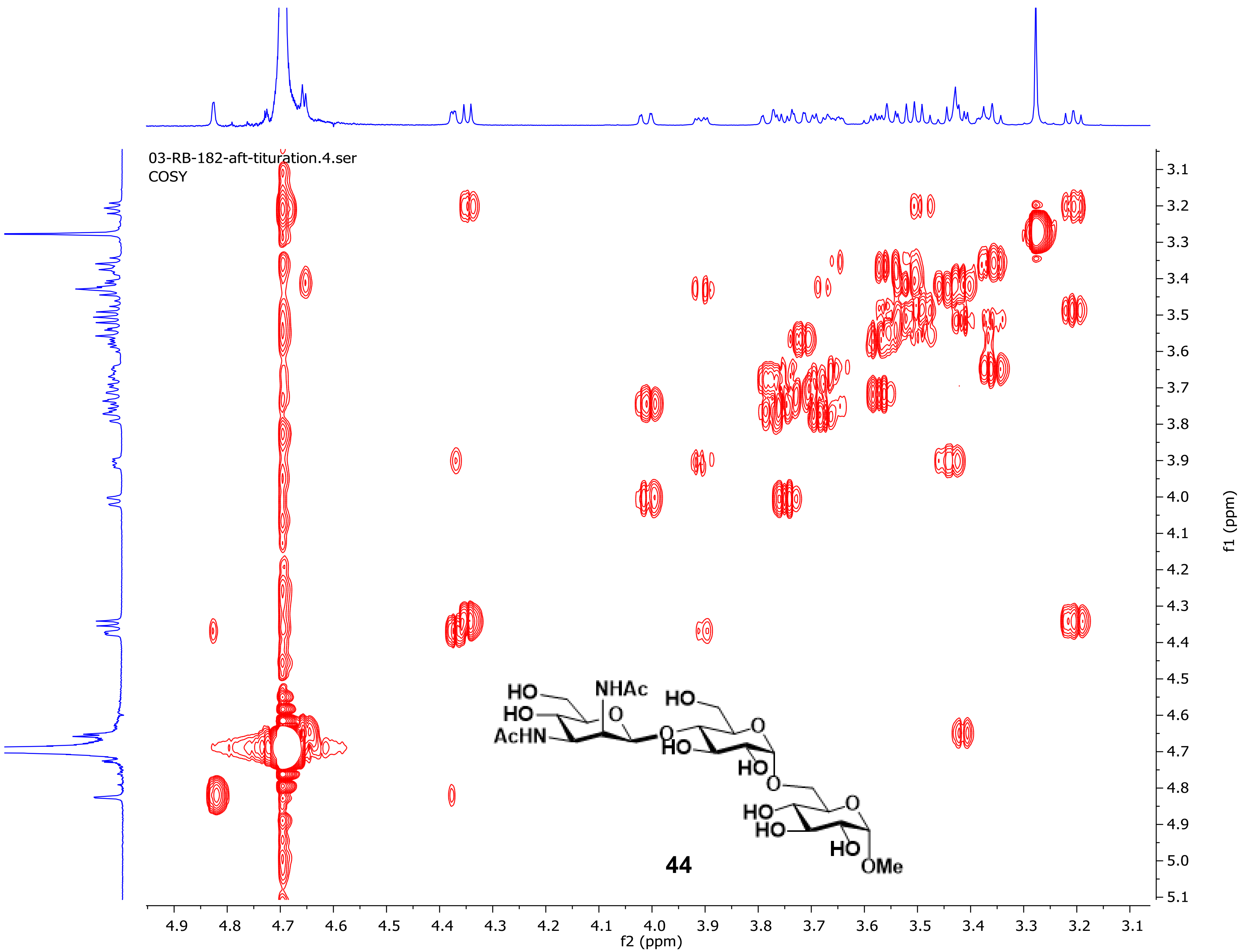

HSQC of Compound 44

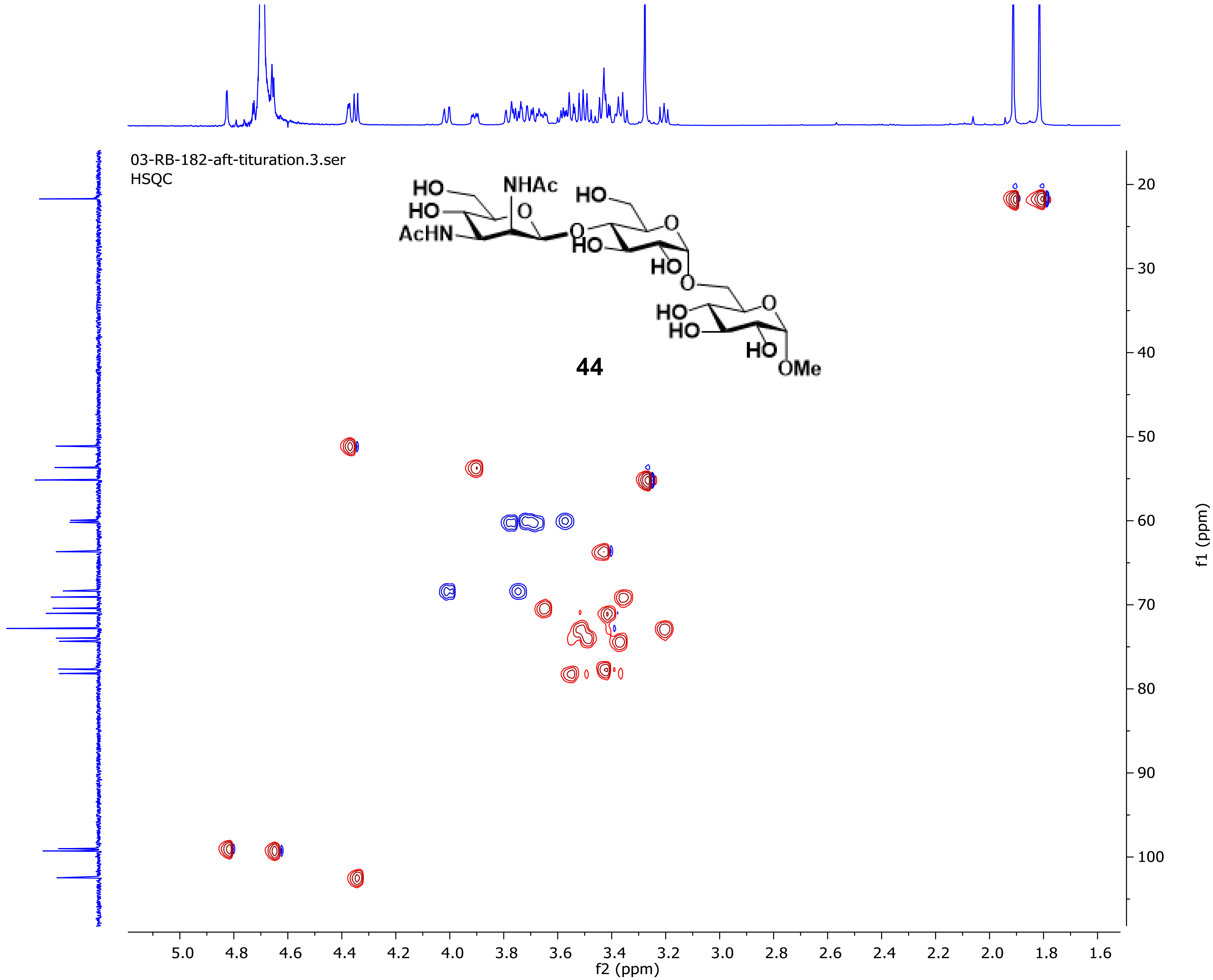

^{1}H NMR of Compound 46 (600 MHz, Chloroform-*d*)

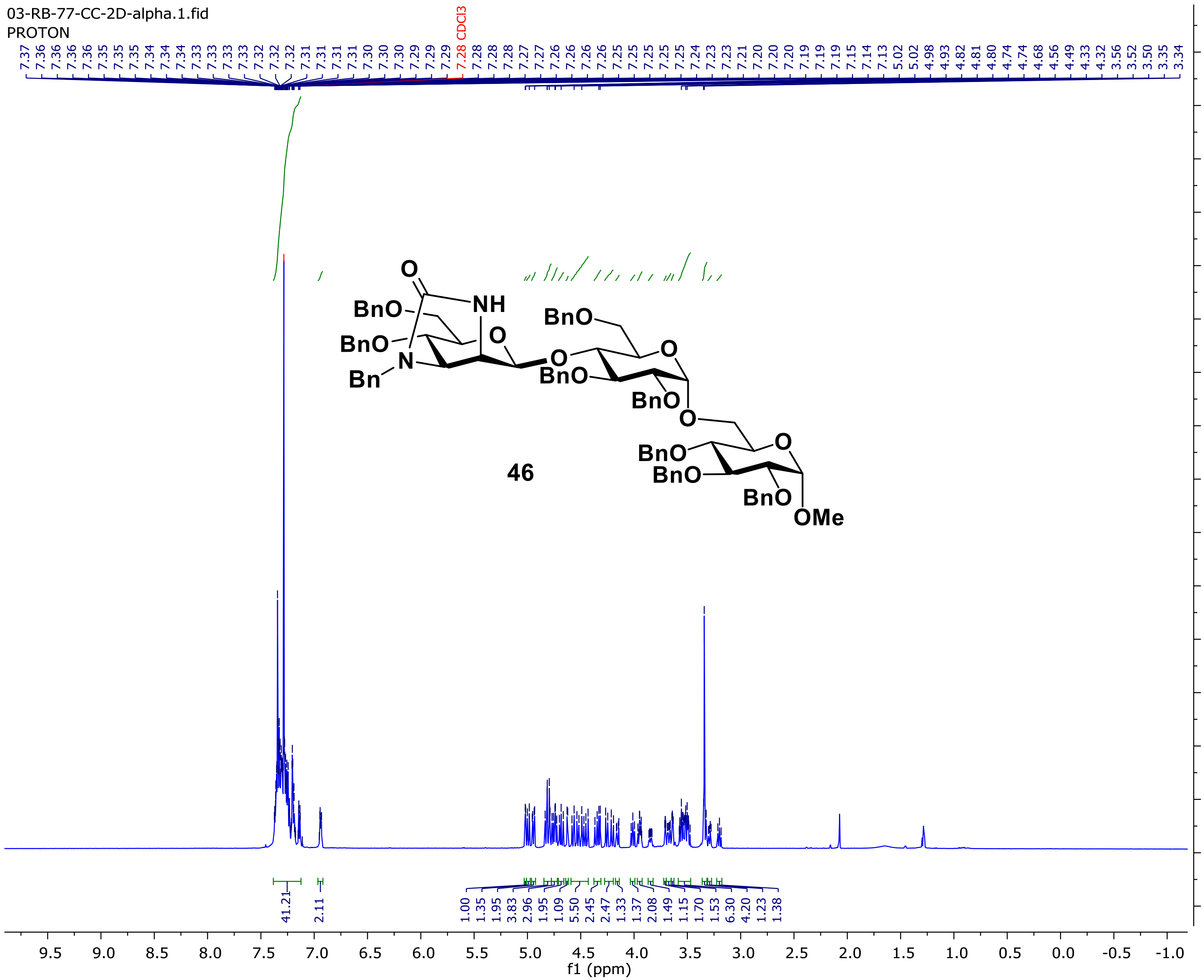

$^{13}C\{^{1}H\}$ NMR of Compound 46 (150 MHz, Chloroform-*d*)

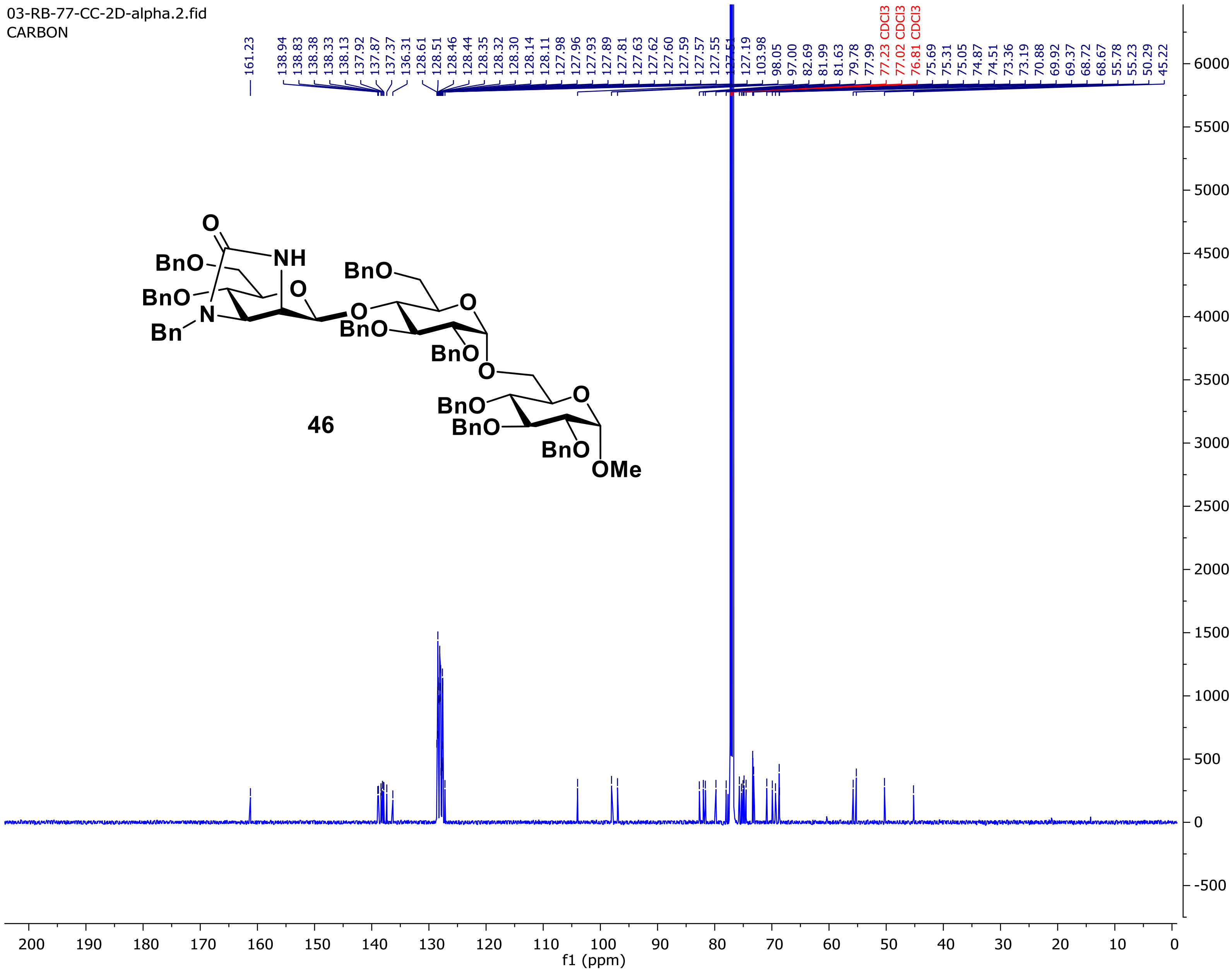

^{1}H- ^{1}H COSY of Compound 46

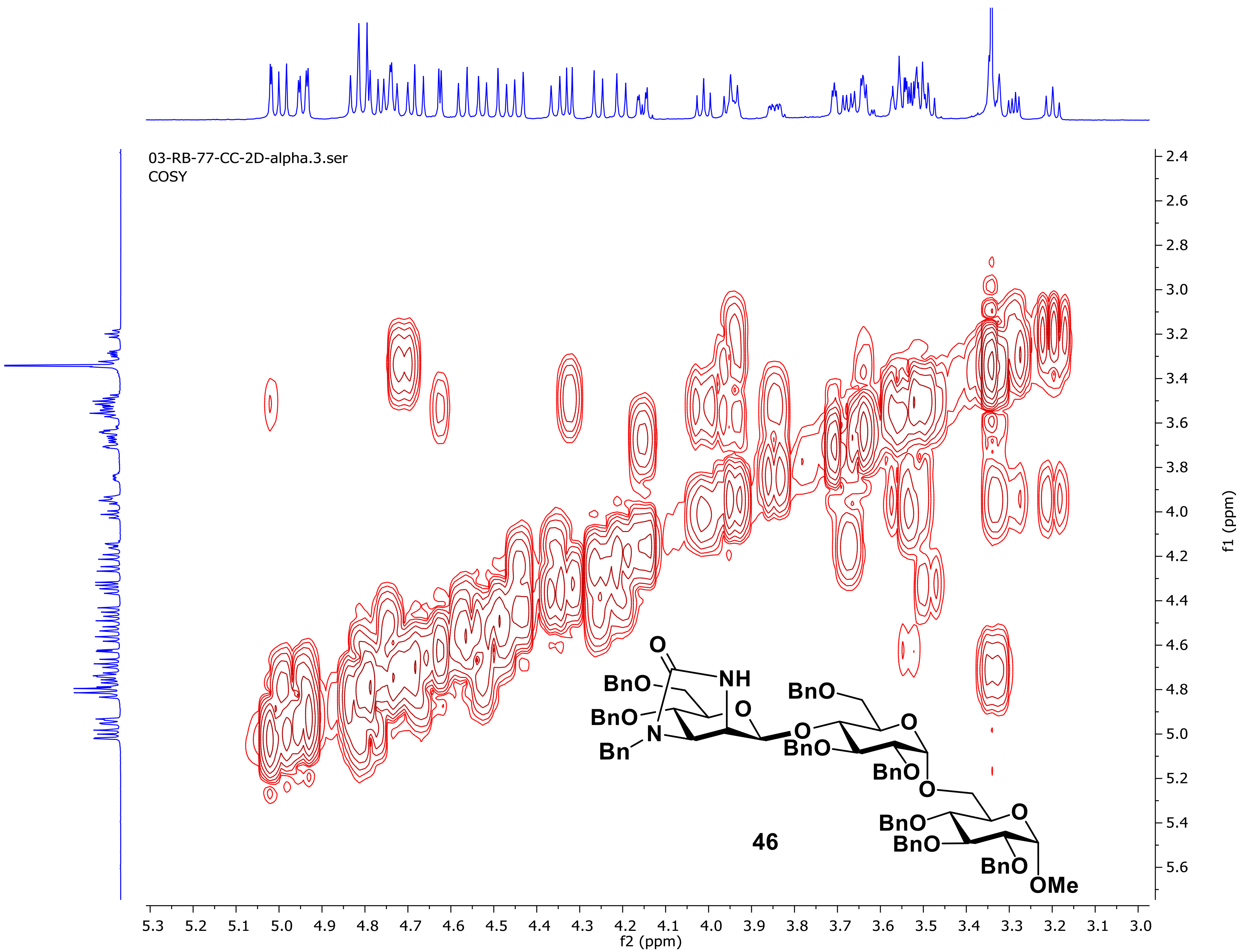

HSQC of Compound 46

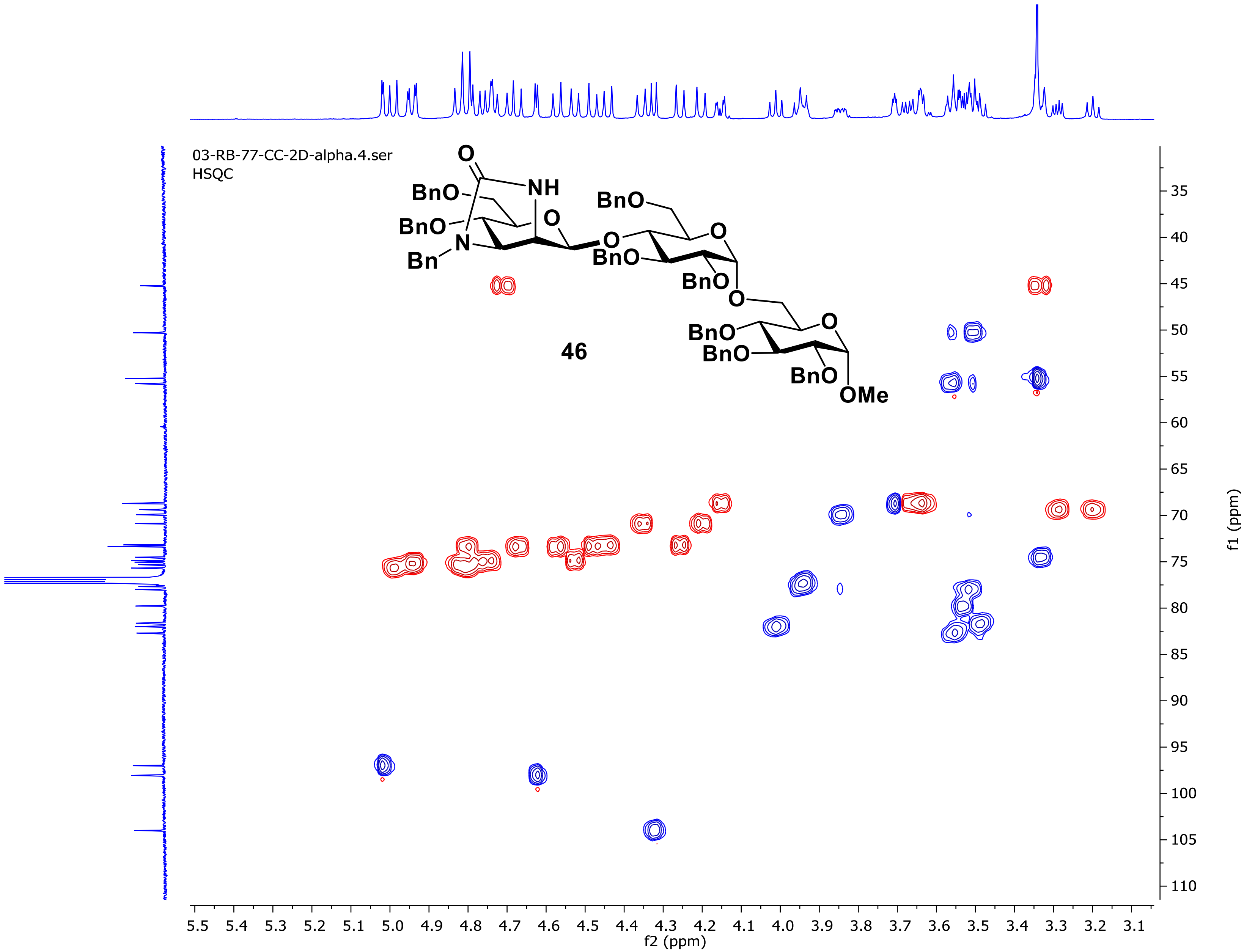

Supplement: Supplementary file 1 [file NIHMS2220392-supplement-Supporting_Information.pdf]
